# Exposure of volunteers to microgravity by dry immersion bed over 21 days results in gene expression changes and adaptation of T cells

**DOI:** 10.1126/sciadv.adg1610

**Published:** 2023-08-25

**Authors:** Carlos J. Gallardo-Dodd, Christian Oertlin, Julien Record, Rômulo G. Galvani, Christian Sommerauer, Nikolai V. Kuznetsov, Evangelos Doukoumopoulos, Liaqat Ali, Mariana M. S. Oliveira, Christina Seitz, Mathias Percipalle, Tijana Nikić, Anastasia A. Sadova, Sofia M. Shulgina, Vjacheslav A. Shmarov, Olga V. Kutko, Daria D. Vlasova, Kseniya D. Orlova, Marina P. Rykova, John Andersson, Piergiorgio Percipalle, Claudia Kutter, Sergey A. Ponomarev, Lisa S. Westerberg

**Affiliations:** ^1^ Department of Microbiology, Tumor and Cell Biology, Karolinska Institutet, Stockholm, Sweden.; ^2^ Science for Life Laboratory, Karolinska Institutet, Stockholm, Sweden.; ^3^ Laboratory of Bioinformatics and Computational Biology, Division of Experimental and Translational Research, Brazilian National Cancer Institute (INCA), Rio de Janeiro, RJ, Brazil.; ^4^ Universidade Veiga de Almeida, Rio de Janeiro, Brazil.; ^5^ Laboratory for Thymus Research (LPT), Oswaldo Cruz Institute, Oswaldo Cruz Foundation (FIOCRUZ), Rio de Janeiro, Brazil.; ^6^ Russian Federation State Research Center Institute of Biomedical Problems RAS, Moscow, Russia.; ^7^ Program in Biology, Division of Science and Mathematics, New York University Abu Dhabi (NYUAD), Abu Dhabi, United Arab Emirates.; ^8^ Core Technology Platform, NYUAD, Abu Dhabi, United Arab Emirates.; ^9^ Institute of Environmental Medicine, Karolinska Institutet, Stockholm, Sweden.; ^10^ Center for Genomics and Systems Biology, NYUAD, Abu Dhabi, United Arab Emirates.; ^11^ Department of Molecular Biosciences, The Wenner-Gren Institute, Stockholm University, Stockholm, Sweden.

## Abstract

The next steps of deep space exploration are manned missions to Moon and Mars. For safe space missions for crew members, it is important to understand the impact of space flight on the immune system. We studied the effects of 21 days dry immersion (DI) exposure on the transcriptomes of T cells isolated from blood samples of eight healthy volunteers. Samples were collected 7 days before DI, at day 7, 14, and 21 during DI, and 7 days after DI. RNA sequencing of CD3^+^ T cells revealed transcriptional alterations across all time points, with most changes occurring 14 days after DI exposure. At day 21, T cells showed evidence of adaptation with a transcriptional profile resembling that of 7 days before DI. At 7 days after DI, T cells again changed their transcriptional profile. These data suggest that T cells adapt by rewiring their transcriptomes in response to simulated weightlessness and that remodeling cues persist when reexposed to normal gravity.

## Teaser

Microgravity exposure reveals adaptation of immune cells with implication for therapeutic approaches for manned space missions.

## INTRODUCTION

Failure to activate and control the immune system results in recurrent infections, hematologic abnormalities, skin manifestations, gastrointestinal symptoms, and autoimmune disease. Space flights pose extreme challenges on the human body and impair the immune system, with changes persisting long after return to normal gravity (*1*–*6*). Among the Apollo crew members, 50% reported bacterial or viral infection upon landing back on Earth, suggesting immune suppression (*5*). T cells together with B cells constitute the adaptive immune system with an enormous breadth to react against pathogens and altered self. T cells have decreased activation in microgravity and reduced expression of many early activation genes (*7*–*9*). This reduced effectiveness is evidenced by increased infections and reactivation of latent viruses in astronauts (*10*). With the planned long-term manned missions to Moon and Mars, it will be important to better understand the effects of space flight on the immune system.

Advances in space research in mice and humans indicate a profound impact of the space environment on adaptive immunity including exposure to microgravity, increased radiation levels, psychological stress, and isolation with artificial environment (*11*–*15*). T cells develop in the thymus and enter into the circulation in a relatively unresponsive state as naïve T cells. Thymic weight and volume decrease during space flight in mice (*16*). While apoptosis of CD4^+^CD8^+^ T cells was ruled out as a cause, transcriptome analysis revealed an overall down-regulation of genes related to T cell activation and proliferation (*9*, *16*). A study in a xenogenic rat/mouse coculture exposed to space flight for 16 days showed a reduction in precursor T cells (*17*). A clinorotation experiment, to simulate microgravity, on fetal thymus organ culture for 12 days indicated that the loss of T cell precursors originated from reduced transition from the CD4^−^CD8^−^ to CD4^+^CD8^+^ T cell stages (*17*). In addition, T cell activation that is required for T cell proliferation and differentiation is consistently negatively affected in microgravity and simulated microgravity (*13*, *18*–*20*).

Naïve T cells require the stroma cell–derived cytokine interleukin-7 (IL-7) for their maintenance and survival (*21*). T cells recognize antigenic peptides presented by major histocompatibility class (MHC) I and II molecules, leading to up-regulation of costimulatory molecules such as CD28. At this T cell–primed stage, T cells turn on the expression of the autocrine growth factor IL-2 and its receptor IL-2R (IL-2RA and IL-2RB), leading to the induction of transcriptional programs driven by transcription factors such as IL-2–inducible activating protein 1 (AP-1), NFAT, and signal transducer and activator of transcription 5 (STAT5) (*22*). The AP-1 complex consists of multiple transcription factors including the JUN, FOS, mammary cell-activating factor (MAF), and activating transcription factor protein families, which are responsible for regulating T cell activation and differentiation. AP-1 in concert with NFAT is required for IL-2 gene transcription (*23*). A recent study defines how IL-2–induced signaling regulates chromatin remodeling that influence expression of transcription factors for T cell lineage commitment and also up-regulation of the IL-7R expression to maintain the primed T cell stage (*24*). IL-2R and IL-7R signal via Janus kinase 1/3 (JAK1/3) that induce phosphorylation of STATs (*25*). This results in dimerization and translocation into the nucleus where STATs, such as STAT5, regulate target gene transcription (*26*, *27*). JAK-STAT signaling is negatively regulated by the suppressor of cytokine signaling (SOCS) protein family, induced by STATs. SOCS1 negatively regulates IL-7 signaling, and deletion of SOCS1 results in a hyperresponsiveness to IL-7 (*28*).

T cell development and differentiation is tightly governed by expression of master regulator transcription factors that lead to expression or suppression of lineage-defining genes. The transcription factors B-cell lymphoma 11B (BCL11B), T cell factor 1 (TCF1), and GATA binding site 3 (GATA3) are critical for T cell development. ETS proto-oncogene 1 (ETS-1), AP-1, NFAT, and STAT regulate IL-2R and IL-7R gene expression in naïve T cells (*23*). Signaling by the T cell receptor (TCR) and cytokine receptors induces changes in transcriptional programs that allows naïve T cells to differentiate into more defined T helper (T_H_) subset such as T_H_1, T_H_2, T_H_17, T regulatory (T_reg_) and, T follicular helper cells (*22*, *25*). T-box transcription factor 21 (TBET) and GATA3 regulate differentiation to T_H_1 cells and T_H_2 cells, respectively, and GATA3 also serves an important role in CD8^+^ T cell differentiation (*29*–*32*). T cell differentiation is further regulated by expression of dual-specificity phosphatase (DUSP) family proteins. Deletion of DUSP4 leads to increased TCR-induced proliferation and expression of IL-2RA (*33*). Studies of the IL-2R have shown reduced cell surface expression in T cells exposed to microgravity during parabolic flight (*34*) but normal IL-2R expression in T cells exposed to microgravity (and hypergravity) in a sounding rocket (*35*). T cells from mice at the International Space Station (ISS) showed decreased IL-2R expression and reduced IL-2 secretion (*36*). These studies indicate that signal transduction and gene expression in T cells merit further investigation to understand how T cells are affected by microgravity.

The effects of space flight are best studied in the space environment. However, conducting research on board a spacecraft is fraught with several difficulties, including a shortage of astronauts’ time, increased requirements for biological and medical safety, lack of necessary laboratory equipment, and a lengthy procedure for approving a space experiment. To overcome these limitations, ground-based analogs are used to study individual factors of space flight, one of which is the dry immersion (DI) analog that simulates the effect of microgravity such as the redistribution of liquids and support unloading (*37*–*41*). Moreover, the ground-based analogs for simulated microgravity provide the possibility to investigate human physiology in the absence of confounding factors such as cosmic radiation, stress, and isolation. During DI, subjects are submerged neck down into a thermo-neutral (31° to 35°C) water bath protected by a waterproof filament (*37*, *38*, *41*, *42*). As compared to other microgravity analogs such as the head down tilt rest, DI bed volunteers more rapidly develop symptoms, which is a clear advantage for the volunteers (*37*, *38*, *40*–*43*). Results from DI are very similar to the real space missions and accurately and rapidly reproduces most of physiological effects of the space flight such as mechanical and axial unloading, physical inactivity, fluid shift, and hypodynamia (*37*, *38*, *40*–*43*). Shorter DI exposure (5 to 7 days) is enough to simulate changes on the neuromuscular and sensory-motor systems.

In this study, we investigated the transcriptional changes in T cells isolated from eight healthy volunteers over a 21-day period of DI exposure. The 21 days of DI were conducted and, compared to shorter DI exposure, considered long-term microgravity exposure. We found that T cells exposed to microgravity rapidly changed their transcriptional program by down-regulating protein-coding and noncoding genes controlling T cell activation and differentiation, whereas genes critical for naïve T cells were up-regulated. At day 21, T cells showed signs of adaptation with a transcriptional profile most similar to that of pre-DI T cells, although some transcriptional changes persisted in T cells 7 days after recovery from the DI. Moreover, we identified subsets of deregulated noncoding RNAs potentially affecting T cell proliferation via separate mechanisms. The study provides new insight into the long-term effects of microgravity by identifying alterations in important T cell gene expression programs.

## RESULTS

### Transcriptomes of CD3^+^ T cells separate temporally according to DI exposure

**Fig. 1.**
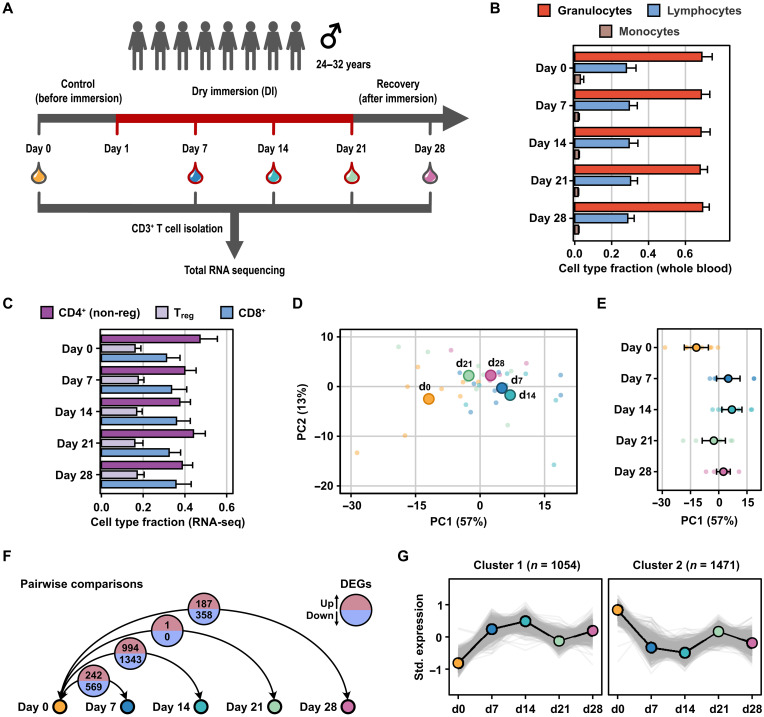
DI exposure induces transcriptome changes in eight volunteers. (**A**) Schematic representation describes the experimental procedure. Blood was taken from eight healthy volunteers at five time points: 7 days before DI exposure (day 0, orange); 7 (blue), 14 (teal), and 21 (green) days during DI exposure; and 7 days after DI exposure (day 28, pink). CD3^+^ T cells were isolated from the blood, and bulk RNA-seq was performed. (**B**) Horizontal bars show the fraction of granulocytes, lymphocytes, and monocytes in blood isolates before CD3^+^ T cell isolation as determined by flow cytometry. Values are indicated as means + 1.96 × SEM. (**C**) Horizontal bars display the estimated fraction of T cell populations detectable in the bulk RNA-seq data as determined by deconvolution analysis with the quanTIseq algorithm. Values are indicated as means + 1.96 × SEM. (**D**) Factorial map of the PC analysis separates bulk RNA-seq gene expression data of each individual volunteers (small dots) and mean values across all volunteers (large dots). The proportion of variance explained by each PC is indicated in parentheses. Color-coding is according to the different time points. (**E**) The dot plot shows PC1 separation of bulk RNA-seq samples by time point. Individual values (small dots) and mean values (large dots) are shown per time point (color-coded). Error bars indicate means ± 1.96 × SEM. (**F**) Illustration displays differentially expressed genes (DEGs) identified by pairwise time point comparisons using DESeq2. The number of significantly (adjusted *P* < 0.01) up- [positive log_2_ FC (fold change), red] and down-regulated (negative log_2_ FC, blue) genes is indicated. (**G**) Line charts show the two *k*-means clusters and number of differentially expressed genes per cluster detected in at least one time point comparison. Standardized gene expression profiles for individual genes (gray lines) and the cluster center (connected dots) are shown.

To study the effects of simulated gravity on the transcriptomes of T cells, volunteers were subjected to a DI protocol for a total of 21 days (Fig. 1A). Blood samples were collected at day 0 (7 days before DI exposure); at days 7, 14, and 21 during DI; and at day 7 after DI (day 28). CD3^+^ T cells were isolated and processed for RNA sequencing (RNA-seq) and transcriptome analysis following rigorous selection criteria to analyze T cell transcriptomes (Fig. 1A, figs. S1 to S4, and data S1 to S3). Samples from 2 of initially 10 volunteers were excluded on the basis of contaminating gene sets from non-T cells (figs. S1 to S4 and data S1). Flow cytometry analysis of blood lymphocytes, granulocytes, and monocytes indicated no major shift in cell populations upon DI exposure (Fig. 1B). Deconvolution of the bulk RNA-seq expression data from isolated CD3^+^ T cells indicated a high purity (>90%) of T cells across all time points (Fig. 1C, fig. S5, and data S2) (*44*, *45*). We detected similar proportions of CD4^+^ T cells (non-T_reg_ cells), T_reg_ cells, and CD8^+^ T cells across all time points (Fig. 1C).

The principal components (PC) analysis of RNA expression data revealed strong interpatient variability. Nevertheless, by comparing the means of the PC values, we found that the samples clustered by time spent in DI, as well as before and after immersion (Fig. 1D and fig. S2). Inspection of the first PC revealed that 14 days spent in DI showed the highest deviation in RNA expression from day 0, supported by the significant expression changes of 994 up- and 1343 down-regulated genes when compared to day 0 (Fig. 1, E and F, and data S3). At 7 days, 242 genes were up-regulated and 569 down-regulated when compared to day 0. After 21 days of DI, the T cell transcriptome showed less changes, with only one gene up-regulated at day 21 when compared to day 0. Increased differences in gene expression were observed again when comparing day 28 to day 0, which suggested a possible rewiring of gene expression changes 7 days after DI (Fig. 1F).

We next clustered the significantly deregulated genes by their expression profiles over time using *k*-means clustering with *n* = 2 centers on the transcripts that were significantly changed in at least one time point comparison. The two clusters represented gene sets showing up-regulation (*n* = 1054) and down-regulation (*n* = 1471) upon DI exposure (Fig. 1G and data S4). Gene ontology (GO) analysis revealed an up-regulation of TCR complex and external plasma membrane components together with generalized down-regulation of key transcriptional regulators and the epigenetic factors (fig. S6 and data S5). GO terms for cluster 1 of up-regulated genes included “T cell receptor complex,” “External side of plasma membrane,” and “Identical protein binding” (fig. S6 and data S5). Among cluster 2 of down-regulated genes, GO terms included “RNA polymerase II transcription,” “Transcription regulator complex,” “Nucleus,” and “Histone demethylase activity” (fig. S6 and data S5). Together, our data revealed that DI extensively perturbed T cell transcriptomes, which could be mediated via epigenetic reprogramming, and that T cells rewire their transcriptomes after DI exposure within the recovery period.

### Simulated microgravity in DI bed leads to down-regulation of differentiation markers and cytokine signaling suppressors

**Fig. 2.**
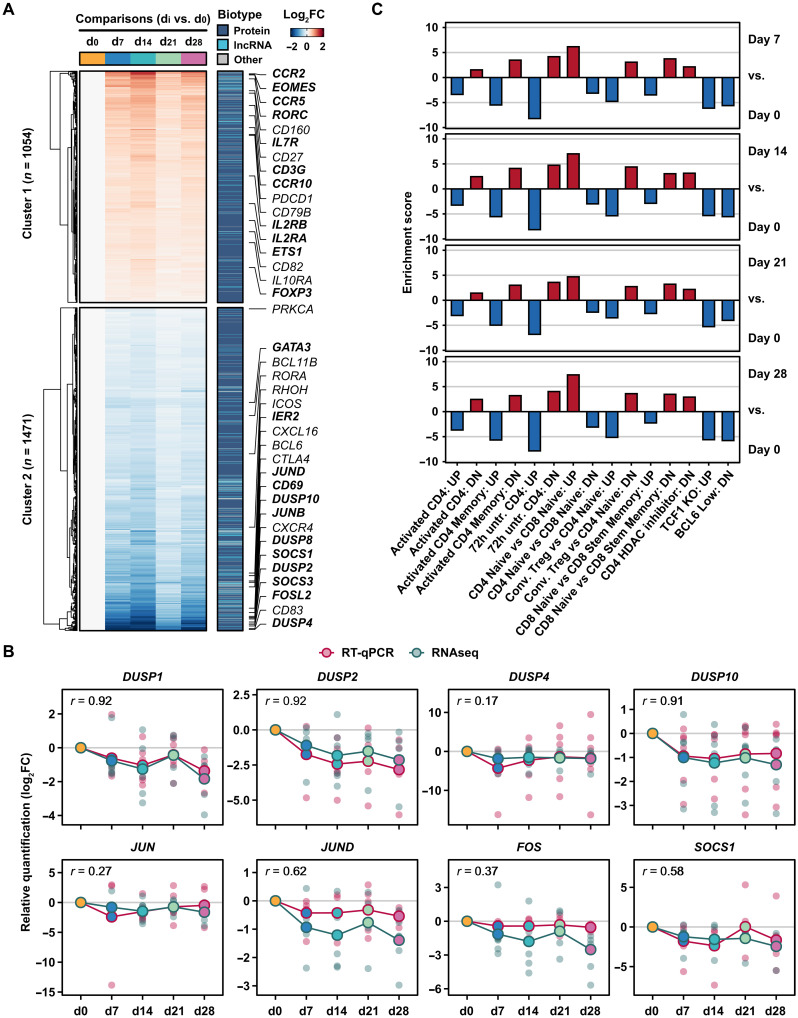
CD3^+^ T cell transcriptome perturbation upon DI affects proliferation, differentiation, and activation. (**A**) Heatmap shows the differentially expressed gene *k*-means clusters as depicted in Fig. 1G. Changes in gene expression are shown as log_2_ FC values relative to day 0. Gene annotations are indicated for protein-coding and long noncoding RNA (lncRNA) genes (color-coded). Relevant genes are annotated and highlighted (bold text). (**B**) Line graphs correlate RT-qPCR and RNA-seq log_2_ FC relative to day 0 for selected genes. Pearson correlation coefficients (*r*) between RT-qPCR and RNA-seq measurements are displayed for each gene (top-left corner). Values of individual volunteers (small dots, color-coded by time point) and mean values across all volunteers (connected dots) are shown for both RT-qPCR (red) and RNA-seq (green). (**C**) Vertical bars show enriched immune gene set groups obtained from gene set enrichment analysis using MSigDB. Enrichment score values are shown for up- (red) and down-regulated (blue) sets across time point comparisons (right labels). Association between individual gene sets and collapsed gene set groups can be found in data S5.

Activation and differentiation of T cells is regulated by changes in gene expression, involving cytokine signaling and activation of specific transcription factors (*22*, *25*, *46*, *47*). We investigated the genes that were up- or down-regulated within each gene cluster affected by DI over time (Fig. 2A and fig. S7). In space, T cells have reduced surface IL-2R expression and IL-2 secretion upon T cell activation (*9*, *36*). *IL-2* gene expression is transcriptionally regulated by the AP-1 complex (*48*). Upon exposure to DI, we found that transcript expression of genes important for T cell activation and differentiation such as the AP-1 complex members *JUNB*, *JUND*, and *FOS*, as well as DUSP family proteins, was consistently down-regulated and confirmed by reverse transcription quantitative polymerase chain reaction (RT-qPCR; Fig. 2, A and B, and data S3 to S6). Moreover, the early T cell activation markers immediate early response gene 2 (*IER2*) and *CD69* were down-regulated upon DI exposure (Fig. 2A). This is consistent with other studies where expression of early activation marker *IER2* and expression of the AP-1 complex members were down-regulated in space-flown T cells (*9*, *11*). T cell homeostasis and differentiation into T_H_ cells is activated by JAK-STAT signaling that is negatively regulated by the SOCS protein family. There are multiple SOCS proteins (SOCS1 to SOCS7) that each impinge on distinct T cell differentiation pathways. SOCS1 and SOSC3 inhibit T_H_1 and T_H_17 differentiation, as well as IL-2R and IL-7R signaling during activation of naïve T cells (*49*). Moreover, SOCS1 regulates CD8^+^ T cell homeostasis (*50*). *SOCS1* and *SOCS3* were down-regulated upon DI exposure (Fig. 2, A to B, and data S3 to S5). In sum, these data suggest that gene expression for T cell homeostasis and differentiation was deregulated upon DI exposure. Among the up-regulated genes in T cells during DI, we identified chemokine receptors *CCR2* and *CCR10* for homing into tissues; the TCR component *CD3G*; and the transcription factors *FOXP3*, *EOMES*, (*51*), and *RORC* (*52*), suggesting that DI exposure up-regulated genes that destine T cells for activation and tissue homing.

We next performed a threshold-independent gene set enrichment analysis using the Molecular Signatures Database (MSigDB) for human immune gene sets to investigate whether the changes in these key processes for T cell fate determination were recapitulated at the transcriptional level (Fig. 2C, fig. S8, and data S5) (*53*). Highly expressed genes in naïve CD4^+^ T cells were up-regulated in T cells upon DI (Fig. 2C). Genes indicative for T cell activation were significantly down-regulated upon DI exposure across all time points (Fig. 2C). Likewise, genes down-regulated upon T cell activation were up-regulated in T cells upon DI (Fig. 2C). Furthermore, gene sets linked to important drivers of T cell differentiation from thymic precursor cells and to generate effector T cells, such as *TCF-1* and *BCL6*, were also perturbed (Fig. 2C). Gene sets that are up-regulated in TCF1 knockout T cells “TCF1 KO: UP” and BCL6 low T cells “BCL6 Low: UP” were down-regulated in DI T cells, indicating that DI T cells were more similar to T cells expressing TCF1 and BCL6 (Fig. 2C). A highly expressed gene signature of CD8^+^ T memory cells, as compared to naïve CD8^+^ T cells, was down-regulated in DI T cells (Fig. 2C). Together, this data comparison suggests that DI T cells have a gene expression signature more similar to naïve T cells.

**Fig. 3.**
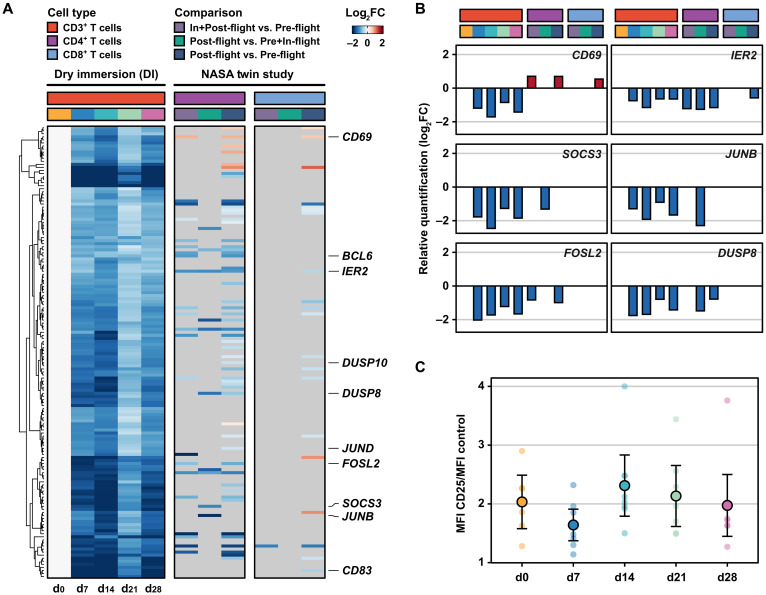
T cell transcriptome changes in DI resemble that of real space flight. (**A**) Heatmap demonstrates expression of overall down-regulated genes in CD3^+^ T cells identified upon DI (cluster 3 in fig. S9, *n* = 148) (left ) that overlap with genes in CD4^+^ (middle) and CD8^+^ (right) T cells obtained in the NASA twin study. Gene expression changes are shown as log_2_ FC to day 0 for DI and log_2_ FC for relevant comparisons in the NASA study (gray if missing). Relevant genes are highlighted. (**B**) Vertical bars display changes in expression for selected genes [*x* axis annotated according to (A)]. Log_2_ FC values show up- (red) and down-regulation (blue). (**C**) The dot plot denotes mean fluorescence intensity (MFI) ratio of cell surface marker CD25 compared to control as determined by flow cytometry. Values for individual volunteers (small dots) and mean values across all volunteers (large dots) are shown for each time point (color-coded). Error bars indicate means ± 1.96 × SEM.

To investigate to what extent these transcript expression changes could be recapitulated in CD3^+^ T cells from ISS crew members, we compared transcriptome changes identified upon DI exposure (this study) to those identified in the NASA twin study in both CD4^+^ and CD8^+^ T cells (*11*). The NASA twin study compared blood samples taken from two genetically identical twins; one twin astronaut was monitored before, during, and after a 1-year mission at ISS and the other twin served as a ground control (*11*). When compared to the highly down-regulated genes of DI T cells, the gene expression analysis of the NASA twin study identified reduced expression of key genes related to T cell activation such as *IER2* and proliferation and differentiation, including *SOCS3*, *JUNB*, and *FOSL2* (Fig. 3, A and B; fig. S9; and data S7). In addition, we confirmed that other regulators of T cell functions, such as *DUSP* genes, were down-regulated in both the DI and the NASA twin study (Fig. 3, A and B; fig. S9; and data S7). There were also genes that showed an opposite pattern, such as CD69, down-regulated in the DI T cells and up-regulated in the NASA T cells (Fig. 3, A and B; fig. S9; and data S7). Together, the transcript expression levels of key regulators for T cell activation, proliferation, and differentiation were down-regulated upon DI exposure, and these changes were comparable to that of T cells of ISS crew members.

### Gene expression of the IL-2 and IL-7 receptors is modulated during DI exposure

IL-2R expression and IL-2 cytokine secretion play a central role in T cell activation and differentiation. Microgravity induces down-regulation of cell surface IL-2R and reduced IL-2 secretion in stimulated T cells (*34*, *35*). While the gene set enrichment analysis indicated an overall down-regulation of T cell activation, transcript levels of both *IL-2RA* and *IL-2RB* were up-regulated at days 7 and 14 of DI exposure (Fig. 2A). However, flow cytometry analysis of CD25 (IL-2RA) cell surface expression on CD3^+^ T cells did not indicate significant fluctuations during DI exposure (Fig. 3C and fig. S10). Transcript levels of the IL-7R (*IL-7RA*), critical for activation of naïve T cells, were up-regulated during DI exposure (Fig. 2A). Similarly, transcript levels of *ETS-1*, a transcription factor that induces transcription of *IL-2R* and *IL-7R*, was also up-regulated, indicating that DI was altering IL-2 and IL-7 signaling pathways and “resetting” T cells into a naïve gene expression program.

### Simulated microgravity in DI bed leads to up-regulation of T cell activation and tissue homing

Among the up-regulated genes in T cells during DI, we identified chemokine receptors *CCR2* and *CCR10* for homing into tissues; the TCR component *CD3G*; and the transcription factors *FOXP3*, *EOMES* (*51*), and *RORC* (*52*), suggesting that DI exposure up-regulated genes that destine T cells for activation and tissue homing.

### A subset of long noncoding RNAs is affected during DI exposure

**Fig. 4.**
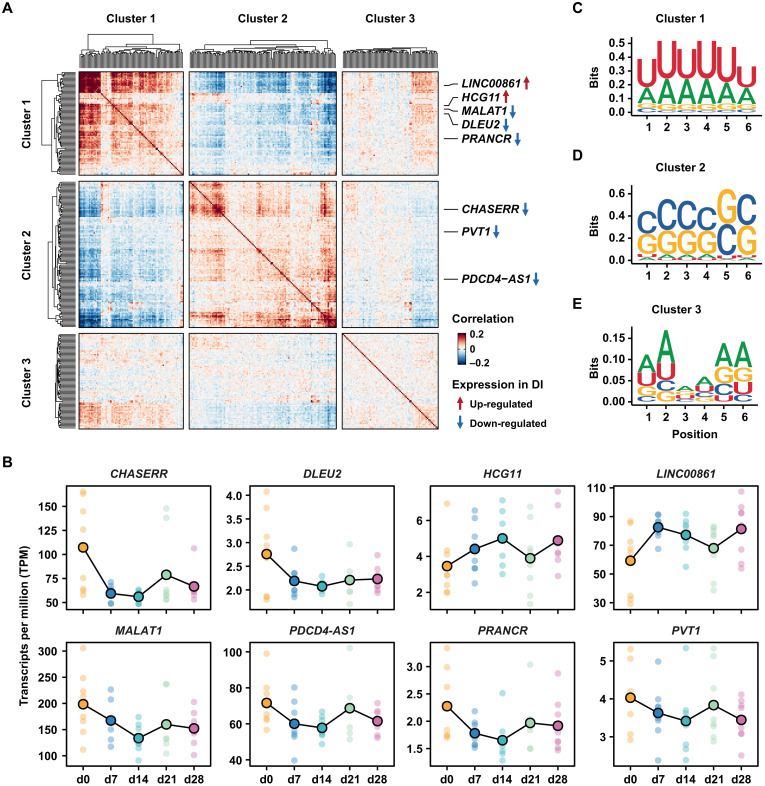
lncRNA groups with regulatory potential in T cells upon DI exposure. (**A**) Heatmap shows *k*-means clustering of differentially expressed lncRNAs based on their 6-mer enrichment profiles. Relevant lncRNAs for T cell proliferation and differentiation are highlighted. Gene expression changes are indicated as up- (red arrow) or down-regulation (blue arrow) in DI. (**B**) Line graphs display changes in gene expression for selected lncRNA genes measured as transcripts per million from the RNA-seq data. Values for individual volunteers (small dots) and mean values across all volunteers (connected dots) are shown for each time point (color-coded). (**C** to **E**) Motifs obtained from the top 100 enriched 6-mers identified in lncRNA cluster 1 (C), cluster 2 (D), and cluster 3 (E) are displayed.

Long noncoding RNAs (lncRNAs) are widely expressed and have emerged as a transcript class that regulates key cellular processes at both the transcriptional and posttranscriptional level (*54*). Functional roles of lncRNAs in T cells remain vastly uncharacterized, especially in the context of microgravity. We therefore explored functionally related groups of lncRNAs with altered expression upon DI that could modulate T cell activation, proliferation, and differentiation. To establish functional relationships between lncRNAs, we assessed the abundance of short motifs (*k*-mers) within the lncRNA sequence (*55*). We tested functional grouping of lncRNAs at several resolutions by using different values of *k* and found a shared pattern for sequence aggregation, which predominantly separated lncRNA transcripts into distinct clusters of high similarity (fig. S11 and data S3). Further inspection of the established clusters identified subsets of lncRNAs with the potential to mediate cellular processes in T cells, including the transcriptome changes observed upon DI (Fig. 4A). We found that *MALAT1*, which has been reported to regulate T cell differentiation by interacting with the transcriptional repressor EZH2 (*56*), was down-regulated in DI (Fig. 4, A and B). *PRANCR* and *DLEU2* were also down-regulated and are associated with cell proliferation and cancer progression (Fig. 4, A and B) (*57*). In contrast, *HCG11* and *LINC00861* were up-regulated upon DI (Fig. 4, A and B) and have been shown to inhibit cell growth and to positively associate with immune checkpoint genes, such as *PD-1* and *CTLA4*, respectively (*58*). These lncRNAs were all part of cluster 1 and shared enrichment for the same A/U-rich motif (Fig. 4C). Cluster 2 contained lncRNAs associated to altered gene expression and proliferation, including *CHASERR* and *PVT1* (Fig. 4, A to B) (*59*, *60*), as well as several antisense transcripts. For example, *PDCD4-AS1* was down-regulated in DI, which could promote increased gene expression of PDCD4, an inhibitor of translation initiation, and showed a similar C/G-rich motif enrichment (Fig. 4D). Last, cluster 3 showed no clear motif enrichment that could be indicative of diverse functional association of lncRNAs found within this cluster (Fig. 4E). Overall, we detected frequent deregulation of lncRNAs upon DI and established distinct functional groups with shared regulatory sequence features in T cells. We further delineate changes in lncRNA gene expression that could potentially alter T cell proliferation and differentiation in microgravity conditions.

## DISCUSSION

The immune system has been identified as a major determinant for the health of crew members during space flights. During long-term space missions (up to a year) and during short exposure to microgravity in space analogs (minutes to days), immune cell activation, gene transcription, and functionality are markedly affected (*3*). We here examined how T cells from eight volunteers were affected over 21 days of simulated microgravity in DI beds. We found that T cells at days 7 and 14 of DI changed their transcriptional profile and acquired a gene signature similar to naïve T cells. We detected signatures of adaption at day 21 where T cells showed a transcriptional profile comparable to that of pre-DI T cells. At 7 days after DI, T cells again changed their transcriptional profile, suggesting that changes in gene expression induced by DI were remodeled upon return to normal gravity.

T cell differentiation from naïve T cells to effector T cells is guided by activation of transcription factors in response to cell external stimuli such as cytokines. Many of these transcription factors, including *BCL11B*, *JUN/FOS* (AP-1), and *GATA3* were down-regulated in DI T cells at days 7 and 14. As an exception, the gene *FOXP3* controlling T_reg_ cell differentiation was up-regulated in DI T cells. The deconvolution of our bulk RNA-seq data revealed that the larger T cell subsets, CD3^+^CD4^+^ T cells, CD3^+^CD8^+^ T cells, and CD3^+^CD4^+^FOXP3^+^ T_reg_ cells, were not significantly changed in terms of cell number before, during, and after DI exposure. To uncover which T cell signature the DI T cell transcriptome resembled the most, we compared DI T cell gene expression with data from immune molecular gene signatures, which suggested that DI T cells had a transcriptional profile most similar to that of naïve T cells. Because the majority of blood T cells are naïve or at least nonactivated, this suggests that the DI T cells rewire the transcriptional program of naivety and may indicate an increased threshold for activation. Many effects of microgravity may influence changes of T cell transcriptional programs toward a naïve program including impaired T cell activation, at least within the first hour of activation (*9*, *34*). We did not detect any changes of proportion of monocytes, neutrophils, and lymphocytes in blood at the different time points, suggesting that the number of blood cells remain constant. Another and perhaps more intriguing possibility is that microgravity affects the mechanical properties of blood T cells. Mechanosensing is an important aspect of T cell activation, and the TCR has been defined as mechanoreceptor. Mechanical force is both required and sufficient to trigger T cell signaling (*61*). Future studies will be needed to address the role of TCR activation and mechanosensing in the presence and absence of gravitational force.

We examined the gene expression of *IL-2R* and *IL-7R*, which are key cytokine receptors activating naïve T cells and promoting differentiation (*23*, *62*). A recent study showed that TCR- and IL-2–dependent signaling poise the chromatin landscape of T cells to progress toward distinct alternative differentiation pathways. The IL-7R via AP-1 and STAT5 helps maintain the poised chromatin landscape for T cell differentiation until the right environmental cues are encountered (*24*). IL-2R consists of the IL-2RA, IL-2RB, and common γ chain. While *IL-2RA* gene expression is regulated during T cell differentiation, *IL-2RB* and the common γ chain are more stably expressed (*23*). We found that the both *IL-2RA* and *IL-2RB* together with *ETS-1*, which drives transcription of *IL-2RA*, *IL-2RB*, and *IL-7RA*, were up-regulated in T cells at DI days 7 and 14. Altered IL-2 production and decreased IL-2R cell surface abundance have been described in T cells obtained from space and space analog samples (*34*, *35*). Using simulated microgravity in mice by hindlimb unloading, T cells show increased cell surface expression of the IL-7R (*63*). IL-7R expression varies throughout T cell development, with high expression in naïve T cells, low expression in activated T cells, and then high expression in memory T cells (*62*). Together, our DI T cell gene expression data suggest that the IL-2R and IL-7R may be affected by microgravity and could be used as biomarkers for monitoring microgravity effects on crew member T cells during long-term flight missions.

Our extensive profiling of human circulating CD3^+^ T cell transcriptomes in DI allowed us to characterize the expression changes of lncRNA genes in a ground-based simulated microgravity setting. We have identified deregulated lncRNAs, which partake in compromising the immune system during DI and are linked to T cell proliferation and differentiation. Widespread functionality for lncRNAs has been reported recently all the way from structural roles in the assembly of nuclear condensates to regulating gene expression via different mechanisms, yet direct interaction with specific RNA binding proteins (RBPs) appears essential for many of their modes of action (*54*, *55*, *64*). Although lncRNAs are poorly conserved and lack a strong association between sequence homology and function, comparison of their *k*-mer abundance profiles has been shown to robustly detect shared functionality (*55*). By comparing the sequence composition profiles of lncRNAs altered in DI, we have established functional groups that can have similar roles in compromising T cell activity and may share RBP interaction partners. We identified two major groups with an A/U and G/C bias in their enrichment motifs, respectively. The presence of A/U-rich elements has been shown to modulate mRNA stability and translation of protein-coding genes in human CD8^+^ T cells (*65*, *66*). In the case of lncRNAs, G/C-biased sequence composition has been associated with cytoplasmic localization, splicing, and transcription efficiency (*66*, *67*). Together, this suggests that the identified A/U- and G/C-rich deregulated lncRNA could localize at different cellular compartments, spatially constraining their interactions with nuclear and cytoplasmic RBPs while primarily affecting transcriptional and posttranscriptional processes, respectively. Expression profiles of functional lncRNAs are often condition specific (*54*), and they may serve as powerful RNA-based biomarkers to monitor the state of T cells during space flight. Further experiments will be needed to identify those that have a key regulatory role in maintaining a functional immune response.

For long-term manned space missions, it is necessary to identify how to counteract effects of the space environment on human physiology (*68*). There has been a rapid expansion of biological therapy to treat diseases affecting the immune system on Earth. These include vaccines, growth factors, immune modulators, and monoclonal antibodies. Likely, such biologicals would be suitable countermeasures to prevent immune system dysfunction during deep space exploration. We have identified the IL-2R and IL-7R as possible targets for countermeasures to drive specific T cell responses (*23*, *69*), and these receptors are targets of several ongoing clinical trials (see ClinicalTrials.gov). IL-2 has been used clinically as a part of immunotherapy for malignancies, but its usefulness has been limited by toxicity and in vivo instability (*70*). Moreover, IL-2 therapy induces preferential expansion of suppressive T_reg_ cells rather than effector T cells because of the high expression of IL-2RA by T_reg_ cells (*70*). Recently, efforts have partially focused on manipulating the ability of IL-2 to target specific cell populations (*70*). Antibody targeting of the IL-2RA that prevents IL-2 from binding to IL-2RA has greater antitumor effects compared to IL-2 itself (*71*) and could be considered as a countermeasure for long space flights. For IL-7/IL-7R targeting, it is worth noting that altered levels of IL-7 and IL-7R are associated with immunopathology. Elevated IL-7R has been linked to multiple inflammatory diseases, leukemia (*72*, *73*), and resolution of chronic infection via SOCS3 (*74*). Moreover, JAK/STAT signaling proteins, downstream of cytokine receptors, pose further interesting targets, and biological compounds already exist to inhibit JAK3 that interacts with the IL-2R and the IL-7R to mediate STAT5 phosphorylation (*75*). Therefore, modulation of IL-2/IL-2R and IL-7/IL-7R signaling with biological therapies has great potential as countermeasures to restore immune dysfunction of crew members. IL-2/IL-7 countermeasures could be used in synergy with antisense oligonucleotides or small molecules targeting relevant regulatory sequences of lncRNAs that negatively regulate T cell functions. This combined approach could allow us to target specific T cells functionality and improve treatment effectiveness.

Because of the complexity of the DI analog, few studies have addressed how the immune system is affected and whether the changes are representative of the exposure to microgravity during space flights. We here detected major differences in the T cell transcriptome during DI followed by adaptation of gene expression changes. Our comparison between DI T cells (this study) and space T cells from the NASA twin study (*11*) suggested that many down-regulated genes were shared in the DI and space T cell data. These include genes encoding the T cell activating molecules IER2, the AP-1 complex member JUNB, SOCS3, and DUSP10, important for T cell differentiation. This indicates that DI exposure recapitulated some aspects of space-flown T cells, especially among down-regulated genes. Moreover, many of the down-regulated genes here were also detected in space-flown T cells upon short-term stimulation (*9*). There were also examples of genes differently regulated in the NASA twin study and the DI study. For example, the early T cell activation marker CD69 was up-regulated in the NASA twin study T cells and down-regulated in DI T cells. In future studies, it would be important to correlate *CD69* mRNA expression with flow cytometry analysis of CD69 cell surface expression to understand how CD69 is dysregulated in T cells exposed to microgravity. We cannot exclude interindividual differences between the NASA twin study and our DI study. Analysis of T cells from the eight volunteers in DI with limited influence of other environmental factors revealed intraindividual differences in transcriptome profiles that could be interesting to examine in future studies. This exploratory study focused on identifying the changes of the T cell transcriptome among the eight volunteers over time. The effects of DI on CD3^+^ T cell gene expression uncovered in this study could not be confirmed by functional assays. This calls for further studies using the power and flexibility of microgravity analogs to validate how altered gene expression leads to functional consequences for immune cells such as cell mechanosensing, proliferation, activation, and differentiation.

We have here focused on the analysis of T cell transcriptomics changes. We selected T cells because they are the most common lymphocyte subset in blood and there is a large body of literature of T cell analysis in space research. The main limitation of the study stems from the complex DI setup where many types of experiments were performed to address different aspects of space physiology because this is the first time such long DI study was performed. During these circumstances, our focus was to generate high-quality deep sequencing datasets to analyze T cell transcriptomics changes. Because of the limited amount of blood samples obtained, it was not possible to validate the data using orthogonal assays. In future studies, it will be important to validate the findings of this study by including analysis to correlate changes in gene transcription with protein expression and perform functional assays and to define transcriptome changes in different T cell subsets.

## MATERIAL AND METHODS

### DI bed study and sample collection

An experiment with 21 days of DI exposure without countermeasures was carried out at the Institute of Biomedical problems, Russian Academy of Sciences/Physiology, with the participation of healthy men aged between 24 and 32 years (*42*, *43*). Under a period of 1 year, the volunteers were split in pairs and were submerged for 21 days in DI beds. Blood was sampled from each volunteer at five time points, 7 days before submersion (day 0); at day 7, day 14, and day 21 in DI bed; and 7 days after submersion (day 28). The Code of Ethics of the World Medical Association (Declaration of Helsinki) for human samples was followed, and written consent was obtained from all individuals. The study was approved by the Biomedicine Ethics Committee of the RF SRC-Institute of Biomedical problems, Russian Academy of Sciences/Physiology Section of the Russian Bioethics Committee Russian Federation National Commission for UNESCO (minutes of meeting no. 483 from 03.08.2018).

### Exclusion criteria

Samples were excluded if the experimental procedure was interrupted by volunteers or technical errors in the equipment or the cell isolation. For unbiased exclusion, not linked to known technical errors, samples were excluded on the basis of gene cluster analysis of log_2_ fold changes if contaminating non-T cell gene signatures reduced statistical power (figs. S1 to S4 and data S1).

### CD3^+^ T cell isolation and flow cytometry analysis

Peripheral blood was anticoagulated by sodium heparin. Peripheral blood mononuclear cells (PBMCs) were isolated using Ficoll isolation (Ficoll-Paque PLUS, Cytiva). Human T cells were enriched by the positive CD3 cell selection Kit (EasySep Human CD3 Positive Selection Kit II, STEMCELL Technology) with a purity of 90 to 95%. For flow cytometry, PBMCs were stained with monoclonal antibodies against human CD3 (OKT3) and CD25 (CD25-4E3) (eBioscience, USA) in phosphate-buffered saline with 5% fetal bovine serum and washed twice before acquisition. Forward scatter and side scatter dot plots were used to determine proportion of lymphocytes, monocytes, and granulocytes (fig. S10). Data were obtained by a FACSCalibur cytometer (Becton, Dickinson and Company) and analyzed using the CellQuest Pro software.

### RNA extraction and sequencing

Total RNA was purified from enriched CD3^+^ T cells using the RNeasy Kit (QIAGEN). Each RNA sample was treated with DNA nuclease using an RNase-Free DNase kit (QIAGEN) during RNA isolation procedure to avoid contamination by genomic DNA. The concentration of purified total RNA was measured by NanoDrop 2000 (Thermo Scientific). RNA integrity and purity were visually evaluated using 1% UltraPure Agarose (Invitrogen) in 1× TAE for gel electrophoresis analysis. Human mRNA sequencing and data quality control were performed with Illumina HiSeq-PE150 Platform at Novogene (Hong Kong).

### Real-time qPCR gene expression profiling

For cDNA synthesis, 2 μg of total RNA per sample was used reaction using SuperScript II Reverse Transcriptase (Invitrogen). RT-qPCR reactions were performed using SsoAdvanced Universal SYBR Green Supermix kit with C1000/CFX96 RT-PCR System (Bio-Rad Laboratories). Gene-specific primers were designed with PrimerBank software (data S6). Gene expression profiling by real-time qPCR was performed (*76*) in three technical replicates for each cDNA template sample, with *C_t_* variation between triplicates less than cutoff ± 0.25. For gene expression data, the mean of the technical triplicate, Δ*C_t_*, RQ value of biological replicates, and SD between RQ of biological replicates were calculated. All RT-qPCR data were normalized to *HPRT1*. qPCR data for volunteers 1 and 2 were omitted from the analysis because of technical errors based on inflated *C_t_* values. Pairwise fold changes between each time points (day 7, day 14, day 21, and day 28) to reference (day 0) were calculated. Per gene Pearson correlation coefficients (*r*) between RNA-seq fold changes and qPCR fold changes were calculated.

### RNA-seq processing and analysis

RNA-seq reads were trimmed using Trimmomatic (v0.36), and reads mapping to ribosomal RNA were removed by aligning to a customized ribosomal RNA reference genome (HISAT2 v2.2.1). Nonaligned reads were further mapped to the hg38 reference genome retrieved from GENCODE v38 (HISAT2, GRCh38.p13). Generated SAM files were converted to BAM files and consequently processed (SAMtools v1.12). bedGraph files were generated using Homer (v4.11), and a count table was generated using SubRead (v2.0.0).

Deconvolution of the bulk RNA-seq data was performed using the immunedeconv R package (v2.1.0) with RNA-seq data in counts per million as input and method parameter set to quanTIseq (*44*, *45*). Paired *t* tests were used to assess the change of inferred T cell types over time. Differential gene expression analysis was performed using DESeq2 (v1.38.3) (*77*). Raw read counts for all time points (*n* = 5) and volunteers (*n* = 8) were used as input. Then, a generalized linear model that included time and volunteers as variables was fit to the data. Results for pairwise comparisons for each time point (day 7, day 14, day 21, and, day 28) to the reference (day 0) were retrieved according to the Wald test procedure. If *P* value adjustments resulted in NA, they were set to 1 (not significant). Genes were annotated using Ensembl release 104. *k*-means clustering with *n* = 2 centers was performed on the transcripts that were significantly changed in at least one time point comparison. Gene set overrepresentation analysis was performed on Gene Ontology (http://geneontology.org/; retrieved: September 2021) and Reactome (https://reactome.org/; retrieved: September 2021) sets with the hypeR R package (*78*). Gene set enrichment analysis was performed using the gage R package (v2.48.0) (*79*). Ranked −log_10_-adjusted *P* values multiplied by the sign of the log_2_ fold change (1 for up-regulated and −1 for down-regulated) from the differential gene expression analysis were used as input. Immune gene sets were retrieved from the MSigDB v7.4 immunologic collection (*53*). Gene sets were then filtered to include only T cell–related gene signatures and collapsed into relevant groups based on similarity. Only significant gene sets at adjusted *P* < 0.05 were considered in the analysis.

### lncRNA analysis

Canonical lncRNA transcript sequences were retrieved from GENCODE v38. Functional groups of lncRNAs with regulatory potential in T cells upon DI were defined using the SEEKR method (*55*). *k*-mer abundances for differentially expressed lncRNAs were normalized by length and *z*-scored, obtaining lncRNA-specific *k*-mer enrichment profiles. Similarity between lncRNAs was defined as the Pearson’s coefficients correlation of their respective *k*-mer enrichment profiles. Three main clusters of deregulated lncRNAs were established by applying the *k*-means clustering algorithm. Cluster-specific motif sequences were inferred from the top 100 enriched 6-mers and visualized using the ggseqlogo R package (*80*).
