# Supplementary material for: Exposure of volunteers to microgravity by dry immersion bed over 21 days results in gene expression changes and adaptation of T cells
Source: Sci Adv. 2023 Aug 25;9(34):eadg1610. doi: 10.1126/sciadv.adg1610 (PMC10456848; doi:10.1126/sciadv.adg1610)
Supplement: Supplementary file 1 — Figs. S1 to S11 Legends for data S1 to S8 [file sciadv.adg1610_sm.pdf]

Science Advances
AAAS

Supplementary Materials for

Exposure of volunteers to microgravity by dry immersion bed over 21 days results in gene expression changes and adaptation of T cells

Carlos J. Gallardo-Dodd *et al.*

Corresponding author: Lisa S. Westerberg, lisa.westerberg@ki.se

Sci. Adv. **9**, eadg1610 (2023)
DOI: 10.1126/sciadv.adg1610

The PDF file includes:

Figs. S1 to S11
Legends for data S1 to S8

Other Supplementary Material for this manuscript includes the following:

Data S1 to S8

Fig. S1.

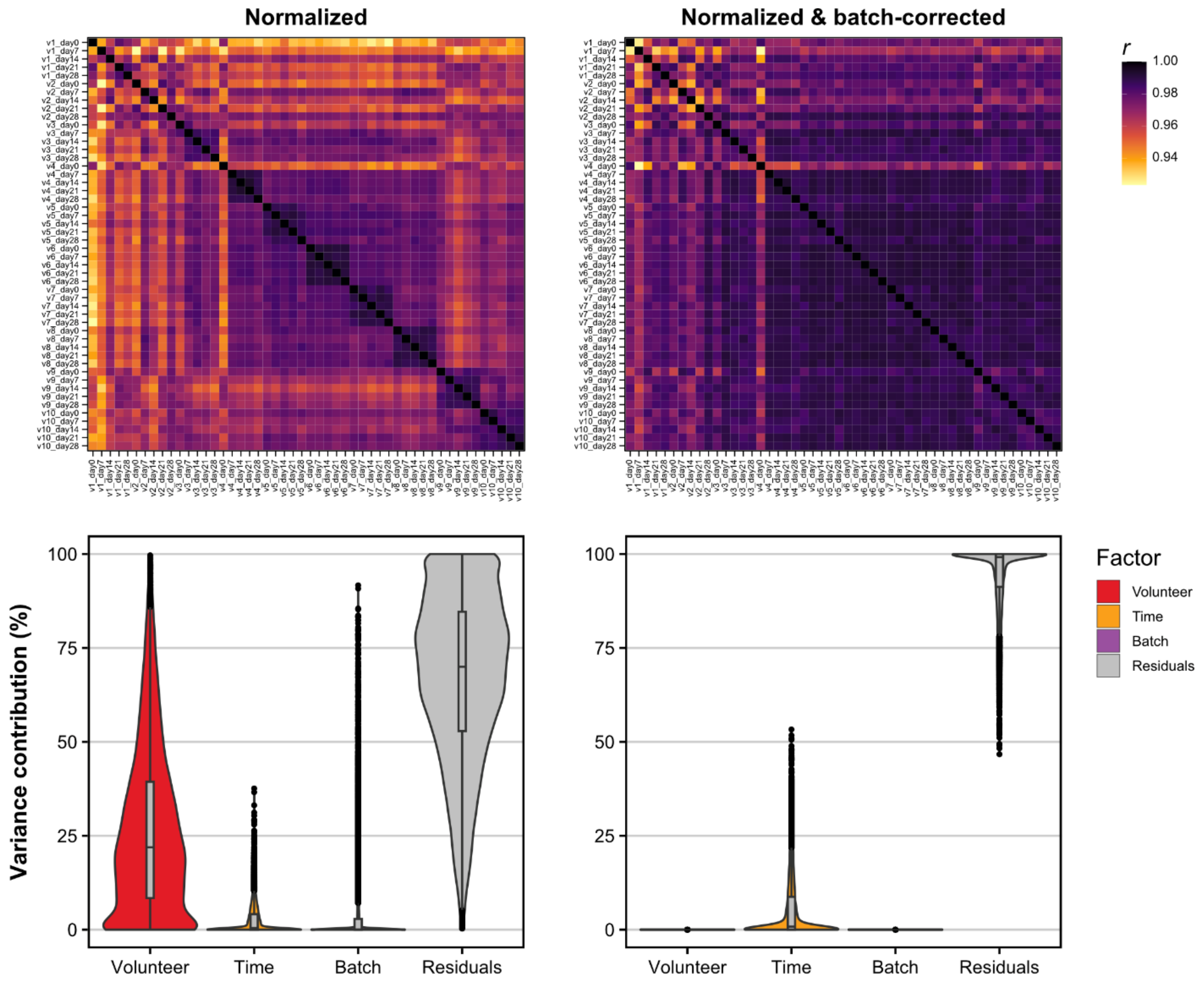

Fig. S1. Normalization and batch-correction of RNAseq data. Heatmaps show transcriptome-wide correlation matrices for individual samples with normalized (top left) as well as normalized and batch-corrected gene expression values (top right). Pearson correlation coefficients (r) high (purple) and low (yellow). Violin plots display proportional gene contribution to the total variance accounted by the different experimental factors for normalized (bottom left) as well as normalized and batch-corrected data (bottom right). Individual violins are color-coded by contributing variance of volunteers (red), time (yellow), batch (purple) and residual factors (grey). The median of the data is shown (black horizonal line) with the interquartile range (grey bar), and the minimum and maximum data value (thin black line).

Fig. S2.

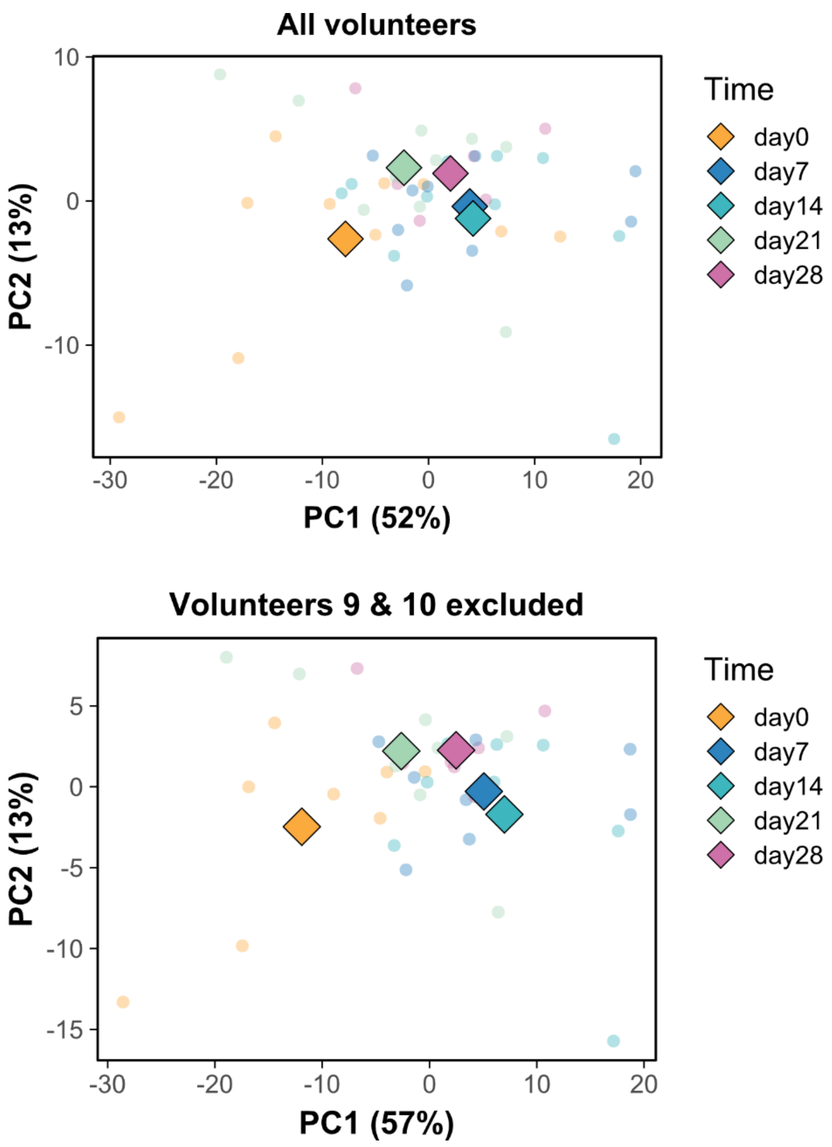

Fig. S2. Principal component analysis (PCA) of RNAseq data. Factorial map of the principial component (PC) analyses separate bulk RNAseq gene expression data of all volunteers (top) and after excluding volunteers 9 and 10 (bottom). The proportion of variance explained by each PC is indicated in parenthesis. Values for individual volunteers (small dots) and mean values across volunteers (diamonds) are shown. Color-coding indicates the different time points.

Fig. S3.

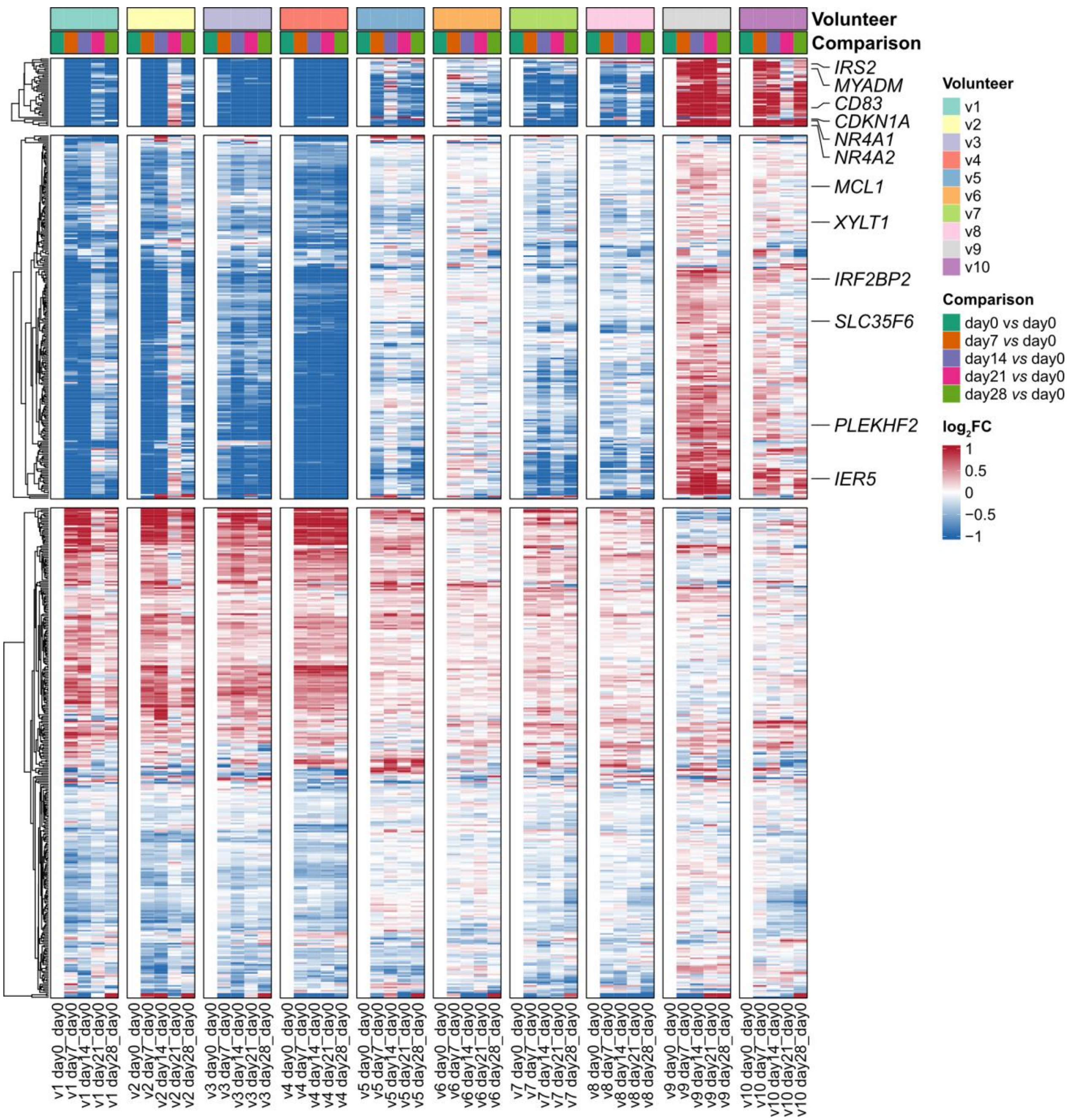

Fig. S3. Differentially expressed genes in all volunteers at FDR < 0.20. Heatmaps represent $\log_2$ fold change values for each volunteer relative to day 0. Genes that are highly upregulated in volunteers 9 and 10 and downregulated in volunteers 1-8 (top cluster) include genes that are highly expressed in blood monocytes such as *CDKN1A*, *NR4A1*, *NR4A2*, *MYADM*, *IRS2*, and *CD83*. Source: Immgen Gene skyline of blood human cell atlas.

Fig. S4.

Number of genes per analysis at FDR < 0.01		
	**All volunteers**	**volunteers 9 + 10 excluded**
**GLMtime**	13	1055
**day7Vsday0**	2	811
**day14Vsday0**	10	2337
**day21Vsday0**	0	1
**day28Vsday0**	9	545

Fig. S4. Differentially expressed genes detected at FDR < 0.01. The number of differentially expressed genes is shown for all testing conditions when including all volunteers (left column) and after excluding volunteers 9 and 10 (right column)**.**

Fig. S5.

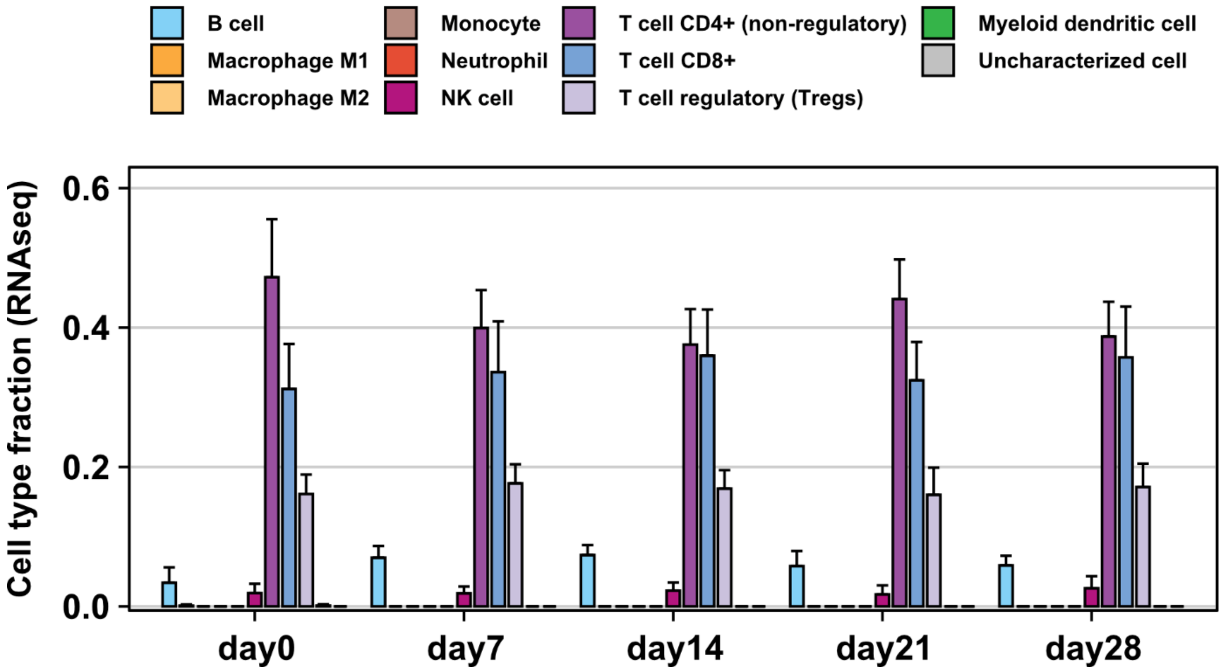

Fig. S5. Estimated fraction of cell populations represented in the RNAseq data. Vertical bars show the fractions of immune cell types determined from the gene expression data using deconvolution analysis with the quantiseq algorithm. Results for both T cell and residual non-T cell types (color-coded) are shown. Values are indicated as mean+1.96×SEM.

Fig. S6.

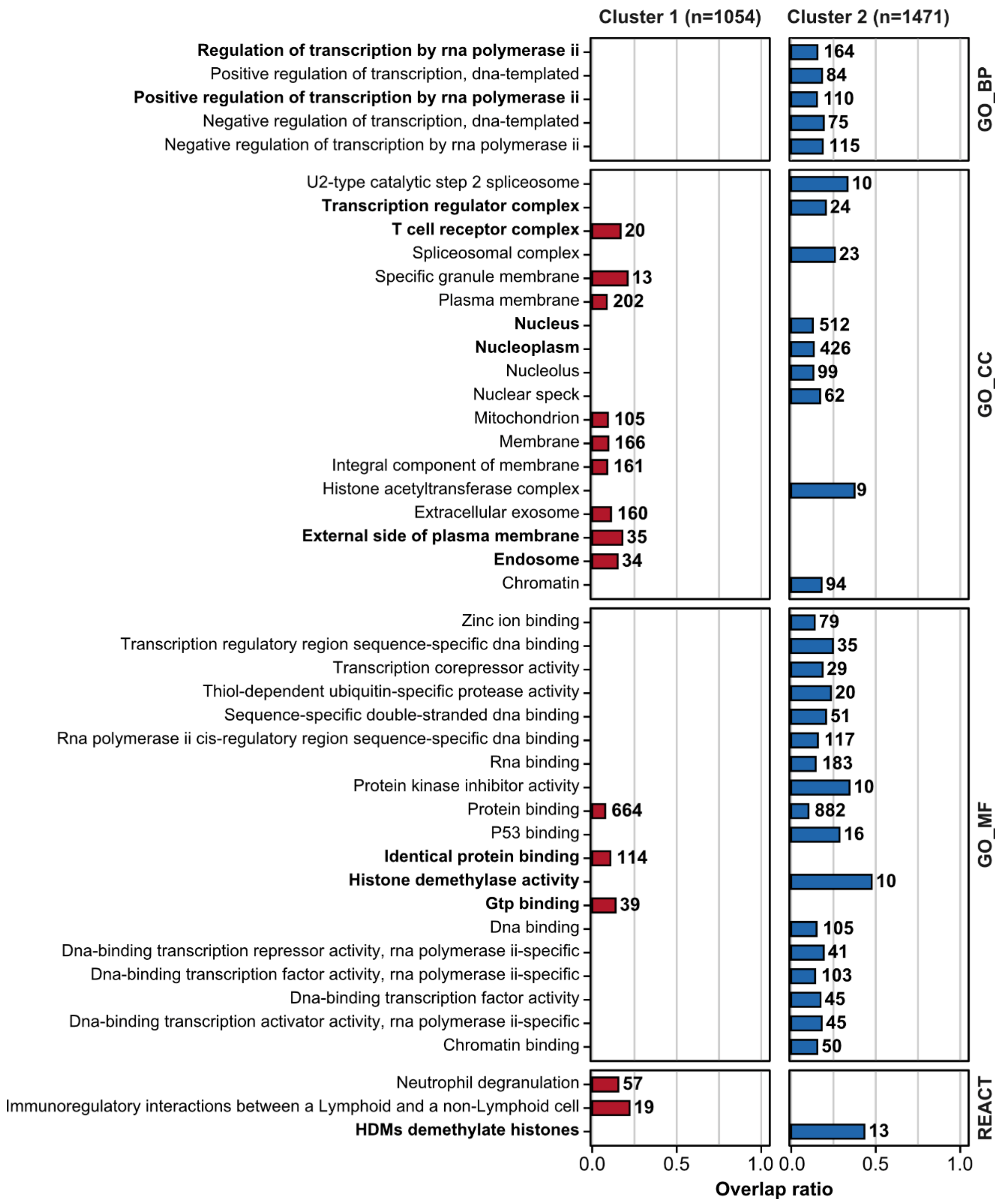

Fig. S6. Over-represented Gene Ontology (GO) and Reactome gene sets. Horizontal bars display the overlap ratios for up- and downregulated genes grouped in cluster 1 (red) cluster 2 (blue), respectively for the top GO terms and Reactome pathways. The gene number per top GO term and Reactome pathway is indicated. Only significant gene sets at FDR < 0.05 are reported. Relevant gene sets are highlighted (bold).

Fig. S7.

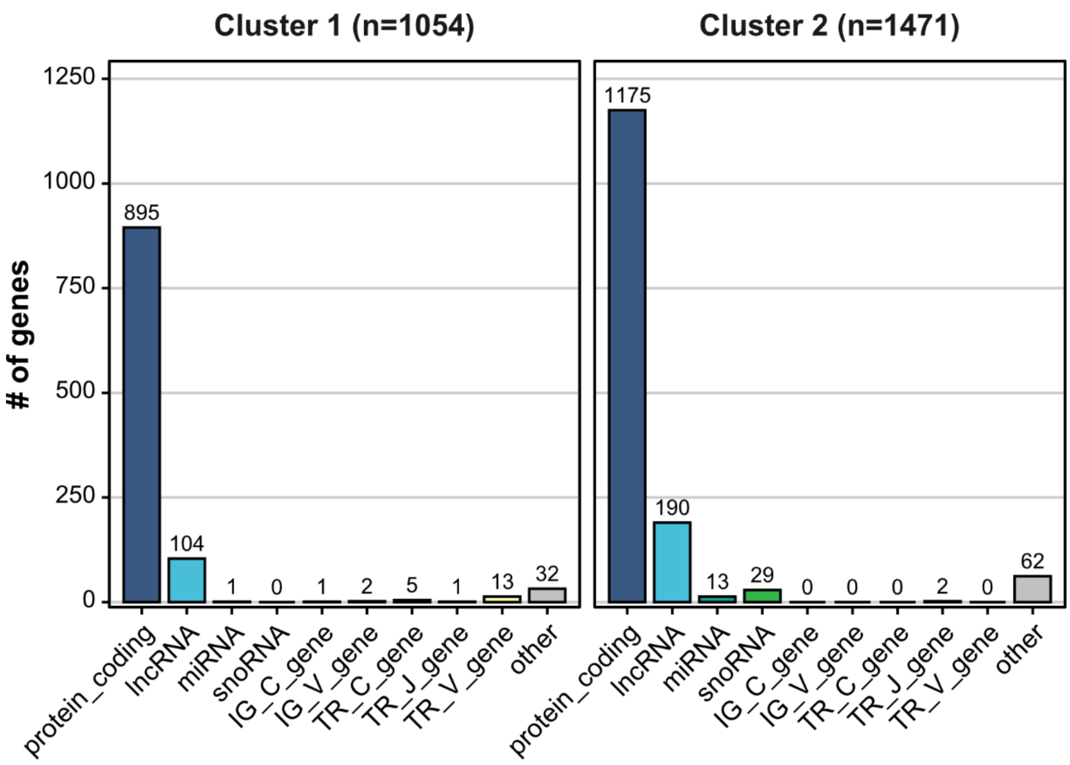

Fig. S7. Biotype distribution for differentially expressed gene clusters. Vertical bars show the number of genes per biotype category for upregulated cluster 1 (left) and downregulated cluster 2 (right).

Fig. S8.

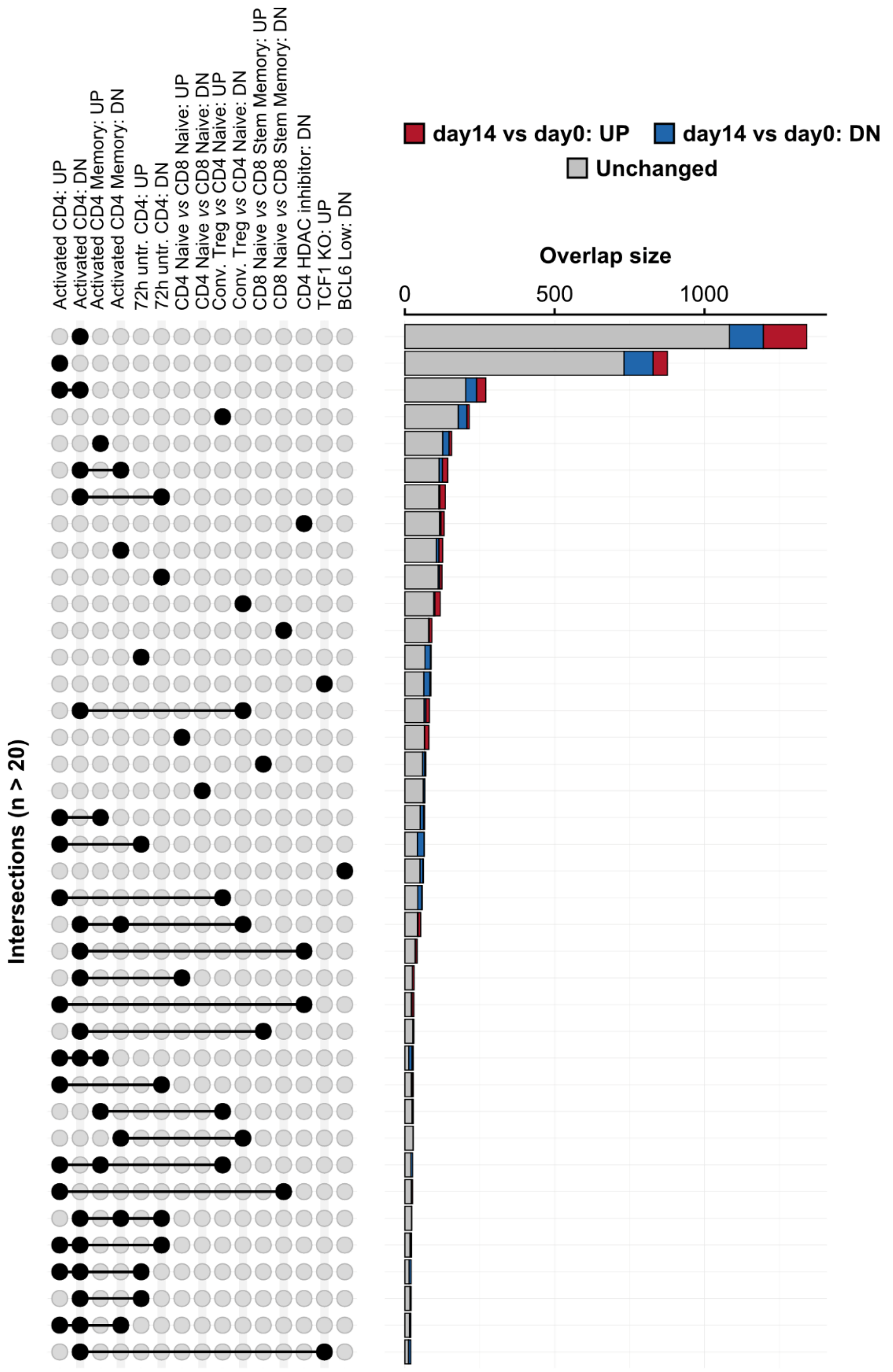

Fig. S8. Overlap of genes in groups of collapsed immune gene sets from MSigDB. Black circles (left) indicate the intersections between the different gene sets filtered for those intersections with at least 20 genes. Bar graph (right) shows the number of overlapping or unique genes for each individual intersection across gene sets (left). Genes detected as up- (red) or downregulated (blue) when comparing day 14 to day 0 are indicated.

Fig. S9.

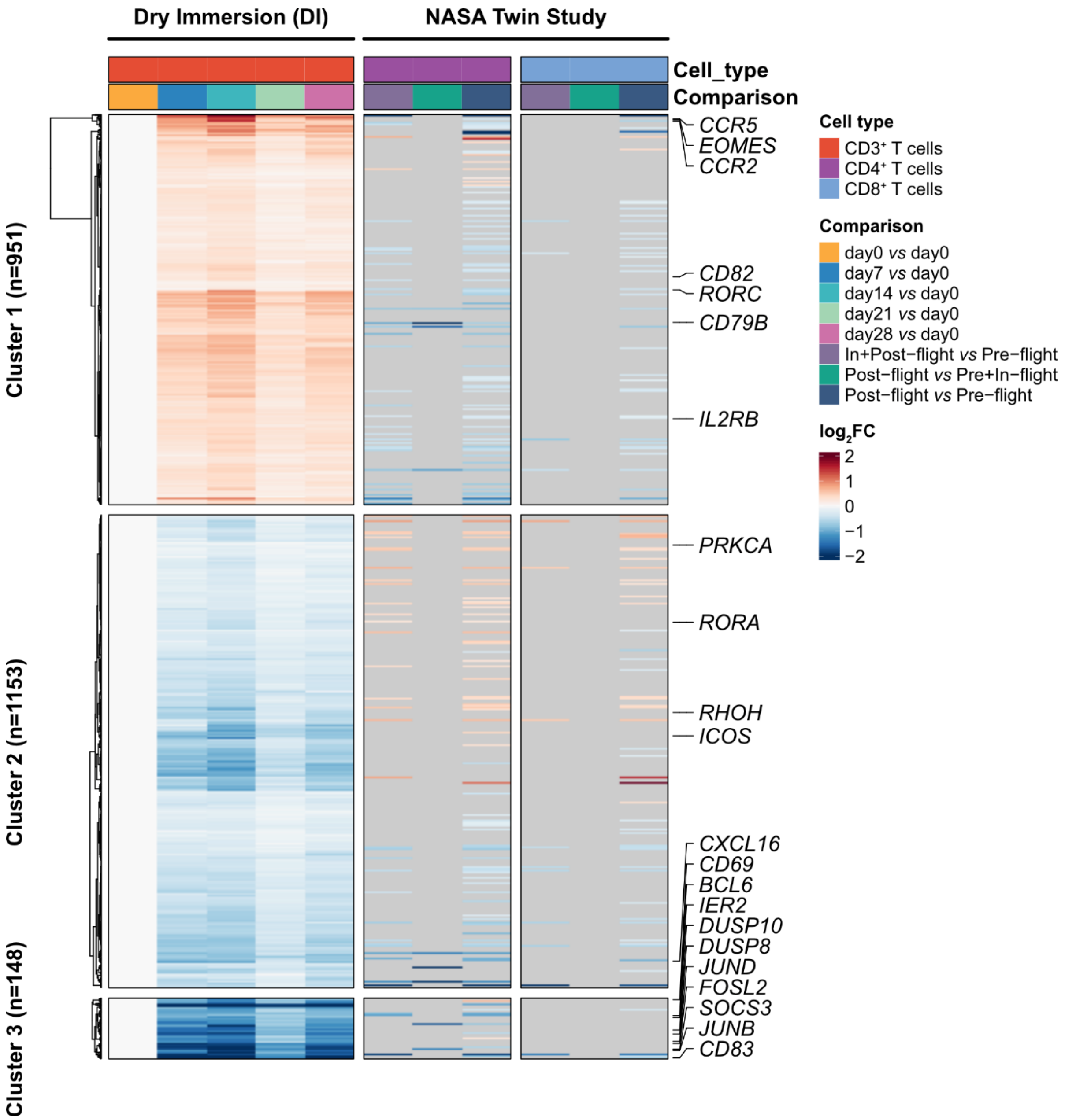

Fig. S9. Heatmap including differentially expressed genes in dry immersion that overlap with genes identified in the NASA twin study. Heatmap demonstrates the *k*-means clusters (n = 3) by gene expression profiles in $CD3^+$ T cells identified upon dry immersion (left panel) that overlap with genes in $CD4^+$ (middle panel) and $CD8^+$ (right panel) T cells obtained in the NASA twin study. The gene number per cluster is shown. Gene expression changes are shown as $\log_2$ FC to day 0 for dry immersion and $\log_2$ FC for relevant comparisons in the NASA study (gray if missing). Relevant genes are highlighted.

Fig. S10.

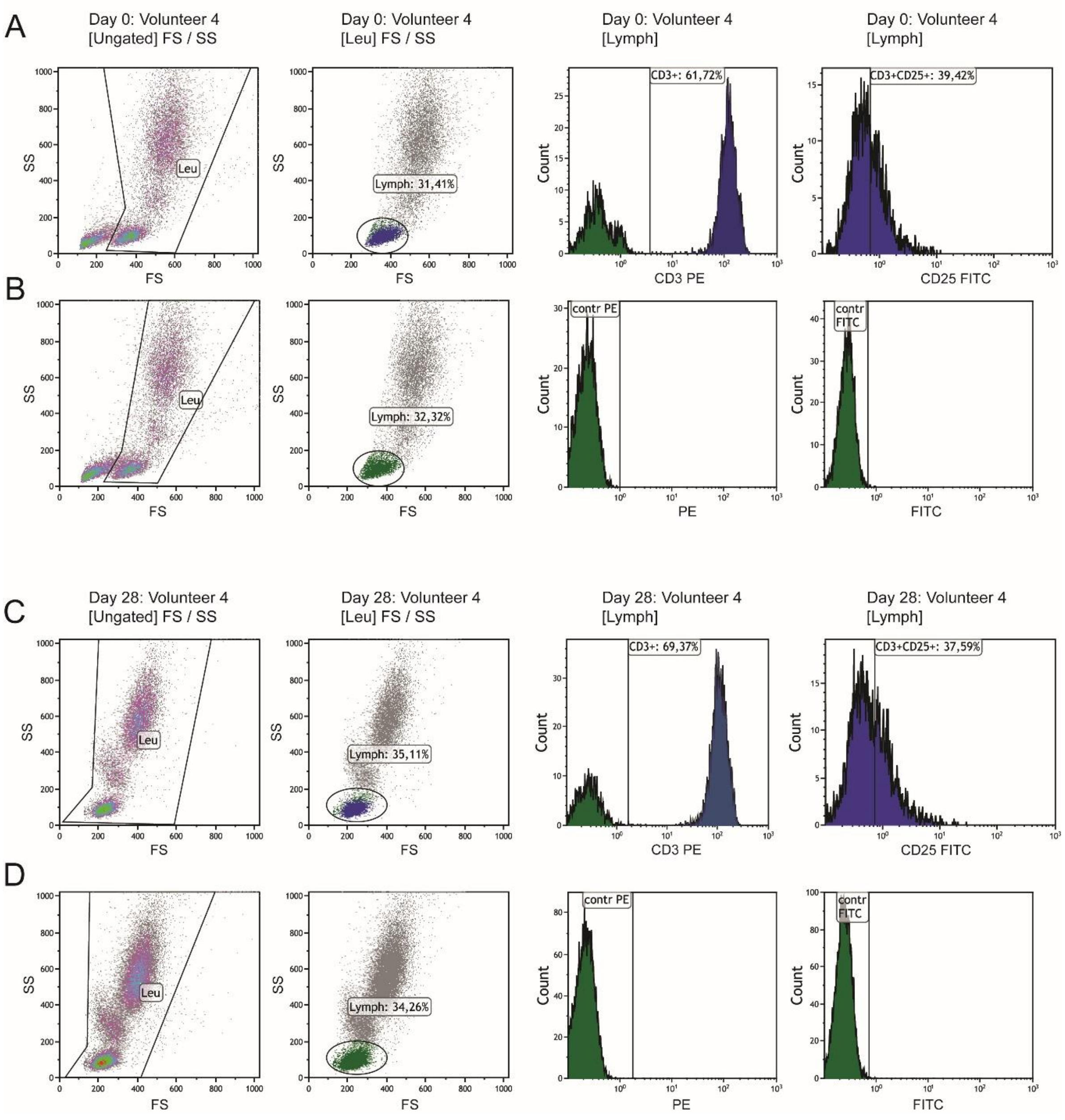

Fig. S10. Representative gating strategy for flow cytoemtry analysis applied to all volunteers. Flow cytometry analysis showing the lymphocyte gate and CD3 and CD25 (IL-RA) expression among the lymphocytes. (A and C) $CD3^+$ cells and $CD3^+CD25^+$ cells among lymphocytes. (B and D) Negative controls for CD3 and CD25 expression.

Fig. S11.

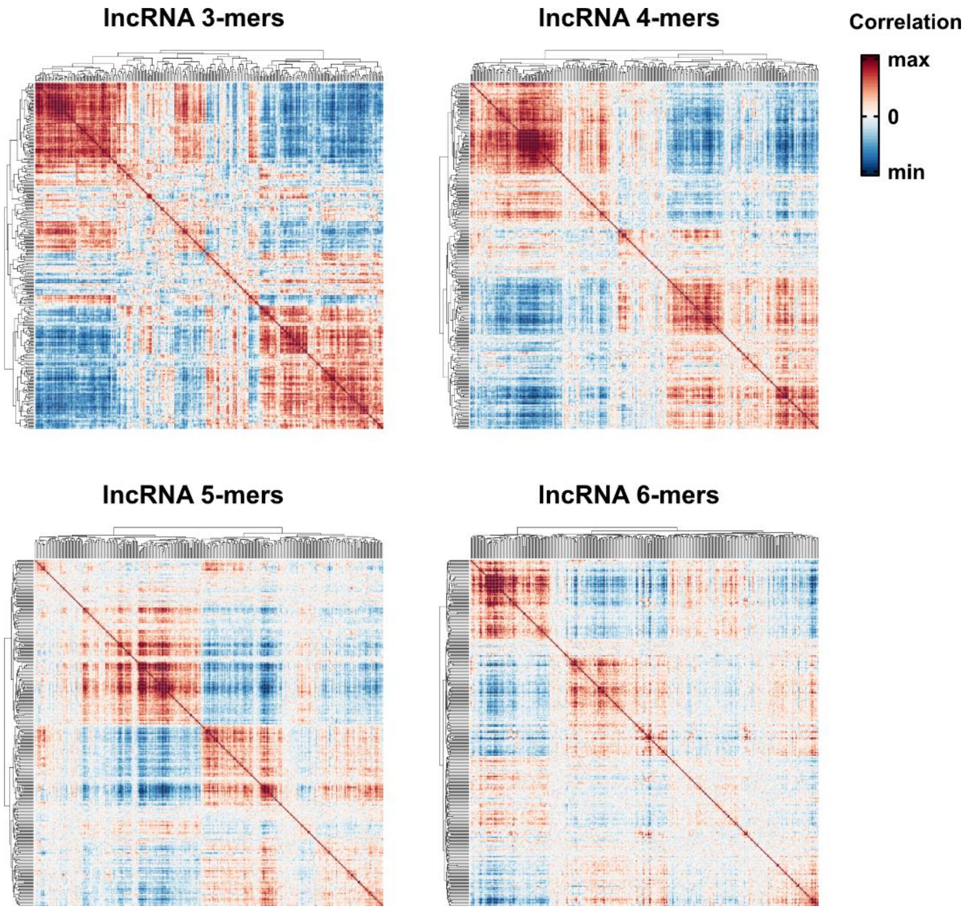

Fig. S11. Granularity of correlations between differentially expressed lncRNA at several *k*-mer resolutions. Pearson correlation coefficients (*r*) matrices are shown for different values of *k*: 3-mers (top left), 4-mers (top right), 5-mers (bottom left) and 6-mers (bottom right). The diagonal contains correlations of *k*-mer enrichment profiles for each lncRNA compared to itself.

Data S1. (separate file)

Differentially expressed genes detected for all volunteers (incl. 9 and 10) at FDR < 0.2 and $\log_2$ FC values relative to day 0 per volunteer.

Data S2. (separate file)

Estimated fractions from cell type deconvolution analysis of RNAseq data and paired *t*-test results for T cell types.

Data S3. (separate file)

Differential gene expression analysis results for all time point comparisons.

Data S4. (separate file)

Differentially expressed genes detected in at least one time point comparison with *k*-means cluster assignments and mean $\log_2$ FC values relative to day 0.

Data S5. (separate file)

Gene set analysis results for GO categories, Reactome pathways and MSigDB immune sets.

Data S6. (separate file)

List of primers used for RT-qPCR experiments.

Data S7. (separate file)

Differentially expressed genes detected in dry immersion and the NASA twin study with $\log_2$ FC values and *k*-means cluster assignments.

Data S8. (separate file)

Code for the computational analysis.
