# Supplementary material for: Exposure of volunteers to microgravity by dry immersion bed over 21 days results in gene expression changes and adaptation of T cells
Source: Sci Adv. 2023 Aug 25;9(34):eadg1610. doi: 10.1126/sciadv.adg1610 (PMC10456848; doi:10.1126/sciadv.adg1610)
Supplement: Supplementary file 2 — Data S1 to S8 [file sciadv.adg1610_data_s1_to_s8.zip › adg1610_Data_S8.pdf]

# Step 0: Pre-processing with all volunteers

Carlos Gallardo & Christian Oertlin

17 April, 2023

```
# Import libraries and helper functions
source("code/helper_functions.R")
library(tidyverse)
library(magrittr)
library(patchwork)
library(DESeq2)
library(limma)
library(variancePartition)
library(RColorBrewer)
library(ComplexHeatmap)

# Colors
colPals <- vector(mode = "list")
colPals$time <- setNames(c("#FBAA3E", "#2C83BE", "#3EB6BD", "#A3D5B3", "#CD71A8"),
                         nm = c("day0", "day7", "day14", "day21", "day28"))
colPals$time_light <- setNames(c("#FDD6A1", "#A2CDE9", "#AFE2E5", "#DDF0E3", "#E8BDD6"),
                               nm = c("day0", "day7", "day14", "day21", "day28"))
colPals$time_dark <- setNames(c("#D87E04", "#174564", "#1F5C60", "#49A065", "#AA3C7E"),
                              nm = c("day0", "day7", "day14", "day21", "day28"))
colPals$inferno <- c("#000004", "#420A68", "#932667", "#DD513A", "#FCA50A", "#FCFFA4")
colPals$factors <- setNames(c('#E31D27','#FA9F1C','#9A509F','#C1C1C1'),
                            nm = c("volunteer","time","batch","Residuals"))
```

## Important note:

> This script contains the exploratory analysis conducted on all 10 volunteers participating in the dry immersion (DI) study. As shown below, v9 and v10 specifically show elevated expression of monocyte genes (e.g., CDKN1A, NR4A1, NR4A2, MYADM, IRS2 and CD83) suggesting contamination for these samples. As per our exclusion criteria we chose to remove these for further analyses. The data provided includes volunteers 1 to 8 which are used for investigation in the study. Data with v9 and v10 can be provided upon request to reproduce the pre-processing steps in this script.

## Load data

### Background annotation

```
ann_data <- read.table(
  file = 'data/resources/gene_annotation_ensembl_v104.txt',
  stringsAsFactors = FALSE,
  sep = "\t",
  header = TRUE,
  fill = FALSE,
  quote = "") %>%
  dplyr::rename(Geneid = ensembl_gene_id)
```

### RNAseq expression data

```
RNAseq <- vector("list")

RNAseq[["unfilt"]][["rawdata"]] <- read.table(file = 'data/rnaseq/exon_counts_all.txt',
                                              stringsAsFactors = FALSE,
```

```
                                              sep = "\t",
                                              header = TRUE)

# Make duplicated gene names unique
RNAseq[["unfilt"]][["rawdata"]] <- RNAseq[["unfilt"]][["rawdata"]] %>%
  mutate(GeneSymbol = uniquify(plyr::mapvalues(.$Geneid,
                                               from = ann_data$Geneid,
                                               to = ann_data$external_gene_name,
                                               warn_missing = F), sep = '_'))

RNAseq[["unfilt"]][["annotation"]] <- RNAseq[["unfilt"]][["rawdata"]] %>%
  select(Geneid, GeneSymbol) %>%
  inner_join(ann_data, by = "Geneid") %>%
  select(-external_gene_name)

RNAseq[["unfilt"]][["design"]] <- read.table(file = 'data/RNAseq/design_mtx_all.txt',
                                             stringsAsFactors = FALSE,
                                             sep = "\t",
                                             header = TRUE) %>%
  mutate(sample = factor(sample, levels = sample),
         batch = factor(batch, levels = unique(batch)),
         volunteer = factor(volunteer, levels = unique(volunteer)),
         time = factor(time, levels = unique(time)))

RNAseq[["unfilt"]][["counts"]] <- RNAseq[["unfilt"]][["rawdata"]] %>%
  select(-c(1:3)) %>%
  column_to_rownames("GeneSymbol")
```

# Pre-processing

## Filtering zero count genes

```
paste("Raw feature count:", nrow(RNAseq$unfilt$counts))
```

```
## [1] "Raw feature count: 60649"
```

```
tokeep <- rowSums(RNAseq$unfilt$counts) > 0
paste("Non-zero feature count:", sum(tokeep))
```

```
## [1] "Non-zero feature count: 42934"
```

```
RNAseq$unfilt$rawdata <- RNAseq$unfilt$rawdata[tokeep,]
RNAseq$unfilt$annotation <- RNAseq$unfilt$annotation[tokeep,]
RNAseq$unfilt$counts <- RNAseq$unfilt$counts[tokeep,]
rm(tokeep)
```

## Library sizes and gene expression distributions

```
df <- RNAseq$unfilt$design
df$lib.size <- colSums(RNAseq$unfilt$counts)

ggplot(df, aes(x=sample, y=lib.size/1e6, fill=volunteer)) +
  geom_bar(stat = "identity", width = 0.8) +
  xlab("") +
  ylab("Library size (million reads)") +
  scale_x_discrete(expand =expansion(mult = c(.02, .02))) +
  scale_y_continuous(expand =expansion(mult = c(.02, .05))) +
  theme_custom(
    axis.text.x.bottom = element_text(angle = 90, hjust = 1, vjust = 0.3)
  )
```

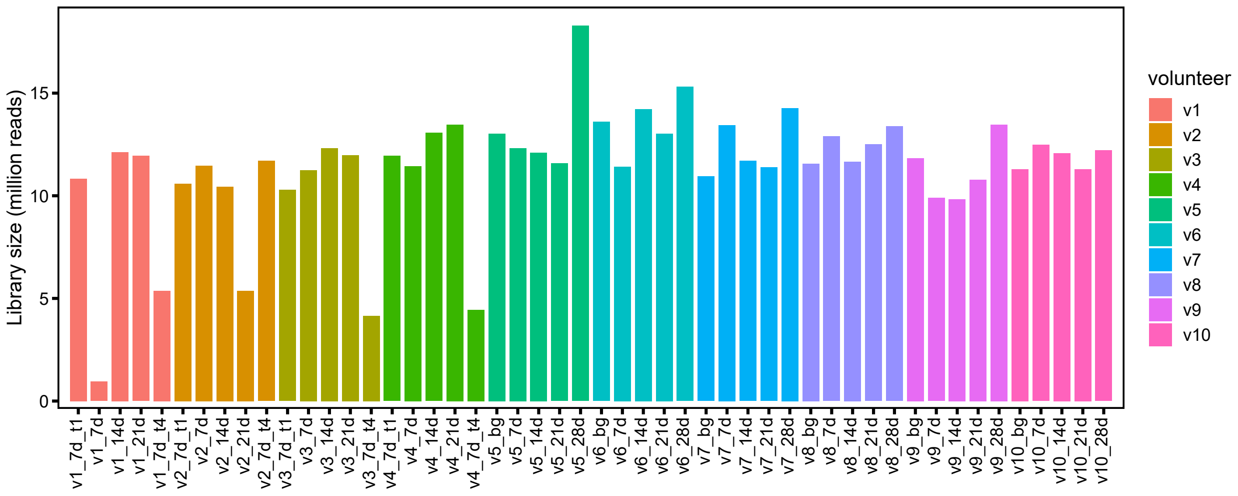


```r
df <- RNAseq$unfilt$counts %>%
  rownames_to_column(var = "geneID") %>%
  pivot_longer(cols = c(2:length(.)),
               names_to = "sample") %>%
  dplyr::rename(counts = value)

df$volunteer <- rep(RNAseq$unfilt$design$volunteer, dim(RNAseq$unfilt$counts)[1])
df$time <- rep(RNAseq$unfilt$design$time, dim(RNAseq$unfilt$counts)[1])
df$sample <- factor(df$sample, levels = names(RNAseq$unfilt$counts))

ggplot(df, aes(x=sample, y=log2(counts+1), fill=volunteer)) +
  geom_violin(scale = "area") +
  xlab("") +
  ylab(expression(Log[2](counts+1))) +
  scale_x_discrete(expand =expansion(mult = c(.02, .02))) +
  scale_y_continuous(expand =expansion(mult = c(.02, .05))) +
  theme_custom(
    axis.text.x.bottom = element_text(angle = 90, hjust = 1, vjust = 0.3),
    legend.position = "top"
  )
```

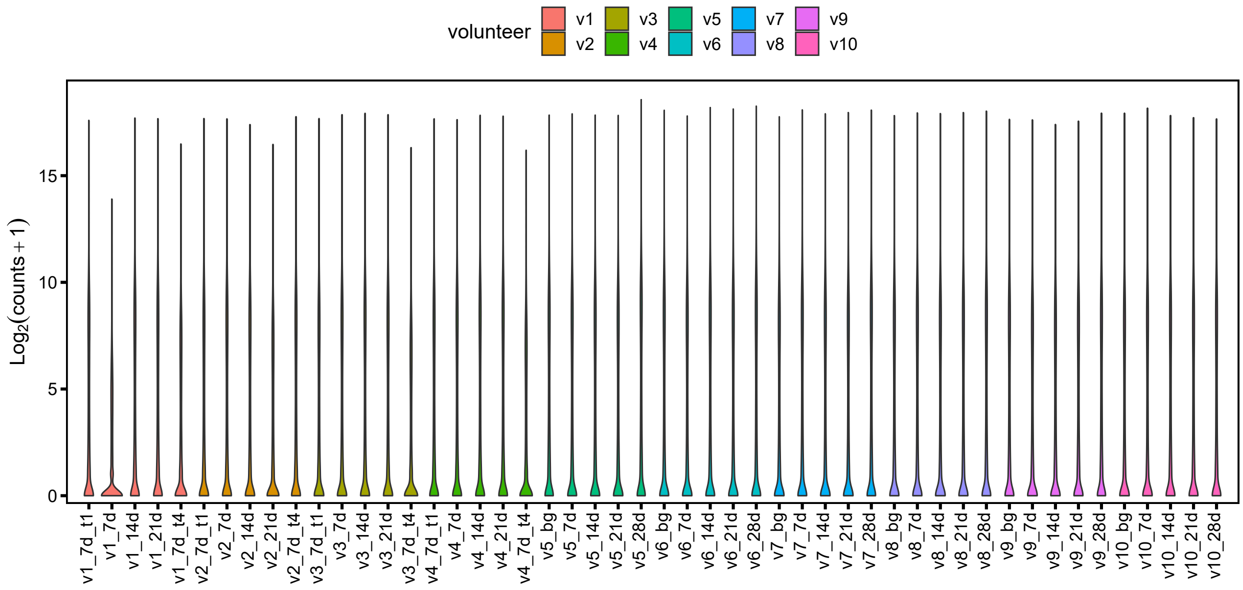

## Filtering low abundance genes

```
# Calculate CPM
RNAseq[["unfilt"]][["cpm"]] <- cpm.normalize(RNAseq$unfilt$counts)

abovethresh <- RNAseq$unfilt$cpm > 1

df <- data.frame(samples = factor(seq(0,ncol(RNAseq$unfilt$cpm),1),
                                  levels = rev(seq(0,ncol(RNAseq$unfilt$cpm),1))),
                 genes = c(table(rowSums(abovethresh)))) %>%
  mutate(cumulative = rev(cumsum(rev(genes)))) %>%
  mutate(remaining = sum(genes)-cumulative) %>%
  pivot_longer(cols = c("cumulative","remaining"),
               names_to = "group") %>%
  mutate(group = factor(group, levels = c("remaining", "cumulative")))

ggplot(data=df, aes(x=samples, y=value, fill=group)) +
  geom_bar(color="black", size=0.5, width=0.8, position="stack", stat="identity") +
  geom_hline(yintercept = unlist(df[df$samples == "20" & df$group == "cumulative", "value"]),
             linetype="solid", size=1, color="#EF2126") +
  geom_vline(xintercept = "20", linetype="solid", size=1, color="#EF2126") +
  xlab("# of samples present (CPM>1)") +
  ylab("# of genes") +
  scale_x_discrete(expand =expansion(mult = c(.02, .02))) +
  scale_y_continuous(expand =expansion(mult = c(.02, .00)),
                     limits = c(0,45000), breaks = seq(0,45000,15000)) +
  scale_fill_manual(values = c("grey80", "grey20")) +
  theme_custom(base_size = 8)
```

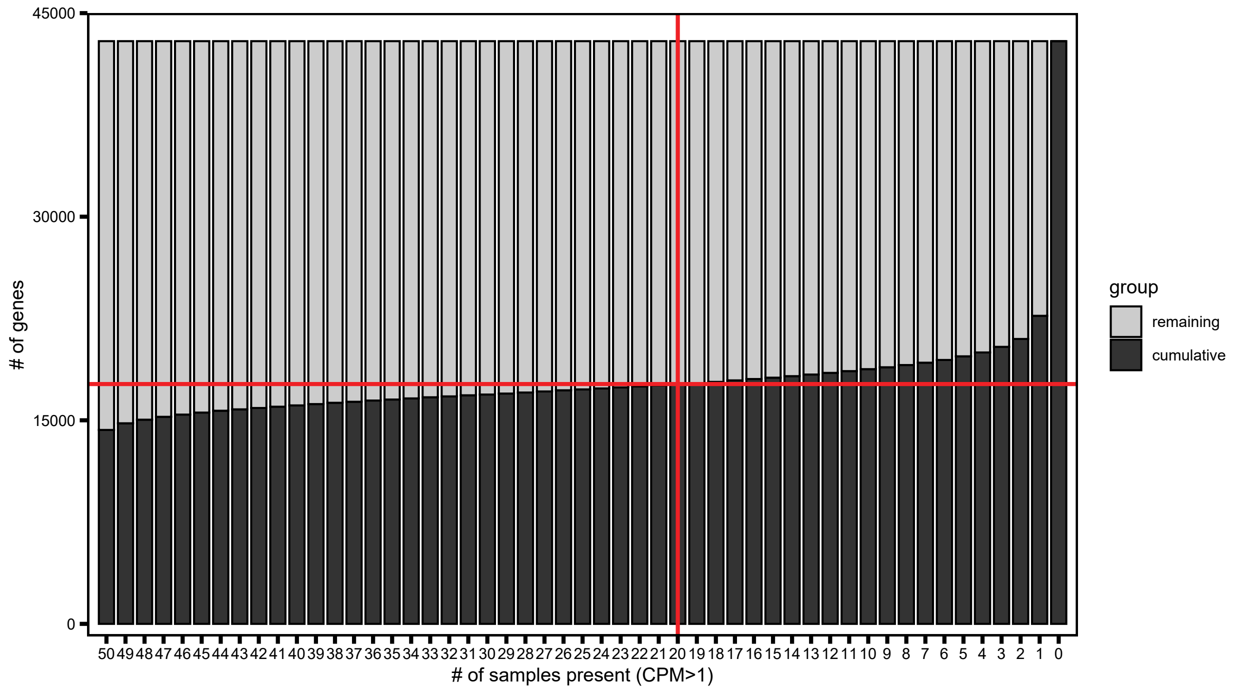


```
tokeep <- rowSums(abovethresh) >= 20
paste("Pre-filtering gene count:", length(tokeep))
```

```
## [1] "Pre-filtering gene count: 42934"
```

```
paste("Genes below abundance threshold:", length(tokeep)-sum(tokeep))
```

```
## [1] "Genes below abundance threshold: 25264"
```

```
paste("Remaining genes:", sum(tokeep))
```

```
## [1] "Remaining genes: 17670"
```

```
# Filter genes
RNAseq[["filt"]][["rawdata"]] <- RNAseq$unfilt$rawdata[tokeep,]
RNAseq[["filt"]][["annotation"]] <- RNAseq$unfilt$annotation[tokeep,]
RNAseq[["filt"]][["design"]] <- RNAseq$unfilt$design
RNAseq[["filt"]][["counts"]] <- RNAseq$unfilt$counts[tokeep,]
```

```r
# Normalize
RNAseq[["filt"]][["cpm"]] <- cpm.normalize(RNAseq$filt$counts)
```

## Gene expression distribution post-filtering

```r
# Expression distribution post-filtering
df <- RNAseq$filt$counts %>%
  rownames_to_column(var = "geneID") %>%
  pivot_longer(cols = c(2:length(.)),
               names_to = "sample") %>%
  dplyr::rename(counts = value)

df$volunteer <- rep(RNAseq$filt$design$volunteer, dim(RNAseq$filt$counts)[1])
df$time <- rep(RNAseq$filt$design$time, dim(RNAseq$filt$counts)[1])
df$sample <- factor(df$sample, levels = names(RNAseq$filt$counts))

ggplot(df, aes(x=sample, y=log2(counts+1), fill=volunteer)) +
  geom_violin(scale = "area") +
  xlab("") +
  ylab(expression(Log[2](counts+1))) +
  scale_x_discrete(expand =expansion(mult = c(.02, .02))) +
  scale_y_continuous(expand =expansion(mult = c(.02, .05))) +
  theme_custom(
    axis.text.x.bottom = element_text(angle = 90, hjust = 1, vjust = 0.3),
    legend.position = "top"
  )
```

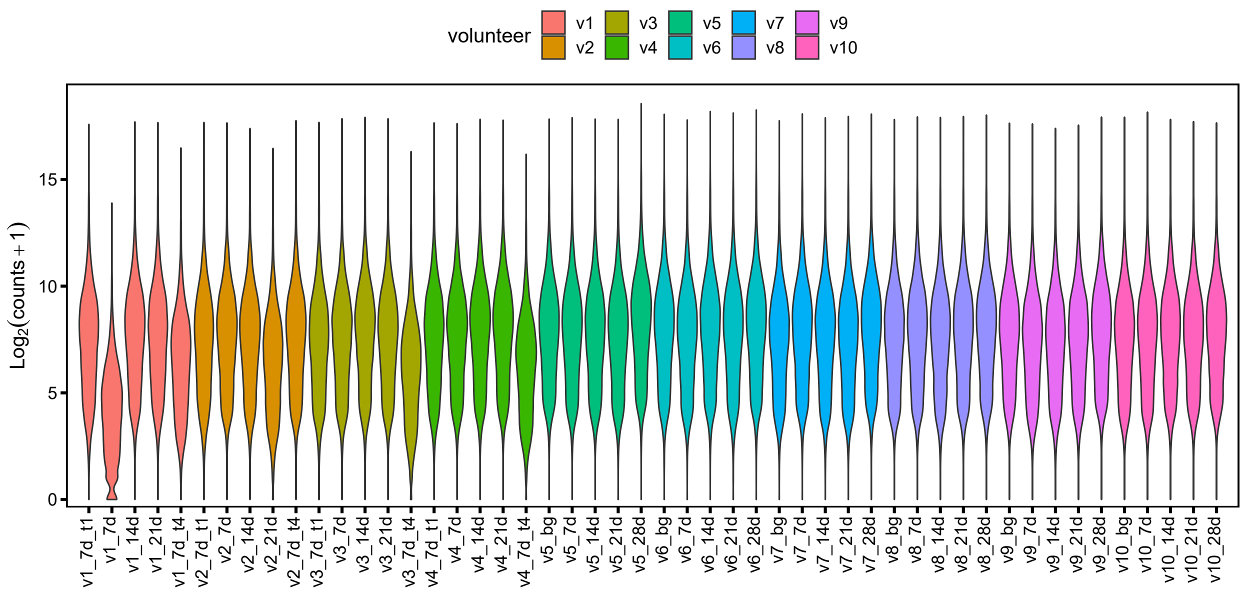


## Normalized gene expression distribution post-filtering

```r
# Normalized expression distribution post-filtering
df <- RNAseq$filt$counts %>%
  rownames_to_column(var = "geneID") %>%
  pivot_longer(cols = c(2:length(.)),
               names_to = "sample") %>%
  dplyr::rename(counts = value)

df$volunteer <- rep(RNAseq$filt$design$volunteer, dim(RNAseq$filt$counts)[1])
df$time <- rep(RNAseq$filt$design$time, dim(RNAseq$filt$counts)[1])
df$sample <- factor(df$sample, levels = names(RNAseq$filt$counts))

df$cpm <- RNAseq$filt$cpm %>%
  pivot_longer(cols = c(1:length(.)),
               names_to = "sample") %>%
  select(value) %>%
  unlist()
```

```r
ggplot(df, aes(x=sample, y=log2(cpm+1), fill=volunteer)) +
  geom_violin(scale = "area") +
  xlab("") +
  ylab(expression(Log[2](CPM+1))) +
  scale_x_discrete(expand =expansion(mult = c(.02, .02))) +
  scale_y_continuous(expand =expansion(mult = c(.02, .05))) +
  theme_custom(
    axis.text.x.bottom = element_text(angle = 90, hjust = 1, vjust = 0.3),
    legend.position = "top"
  )
```

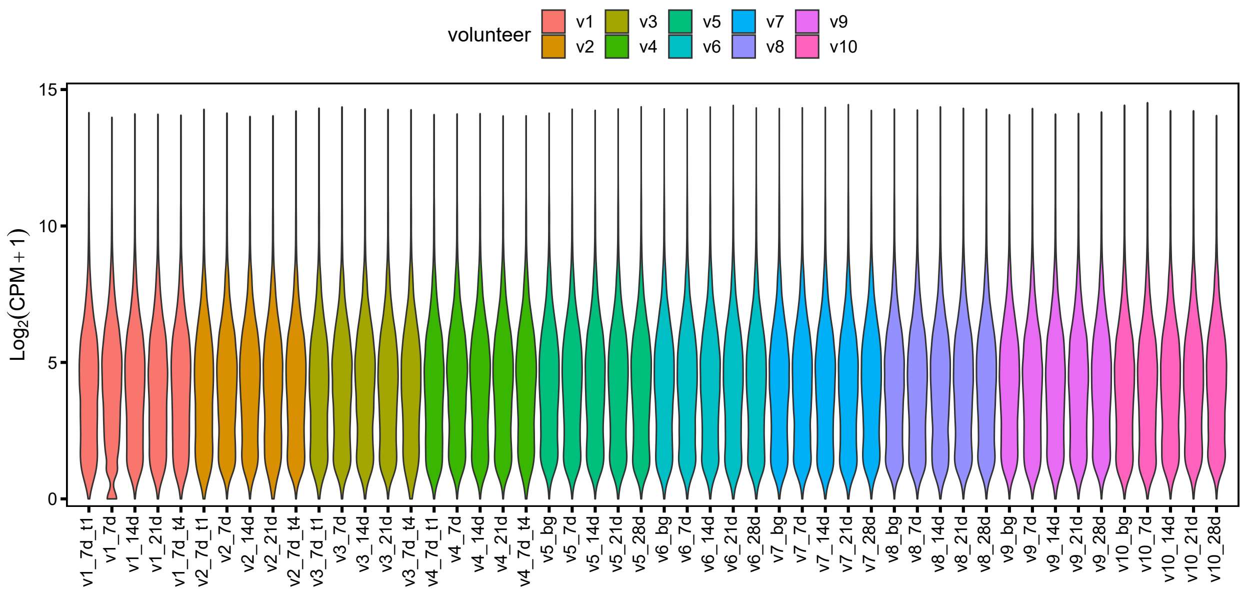


# Transcriptome differences

## Sample correlations

```r
df <- cor(log2(RNAseq$filt$cpm+1), method = "spearman") %>%
  as.data.frame() %>%
  rownames_to_column(var = "sample1") %>%
  mutate(across(everything(), as.character)) %>%
  pivot_longer(cols = c(2:length(.)),
               names_to = "sample2") %>%
  dplyr::rename(r = value) %>%
  mutate(sample1 = factor(sample1, levels = names(RNAseq$filt$counts)),
         sample2 = factor(sample2, levels = names(RNAseq$filt$counts)),
         r = as.numeric(r))

ggplot(df, aes(x=sample1, y=sample2, fill= r)) +
  geom_tile() +
  scale_y_discrete(limits=rev) +
  scale_fill_gradientn(colours = rev(colPals$inferno)) +
  theme_custom(
    axis.text.x.bottom = element_text(angle = 90, hjust = 1, vjust = 0.3),
    legend.position = "right",
    legend.justification = "top"
  )
```

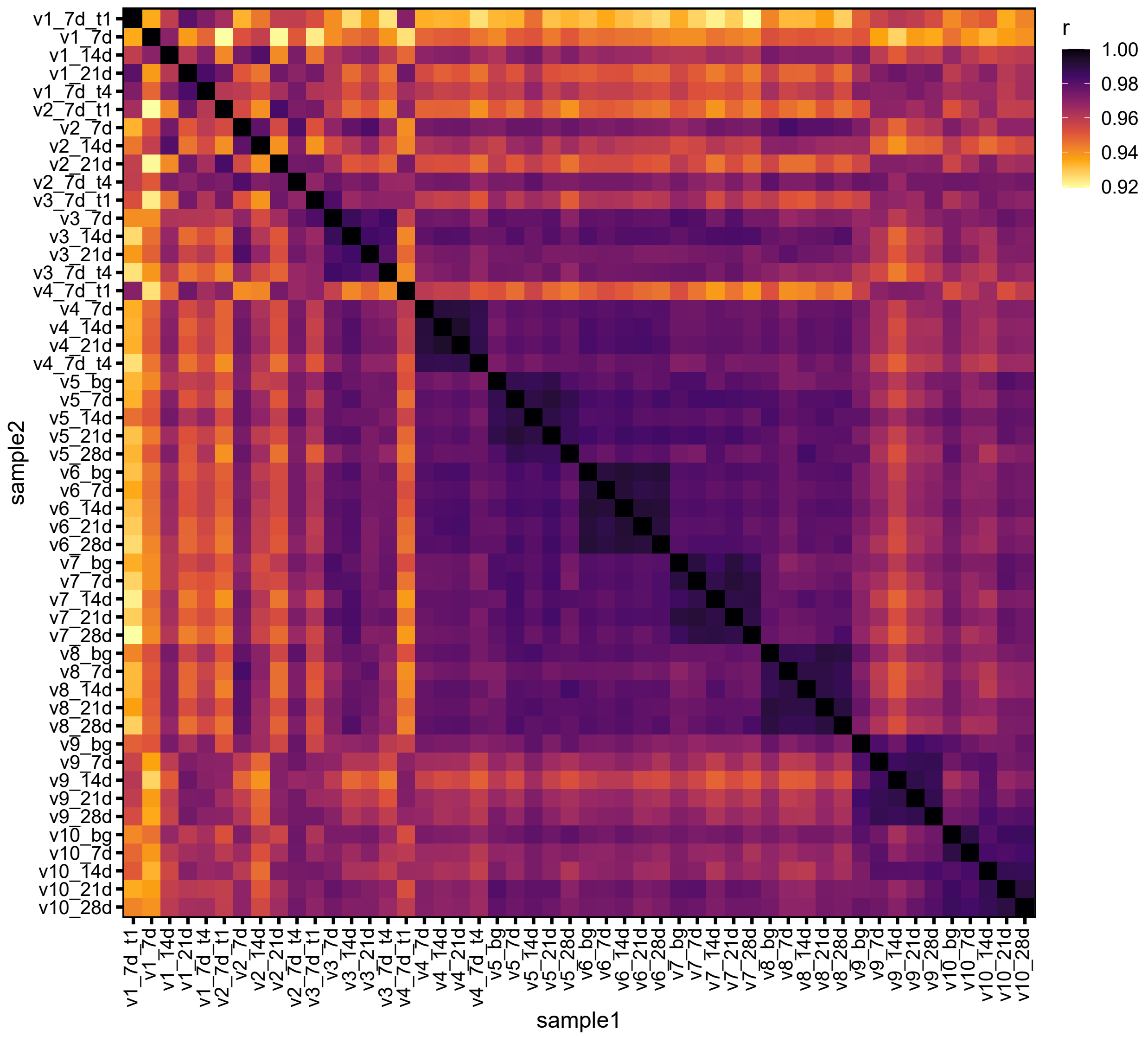

## DESeq2 analysis

```
# DESeq2 pipeline
dsData <- DESeqDataSetFromMatrix(countData = RNAseq$filt$counts,
                                 colData = RNAseq$filt$design,
                                 design = ~volunteer + time)
dsData <- estimateSizeFactors(dsData)
dsData <- DESeq(dsData, test = "LRT", reduced = ~volunteer)

RNAseq$filt[["DESeq_norm"]] <- counts(dsData, normalized=TRUE) %>% as.data.frame()
RNAseq$filt[["DESeq_vst"]] <- assay(vst(dsData, blind=FALSE)) %>% as.data.frame()
RNAseq$filt[["DESeq_rlog"]] <- assay(rlog(dsData, blind=FALSE)) %>% as.data.frame()

DESeq2_DEGs <- list(
  GLMtime = results(dsData, test = "LRT", independentFiltering = T),
  day7Vsday0 = results(dsData, contrast=c("time", "day7", "day0"), test = "Wald", independentFiltering = T),
  day14Vsday0 = results(dsData, contrast=c("time", "day14", "day0"), test = "Wald", independentFiltering = T),
  day21Vsday0 = results(dsData, contrast=c("time", "day21", "day0"), test = "Wald", independentFiltering = T),
  day28Vsday0 = results(dsData, contrast=c("time", "day28", "day0"), test = "Wald", independentFiltering = T)
)
DESeq2_DEGs <- lapply(DESeq2_DEGs, as.data.frame)
DESeq2_DEGs$GLMtime <- DESeq2_DEGs$GLMtime %>%
  add_column(logFC_day7Vsday0 = DESeq2_DEGs$day7Vsday0$log2FoldChange, .before = "log2FoldChange") %>%
  add_column(logFC_day14Vsday0 = DESeq2_DEGs$day14Vsday0$log2FoldChange, .before = "log2FoldChange") %>%
  add_column(logFC_day21Vsday0 = DESeq2_DEGs$day21Vsday0$log2FoldChange, .before = "log2FoldChange") %>%
```

```
  add_column(logFC_day28Vsday0 = DESeq2_DEGs$day28Vsday0$log2FoldChange, .before = "log2FoldChange") %>%
  select(-log2FoldChange)
DESeq2_DEGs <- lapply(DESeq2_DEGs, function(x) mutate(x, padj=ifelse(is.na(padj), 1, padj)))
DESeq2_DEGs <- lapply(DESeq2_DEGs, function(x) arrange(x, padj))
DESeq2_DEGs <- lapply(DESeq2_DEGs, rownames_to_column, var = "GeneSymbol")
DESeq2_DEGs <- lapply(DESeq2_DEGs, inner_join, y = RNAseq$filt$annotation, by = "GeneSymbol")

DESeq2_DEGs_filt <- list(
  padj_02 = lapply(DESeq2_DEGs, function(x) x %>% filter(padj<0.2)),
  padj_005 = lapply(DESeq2_DEGs, function(x) x %>% filter(padj<0.05)),
  padj_001 = lapply(DESeq2_DEGs, function(x) x %>% filter(padj<0.01))
)
```

## Batch correction

```
# Remove batch effects from data
design_mtx <- model.matrix(~time, data = RNAseq$filt$design)
RNAseq[["filt"]][["DESeq_vst_nobatch"]] <- limma::removeBatchEffect(RNAseq$filt$DESeq_vst,
                                                                    batch = dsData$volunteer,
                                                                    design = design_mtx) %>% as.data.frame()
RNAseq[["filt"]][["DESeq_rlog_nobatch"]] <- limma::removeBatchEffect(RNAseq$filt$DESeq_rlog,
                                                                     batch = dsData$volunteer,
                                                                     design = design_mtx) %>% as.data.frame()

# Plot sample correlations
df <- cor(RNAseq$filt$DESeq_vst, method = "spearman") %>%
  as.data.frame() %>%
  rownames_to_column(var = "sample1") %>%
  mutate(across(everything(), as.character)) %>%
  pivot_longer(cols = c(2:length(.)),
               names_to = "sample2") %>%
  dplyr::rename(r = value) %>%
  mutate(sample1 = factor(sample1, levels = names(RNAseq$filt$counts)),
         sample2 = factor(sample2, levels = names(RNAseq$filt$counts)),
         r = as.numeric(r))

p1 <- ggplot(df, aes(x=sample1, y=sample2, fill= r)) +
  geom_tile() +
  scale_x_discrete(labels=paste(RNAseq$filt$design$volunteer,
                                RNAseq$filt$design$time,
                                sep = '_')) +
  scale_y_discrete(limits=rev, labels=rev(paste(RNAseq$filt$design$volunteer,
                                                RNAseq$filt$design$time,
                                                sep = '_'))) +
  scale_fill_gradientn(colours = rev(colPals$inferno)) +
  xlab('') +
  ylab('') +
  ggtitle('Normalized') +
  theme_custom(
    base_size = 6,
    axis.text.x.bottom = element_text(angle = 90, hjust = 1, vjust = 0.3),
    legend.position = "none",
    plot.title = element_text(size=16, face='bold', hjust=0.5)
  )

df2 <- cor(RNAseq$filt$DESeq_vst_nobatch, method = "spearman") %>%
  as.data.frame() %>%
  rownames_to_column(var = "sample1") %>%
  mutate(across(everything(), as.character)) %>%
  pivot_longer(cols = c(2:length(.)),
               names_to = "sample2") %>%
  dplyr::rename(r = value) %>%
  mutate(sample1 = factor(sample1, levels = names(RNAseq$filt$counts)),
         sample2 = factor(sample2, levels = names(RNAseq$filt$counts)),
         r = as.numeric(r))

p2 <- ggplot(df2, aes(x=sample1, y=sample2, fill= r)) +
  geom_tile() +
  scale_x_discrete(labels=paste(RNAseq$filt$design$volunteer,
                                RNAseq$filt$design$time,
                                sep = '_')) +
  scale_y_discrete(limits=rev, labels=rev(paste(RNAseq$filt$design$volunteer,
                                                RNAseq$filt$design$time,
                                                sep = '_'))) +
  scale_fill_gradientn(colours = rev(colPals$inferno)) +
  xlab('') +
  ylab('') +
  ggtitle('Normalized & batch-corrected') +
```

```
  theme_custom(
    base_size = 6,
    axis.text.x.bottom = element_text(angle = 90, hjust = 1, vjust = 0.3),
    legend.position = "right",
    legend.justification = "top",
    plot.title = element_text(size=16, face='bold', hjust=0.5)
  )

p1 + p2
```

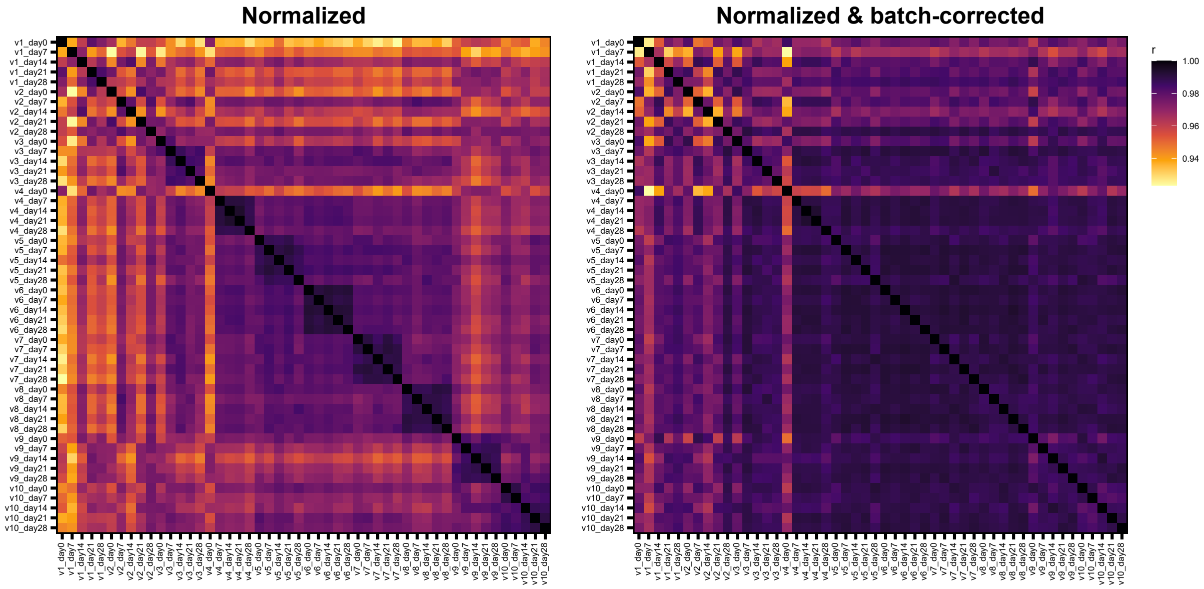


# Variance partition

## Without batch-correction

```
varPart <- fitExtractVarPartModel(as.matrix(RNAseq$filt$DESeq_vst),
                                  ~(1|batch) +(1|volunteer) + (1|time),
                                  RNAseq$filt$design)

vp <- sortCols( varPart ) %>%
  as.data.frame() %>%
  arrange(desc(`time`))

plotPercentBars(vp[1:20,]) +
  scale_fill_manual(values = colPals$factors)
```

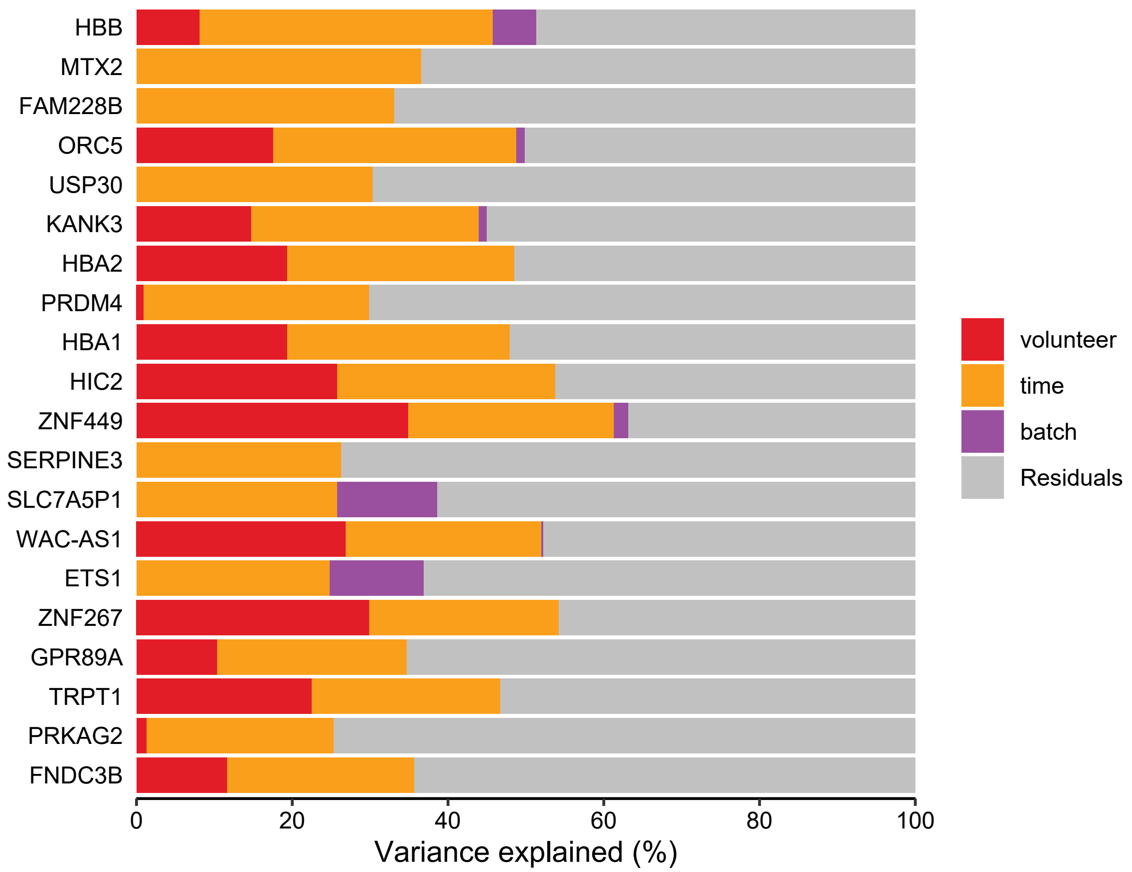


```
p3 <- plotVarPart(vp) +
  scale_fill_manual(values = colPals$factors) +
  scale_x_discrete(labels=c('Volunteer','Time','Batch','Residuals')) +
  ylab('Variance contribution (%)') +
  labs(fill = "Factor") +
  theme_bw(base_size = 16) +
  theme(
    legend.position = "none",
    axis.title.y = element_text(size=16, face='bold'),
    panel.grid.major.y = element_line(color = "grey80", linetype = "solid", size = 1.25),
    panel.grid.major.x = element_blank(),
    panel.grid.minor = element_blank(),
    panel.border = element_rect(color = "black", fill = NA, size = 2),
    axis.ticks = element_line(color = "black", size = 1.25)
  )

p3
```

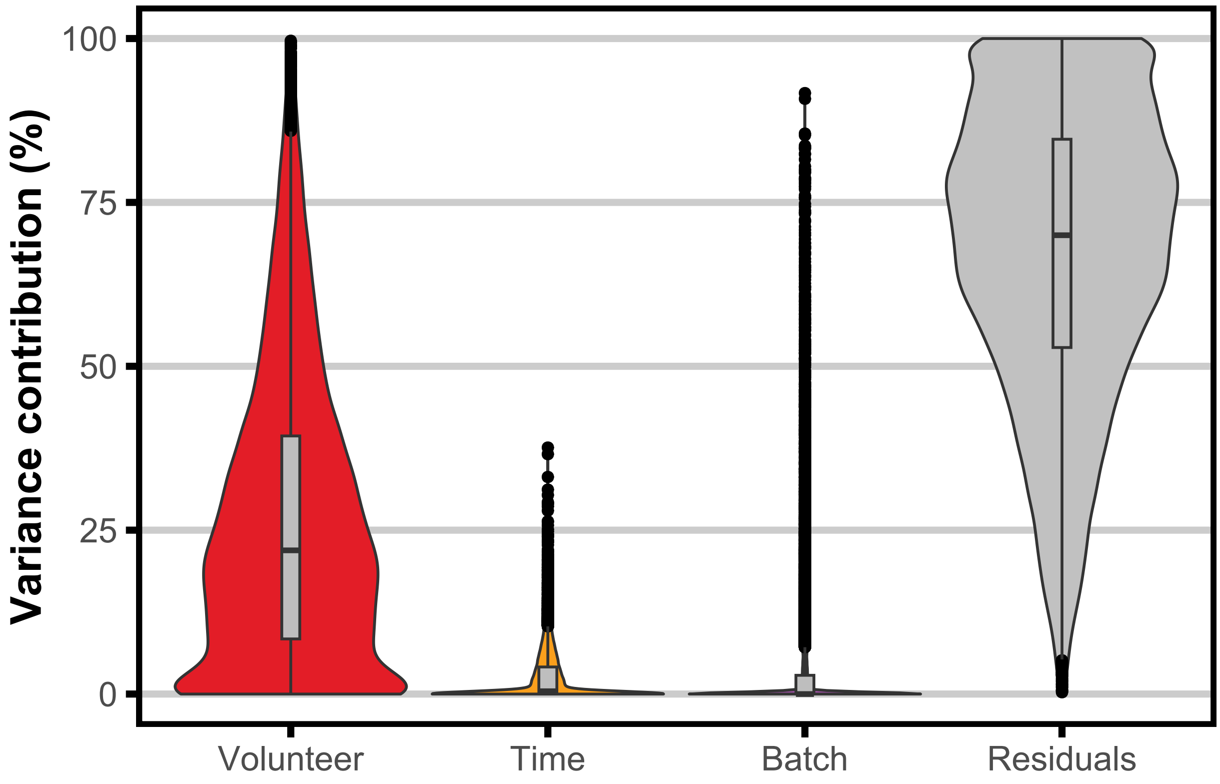


### With batch-correction

```
varPart <- fitExtractVarPartModel(as.matrix(RNAseq$filt$DESeq_vst_nobatch),
                                  ~(1|batch) +(1|volunteer) + (1|time),
                                  RNAseq$filt$design)

vp <- sortCols( varPart ) %>%
  as.data.frame() %>%
  arrange(desc(`time`))

plotPercentBars(vp[1:20,]) +
  scale_fill_manual(values = colPals$factors)
```

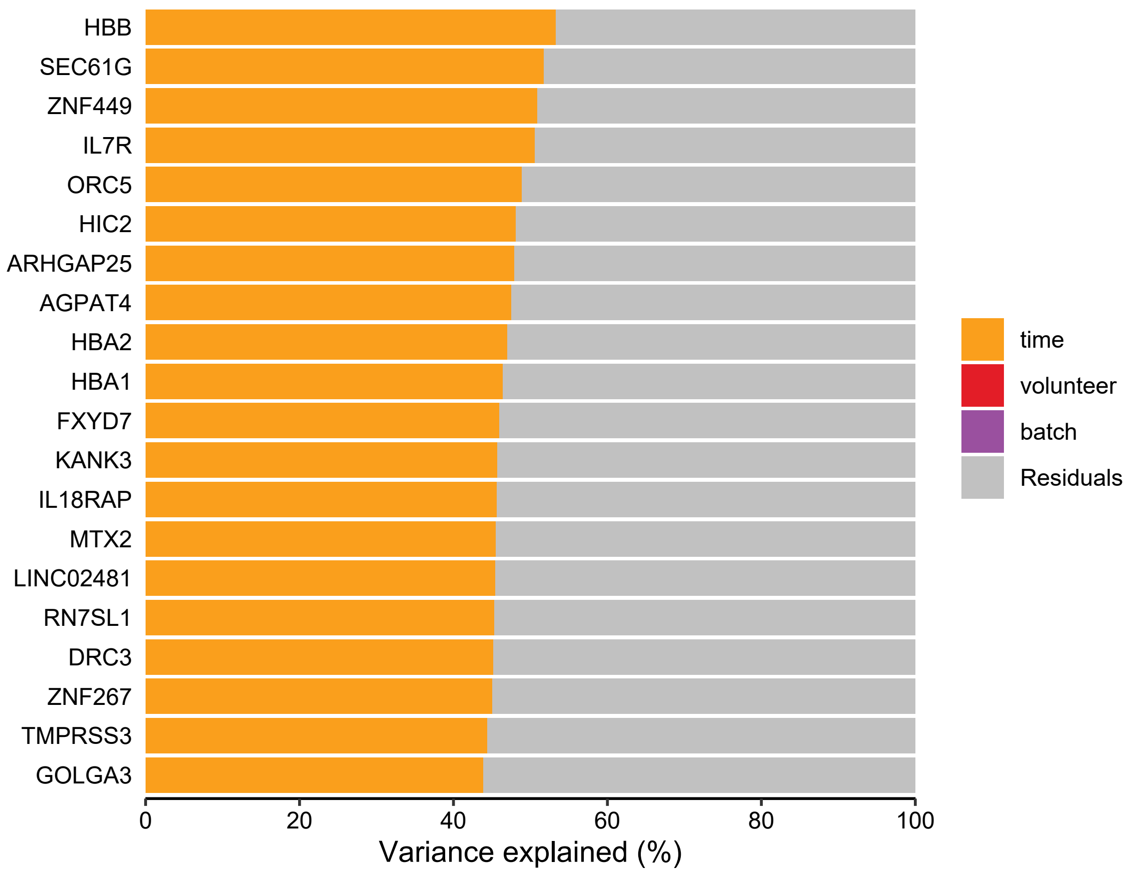


```
p4 <- plotVarPart(vp) +
  scale_x_discrete(limits = factor(names(colPals$factors),
                                   levels = names(colPals$factors)),
                   labels = c('Volunteer','Time','Batch','Residuals')) +
  scale_fill_manual(values = colPals$factors) +
  labs(fill = "Factor") +
  theme_bw(base_size = 16) +
  theme(
    legend.position = "right",
    legend.justification = "top",
    legend.text = element_text(size = 10),
    panel.grid.major.y = element_line(color = "grey80", linetype = "solid", size = 1.25),
    panel.grid.major.x = element_blank(),
    panel.grid.minor = element_blank(),
    panel.border = element_rect(color = "black", fill = NA, size = 2),
    axis.ticks = element_line(color = "black", size = 1.25)
  )

p4
```

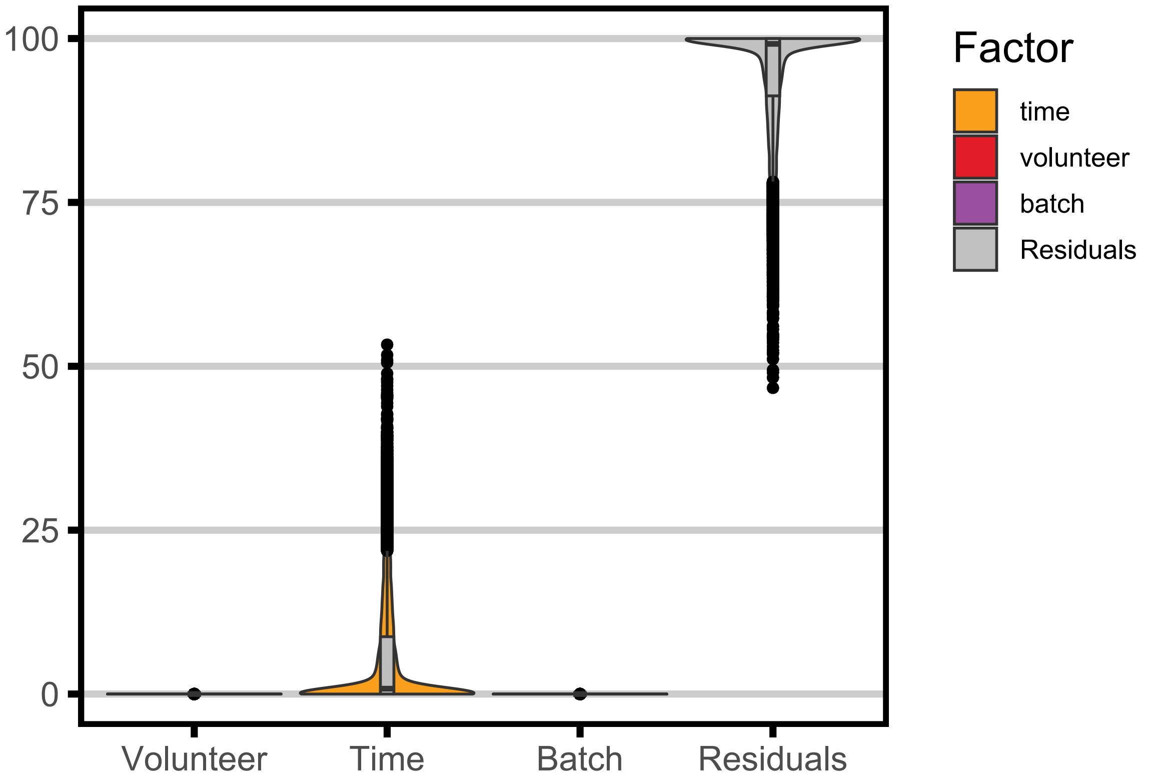


```
p <- patchwork::wrap_plots(p1,p2,p3,p4,ncol=2)

p
```

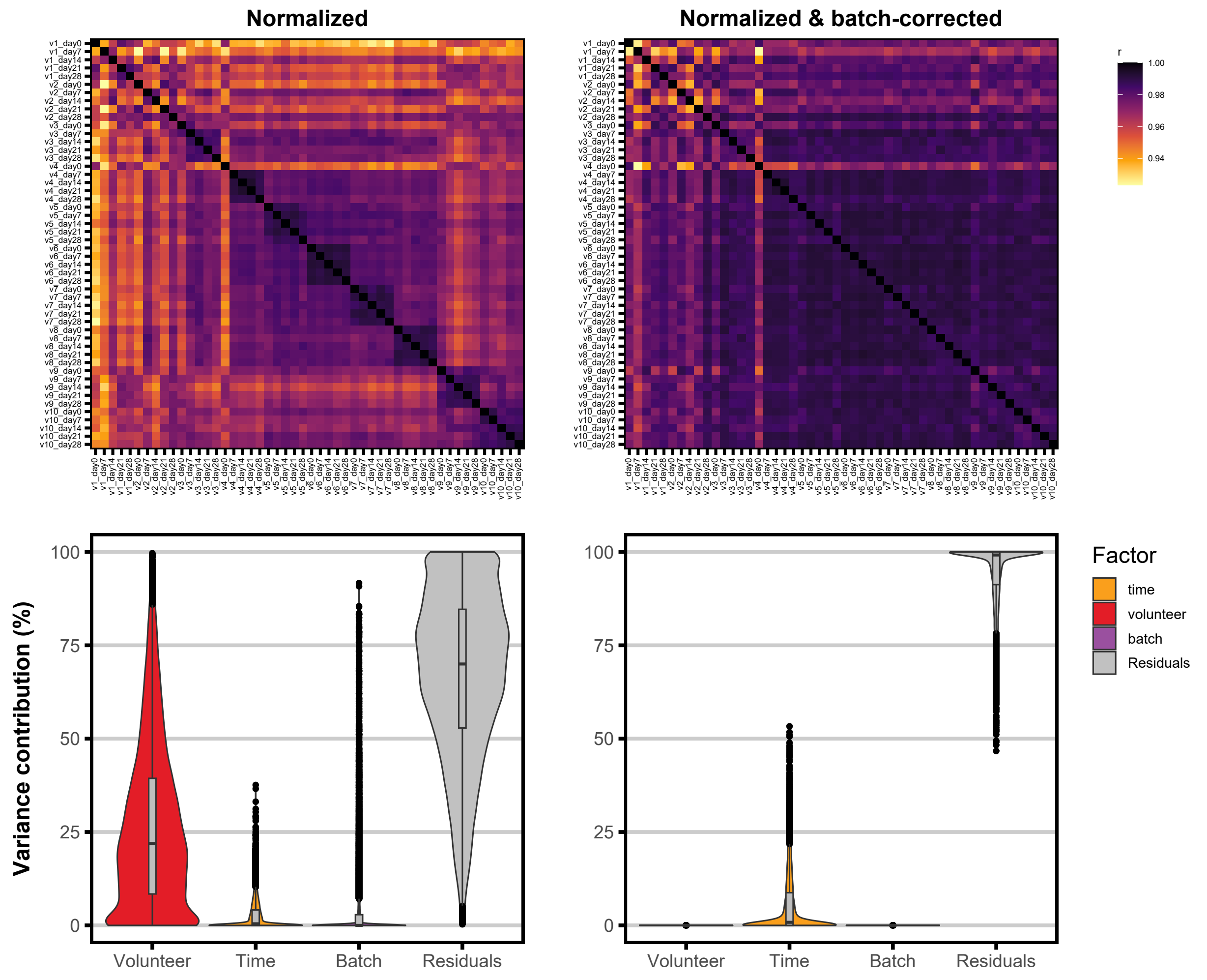


```
ggsave("plots/figS1_batch_correction.pdf", plot = p, width = 12, height = 10, units = "in", dpi = 300, device = cairo_pdf)
```

# PCA plots

## Mean

```
# PCA with mean summarisation
pca <- doPCA(RNAseq$filt$DESeq_vst_nobatch)

df <- pca$pcs %>%
  cbind(RNAseq$filt$design)

df2 <- df %>%
  group_by(time) %>%
  summarize(PC1 = mean(PC1),
            PC2 = mean(PC2),
            PC3 = mean(PC3),) %>%
  dplyr::rename(time2 = time)

ggplot() +
  geom_point(data = df, aes(x=PC1, y=PC2, color=time), shape=16, size=3, stroke=0, alpha=0.4) +
  geom_point(data = df2, aes(x=PC1, y=PC2, fill=time2), color="black", shape=23, size=6, stroke=1.5, alpha=1) +
  ggrepel::geom_label_repel() +
  xlab(paste("PC1 (", round(pca$percentVar[1],0), "%)", sep = "")) +
  ylab(paste("PC2 (", round(pca$percentVar[2],0), "%)", sep = "")) +
  scale_color_manual(values = colPals$time) +
  scale_fill_manual(values = colPals$time) +
  ggtitle('All volunteers') +
  theme_bw(base_size = 16) +
```

```
  theme(
    legend.position = "none",
    plot.title = element_text(size=16, face='bold', hjust=0.5),
    axis.title = element_text(size=16, face='bold'),
    panel.grid.major = element_blank(),
    panel.grid.minor = element_blank(),
    panel.border = element_rect(color = "black", fill = NA, size = 2),
    axis.ticks = element_line(color = "black", size = 1.25)
  )
```

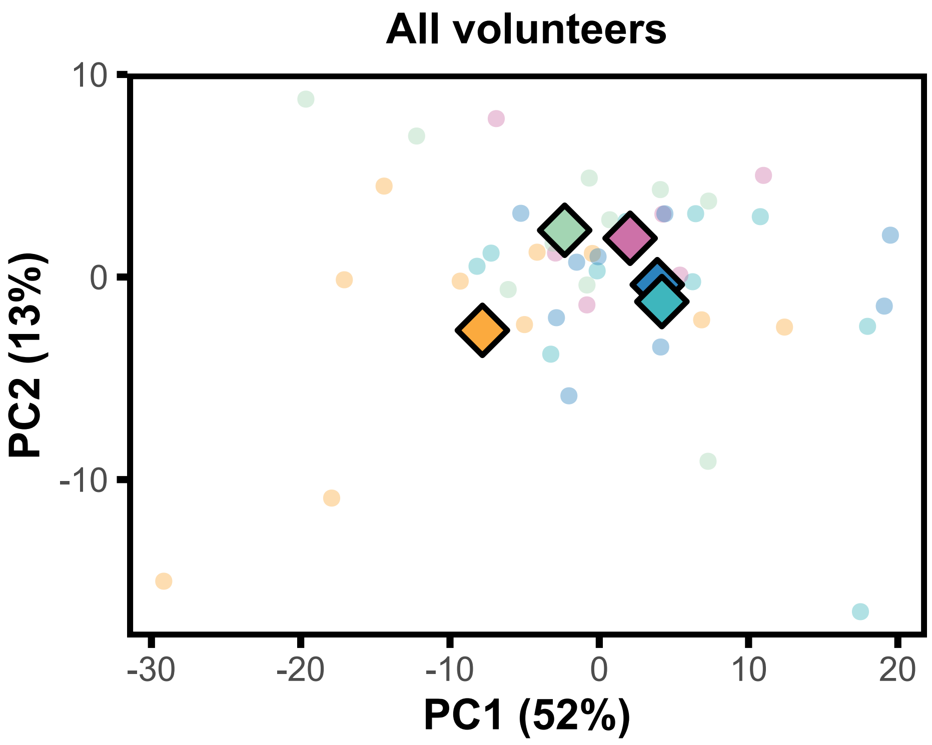


```
ggsave("plots/figS2_pca_all_volunteers.pdf", width = 5, height = 4, units = "in", dpi = 300, device = cairo_pdf)
```

### Median

```
# PCA with median summarisation
pca <- doPCA(RNAseq$filt$DESeq_vst_nobatch)

df <- pca$pcs %>%
  cbind(RNAseq$filt$design)

df2 <- df %>%
  group_by(time) %>%
  summarize(PC1 = median(PC1),
            PC2 = median(PC2),
            PC3 = median(PC3),) %>%
  dplyr::rename(time2 = time)

ggplot() +
  geom_point(data = df, aes(x=PC1, y=PC2, color=time), shape=16, size=3, stroke=0, alpha=0.4) +
  geom_point(data = df2, aes(x=PC1, y=PC2, fill=time2), color="black", shape=23, size=6, stroke=1.5, alpha=1) +
  ggrepel::geom_label_repel() +
  theme_custom(legend.position = "right") +
  xlab(paste("PC1 (", round(pca$percentVar[1],0), "%)", sep = "")) +
  ylab(paste("PC2 (", round(pca$percentVar[2],0), "%)", sep = "")) +
  scale_color_manual(values = colPals$time) +
  scale_fill_manual(values = colPals$time) +
  geom_text(data = df,aes(x=PC1,y=PC2,label=volunteer))
```

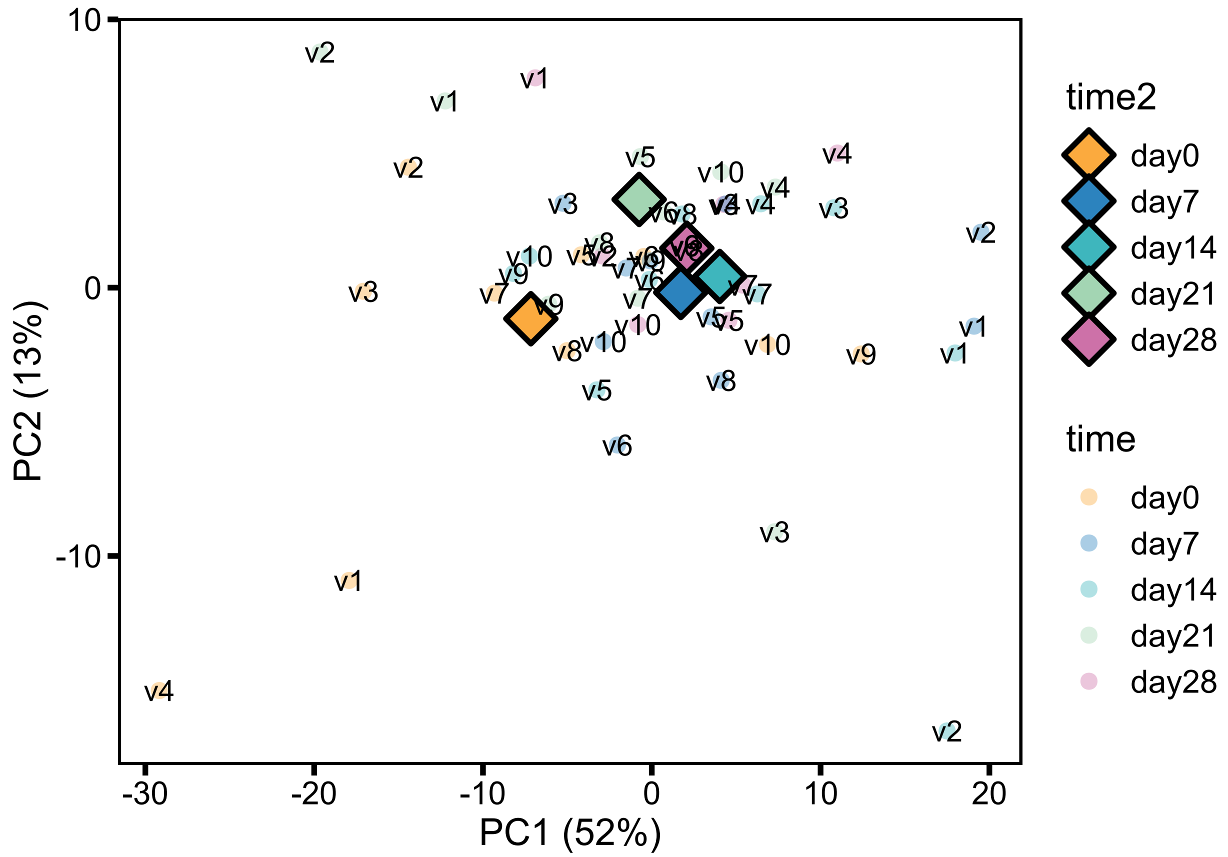


```
# PCA with median summarisation
pca <- doPCA(RNAseq$filt$DESeq_vst_nobatch)

df <- pca$pcs %>%
  cbind(RNAseq$filt$design)

df2 <- df %>%
  group_by(time) %>%
  summarize(PC1 = median(PC1),
            PC2 = median(PC2),
            PC3 = median(PC3),) %>%
  dplyr::rename(time2 = time)

ggplot() +
  geom_point(data = df, aes(x=PC1, y=PC3, color=time), shape=16, size=3, stroke=0, alpha=0.4) +
  geom_point(data = df2, aes(x=PC1, y=PC3, fill=time2), color="black", shape=23, size=6, stroke=1.5, alpha=1) +
  ggrepel::geom_label_repel() +
  theme_custom(legend.position = "right") +
  xlab(paste("PC1 (", round(pca$percentVar[1],0), "%)", sep = "")) +
  ylab(paste("PC3 (", round(pca$percentVar[3],0), "%)", sep = "")) +
  scale_color_manual(values = colPals$time) +
  scale_fill_manual(values = colPals$time) +
  geom_text(data = df,aes(x=PC1,y=PC3,label=volunteer))
```

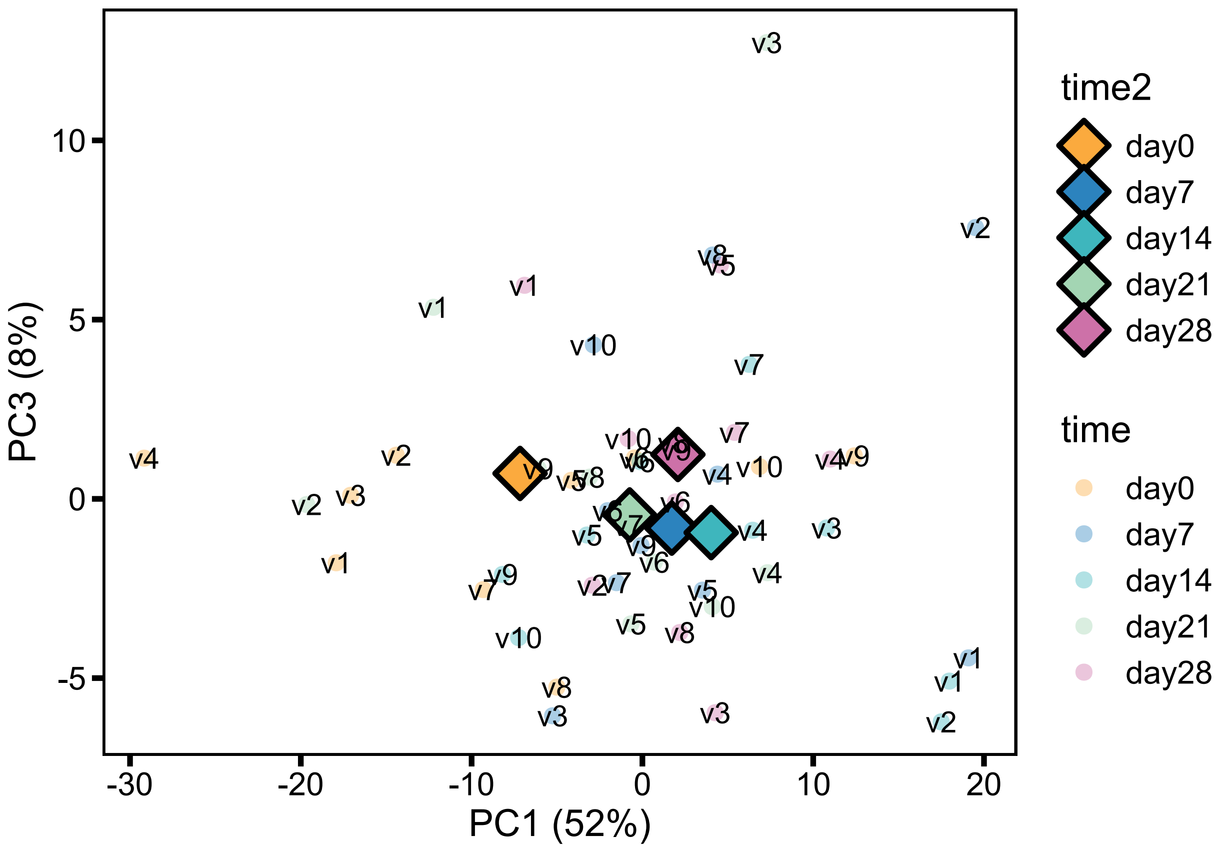


```
# boxplot with mean summarisation PC1
pca <- doPCA(RNAseq$filt$DESeq_vst_nobatch)

df <- pca$pcs %>%
  cbind(RNAseq$filt$design)

df2 <- df %>%
  group_by(time) %>%
  select(time, PC1) %>%
  summarize_each(dplyr::funs(mean, sd, se=sd(.)/sqrt(n())), PC1) %>%
  dplyr::rename(time2 = time)

ggplot() +
  geom_point(data = df, aes(x=time, y=PC1, color=time), shape=16, size=3, stroke=0, alpha=0.4) +
  geom_text(data = df,aes(x=time,y=PC1,label=volunteer),hjust=1)+
  geom_errorbar(data=df2, aes(x=time2, y=mean, ymin=mean-se, ymax=mean+se), width=.1, lwd=1) +
  geom_point(data = df2, aes(x=time2, y=mean, fill=time2), color="black", shape=21, size=8, stroke=1.5, alpha=1) +
  theme_custom(legend.position = "right") +
  ylab(paste("PC1 (", round(pca$percentVar[1],0), "%)", sep = "")) +
  scale_color_manual(values = colPals$time) +
  scale_fill_manual(values = colPals$time)
```

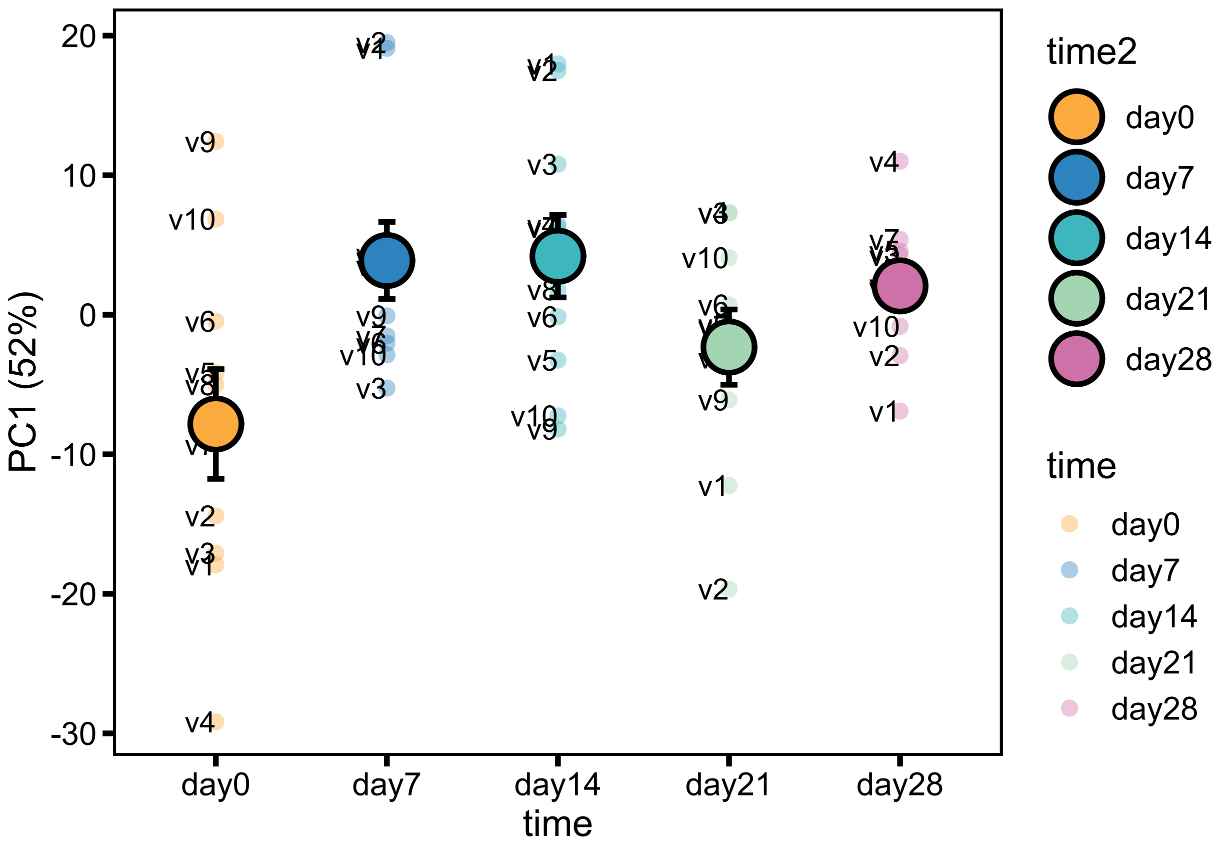


```
# boxplot with median summarisation PC1
pca <- doPCA(RNAseq$filt$DESeq_vst_nobatch)

df <- pca$pcs %>%
  cbind(RNAseq$filt$design)

df2 <- df %>%
  group_by(time) %>%
  select(time, PC1) %>%
  summarize_each(dplyr::funs(median, sd, se=sd(.)/sqrt(n())), PC1) %>%
  dplyr::rename(time2 = time)

ggplot() +
  geom_point(data = df, aes(x=time, y=PC1, color=time), shape=16, size=3, stroke=0, alpha=0.4) +
  geom_text(data = df,aes(x=time,y=PC1,label=volunteer),hjust=1)+
  geom_errorbar(data=df2, aes(x=time2, y=median, ymin=median-se, ymax=median+se), width=.1, lwd=1) +
  geom_point(data = df2, aes(x=time2, y=median, fill=time2), color="black", shape=21, size=8, stroke=1.5, alpha=1) +
  theme_custom(legend.position = "right") +
  ylab(paste("PC1 (", round(pca$percentVar[1],0), "%)", sep = "")) +
  scale_color_manual(values = colPals$time) +
  scale_fill_manual(values = colPals$time)
```

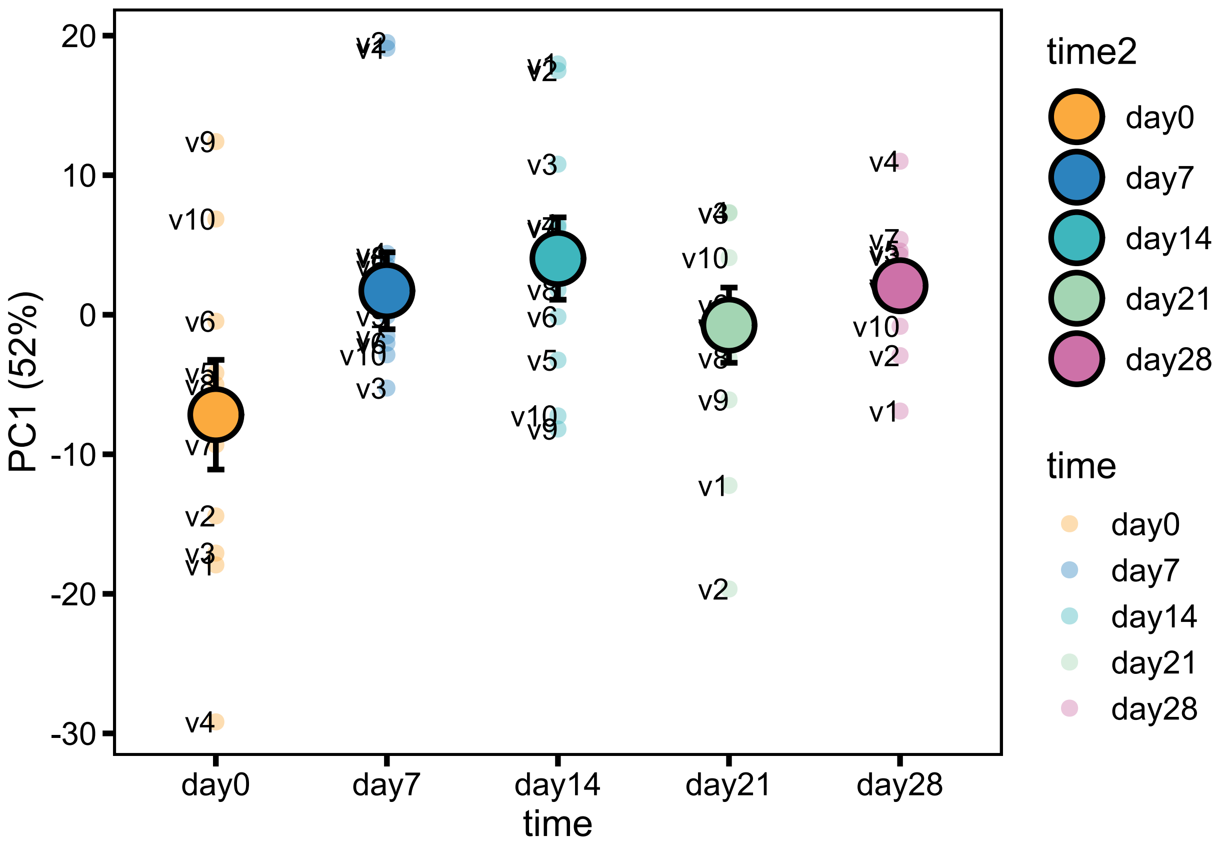


```
#boxplot with mean summarisation PC2
pca <- doPCA(RNAseq$filt$DESeq_vst_nobatch)

df <- pca$pcs %>%
  cbind(RNAseq$filt$design)

df2 <- df %>%
  group_by(time) %>%
  select(time, PC2) %>%
  summarize_each(dplyr::funs(mean, sd, se=sd(.)/sqrt(n())), PC2) %>%
  dplyr::rename(time2 = time)

ggplot() +
  geom_point(data = df, aes(x=time, y=PC2, color=time), shape=16, size=3, stroke=0, alpha=0.4) +
  geom_text(data = df,aes(x=time,y=PC2,label=volunteer),hjust=1)+
  geom_errorbar(data=df2, aes(x=time2, y=mean, ymin=mean-se, ymax=mean+se), width=.1, lwd=1) +
  geom_point(data = df2, aes(x=time2, y=mean, fill=time2), color="black", shape=21, size=8, stroke=1.5, alpha=1) +
  theme_custom(legend.position = "right") +
  ylab(paste("PC2 (", round(pca$percentVar[2],0), "%)", sep = "")) +
  scale_color_manual(values = colPals$time) +
  scale_fill_manual(values = colPals$time)
```

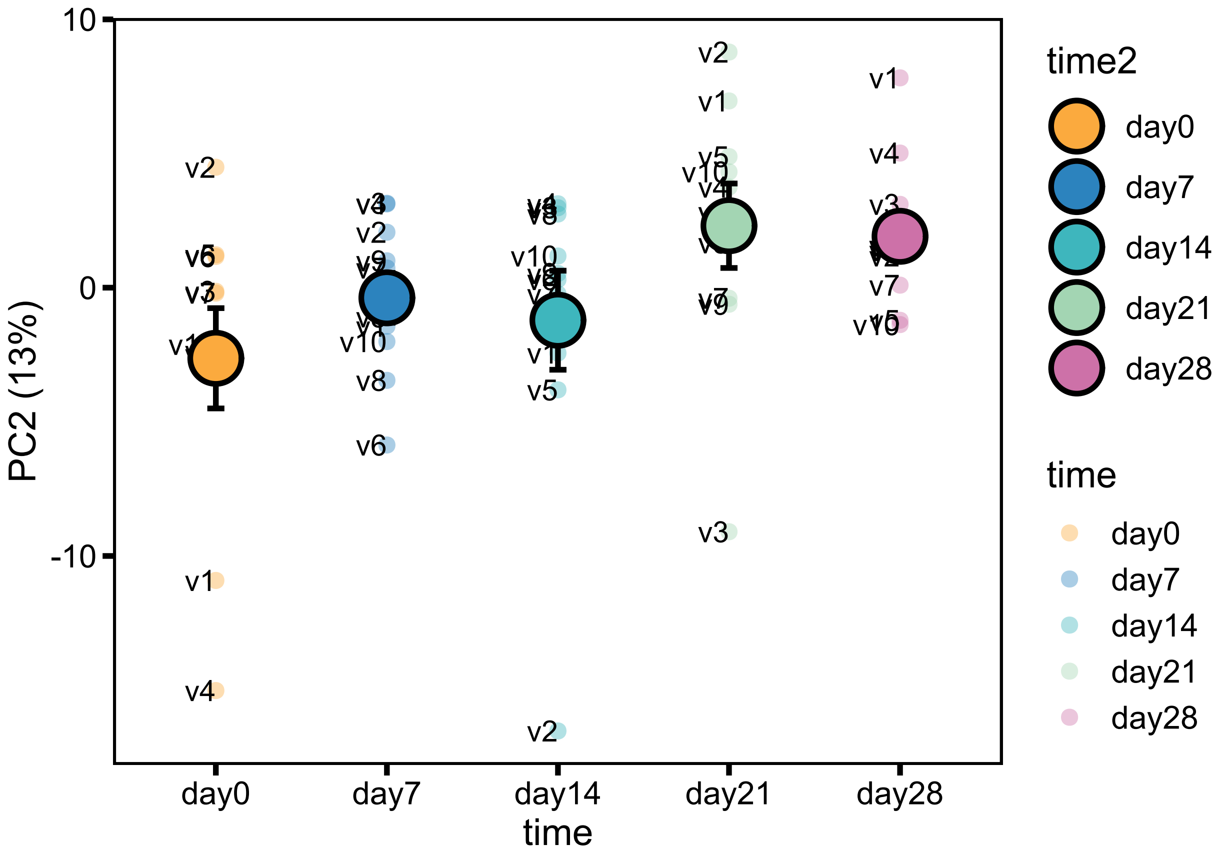


```
#boxplot with median summarisation PC2
pca <- doPCA(RNAseq$filt$DESeq_vst_nobatch)

df <- pca$pcs %>%
  cbind(RNAseq$filt$design)

df2 <- df %>%
  group_by(time) %>%
  select(time, PC2) %>%
  summarize_each(dplyr::funs(median, sd, se=sd(.)/sqrt(n())), PC2) %>%
  dplyr::rename(time2 = time)

ggplot() +
  geom_point(data = df, aes(x=time, y=PC2, color=time), shape=16, size=3, stroke=0, alpha=0.4) +
  geom_text(data = df,aes(x=time,y=PC2,label=volunteer),hjust=1)+
  geom_errorbar(data=df2, aes(x=time2, y=median, ymin=median-se, ymax=median+se), width=.1, lwd=1) +
  geom_point(data = df2, aes(x=time2, y=median, fill=time2), color="black", shape=21, size=8, stroke=1.5, alpha=1) +
  theme_custom(legend.position = "right") +
  ylab(paste("PC2 (", round(pca$percentVar[2],0), "%)", sep = "")) +
  scale_color_manual(values = colPals$time) +
  scale_fill_manual(values = colPals$time)
```

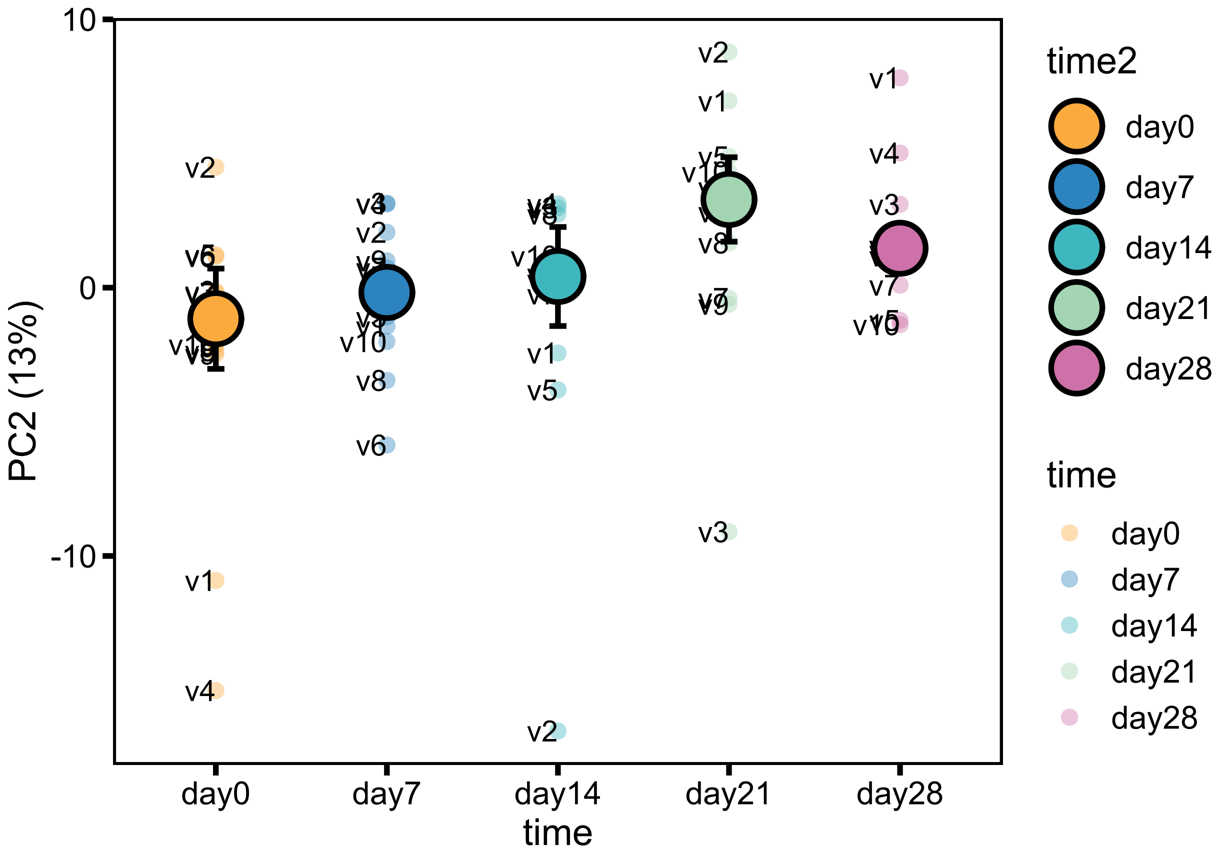


```
#boxplot with mean summarisation PC3
pca <- doPCA(RNAseq$filt$DESeq_vst_nobatch)

df <- pca$pcs %>%
  cbind(RNAseq$filt$design)

df2 <- df %>%
  group_by(time) %>%
  select(time, PC3) %>%
  summarize_each(dplyr::funs(mean, sd, se=sd(.)/sqrt(n())), PC3) %>%
  dplyr::rename(time2 = time)

ggplot() +
  geom_point(data = df, aes(x=time, y=PC3, color=time), shape=16, size=3, stroke=0, alpha=0.4) +
  geom_text(data = df,aes(x=time,y=PC3,label=volunteer),hjust=1)+
  geom_errorbar(data=df2, aes(x=time2, y=mean, ymin=mean-se, ymax=mean+se), width=.1, lwd=1) +
  geom_point(data = df2, aes(x=time2, y=mean, fill=time2), color="black", shape=21, size=8, stroke=1.5, alpha=1) +
  theme_custom(legend.position = "right") +
  ylab(paste("PC3 (", round(pca$percentVar[3],0), "%)", sep = "")) +
  scale_color_manual(values = colPals$time) +
  scale_fill_manual(values = colPals$time)
```

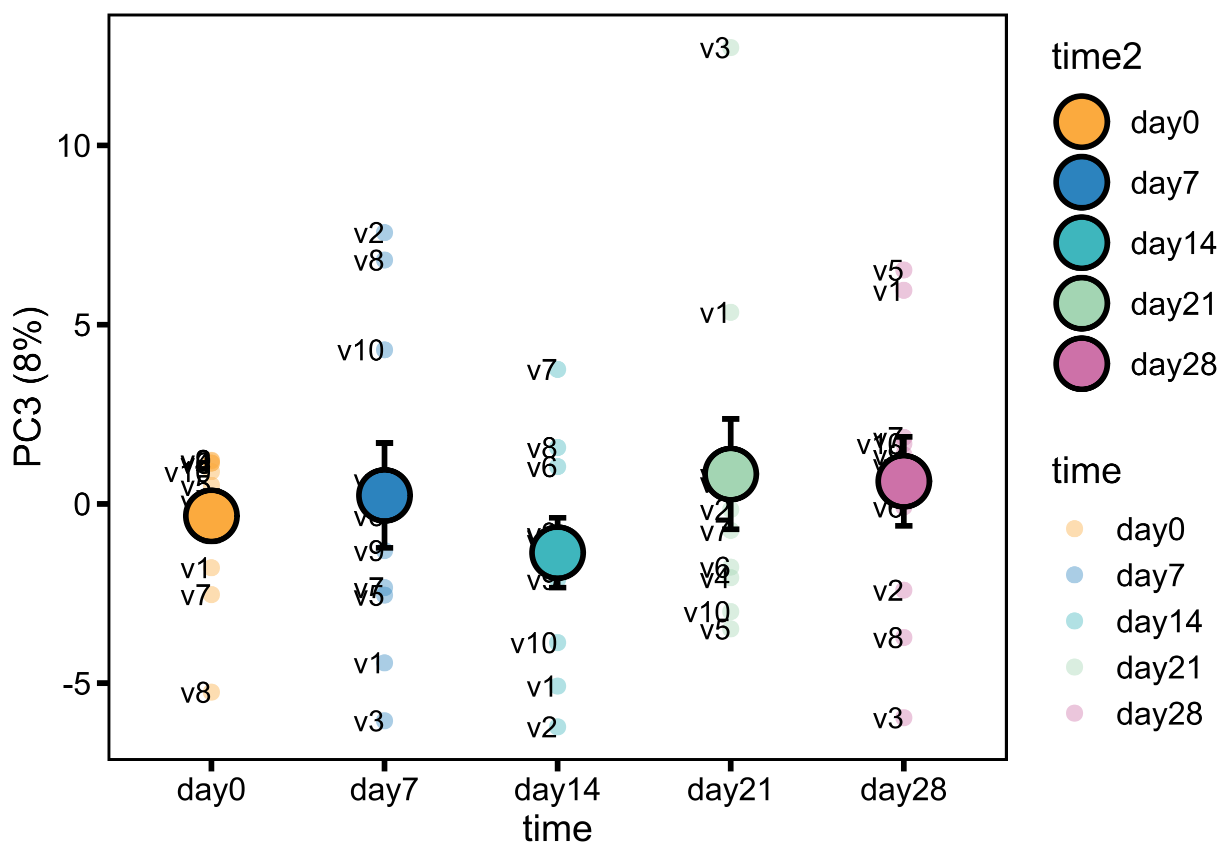


```
#boxplot with median summarisation PC3
pca <- doPCA(RNAseq$filt$DESeq_vst_nobatch)

df <- pca$pcs %>%
  cbind(RNAseq$filt$design)

df2 <- df %>%
  group_by(time) %>%
  select(time, PC3) %>%
  summarize_each(dplyr::funs(median, sd, se=sd(.)/sqrt(n())), PC3) %>%
  dplyr::rename(time2 = time)

ggplot() +
  geom_point(data = df, aes(x=time, y=PC3, color=time), shape=16, size=3, stroke=0, alpha=0.4) +
  geom_text(data = df,aes(x=time,y=PC3,label=volunteer),hjust=1)+
  geom_errorbar(data=df2, aes(x=time2, y=median, ymin=median-se, ymax=median+se), width=.1, lwd=1) +
  geom_point(data = df2, aes(x=time2, y=median, fill=time2), color="black", shape=21, size=8, stroke=1.5, alpha=1) +
  theme_custom(legend.position = "right") +
  ylab(paste("PC3 (", round(pca$percentVar[3],0), "%)", sep = "")) +
  scale_color_manual(values = colPals$time) +
  scale_fill_manual(values = colPals$time)
```

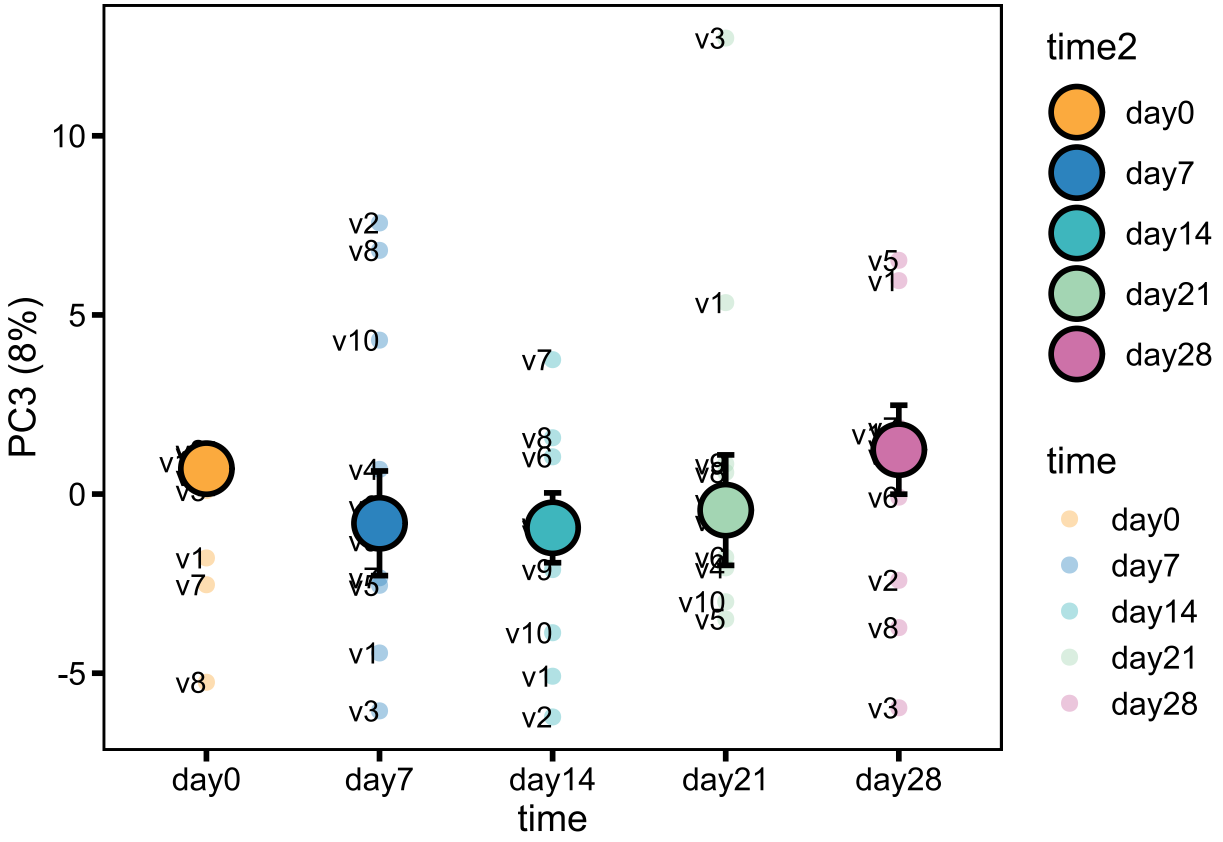


## Heatmap of DE gene fold change per volunteer

```
##  [1] "v1"  "v2"  "v3"  "v4"  "v5"  "v6"  "v7"  "v8"  "v9"  "v10"
## [1] "v1"
## [1] "v2"
## [1] "v3"
## [1] "v4"
## [1] "v5"
## [1] "v6"
## [1] "v7"
## [1] "v8"
## [1] "v9"
## [1] "v10"
```

```
mark.genes <- c('CDKN1A','NR4A1','NR4A2','MYADM','IRS2', 'CD83',
                'MCL1','XYLT1','IRF2BP2','PLEKHF2','IER5','SLC35F6')

m <- FCOutBg[rownames(FCOutBg) %in% DESeq2_DEGs_filt$padj_02$GLMtime$GeneSymbol,]

volunteer <- gsub('(v\\d+) .*$', '\\1', colnames(m))
comparison <- gsub('_', ' Vs ', gsub('v\\d+ ', '', colnames(m)))

ha_top <- HeatmapAnnotation(
  Volunteer = factor(volunteer, levels = unique(volunteer)),
  Comparison = factor(comparison, levels = unique(comparison)),
  col = list(
    Volunteer = setNames(brewer.pal(length(unique(volunteer)),"Set3"),
                     nm = unique(volunteer)),
    Comparison = setNames(brewer.pal(length(unique(comparison)),"Dark2"),
                     nm = unique(comparison))
  ),
  annotation_name_gp = gpar(fontface = 'bold'),
  border = T
)

ha_right <- rowAnnotation(
  mark = anno_mark(at=which(rownames(m) %in% mark.genes),
```

```
                    labels = rownames(m)[which(rownames(m) %in% mark.genes)],
                    padding = unit(1,"mm"))
)

hc_tree <- hclust(dist(m, method = 'euclidean'), method = 'complete')
clust <- cutree(hc_tree, k=3)

p <- Heatmap(m, name = "log2FC",
             row_split=factor(clust, levels = c(3,2,1)), cluster_row_slices = F, cluster_rows = T,
             column_title = NULL, column_split=factor(volunteer, levels = unique(volunteer)), cluster_columns = F,
             col = circlize::colorRamp2(breaks=seq(-1, 1, length.out=21),
                                        colors=colorRampPalette(c("#2166AC", "white", "#B2182B"))(21)),
             top_annotation = ha_top,
             right_annotation = ha_right,
             width = unit(170, "mm"),
             show_row_names = F, row_title = NULL, show_row_dend = T, row_dend_width=unit(10, "mm"), row_gap = unit(2, "mm"),
             show_column_names = T, column_names_gp = gpar(fontsize = 10), column_gap = unit(2, "mm"),
             border = T)

draw(p, merge_legend = T, align_heatmap_legend = "heatmap_top")
```

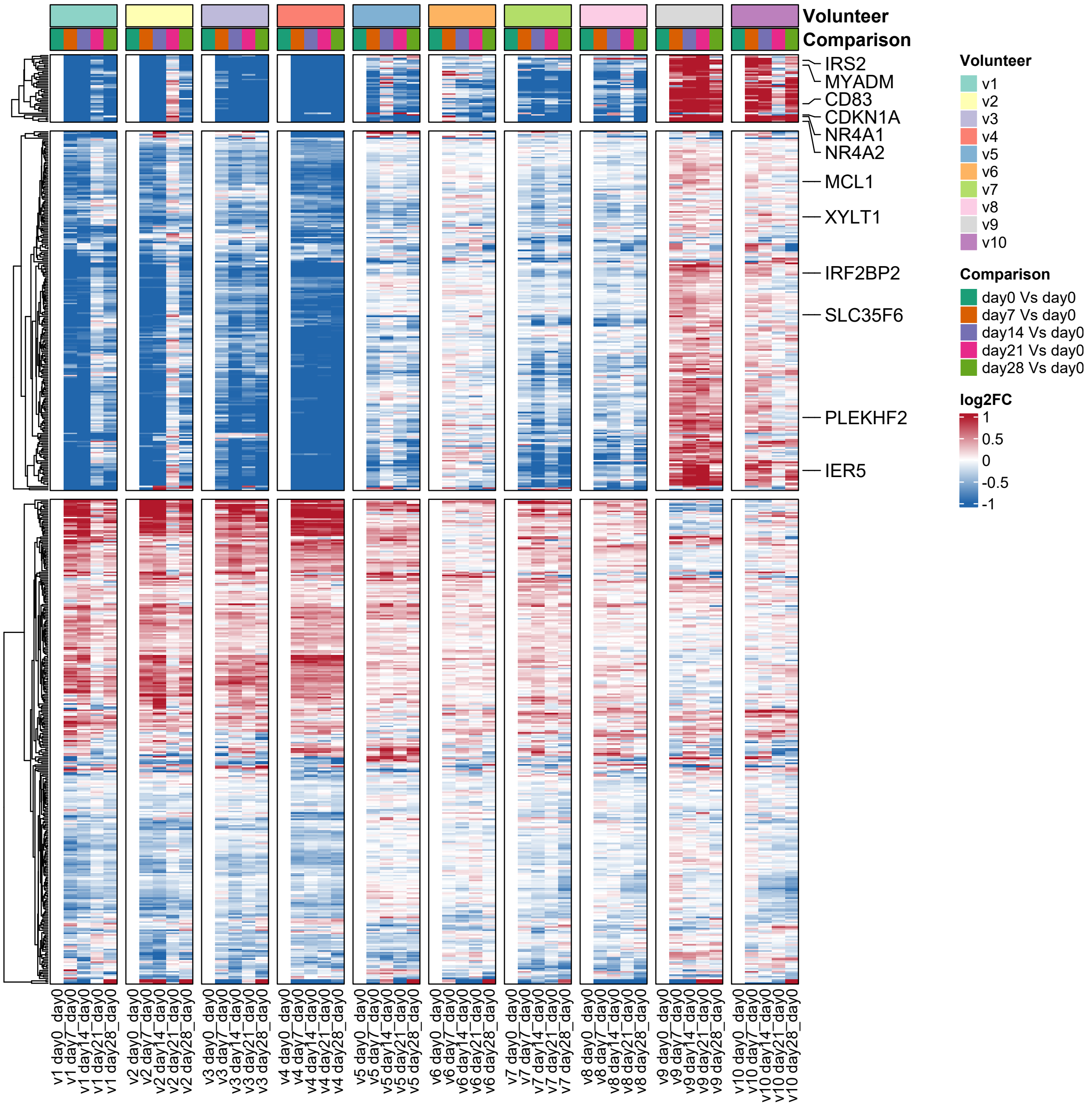

```
pdf("plots/figS3_heatmap_GLMtime_DEGs_fdr02_all_volunteers.pdf", width = 10, height = 10)
draw(p, merge_legend = T, align_heatmap_legend = "heatmap_top")
dev.off()
```

```
## cairo_pdf
##         2
```

```
# add cluster information
df <- m %>%
  as.data.frame() %>%
  rownames_to_column(var = 'GeneSymbol') %>%
  add_column(Cluster = recode(.$GeneSymbol, !!!clust), .after = 'GeneSymbol') %>%
  arrange(desc(Cluster))
colnames(df) <- gsub(' ', '_', colnames(df))

head(df)
```

```
##   GeneSymbol Cluster v1_day0_day0 v1_day7_day0 v1_day14_day0 v1_day21_day0
## 1    SNORD55       3            0    -2.555755     -3.100324    -2.2474142
## 2    GADD45A       3            0    -3.498003     -3.849928    -0.3460918
## 3     TENT5C       3            0    -5.941867     -4.415470    -0.6892786
## 4       LMNA       3            0    -3.341684     -3.122239    -1.7824649
## 5       RGS2       3            0    -2.891241     -3.197108    -1.3121941
## 6       BTG2       3            0    -2.677152     -3.203831    -0.2567009
##   v1_day28_day0 v2_day0_day0 v2_day7_day0 v2_day14_day0 v2_day21_day0
## 1    -1.0596099            0    -2.361799     -4.288352    0.54818444
## 2    -1.4872851            0    -3.881534     -4.078899    0.64532812
## 3    -1.5360862            0    -4.124591     -4.863170   -0.03369748
## 4    -1.9272633            0    -3.163733     -2.900545   -0.54052050
## 5    -1.8856238            0    -3.094777     -1.531801    0.05688757
## 6    -0.1475172            0    -3.244029     -3.054314    0.27180629
##   v2_day28_day0 v3_day0_day0 v3_day7_day0 v3_day14_day0 v3_day21_day0
## 1    -3.4971052            0   -4.2115255     -4.666718     -3.704865
## 2    -1.3191874            0   -0.9945114     -3.641905     -2.138811
## 3    -1.4524291            0   -1.6222933     -3.801901     -2.385167
## 4    -1.4564632            0   -2.4730788     -3.244047     -1.511365
## 5    -0.6457841            0   -0.8666366     -2.985528     -1.143766
## 6    -0.3284787            0   -0.6583088     -2.652504     -1.454440
##   v3_day28_day0 v4_day0_day0 v4_day7_day0 v4_day14_day0 v4_day21_day0
## 1     -4.159354            0    -4.638925    -0.4668353    -0.2943534
## 2     -2.530564            0    -4.762237    -4.8350593    -5.0677225
## 3     -4.677311            0    -5.434495    -5.3686521    -5.7826529
## 4     -2.451281            0    -4.194071    -3.7918571    -3.7018251
## 5     -2.037847            0    -3.508136    -3.3399718    -3.5262364
## 6     -1.892690            0    -2.572266    -2.7798637    -3.0511374
##   v4_day28_day0 v5_day0_day0 v5_day7_day0 v5_day14_day0 v5_day21_day0
## 1     -5.096689            0    0.9406646     0.2269535     1.6110676
## 2     -5.353752            0   -0.8049867     0.3798050    -0.3164543
## 3     -5.733997            0   -0.8953110     0.5832916    -0.7666483
## 4     -3.712691            0   -0.6821185    -0.3026732    -1.0363464
## 5     -4.608578            0   -0.6665507     0.6666530    -0.3400228
## 6     -4.139334            0   -1.3979919    -0.2881216    -0.8464381
##   v5_day28_day0 v6_day0_day0 v6_day7_day0 v6_day14_day0 v6_day21_day0
## 1    0.08364401            0   -1.1113216    -1.9952208    -1.1637307
## 2   -0.75569910            0   -0.7216933    -0.6353398    -0.9964948
## 3   -0.60395551            0   -0.2705872    -0.4928175    -0.5601680
## 4    0.26137441            0   -0.3722137    -0.4844233    -0.2769652
## 5   -0.47418236            0    0.1557997     0.2824366    -0.3330260
## 6   -1.14626092            0    0.6041653    -0.3396026    -0.6406625
##   v6_day28_day0 v7_day0_day0 v7_day7_day0 v7_day14_day0 v7_day21_day0
## 1  -0.257496605            0    0.3226219    -1.6390420    -1.4882147
## 2  -0.770642757            0   -0.5551318    -0.8629478    -0.1099975
## 3  -0.682106816            0   -1.0730738    -0.9967463    -0.8703376
## 4  -0.001913474            0   -1.0953689    -0.7192124    -1.4160649
## 5  -0.321396078            0   -0.4984369    -2.0649872    -0.5909377
## 6  -0.479241690            0   -1.7743363    -3.1194326    -1.5599699
##   v7_day28_day0 v8_day0_day0 v8_day7_day0 v8_day14_day0 v8_day21_day0
## 1     -2.286330            0   -1.5910509     0.4758088   -0.10690660
## 2     -1.478975            0   -0.4434937    -0.8269000   -0.21910868
## 3     -1.264850            0    0.4848386    -0.9347691   -0.04364865
## 4     -1.105758            0    0.7388150     0.9827688    0.89128324
## 5     -2.031201            0   -0.9325318    -1.3502999   -0.31276779
## 6     -2.313138            0   -1.2774450    -1.4533614   -0.28814125
##   v8_day28_day0 v9_day0_day0 v9_day7_day0 v9_day14_day0 v9_day21_day0
## 1    -1.8172074            0   -1.2302058    -0.4438439    -0.3225177
## 2    -1.1309107            0    1.4634409     0.9988098     1.8814505
## 3    -0.4436283            0    1.4542367     1.7726102     2.0297752
## 4     0.9255282            0    0.7952818     2.1825208     2.6578440
## 5    -1.1566376            0    0.7150694     0.5704871     0.9795493
## 6    -0.9755160            0    0.5932704     1.2218654     1.2541658
##   v9_day28_day0 v10_day0_day0 v10_day7_day0 v10_day14_day0 v10_day21_day0
```

```
## 1    0.14059077            0    0.04597312     -0.5915053    -0.40348078
## 2    1.18047827            0    1.34028150      0.6660343     0.27391168
## 3    0.79671625            0    1.36796374      0.8252115    -0.53587054
## 4    0.08485952            0    1.60149823      1.0380204    -0.24140983
## 5    0.50719739            0    0.66820496      0.3004350    -0.16144176
## 6    0.76099277            0    0.49181900      0.9057045    -0.06929183
##   v10_day28_day0
## 1     -0.4564566
## 2      2.0459528
## 3      0.8452243
## 4      0.4277745
## 5      0.8635131
## 6      0.8805784
```

# Exports

```
# DE gene clusters all volunteers
openxlsx::write.xlsx(df, file = "tables/dataS1_GLMtime_DEGs_fdr02_all_volunteers.xlsx", rowNames=F, overwrite=T)

# RNAseq count table excluding volunteers 9 & 10
# df <- read.table(file = 'data/rnaseq/exon_counts_all.txt',stringsAsFactors = FALSE,sep = "\t",header = TRUE)
# df <- df[,!grepl("v10",colnames(df)) & !grepl("v9",colnames(df))]
# write.table(df, file = 'data/rnaseq/rnaseq_count_mtx.tsv', quote = F, sep = '\t', col.names = T, row.names = F)

# RNAseq design table excluding volunteers 9 & 10
# df <- RNAseq$unfilt$design[!RNAseq$unfilt$design$volunteer %in% c("v9","v10"),]
# write.table(df, file = 'data/rnaseq/rnaseq_design_mtx.tsv', quote = F, sep = '\t', col.names = T, row.names = F)
```

# SessionInfo

```
sessionInfo()
```

```
## R version 4.2.1 (2022-06-23 ucrt)
## Platform: x86_64-w64-mingw32/x64 (64-bit)
## Running under: Windows 10 x64 (build 19044)
##
## Matrix products: default
##
## locale:
## [1] LC_COLLATE=English_United States.utf8
## [2] LC_CTYPE=English_United States.utf8
## [3] LC_MONETARY=English_United States.utf8
## [4] LC_NUMERIC=C
## [5] LC_TIME=English_United States.utf8
##
## attached base packages:
## [1] grid      stats4    stats     graphics  grDevices utils     datasets
## [8] methods   base
##
## other attached packages:
##  [1] ComplexHeatmap_2.14.0       RColorBrewer_1.1-3
##  [3] variancePartition_1.28.9    BiocParallel_1.32.6
##  [5] limma_3.54.2                DESeq2_1.38.3
##  [7] SummarizedExperiment_1.28.0 Biobase_2.58.0
##  [9] MatrixGenerics_1.10.0       matrixStats_0.63.0
## [11] GenomicRanges_1.50.2        GenomeInfoDb_1.34.9
## [13] IRanges_2.32.0              S4Vectors_0.36.2
## [15] BiocGenerics_0.44.0         patchwork_1.1.2
## [17] magrittr_2.0.3              forcats_1.0.0
## [19] stringr_1.5.0               dplyr_1.1.1
## [21] purrr_1.0.1                 readr_2.1.4
## [23] tidyr_1.3.0                 tibble_3.2.1
## [25] ggplot2_3.4.2               tidyverse_1.3.2
##
## loaded via a namespace (and not attached):
##   [1] readxl_1.4.2           backports_1.4.1        circlize_0.4.15
##   [4] plyr_1.8.8             remaCor_0.0.11         splines_4.2.1
##   [7] digest_0.6.31          foreach_1.5.2          htmltools_0.5.5
##  [10] fansi_1.0.4            memoise_2.0.1          googlesheets4_1.1.0
##  [13] cluster_2.1.3          doParallel_1.0.17      aod_1.3.2
##  [16] openxlsx_4.2.5.1       tzdb_0.3.0             Biostrings_2.66.0
##  [19] annotate_1.76.0        modelr_0.1.11          timechange_0.2.0
##  [22] prettyunits_1.1.1      colorspace_2.1-0       ggrepel_0.9.3
##  [25] blob_1.2.4             rvest_1.0.3            haven_2.5.2
##  [28] rbibutils_2.2.13       xfun_0.38              crayon_1.5.2
```

```
##  [31] RCurl_1.98-1.12        jsonlite_1.8.4         lme4_1.1-32
##  [34] iterators_1.0.14       glue_1.6.2             gtable_0.3.3
##  [37] gargle_1.3.0           zlibbioc_1.44.0        XVector_0.38.0
##  [40] GetoptLong_1.0.5       DelayedArray_0.24.0    shape_1.4.6
##  [43] scales_1.2.1           mvtnorm_1.1-3          DBI_1.1.3
##  [46] Rcpp_1.0.10            xtable_1.8-4           progress_1.2.2
##  [49] clue_0.3-64            bit_4.0.5              httr_1.4.5
##  [52] gplots_3.1.3           pkgconfig_2.0.3        XML_3.99-0.14
##  [55] farver_2.1.1           dbplyr_2.3.2           locfit_1.5-9.7
##  [58] utf8_1.2.3             tidyselect_1.2.0       labeling_0.4.2
##  [61] rlang_1.1.0            reshape2_1.4.4         AnnotationDbi_1.60.2
##  [64] munsell_0.5.0          cellranger_1.1.0       tools_4.2.1
##  [67] cachem_1.0.7           cli_3.6.1              generics_0.1.3
##  [70] RSQLite_2.3.0          broom_1.0.4            evaluate_0.20
##  [73] fastmap_1.1.1          yaml_2.3.7             RhpcBLASctl_0.23-42
##  [76] knitr_1.42             bit64_4.0.5            fs_1.6.1
##  [79] zip_2.2.2              caTools_1.18.2         KEGGREST_1.38.0
##  [82] nlme_3.1-157           xml2_1.3.3             compiler_4.2.1
##  [85] pbkrtest_0.5.2         rstudioapi_0.14        png_0.1-8
##  [88] reprex_2.0.2           clusterGeneration_1.3.7 geneplotter_1.76.0
##  [91] stringi_1.7.12         highr_0.10             lattice_0.20-45
##  [94] Matrix_1.5-3           nloptr_2.0.3           vctrs_0.6.1
##  [97] pillar_1.9.0           lifecycle_1.0.3        RUnit_0.4.32
## [100] Rdpack_2.4             GlobalOptions_0.1.2    bitops_1.0-7
## [103] R6_2.5.1               KernSmooth_2.23-20     codetools_0.2-18
## [106] boot_1.3-28            MASS_7.3-57            gtools_3.9.4
## [109] rjson_0.2.21           withr_2.5.0            GenomeInfoDbData_1.2.9
## [112] parallel_4.2.1         hms_1.1.3              minqa_1.2.5
## [115] rmarkdown_2.21         googledrive_2.1.0      lubridate_1.9.2
```

# Step 1.2: Cell type deconvolution

Carlos Gallardo & Christian Oertlin

17 April, 2023

```
# Import libraries and helper functions
source("code/helper_functions.R")
library(tidyverse)
library(magrittr)
library(patchwork)
library(immunedeconv)
library(RColorBrewer)
library(broom)

# Colors
colPals <- vector(mode = "list")
colPals$time <- setNames(c("#FBAA3E", "#2C83BE", "#3EB6BD", "#A3D5B3", "#CD71A8"),
                         nm = c("day0", "day7", "day14", "day21", "day28"))
colPals$time_light <- setNames(c("#FDD6A1", "#A2CDE9", "#AFE2E5", "#DDF0E3", "#E8BDD6"),
                               nm = c("day0", "day7", "day14", "day21", "day28"))
colPals$time_dark <- setNames(c("#D87E04", "#174564", "#1F5C60", "#49A065", "#AA3C7E"),
                              nm = c("day0", "day7", "day14", "day21", "day28"))
colPals$inferno <- c("#000004", "#420A68", "#932667", "#DD513A", "#FCA50A", "#FCFFA4")
colPals$blood_cells <- setNames(c("#E54D34","#77A2D5","#B58B80"),
                                nm = c("granulocytes", "lymphocytes", "monocytes"))
colPals$cell_types <- setNames(c("#83D1F6","#FBAA3E","#FCCA7C","#B58B80","#E54D34",
                                 "#B3177E","#9A509F","#77A2D5","#CAC1DD","#36B449","#C1C1C1"),
                               nm = c("B cell", "Macrophage M1", "Macrophage M2",
                                      "Monocyte", "Neutrophil", "NK cell",
                                      "T cell CD4+ (non-regulatory)", "T cell CD8+",
                                      "T cell regulatory (Tregs)", "Myeloid dendritic cell",
                                      "uncharacterized cell"))
```

# Load data

```
# RNA-seq
RNAseq <- readRDS(file='data/rnaseq/rnaseq_volunteers_9&10_excl.rds')
DESeq2_DEGs <- readRDS(file='data/rnaseq/DESeq2_DEGs_unfilt_volunteers_9&10_excl.rds')
DESeq2_DEGs_filt <- readRDS(file='data/rnaseq/DESeq2_DEGs_filt_volunteers_9&10_excl.rds')

# FACS
facs_frac <- read.table(file = 'data/facs/cellType_percentages_facs.csv',
                        stringsAsFactors = F,
                        sep = ",",
                        header = T)
```

# Cell type fractions (FACS)

```
df <- facs_frac %>%
  pivot_longer(day0:day28, names_to = 'time', values_to = 'fraction') %>%
  filter(!(is.na(fraction))) %>%
  mutate(fraction = fraction/100,
         type = factor(type, levels = c('monocytes','lymphocytes','granulocytes')),
         time = factor(time, levels = names(colPals$time))) %>%
  group_by(time, type) %>%
  summarize_each(dplyr::funs(mean,
                             sd,
                             se=sd(.)/sqrt(n())), fraction)

df
```

```
## # A tibble: 15 x 5
## # Groups:   time [5]
```

```
##    time  type          mean      sd      se
##    <fct> <fct>         <dbl>   <dbl>   <dbl>
##  1 day0  monocytes    0.0302 0.0296  0.00935
##  2 day0  lymphocytes  0.280  0.0843  0.0267
##  3 day0  granulocytes 0.689  0.0903  0.0286
##  4 day7  monocytes    0.0188 0.00840 0.00297
##  5 day7  lymphocytes  0.296  0.0626  0.0221
##  6 day7  granulocytes 0.686  0.0668  0.0236
##  7 day14 monocytes    0.0209 0.00644 0.00204
##  8 day14 lymphocytes  0.295  0.0767  0.0243
##  9 day14 granulocytes 0.684  0.0793  0.0251
## 10 day21 monocytes    0.0178 0.00549 0.00174
## 11 day21 lymphocytes  0.302  0.0607  0.0192
## 12 day21 granulocytes 0.680  0.0638  0.0202
## 13 day28 monocytes    0.0197 0.00552 0.00175
## 14 day28 lymphocytes  0.287  0.0563  0.0178
## 15 day28 granulocytes 0.693  0.0580  0.0183
```

```
ggplot(df, aes(x=time, y=mean, group=type, fill=type)) +
  geom_bar(stat="identity", position = position_dodge(0.9), width = 0.7, size=1, color='black') +
  geom_errorbar(aes(ymin=mean, ymax=mean+se*1.96), width=.6, lwd=1, position = position_dodge(width = 0.9)) +
  scale_fill_manual(values = colPals$blood_cells) +
  scale_x_discrete(limits=rev) +
  scale_y_continuous(limits = c(0,0.8),
                     breaks = seq(0,0.6,0.2),
                     expand = expansion(mult = c(.01, .05))) +
  coord_flip() +
  xlab("") +
  ylab("Cell type fraction") +
  ggtitle("Whole blood cell fractions (FACS)") +
  theme_bw(base_size = 20) +
  theme(
    title = element_text(size=15),
    panel.grid.major.x = element_line(color = "grey80", linetype = "solid", size = 1.25),
    panel.grid.major.y = element_blank(),
    panel.grid.minor = element_blank(),
    panel.border = element_rect(color = "black", fill = NA, size = 2),
    axis.ticks = element_line(color = "black", size = 1.25),
    legend.position = 'right',
    legend.title = element_blank(),
    legend.justification = 'top',
    legend.text = element_text(size=10)
  )
```

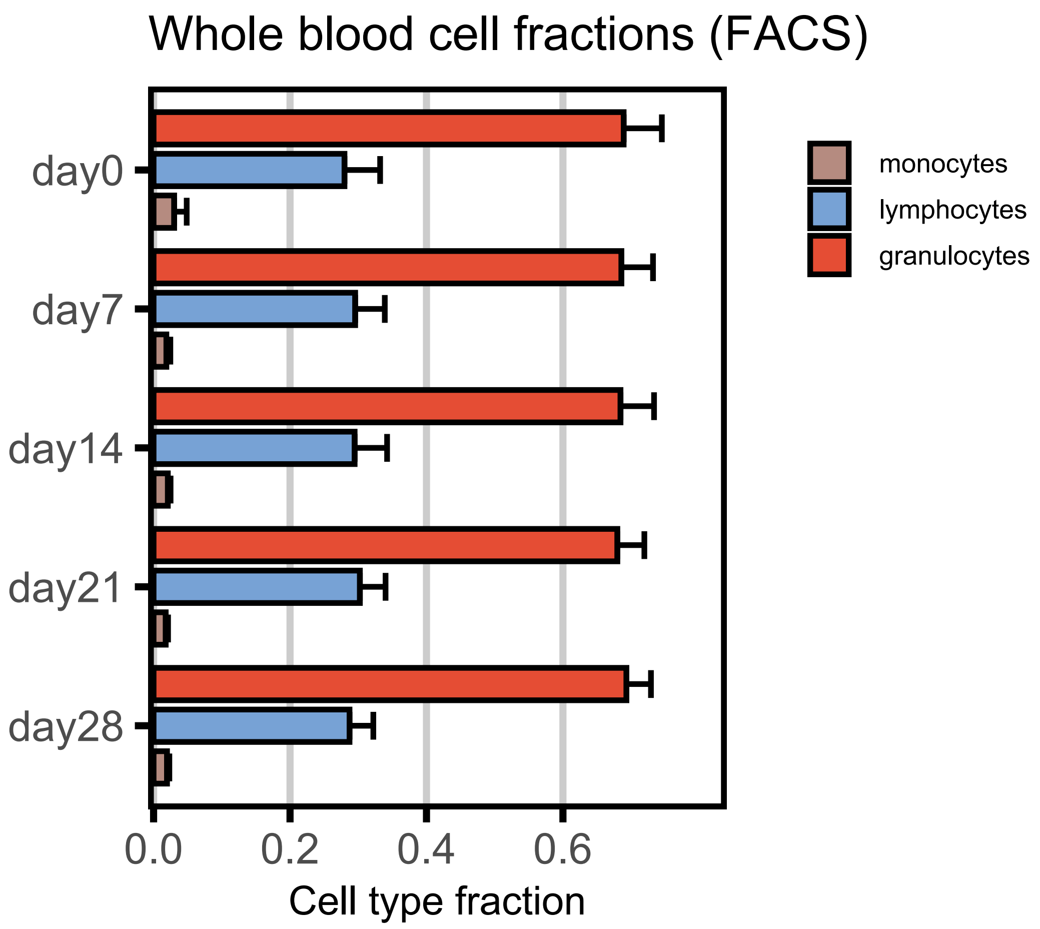


```
ggsave(filename = "plots/fig1B_whole_blood_cell_fraction.pdf", width = 6, height = 5, units = "in", dpi = 300, device = cairo_pdf)
```

# Cell type deconvolution (quantiseq)

```
# Run deconvolution
df <- RNAseq$filt$cpm %>%
  rownames_to_column(var = 'GeneSymbol') %>%
  mutate(GeneSymbol = gsub('(.+)_\\d+', '\\1', GeneSymbol)) %>%
  filter(!duplicated(GeneSymbol)) %>%
  column_to_rownames(var = 'GeneSymbol')
rnaseq_deconv <- immunedeconv::deconvolute(df, method = "quantiseq")

df <- rnaseq_deconv %>%
  column_to_rownames(var = 'cell_type') %>%
  t() %>%
  as.data.frame() %>%
  rownames_to_column(var = 'sample') %>%
  left_join(RNAseq$filt$design, by = 'sample') %>%
  pivot_longer(`B cell`:`uncharacterized cell`, names_to = 'cell_type', values_to = 'fraction')
```

## Without non-T cell types

```
df2 <- df %>%
  filter(cell_type %in% c('T cell CD4+ (non-regulatory)',
                          'T cell CD8+',
                          'T cell regulatory (Tregs)')) %>%
  mutate(cell_type = factor(cell_type,
                            levels = c('T cell CD8+',
                                       'T cell regulatory (Tregs)',
```

```
                                       'T cell CD4+ (non-regulatory)'))) %>%
  group_by(time, cell_type) %>%
  summarize_each(dplyr::funs(mean, sd, se=sd(.)/sqrt(n())), fraction)

ggplot(df2, aes(x=time, y=mean, group=cell_type, fill=cell_type)) +
  geom_bar(stat="identity", position = position_dodge(0.9), width = 0.7, size=1, color='black') +
  geom_errorbar(aes(ymin=mean, ymax=mean+se*1.96), width=.6, lwd=1, position = position_dodge(width = 0.9)) +
  scale_fill_manual(values = colPals$cell_types) +
  scale_x_discrete(limits=rev) +
  scale_y_continuous(limits = c(0,0.6),
                     breaks = seq(0,0.6,0.2),
                     expand = expansion(mult = c(.01, .05))) +
  coord_flip() +
  xlab("") +
  ylab("Cell type fraction") +
  ggtitle("RNAseq deconvolution (quantiseq)") +
  theme_bw(base_size = 20) +
  theme(
    title = element_text(size=15),
    panel.grid.major.x = element_line(color = "grey80", linetype = "solid", size = 1.25),
    panel.grid.major.y = element_blank(),
    panel.grid.minor = element_blank(),
    panel.border = element_rect(color = "black", fill = NA, size = 2),
    axis.ticks = element_line(color = "black", size = 1.25),
    legend.position = 'right',
    legend.title = element_blank(),
    legend.justification = 'top',
    legend.text = element_text(size=10)
  )
```

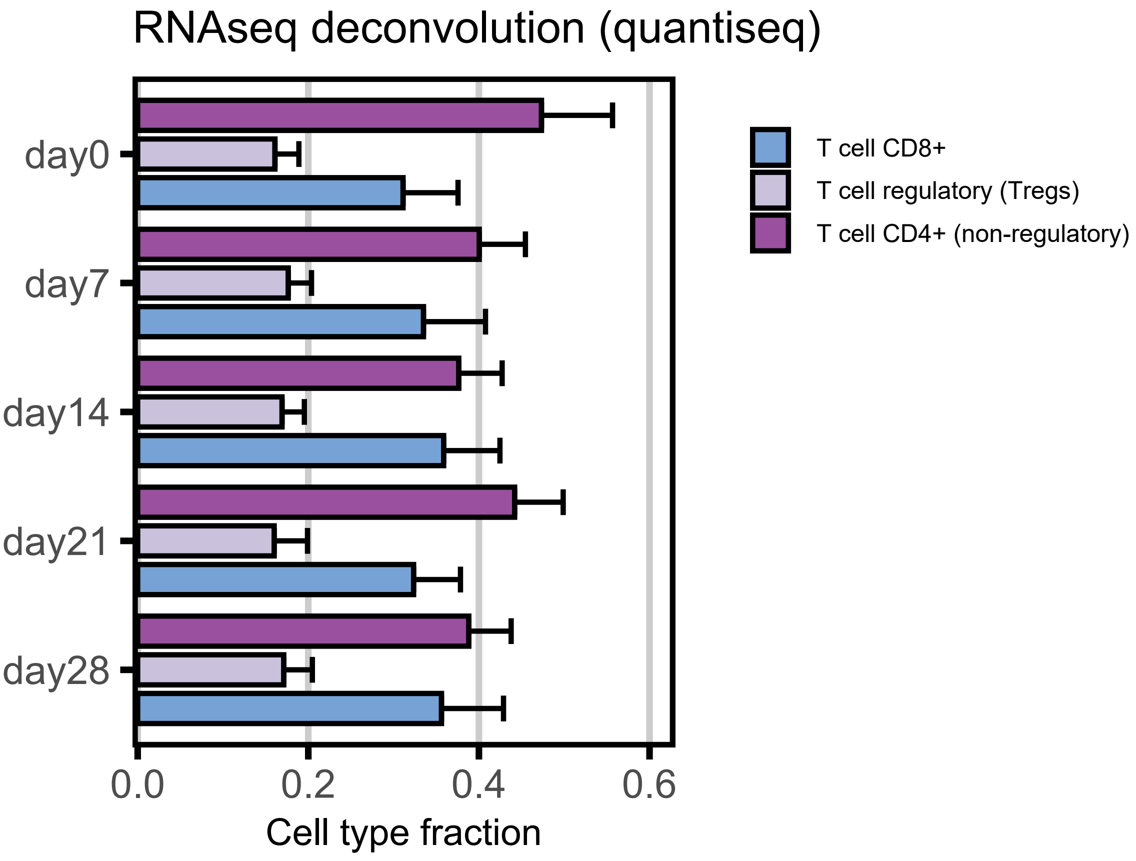


```
ggsave(filename = "plots/fig1C_cell_type_deconvolution.pdf", width = 7, height = 5, units = "in", dpi = 300, device = cairo_pdf)
```

```
# Perform paired t-tests
T_cell_types <- c('T cell CD4+ (non-regulatory)', 'T cell regulatory (Tregs)', 'T cell CD8+')
comparisons <- c('day7_day0','day14_day0','day21_day0','day28_day0')
tests_comb <- expand.grid(cell_type = T_cell_types, time_comp = comparisons) %>%
  t() %>%
  as.data.frame()
```

```
res_t_test <- lapply(tests_comb, function(x) {
  cell <- x[1]
  time1 <- str_split(x[2], pattern = '_')[[1]][1]
  time2 <- str_split(x[2], pattern = '_')[[1]][2]

  group1 <- df %>%
    filter(cell_type == cell & time == time1) %>%
    pull(fraction)

  group2 <- df %>%
    filter(cell_type == cell & time == time2) %>%
    pull(fraction)

  c(cell_type = cell,
    time_1 = time1,
    time_2 = time2,
    p_val = t.test(group1, group2, paired = T)$p.value)

}) %>% bind_rows() %>%
  mutate(p_adj = p.adjust(p_val, method = 'BH'))

res_t_test
```

```
## # A tibble: 12 x 5
##    cell_type                    time_1 time_2 p_val              p_adj
##    <chr>                        <chr>  <chr>  <chr>              <dbl>
##  1 T cell CD4+ (non-regulatory) day7   day0   0.103492933593285  0.310
##  2 T cell regulatory (Tregs)    day7   day0   0.279300080913131  0.530
##  3 T cell CD8+                  day7   day0   0.401313899684524  0.535
##  4 T cell CD4+ (non-regulatory) day14  day0   0.0182048942412375 0.212
##  5 T cell regulatory (Tregs)    day14  day0   0.610321922325501  0.667
##  6 T cell CD8+                  day14  day0   0.0353146174302014 0.212
##  7 T cell CD4+ (non-regulatory) day21  day0   0.353059297908559  0.530
##  8 T cell regulatory (Tregs)    day21  day0   0.960290547277272  0.960
##  9 T cell CD8+                  day21  day0   0.34625148468339   0.530
## 10 T cell CD4+ (non-regulatory) day28  day0   0.0582454485005906 0.233
## 11 T cell regulatory (Tregs)    day28  day0   0.611099982346776  0.667
## 12 T cell CD8+                  day28  day0   0.146916847732862  0.353
```

## Including non-T cell types

```
df2 <- df %>%
  mutate(cell_type = factor(cell_type,
                            levels = names(colPals$cell_types))) %>%
  group_by(time, cell_type) %>%
  summarize_each(dplyr::funs(mean, sd, se=sd(.)/sqrt(n())), fraction)

ggplot(df2, aes(x=time, y=mean, group=cell_type, fill=cell_type)) +
  geom_bar(stat="identity", position = position_dodge(0.9), width = 0.7, size=1, color='black') +
  geom_errorbar(aes(ymin=mean, ymax=mean+se*1.96), width=.6, lwd=1, position = position_dodge(width = 0.9)) +
  scale_fill_manual(values = colPals$cell_types) +
  scale_y_continuous(limits = c(0,0.6),
                     breaks = seq(0,0.6,0.2),
                     expand = expansion(mult = c(.01, .05))) +
  xlab("") +
  ylab("Cell type fraction (RNAseq)") +
  theme_bw(base_size = 20) +
  theme(
    title = element_text(size=15),
    panel.grid.major.y = element_line(color = "grey80", linetype = "solid", size = 1.25),
    panel.grid.major.x = element_blank(),
    panel.grid.minor = element_blank(),
    panel.border = element_rect(color = "black", fill = NA, size = 2),
    axis.ticks = element_line(color = "black", size = 1.25),
    legend.position = 'top',
    legend.title = element_blank(),
    legend.justification = 'top',
    legend.text = element_text(size=10)
  )
```

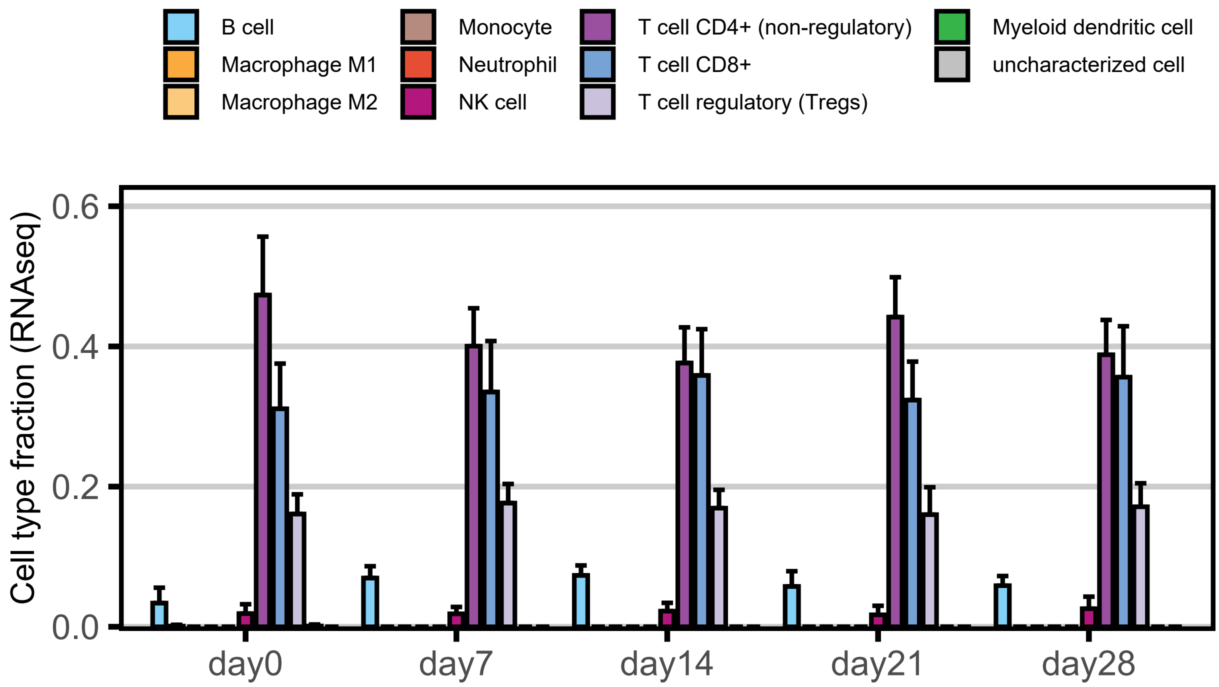


```
ggsave(filename = "plots/figS5_cell_type_deconvolution_all.pdf", width = 8, height = 5, units = "in", dpi = 300, device = cairo_pdf)
```

## Exports

```
openxlsx::write.xlsx(
  list(cell_type_fractions = df,
       t_tests = res_t_test),
  file = "tables/dataS2_RNAseq_deconvolution.xlsx",
  rowNames=F,
  overwrite=T
)
```

## SessionInfo

```
sessionInfo()
```

```
## R version 4.2.1 (2022-06-23 ucrt)
## Platform: x86_64-w64-mingw32/x64 (64-bit)
## Running under: Windows 10 x64 (build 19044)
##
## Matrix products: default
##
## locale:
## [1] LC_COLLATE=English_United States.utf8
## [2] LC_CTYPE=English_United States.utf8
## [3] LC_MONETARY=English_United States.utf8
## [4] LC_NUMERIC=C
## [5] LC_TIME=English_United States.utf8
##
## attached base packages:
## [1] stats     graphics  grDevices utils     datasets  methods   base
##
## other attached packages:
##  [1] broom_1.0.4        RColorBrewer_1.1-3 immunedeconv_2.1.0 EPIC_1.1.6
##  [5] patchwork_1.1.2    magrittr_2.0.3     forcats_1.0.0      stringr_1.5.0
##  [9] dplyr_1.1.1        purrr_1.0.1        readr_2.1.4        tidyr_1.3.0
## [13] tibble_3.2.1       ggplot2_3.4.2      tidyverse_1.3.2
```

```
##
## loaded via a namespace (and not attached):
##   [1] googledrive_2.1.0           colorspace_2.1-0
##   [3] XVector_0.38.0              GenomicRanges_1.50.2
##   [5] fs_1.6.1                    rstudioapi_0.14
##   [7] farver_2.1.1                ComICS_1.0.4
##   [9] bit64_4.0.5                 AnnotationDbi_1.60.2
##  [11] fansi_1.0.4                 lubridate_1.9.2
##  [13] xml2_1.3.3                  codetools_0.2-18
##  [15] splines_4.2.1               cachem_1.0.7
##  [17] knitr_1.42                  jsonlite_1.8.4
##  [19] annotate_1.76.0             dbplyr_2.3.2
##  [21] png_0.1-8                   data.tree_1.0.0
##  [23] compiler_4.2.1              httr_1.4.5
##  [25] backports_1.4.1             Matrix_1.5-3
##  [27] fastmap_1.1.1               gargle_1.3.0
##  [29] limma_3.54.2                cli_3.6.1
##  [31] htmltools_0.5.5             prettyunits_1.1.1
##  [33] tools_4.2.1                 gtable_0.3.3
##  [35] glue_1.6.2                  GenomeInfoDbData_1.2.9
##  [37] quantiseqr_1.6.0            rappdirs_0.3.3
##  [39] Rcpp_1.0.10                 limSolve_1.5.6
##  [41] Biobase_2.58.0              cellranger_1.1.0
##  [43] vctrs_0.6.1                 Biostrings_2.66.0
##  [45] preprocessCore_1.60.2       nlme_3.1-157
##  [47] xfun_0.38                   openxlsx_4.2.5.1
##  [49] rvest_1.0.3                 lpSolve_5.6.18
##  [51] timechange_0.2.0            lifecycle_1.0.3
##  [53] XML_3.99-0.14               googlesheets4_1.1.0
##  [55] edgeR_3.40.2                zlibbioc_1.44.0
##  [57] MASS_7.3-57                 scales_1.2.1
##  [59] MatrixGenerics_1.10.0       hms_1.1.3
##  [61] SummarizedExperiment_1.28.0 parallel_4.2.1
##  [63] yaml_2.3.7                  curl_5.0.0
##  [65] memoise_2.0.1               biomaRt_2.46.3
##  [67] stringi_1.7.12              RSQLite_2.3.0
##  [69] highr_0.10                  genefilter_1.80.3
##  [71] S4Vectors_0.36.2            BiocGenerics_0.44.0
##  [73] zip_2.2.2                   BiocParallel_1.32.6
##  [75] testit_0.13                 GenomeInfoDb_1.34.9
##  [77] rlang_1.1.0                 pkgconfig_2.0.3
##  [79] bitops_1.0-7                matrixStats_0.63.0
##  [81] evaluate_0.20               lattice_0.20-45
##  [83] bit_4.0.5                   tidyselect_1.2.0
##  [85] R6_2.5.1                    IRanges_2.32.0
##  [87] generics_0.1.3              DelayedArray_0.24.0
##  [89] DBI_1.1.3                   pillar_1.9.0
##  [91] haven_2.5.2                 withr_2.5.0
##  [93] mgcv_1.8-40                 survival_3.3-1
##  [95] KEGGREST_1.38.0             RCurl_1.98-1.12
##  [97] modelr_0.1.11               crayon_1.5.2
##  [99] utf8_1.2.3                  BiocFileCache_1.14.0
## [101] tzdb_0.3.0                  rmarkdown_2.21
## [103] mMCPcounter_1.1.0           progress_1.2.2
## [105] locfit_1.5-9.7              grid_4.2.1
## [107] readxl_1.4.2                sva_3.46.0
## [109] blob_1.2.4                  reprex_2.0.2
## [111] digest_0.6.31               xtable_1.8-4
## [113] openssl_2.0.6               stats4_4.2.1
## [115] munsell_0.5.0               quadprog_1.5-8
## [117] askpass_1.1
```

# Step 1.1: Pre-processing without volunteers 9 & 10

Carlos Gallardo & Christian Oertlin

17 April, 2023

```
# Import libraries and helper functions
source("code/helper_functions.R")
library(tidyverse)
library(magrittr)
library(patchwork)
library(DESeq2)
library(limma)
library(variancePartition)
library(RColorBrewer)

# Colors
colPals <- vector(mode = "list")
colPals$time <- setNames(c("#FBAA3E", "#2C83BE", "#3EB6BD", "#A3D5B3", "#CD71A8"),
                         nm = c("day0", "day7", "day14", "day21", "day28"))
colPals$time_light <- setNames(c("#FDD6A1", "#A2CDE9", "#AFE2E5", "#DDF0E3", "#E8BDD6"),
                               nm = c("day0", "day7", "day14", "day21", "day28"))
colPals$time_dark <- setNames(c("#D87E04", "#174564", "#1F5C60", "#49A065", "#AA3C7E"),
                              nm = c("day0", "day7", "day14", "day21", "day28"))
colPals$inferno <- c("#000004", "#420A68", "#932667", "#DD513A", "#FCA50A", "#FCFFA4")
```

# Load data

## Background annotation

```
ann_data <- read.table(
  file = 'data/resources/gene_annotation_ensembl_v104.txt',
  stringsAsFactors = FALSE,
  sep = "\t",
  header = TRUE,
  fill = FALSE,
  quote = "") %>%
  dplyr::rename(Geneid = ensembl_gene_id)
```

## RNAseq expression data

> From a previous pre-processing we saw that volunteers 9 and 10 have opposite fold change profiles when compared to other volunteers. This includes genes that are highly expressed in blood monocytes (e.g., CDKN1A, NR4A1, NR4A2, MYADM, IRS2 and CD83). Here we perform a pre-processing where these volunteers have been removed according to our exclusion criteria.

```
RNAseq <- vector("list")

RNAseq[["unfilt"]][["rawdata"]] <- read.table(file = 'data/rnaseq/rnaseq_count_mtx.tsv',
                                              stringsAsFactors = FALSE,
                                              sep = "\t",
                                              header = TRUE)

# Make duplicated gene names unique
RNAseq[["unfilt"]][["rawdata"]] <- RNAseq[["unfilt"]][["rawdata"]] %>%
  mutate(GeneSymbol = uniquify(plyr::mapvalues(.$Geneid,
                                               from = ann_data$Geneid,
                                               to = ann_data$external_gene_name,
                                               warn_missing = F), sep = '_'))

RNAseq[["unfilt"]][["annotation"]] <- RNAseq[["unfilt"]][["rawdata"]] %>%
  select(Geneid, GeneSymbol) %>%
  inner_join(ann_data, by = "Geneid") %>%
```

```
  select(-c(external_gene_name))

RNAseq[["unfilt"]][["design"]] <- read.table(file = 'data/RNAseq/rnaseq_design_mtx.tsv',
                                             stringsAsFactors = FALSE,
                                             sep = "\t",
                                             header = TRUE) %>%
  mutate(sample = factor(sample, levels = sample),
         batch = factor(batch, levels = unique(batch)),
         volunteer = factor(volunteer, levels = unique(volunteer)),
         time = factor(time, levels = unique(time)))

RNAseq[["unfilt"]][["counts"]] <- RNAseq[["unfilt"]][["rawdata"]] %>%
  select(-c(1:3)) %>%
  column_to_rownames("GeneSymbol")
```

# Pre-processing

## Filtering zero count genes

```
paste("Raw feature count:", nrow(RNAseq$unfilt$counts))

## [1] "Raw feature count: 60649"

tokeep <- rowSums(RNAseq$unfilt$counts) > 0
paste("Non-zero feature count:", sum(tokeep))

## [1] "Non-zero feature count: 42112"

RNAseq$unfilt$rawdata <- RNAseq$unfilt$rawdata[tokeep,]
RNAseq$unfilt$annotation <- RNAseq$unfilt$annotation[tokeep,]
RNAseq$unfilt$counts <- RNAseq$unfilt$counts[tokeep,]
rm(tokeep)
```

## Library sizes and gene expression distributions

```
df <- RNAseq$unfilt$design
df$lib.size <- colSums(RNAseq$unfilt$counts)

ggplot(df, aes(x=sample, y=lib.size/1e6, fill=volunteer)) +
  geom_bar(stat = "identity", width = 0.8) +
  xlab("") +
  ylab("Library size (million reads)") +
  scale_x_discrete(expand =expansion(mult = c(.02, .02))) +
  scale_y_continuous(expand =expansion(mult = c(.02, .05))) +
  theme_custom(
    axis.text.x.bottom = element_text(angle = 90, hjust = 1, vjust = 0.3)
  )
```

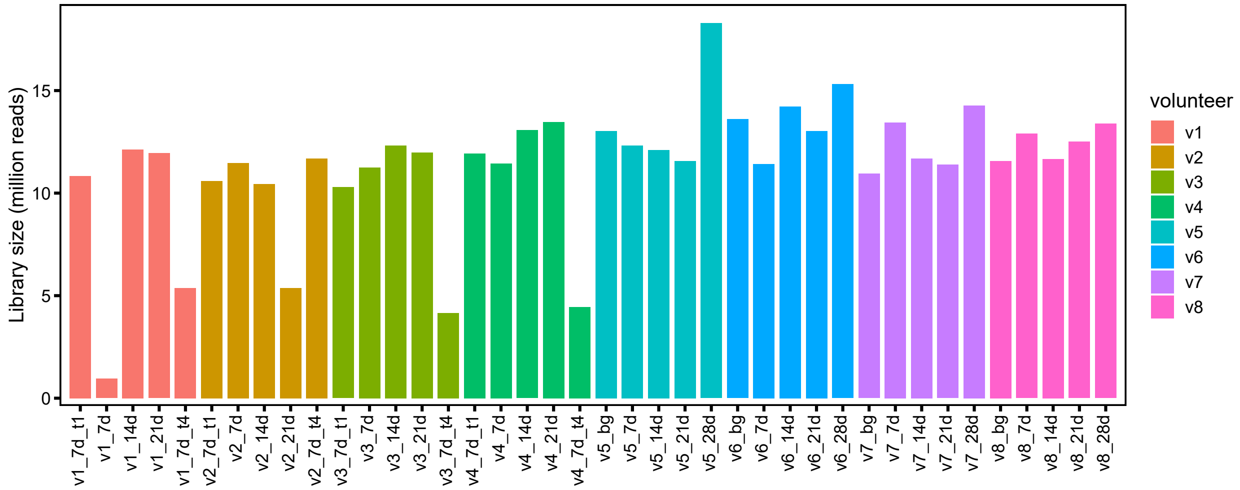


```
df <- RNAseq$unfilt$counts %>%
  rownames_to_column(var = "geneID") %>%
```

```
  pivot_longer(cols = c(2:length(.)),
               names_to = "sample") %>%
  dplyr::rename(counts = value)

df$volunteer <- rep(RNAseq$unfilt$design$volunteer, dim(RNAseq$unfilt$counts)[1])
df$time <- rep(RNAseq$unfilt$design$time, dim(RNAseq$unfilt$counts)[1])
df$sample <- factor(df$sample, levels = names(RNAseq$unfilt$counts))

ggplot(df, aes(x=sample, y=log2(counts+1), fill=volunteer)) +
  geom_violin(scale = "area") +
  xlab("") +
  ylab(expression(Log[2](counts+1))) +
  scale_x_discrete(expand =expansion(mult = c(.02, .02))) +
  scale_y_continuous(expand =expansion(mult = c(.02, .05))) +
  theme_custom(
    axis.text.x.bottom = element_text(angle = 90, hjust = 1, vjust = 0.3),
    legend.position = "top"
  )
```

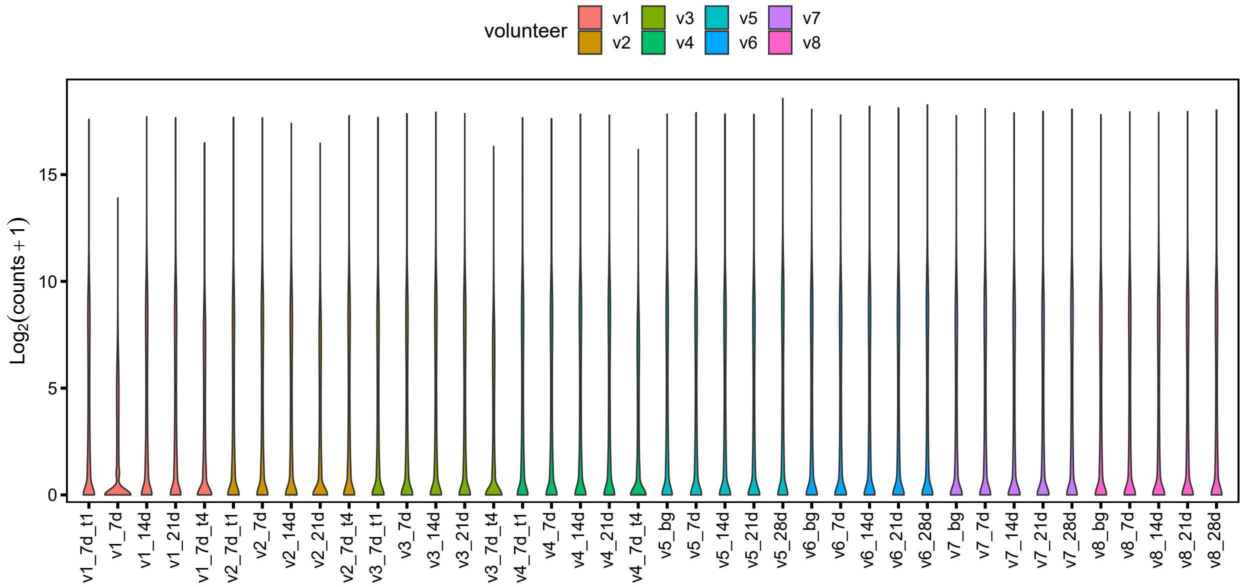


# Filtering low abundance genes

```
# Calculate CPM
RNAseq[["unfilt"]][["cpm"]] <- cpm.normalize(RNAseq$unfilt$counts)

abovethresh <- RNAseq$unfilt$cpm > 1

df <- data.frame(samples = factor(seq(0,ncol(RNAseq$unfilt$cpm),1),
                                  levels = rev(seq(0,ncol(RNAseq$unfilt$cpm),1))),
                 genes = c(table(rowSums(abovethresh)))) %>%
  mutate(cumulative = rev(cumsum(rev(genes)))) %>%
  mutate(remaining = sum(genes)-cumulative) %>%
  pivot_longer(cols = c("cumulative","remaining"),
               names_to = "group") %>%
  mutate(group = factor(group, levels = c("remaining", "cumulative")))

ggplot(data=df, aes(x=samples, y=value, fill=group)) +
  geom_bar(color="black", size=0.5, width=0.8, position="stack", stat="identity") +
  geom_hline(yintercept = unlist(df[df$samples == "20" & df$group == "cumulative", "value"]),
             linetype="solid", size=1, color="#EF2126") +
  geom_vline(xintercept = "20", linetype="solid", size=1, color="#EF2126") +
  xlab("# of samples present (CPM>1)") +
  ylab("# of genes") +
  scale_x_discrete(expand =expansion(mult = c(.02, .02))) +
  scale_y_continuous(expand =expansion(mult = c(.02, .00)),
                     limits = c(0,45000), breaks = seq(0,45000,15000)) +
  scale_fill_manual(values = c("grey80", "grey20")) +
  theme_custom(base_size = 8)
```

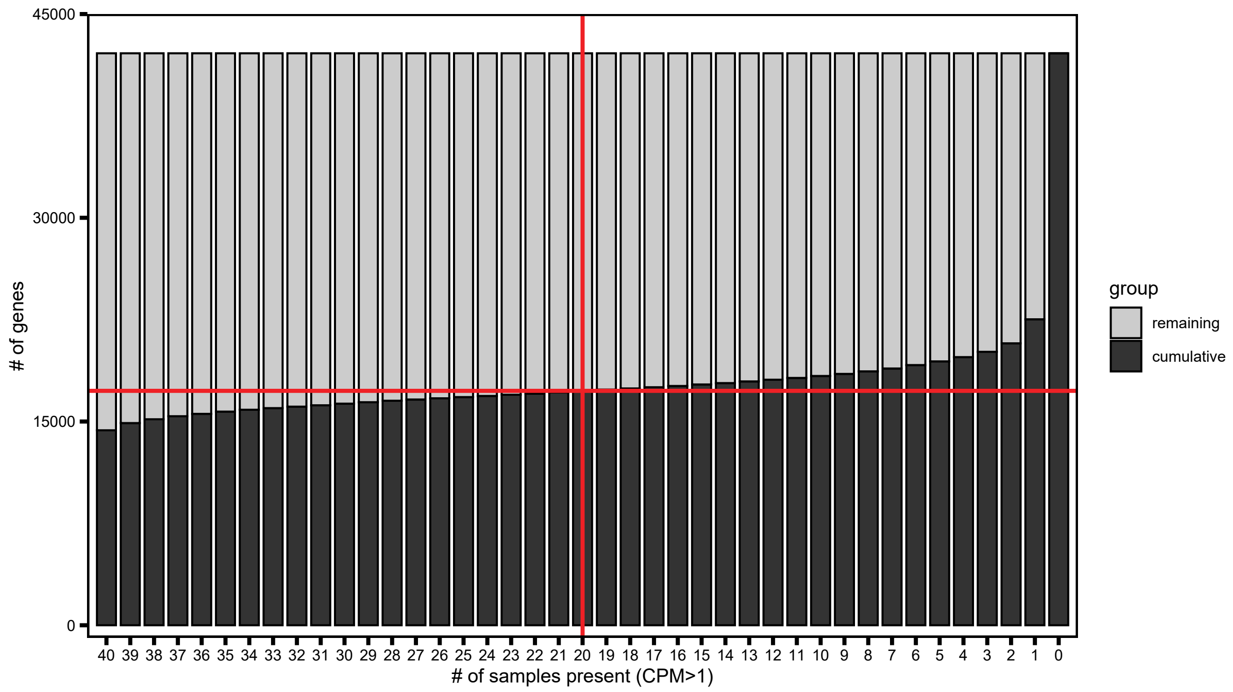


```
tokeep <- rowSums(abovethresh) >= 20
paste("Pre-filtering gene count:", length(tokeep))
```

```
## [1] "Pre-filtering gene count: 42112"
```

```
paste("Genes below abundance threshold:", length(tokeep)-sum(tokeep))
```

```
## [1] "Genes below abundance threshold: 24853"
```

```
paste("Remaining genes:", sum(tokeep))
```

```
## [1] "Remaining genes: 17259"
```

```
# Filter genes
RNAseq[["filt"]][["rawdata"]] <- RNAseq$unfilt$rawdata[tokeep,]
RNAseq[["filt"]][["annotation"]] <- RNAseq$unfilt$annotation[tokeep,]
RNAseq[["filt"]][["design"]] <- RNAseq$unfilt$design
RNAseq[["filt"]][["counts"]] <- RNAseq$unfilt$counts[tokeep,]

# Normalize
RNAseq[["filt"]][["cpm"]] <- cpm.normalize(RNAseq$filt$counts)
```

```
# Expression distribution post-filtering
df <- RNAseq$filt$counts %>%
  rownames_to_column(var = "geneID") %>%
  pivot_longer(cols = c(2:length(.)),
               names_to = "sample") %>%
  dplyr::rename(counts = value)

df$volunteer <- rep(RNAseq$filt$design$volunteer, dim(RNAseq$filt$counts)[1])
df$time <- rep(RNAseq$filt$design$time, dim(RNAseq$filt$counts)[1])
df$sample <- factor(df$sample, levels = names(RNAseq$filt$counts))

ggplot(df, aes(x=sample, y=log2(counts+1), fill=volunteer)) +
  geom_violin(scale = "area") +
  xlab("") +
  ylab(expression(Log[2](counts+1))) +
  scale_x_discrete(expand =expansion(mult = c(.02, .02))) +
  scale_y_continuous(expand =expansion(mult = c(.02, .05))) +
  theme_custom(
    axis.text.x.bottom = element_text(angle = 90, hjust = 1, vjust = 0.3),
    legend.position = "top"
  )
```

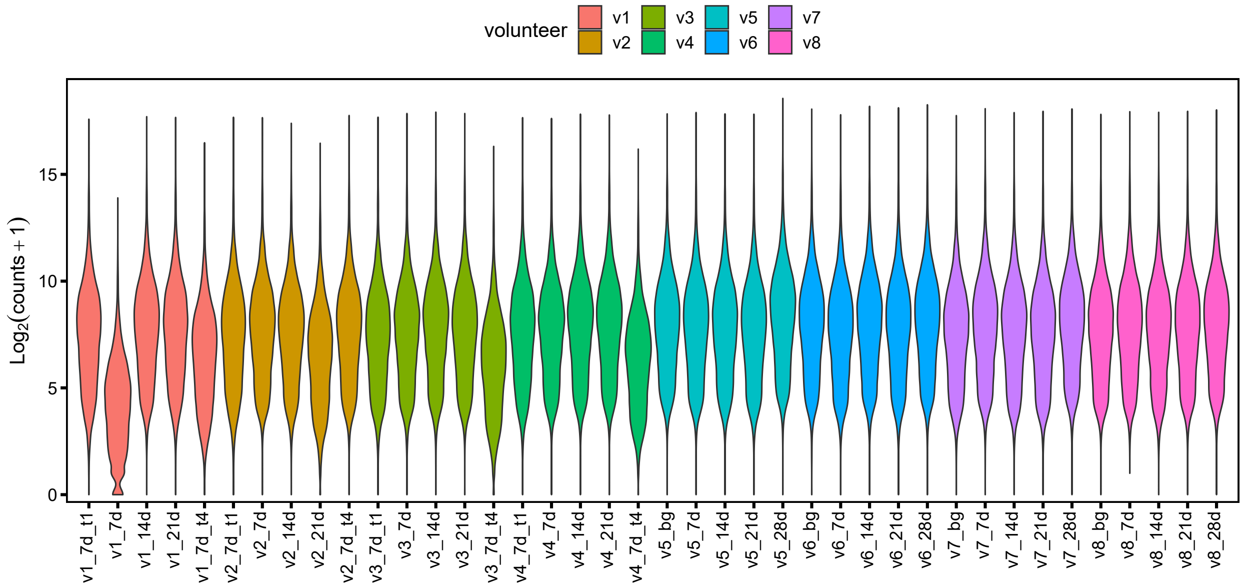


```r
# Normalized expression distribution post-filtering
df <- RNAseq$filt$counts %>%
  rownames_to_column(var = "geneID") %>%
  pivot_longer(cols = c(2:length(.)),
               names_to = "sample") %>%
  dplyr::rename(counts = value)

df$volunteer <- rep(RNAseq$filt$design$volunteer, dim(RNAseq$filt$counts)[1])
df$time <- rep(RNAseq$filt$design$time, dim(RNAseq$filt$counts)[1])
df$sample <- factor(df$sample, levels = names(RNAseq$filt$counts))

df$cpm <- RNAseq$filt$cpm %>%
  pivot_longer(cols = c(1:length(.)),
               names_to = "sample") %>%
  select(value) %>%
  unlist()

ggplot(df, aes(x=sample, y=log2(cpm+1), fill=volunteer)) +
  geom_violin(scale = "area") +
  xlab("") +
  ylab(expression(Log[2](CPM+1))) +
  scale_x_discrete(expand =expansion(mult = c(.02, .02))) +
  scale_y_continuous(expand =expansion(mult = c(.02, .05))) +
  theme_custom(
    axis.text.x.bottom = element_text(angle = 90, hjust = 1, vjust = 0.3),
    legend.position = "top"
  )
```

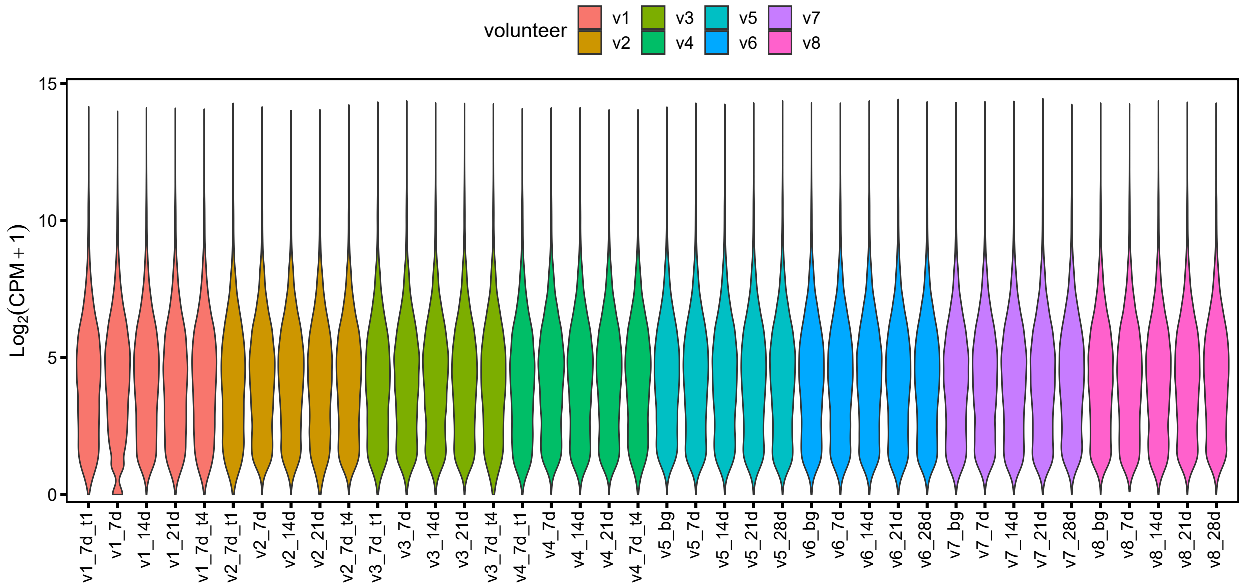


# Transcriptome differences

## Sample correlations

```
df <- cor(log2(RNAseq$filt$cpm+1), method = "spearman") %>%
  as.data.frame() %>%
  rownames_to_column(var = "sample1") %>%
  mutate(across(everything(), as.character)) %>%
  pivot_longer(cols = c(2:length(.)),
               names_to = "sample2") %>%
  dplyr::rename(r = value) %>%
  mutate(sample1 = factor(sample1, levels = names(RNAseq$filt$counts)),
         sample2 = factor(sample2, levels = names(RNAseq$filt$counts)),
         r = as.numeric(r))

ggplot(df, aes(x=sample1, y=sample2, fill= r)) +
  geom_tile() +
  scale_y_discrete(limits=rev) +
  scale_fill_gradientn(colours = rev(colPals$inferno)) +
  theme_custom(
    axis.text.x.bottom = element_text(angle = 90, hjust = 1, vjust = 0.3),
    legend.position = "right",
    legend.justification = "top"
  )
```

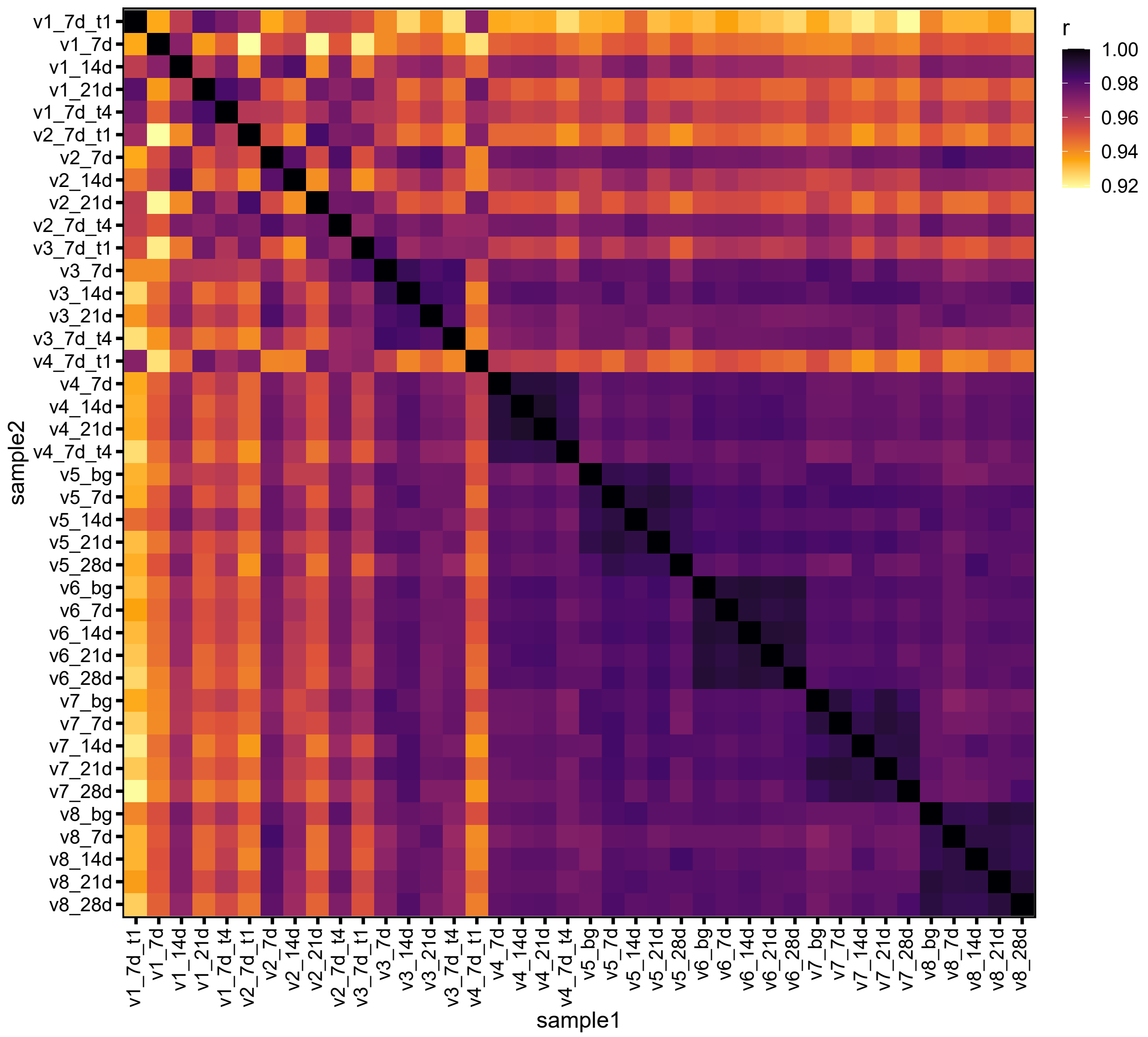


## DESeq2 analysis

```
# DESeq2 pipeline
dsData <- DESeqDataSetFromMatrix(countData = RNAseq$filt$counts,
                                 colData = RNAseq$filt$design,
                                 design = ~volunteer + time)
dsData <- estimateSizeFactors(dsData)
dsData <- DESeq(dsData, test = "LRT", reduced = ~volunteer)

RNAseq$filt[["DESeq_norm"]] <- counts(dsData, normalized=TRUE) %>% as.data.frame()
RNAseq$filt[["DESeq_vst"]] <- assay(vst(dsData, blind=FALSE)) %>% as.data.frame()
RNAseq$filt[["DESeq_rlog"]] <- assay(rlog(dsData, blind=FALSE)) %>% as.data.frame()

DESeq2_DEGs <- list(
  GLMtime = results(dsData, test = "LRT", independentFiltering = T),
  day7Vsday0 = results(dsData, contrast=c("time", "day7", "day0"), test = "Wald", independentFiltering = T),
  day14Vsday0 = results(dsData, contrast=c("time", "day14", "day0"), test = "Wald", independentFiltering = T),
  day21Vsday0 = results(dsData, contrast=c("time", "day21", "day0"), test = "Wald", independentFiltering = T),
  day28Vsday0 = results(dsData, contrast=c("time", "day28", "day0"), test = "Wald", independentFiltering = T)
)
DESeq2_DEGs <- lapply(DESeq2_DEGs, as.data.frame)
DESeq2_DEGs$GLMtime <- DESeq2_DEGs$GLMtime %>%
  add_column(logFC_day7Vsday0 = DESeq2_DEGs$day7Vsday0$log2FoldChange, .before = "log2FoldChange") %>%
  add_column(logFC_day14Vsday0 = DESeq2_DEGs$day14Vsday0$log2FoldChange, .before = "log2FoldChange") %>%
  add_column(logFC_day21Vsday0 = DESeq2_DEGs$day21Vsday0$log2FoldChange, .before = "log2FoldChange") %>%
```

```
  add_column(logFC_day28Vsday0 = DESeq2_DEGs$day28Vsday0$log2FoldChange, .before = "log2FoldChange") %>%
  select(-log2FoldChange)
DESeq2_DEGs <- lapply(DESeq2_DEGs, function(x) mutate(x, padj=ifelse(is.na(padj), 1, padj)))
DESeq2_DEGs <- lapply(DESeq2_DEGs, function(x) arrange(x, padj))
DESeq2_DEGs <- lapply(DESeq2_DEGs, rownames_to_column, var = "GeneSymbol")
DESeq2_DEGs <- lapply(DESeq2_DEGs, inner_join, y = RNAseq$filt$annotation, by = "GeneSymbol")

DESeq2_DEGs_filt <- lapply(DESeq2_DEGs, function(x) x %>% filter(padj<0.01))
```

# Batch correction

```
# Remove batch effects from data
design_mtx <- model.matrix(~time, data = RNAseq$filt$design)
RNAseq[["filt"]][["DESeq_vst_nobatch"]] <- removeBatchEffect(
  RNAseq$filt$DESeq_vst,
  batch = dsData$volunteer,
  design = design_mtx) %>% as.data.frame()
RNAseq[["filt"]][["DESeq_rlog_nobatch"]] <- removeBatchEffect(
  RNAseq$filt$DESeq_rlog,
  batch = dsData$volunteer,
  design = design_mtx) %>% as.data.frame()

# Plot sample correlations
df <- cor(RNAseq$filt$DESeq_vst, method = "spearman") %>%
  as.data.frame() %>%
  rownames_to_column(var = "sample1") %>%
  mutate(across(everything(), as.character)) %>%
  pivot_longer(cols = c(2:length(.)),
               names_to = "sample2") %>%
  dplyr::rename(r = value) %>%
  mutate(sample1 = factor(sample1, levels = names(RNAseq$filt$counts)),
         sample2 = factor(sample2, levels = names(RNAseq$filt$counts)),
         r = as.numeric(r))

p1 <- ggplot(df, aes(x=sample1, y=sample2, fill= r)) +
  geom_tile() +
  scale_x_discrete(labels=paste(RNAseq$filt$design$volunteer,
                                RNAseq$filt$design$time,
                                sep = '_')) +
  scale_y_discrete(limits=rev, labels=rev(paste(RNAseq$filt$design$volunteer,
                                                RNAseq$filt$design$time,
                                                sep = '_'))) +
  scale_fill_gradientn(colours = rev(colPals$inferno)) +
  xlab('') +
  ylab('') +
  ggtitle('Normalized') +
  theme_custom(
    base_size = 6,
    axis.text.x.bottom = element_text(angle = 90, hjust = 1, vjust = 0.3),
    legend.position = "none",
    plot.title = element_text(size=14, face='bold', hjust=0.5)
  )

df2 <- cor(RNAseq$filt$DESeq_vst_nobatch, method = "spearman") %>%
  as.data.frame() %>%
  rownames_to_column(var = "sample1") %>%
  mutate(across(everything(), as.character)) %>%
  pivot_longer(cols = c(2:length(.)),
               names_to = "sample2") %>%
  dplyr::rename(r = value) %>%
  mutate(sample1 = factor(sample1, levels = names(RNAseq$filt$counts)),
         sample2 = factor(sample2, levels = names(RNAseq$filt$counts)),
         r = as.numeric(r))

p2 <- ggplot(df2, aes(x=sample1, y=sample2, fill= r)) +
  geom_tile() +
  scale_x_discrete(labels=paste(RNAseq$filt$design$volunteer,
                                RNAseq$filt$design$time,
                                sep = '_')) +
  scale_y_discrete(limits=rev, labels=rev(paste(RNAseq$filt$design$volunteer,
                                                RNAseq$filt$design$time,
                                                sep = '_'))) +
  scale_fill_gradientn(colours = rev(colPals$inferno)) +
  xlab('') +
  ylab('') +
  ggtitle('Normalized & batch-corrected') +
  theme_custom(
    base_size = 6,
```

```
    axis.text.x.bottom = element_text(angle = 90, hjust = 1, vjust = 0.3),
    legend.position = "right",
    legend.justification = "top",
    plot.title = element_text(size=14, face='bold', hjust=0.5)
  )

p1 + p2
```

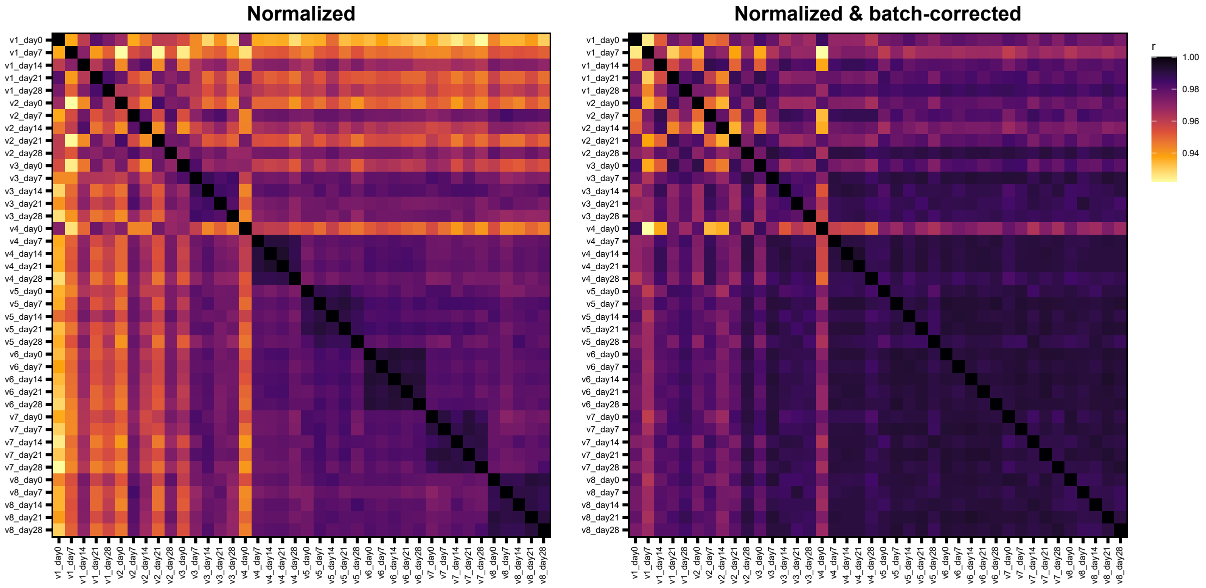


# PCA plots

```
# PCA with mean summarisation
pca <- doPCA(RNAseq$filt$DESeq_vst_nobatch)

df <- pca$pcs %>%
  cbind(RNAseq$filt$design)

df2 <- df %>%
  group_by(time) %>%
  summarize(PC1 = mean(PC1),
            PC2 = mean(PC2),
            PC3 = mean(PC3),) %>%
  dplyr::rename(time2 = time)

ggplot() +
  geom_point(data = df, aes(x=PC1, y=PC2, color=time), shape=16, size=3, stroke=0, alpha=0.4) +
  geom_point(data = df2, aes(x=PC1, y=PC2, fill=time2), color="black", shape=23, size=6, stroke=1.5, alpha=1) +
  ggrepel::geom_label_repel() +
  xlab(paste("PC1 (", round(pca$percentVar[1],0), "%)", sep = "")) +
  ylab(paste("PC2 (", round(pca$percentVar[2],0), "%)", sep = "")) +
  scale_color_manual(values = colPals$time) +
  scale_fill_manual(values = colPals$time, name='Time') +
  guides(color = F, fill = guide_legend(override.aes = list(size=4))) +
  ggtitle('Volunteers 9 & 10 excluded') +
  theme_bw(base_size = 16) +
  theme(
    legend.position = 'right',
    legend.justification = 'top',
    plot.title = element_text(size=16, face='bold', hjust=0.5),
    axis.title = element_text(size=16, face='bold'),
    panel.grid.major = element_blank(),
    panel.grid.minor = element_blank(),
    panel.border = element_rect(color = "black", fill = NA, size = 2),
    axis.ticks = element_line(color = "black", size = 1.25)
  )
```

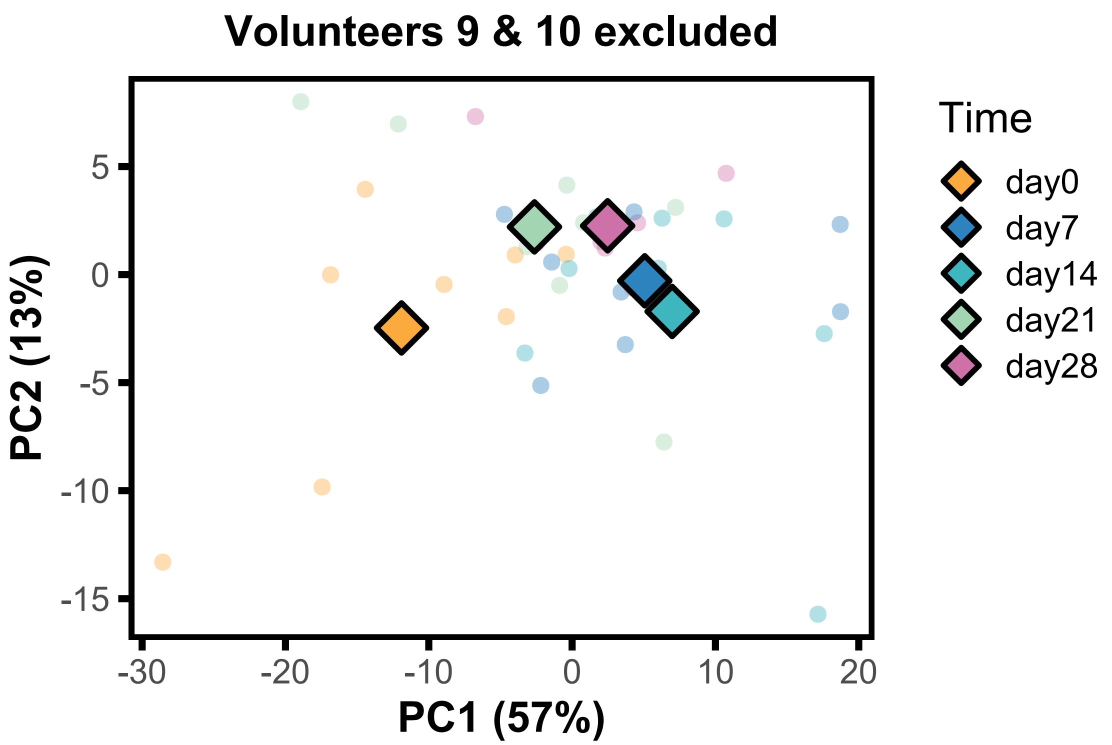


```
ggsave("plots/figS2_pca_volunteers_9&10_excl.pdf", width = 6, height = 4, units = "in", dpi = 300, device = cairo_pdf)
```

```
pca <- doPCA(RNAseq$filt$DESeq_vst_nobatch)

df <- pca$pcs %>%
  cbind(RNAseq$filt$design)

df2 <- df %>%
  group_by(time) %>%
  summarize(PC1 = mean(PC1),
            PC2 = mean(PC2),
            PC3 = mean(PC3),) %>%
  dplyr::rename(time2 = time)

ggplot() +
  geom_point(data = df, aes(x=PC1, y=PC2, color=time), shape=16, size=4, stroke=0, alpha=0.4) +
  geom_point(data = df2, aes(x=PC1, y=PC2, color=time2, fill=time2), shape=21, size=7.5, stroke=2, alpha=1) +
  ggrepel::geom_label_repel() +
  theme_custom(base_size = 20) +
  xlab(paste("PC1 (", round(pca$percentVar[1],0), "%)", sep = "")) +
  ylab(paste("PC2 (", round(pca$percentVar[2],0), "%)", sep = "")) +
  scale_color_manual(values = colPals$time_dark) +
  scale_fill_manual(values = colPals$time) +
  scale_x_continuous(limits = c(-30,20), breaks = seq(-30,15,15)) +
  scale_y_continuous(limits = c(-20,10),breaks = seq(-20,10,10)) +
  theme(panel.grid.major = element_line(color = "grey80", linetype = "solid", size = 1.25),
        panel.grid.minor = element_line(color = "transparent", linetype = "solid"),
        panel.border = element_rect(color = "black", fill = NA, size = 2),
        axis.ticks = element_line(color = "black", size = 1.25),
        legend.position = "none")
```

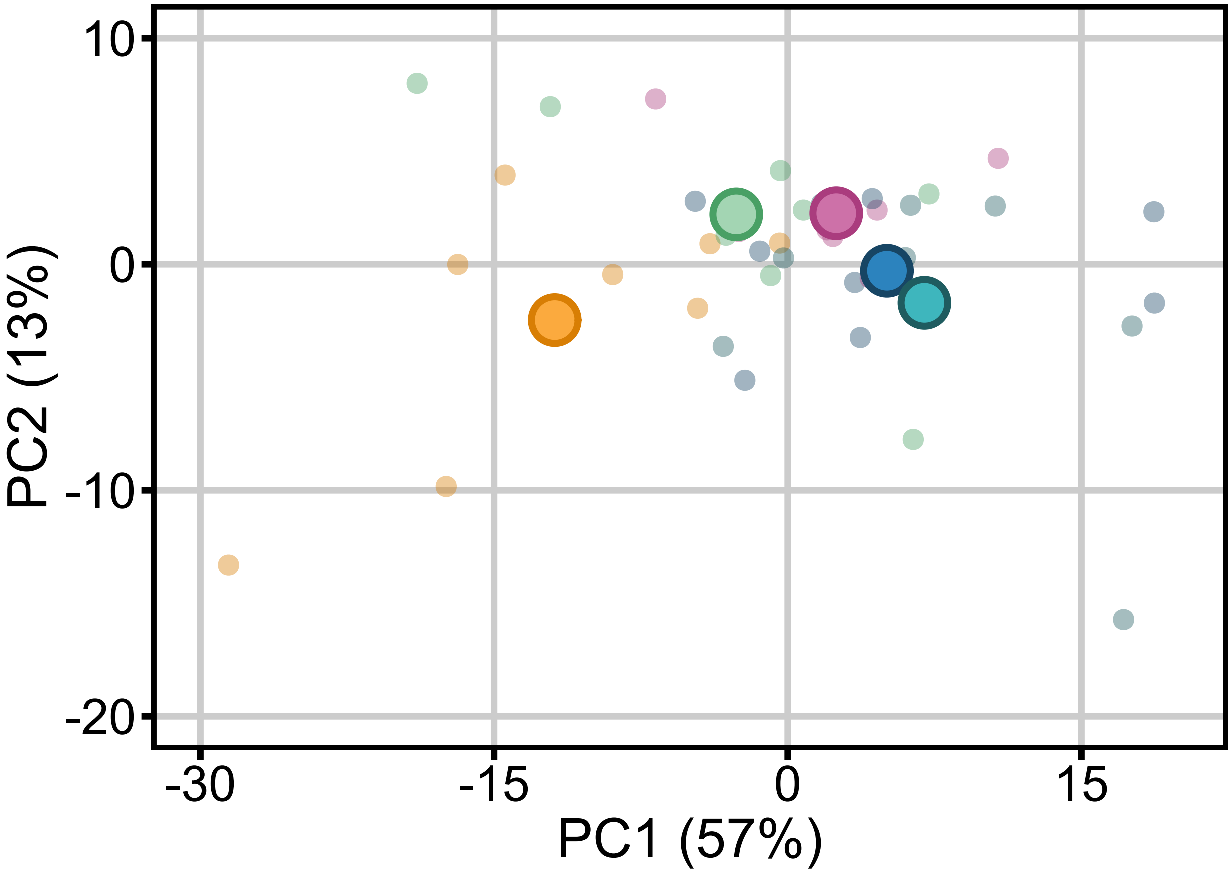


```
ggsave(filename = "plots/fig1D_pca_volunteers_9&10_excl.pdf", width = 7, height = 5, units = "in", dpi = 300, device = cairo_pdf)
```

```
df2 <- df %>%
  group_by(time) %>%
  select(time, PC1) %>%
  summarize_each(dplyr::funs(mean, sd, se=sd(.)/sqrt(n())), PC1) %>%
  dplyr::rename(time2 = time)

ggplot() +
  geom_point(data = df, aes(x=time, y=PC1, color=time), shape=16, size=4, stroke=0, alpha=0.5) +
  geom_errorbar(data=df2, aes(x=time2, y=mean, ymin=mean-se*1.96, ymax=mean+se*1.96), width=.2, lwd=1.5) +
  geom_point(data = df2, aes(x=time2, y=mean, fill=time2), color="black", shape=21, size=5, stroke=2, alpha=1) +
  scale_x_discrete(limits = rev(levels(df$time))) +
  scale_y_continuous(limits = c(-30,20), breaks = seq(-30,15,15)) +
  coord_flip() +
  theme_custom(base_size = 20) +
  ylab(paste("PC1 (", round(pca$percentVar[1],0), "%)", sep = "")) +
  scale_color_manual(values = colPals$time) +
  scale_fill_manual(values = colPals$time) +
  theme(panel.grid.major.x = element_line(color = "grey80", linetype = "solid", size = 1.25),
        panel.grid.minor = element_line(color = "transparent", linetype = "solid"),
        panel.border = element_rect(color = "black", fill = NA, size = 2),
        axis.ticks = element_line(color = "black", size = 1.25),legend.position = "none")
```

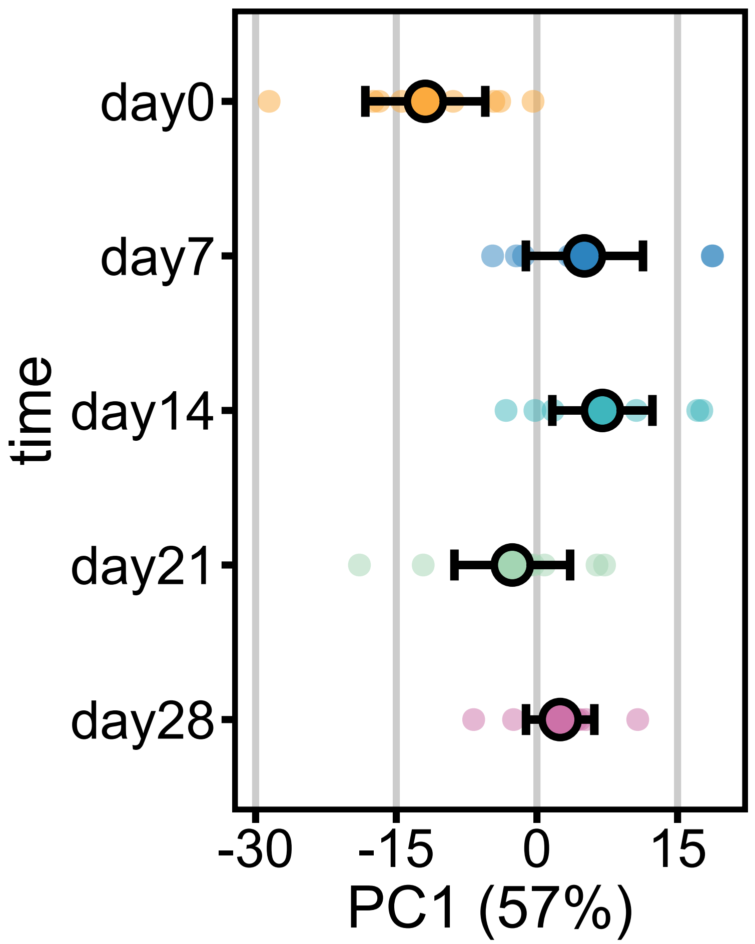


```
ggsave(filename = "plots/fig1E_pca_volunteers_9&10_excl_PC1.pdf", width = 4, height = 5, units = "in", dpi = 300, device = cairo_pdf)
```

```
df2 <- as.data.frame(pca$rotation) %>%
  rownames_to_column(var = "GeneSymbol") %>%
  select(GeneSymbol, PC1) %>%
  mutate(sign = sign(PC1)) %>%
  mutate(PC1_abs = abs(PC1)) %>%
  arrange(desc(PC1_abs))

df2$GeneSymbol[1:10]
```

```
##  [1] "SIK1B"   "NR4A2"   "SIK1"    "TNFAIP3" "RGS1"    "TENT5C"  "CSRNP1"
##  [8] "DUSP2"   "PER1"    "SOCS3"
```

```
df2 <- df %>%
  group_by(time) %>%
  select(time, PC2) %>%
  summarize_each(dplyr::funs(mean, sd, se=sd(.)/sqrt(n())), PC2) %>%
  dplyr::rename(time2 = time)

ggplot() +
  geom_point(data = df, aes(x=time, y=PC2, color=time), shape=16, size=4, stroke=0, alpha=0.5) +
  geom_errorbar(data=df2, aes(x=time2, y=mean, ymin=mean-se*1.96, ymax=mean+se*1.96), width=.2, lwd=1.5) +
  geom_point(data = df2, aes(x=time2, y=mean, fill=time2), color="black", shape=21, size=5, stroke=2, alpha=1) +
  scale_x_discrete(limits = rev(levels(df$time))) +
  scale_y_continuous(limits = c(-20,10), breaks = seq(-20,10,10)) +
  coord_flip() +
  theme_custom(base_size = 20) +
  ylab(paste("PC2 (", round(pca$percentVar[2],0), "%)", sep = "")) +
  scale_color_manual(values = colPals$time) +
  scale_fill_manual(values = colPals$time) +
  theme(panel.grid.major.x = element_line(color = "grey80", linetype = "solid", size = 1.25),
        panel.grid.minor = element_line(color = "transparent", linetype = "solid"),
        panel.border = element_rect(color = "black", fill = NA, size = 2),
        axis.ticks = element_line(color = "black", size = 1.25),legend.position = "none")
```

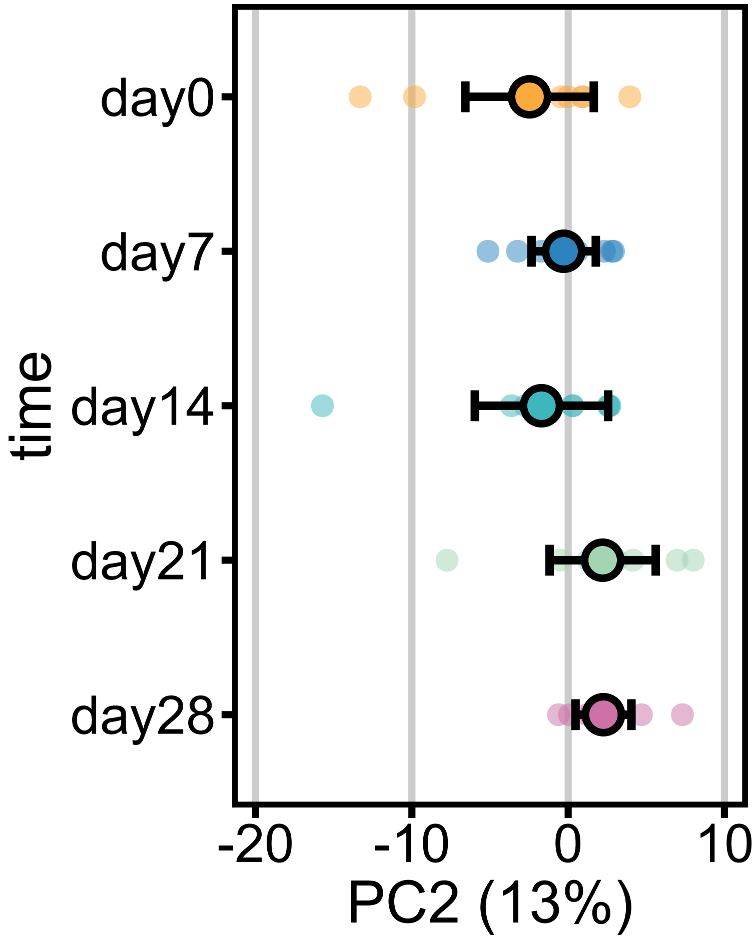


```
df2 <- as.data.frame(pca$rotation) %>%
  rownames_to_column(var = "GeneSymbol") %>%
  select(GeneSymbol, PC2) %>%
  mutate(sign = sign(PC2)) %>%
  mutate(PC2_abs = abs(PC2)) %>%
  arrange(desc(PC2_abs))

df2$GeneSymbol[1:11]
```

```
##  [1] "LYZ"             "ENSG00000257764" "FCN1"            "S100A9"
##  [5] "S100A8"          "VCAN"            "CSF3R"           "SERPINA1"
##  [9] "TNFAIP2"         "MPEG1"           "IFI30"
```

# Exports

```
saveRDS(RNAseq, file= "data/rnaseq/rnaseq_volunteers_9&10_excl.rds")
saveRDS(DESeq2_DEGs, file= "data/rnaseq/DESeq2_DEGs_unfilt_volunteers_9&10_excl.rds")
saveRDS(DESeq2_DEGs_filt, file= "data/rnaseq/DESeq2_DEGs_filt_volunteers_9&10_excl.rds")

openxlsx::write.xlsx(DESeq2_DEGs_filt[2:5], file = "tables/dataS3_DEGs_filtered.xlsx", rowNames=F, overwrite=T)
```

# SessionInfo

```
sessionInfo()
```

```
## R version 4.2.1 (2022-06-23 ucrt)
## Platform: x86_64-w64-mingw32/x64 (64-bit)
## Running under: Windows 10 x64 (build 19044)
##
```

```
## Matrix products: default
##
## locale:
## [1] LC_COLLATE=English_United States.utf8
## [2] LC_CTYPE=English_United States.utf8
## [3] LC_MONETARY=English_United States.utf8
## [4] LC_NUMERIC=C
## [5] LC_TIME=English_United States.utf8
##
## attached base packages:
## [1] stats4    stats     graphics  grDevices utils     datasets  methods
## [8] base
##
## other attached packages:
##  [1] RColorBrewer_1.1-3          variancePartition_1.28.9
##  [3] BiocParallel_1.32.6         limma_3.54.2
##  [5] DESeq2_1.38.3               SummarizedExperiment_1.28.0
##  [7] Biobase_2.58.0              MatrixGenerics_1.10.0
##  [9] matrixStats_0.63.0          GenomicRanges_1.50.2
## [11] GenomeInfoDb_1.34.9         IRanges_2.32.0
## [13] S4Vectors_0.36.2            BiocGenerics_0.44.0
## [15] patchwork_1.1.2             magrittr_2.0.3
## [17] forcats_1.0.0               stringr_1.5.0
## [19] dplyr_1.1.1                 purrr_1.0.1
## [21] readr_2.1.4                 tidyr_1.3.0
## [23] tibble_3.2.1                ggplot2_3.4.2
## [25] tidyverse_1.3.2
##
## loaded via a namespace (and not attached):
##   [1] googledrive_2.1.0       minqa_1.2.5             colorspace_2.1-0
##   [4] XVector_0.38.0          fs_1.6.1                rstudioapi_0.14
##   [7] farver_2.1.1            ggrepel_0.9.3           bit64_4.0.5
##  [10] mvtnorm_1.1-3           AnnotationDbi_1.60.2    fansi_1.0.4
##  [13] lubridate_1.9.2         xml2_1.3.3              codetools_0.2-18
##  [16] splines_4.2.1           doParallel_1.0.17       cachem_1.0.7
##  [19] geneplotter_1.76.0      knitr_1.42              jsonlite_1.8.4
##  [22] nloptr_2.0.3            pbkrtest_0.5.2          RhpcBLASctl_0.23-42
##  [25] broom_1.0.4             annotate_1.76.0         dbplyr_2.3.2
##  [28] png_0.1-8               aod_1.3.2               compiler_4.2.1
##  [31] httr_1.4.5              backports_1.4.1         Matrix_1.5-3
##  [34] fastmap_1.1.1           gargle_1.3.0            cli_3.6.1
##  [37] prettyunits_1.1.1       htmltools_0.5.5         tools_4.2.1
##  [40] gtable_0.3.3            glue_1.6.2              GenomeInfoDbData_1.2.9
##  [43] reshape2_1.4.4          clusterGeneration_1.3.7 Rcpp_1.0.10
##  [46] cellranger_1.1.0        vctrs_0.6.1             Biostrings_2.66.0
##  [49] nlme_3.1-157            iterators_1.0.14        remaCor_0.0.11
##  [52] xfun_0.38               rbibutils_2.2.13        openxlsx_4.2.5.1
##  [55] lme4_1.1-32             rvest_1.0.3             timechange_0.2.0
##  [58] lifecycle_1.0.3         gtools_3.9.4            XML_3.99-0.14
##  [61] googlesheets4_1.1.0     zlibbioc_1.44.0         MASS_7.3-57
##  [64] scales_1.2.1            hms_1.1.3               parallel_4.2.1
##  [67] yaml_2.3.7              memoise_2.0.1           stringi_1.7.12
##  [70] RSQLite_2.3.0           highr_0.10              foreach_1.5.2
##  [73] caTools_1.18.2          zip_2.2.2               boot_1.3-28
##  [76] Rdpack_2.4              rlang_1.1.0             pkgconfig_2.0.3
##  [79] bitops_1.0-7            evaluate_0.20           lattice_0.20-45
##  [82] labeling_0.4.2          bit_4.0.5               tidyselect_1.2.0
##  [85] plyr_1.8.8              R6_2.5.1                gplots_3.1.3
##  [88] generics_0.1.3          RUnit_0.4.32            DelayedArray_0.24.0
##  [91] DBI_1.1.3               pillar_1.9.0            haven_2.5.2
##  [94] withr_2.5.0             KEGGREST_1.38.0         RCurl_1.98-1.12
##  [97] modelr_0.1.11           crayon_1.5.2            KernSmooth_2.23-20
## [100] utf8_1.2.3              tzdb_0.3.0              rmarkdown_2.21
## [103] progress_1.2.2          locfit_1.5-9.7          grid_4.2.1
## [106] readxl_1.4.2            blob_1.2.4              reprex_2.0.2
## [109] digest_0.6.31           xtable_1.8-4            munsell_0.5.0
```

# Step 2.1: Clustering of gene expression profiles

Carlos Gallardo & Christian Oertlin

17 April, 2023

```
# Import libraries and helper functions
source("code/helper_functions.R")
library(tidyverse)
library(magrittr)
library(patchwork)
library(RColorBrewer)
library(vegan)
library(cluster)
library(ComplexHeatmap)

# Colors
colPals <- vector(mode = "list")
colPals$time <- setNames(c("#FBAA3E", "#2C83BE", "#3EB6BD", "#A3D5B3", "#CD71A8"),
                         nm = c("day0", "day7", "day14", "day21", "day28"))
colPals$time_light <- setNames(c("#FDD6A1", "#A2CDE9", "#AFE2E5", "#DDF0E3", "#E8BDD6"),
                               nm = c("day0", "day7", "day14", "day21", "day28"))
colPals$time_dark <- setNames(c("#D87E04", "#174564", "#1F5C60", "#49A065", "#AA3C7E"),
                              nm = c("day0", "day7", "day14", "day21", "day28"))
colPals$inferno <- c("#000004", "#420A68", "#932667", "#DD513A", "#FCA50A", "#FCFFA4")
colPals$blood_cells <- setNames(c("#E54D34","#77A2D5","#B58B80"),
                                nm = c("granulocytes", "lymphocytes", "monocytes"))
colPals$cell_types <- setNames(c("#83D1F6","#FBAA3E","#FCCA7C","#B58B80","#E54D34",
                                 "#B3177E","#9A509F","#77A2D5","#CAC1DD","#36B449","#C1C1C1"),
                               nm = c("B cell", "Macrophage M1", "Macrophage M2",
                                      "Monocyte", "Neutrophil", "NK cell",
                                      "T cell CD4+ (non-regulatory)", "T cell CD8+",
                                      "T cell regulatory (Tregs)", "Myeloid dendritic cell",
                                      "uncharacterized cell"))
colPals$RdBu <- brewer.pal(11, name = "RdBu")
colPals$biotype <- setNames(c("#395982","#49BED9","#18A38A","#36B449","#826F99",
                              "#9852A5","#FBAA3E","#FCCA7C","#FCFFA4","#C1C1C1"),
                            nm = c("protein_coding", "lncRNA", "miRNA", "snoRNA",
                                   "IG_C_gene", "IG_V_gene", "TR_C_gene",
                                   "TR_J_gene", "TR_V_gene", "other"))
```

# Load data

```
# RNA-seq
RNAseq <- readRDS(file='data/rnaseq/rnaseq_volunteers_9&10_excl.rds')
DESeq2_DEGs <- readRDS(file='data/rnaseq/DESeq2_DEGs_unfilt_volunteers_9&10_excl.rds')
DESeq2_DEGs_filt <- readRDS(file='data/rnaseq/DESeq2_DEGs_filt_volunteers_9&10_excl.rds')
```

# Differentially expressed genes (DEGs)

```
# Count up-/downregulated genes for each comparison (di Vs d0)
df <- data.frame(cond = names(DESeq2_DEGs_filt)[1],
                 val = dim(DESeq2_DEGs_filt$GLMtime)[1],
                 type = 'none')

df2 <- lapply(DESeq2_DEGs_filt[-1], function(x) sum(x$log2FoldChange > 0)) %>%
  unlist() %>%
  as.data.frame() %>%
  dplyr::rename(val = '.') %>%
  rownames_to_column(var = 'cond') %>%
  mutate(type = 'up')

df3 <- lapply(DESeq2_DEGs_filt[-1], function(x) sum(x$log2FoldChange < 0)) %>%
```

```r
  unlist() %>%
  as.data.frame() %>%
  dplyr::rename(val = '.') %>%
  rownames_to_column(var = 'cond') %>%
  mutate(type = 'down')

df <- df %>%
  bind_rows(df2) %>%
  bind_rows(df3) %>%
  mutate(cond = factor(cond, levels = names(DESeq2_DEGs_filt)),
         type = factor(type, levels = c('up', 'down', 'none')))

ggplot(df, aes(x=cond, y=val, fill=type)) +
  geom_bar(position="stack", stat="identity", color="black", size=1, width=0.9) +
  scale_fill_manual(values = colPals$RdBu[c(2,10,6)]) +
  scale_x_discrete(expand = expansion(mult = c(.15, .15))) +
  scale_y_continuous(
    name="# of DEGs",
    expand =expansion(mult = c(.002, .03))) +
  xlab('') +
  theme_bw(base_size = 20) +
  theme(
    axis.title.y.right = element_text(angle = 90),
    axis.text.x.bottom = element_text(angle = 45, hjust = 1, vjust = 1),
    axis.text.y = element_text(vjust = 0.3),
    legend.title = element_blank(),
    legend.justification=c(0,1),
    panel.grid.major.y = element_line(color = "grey80", linetype = "solid", size = 1.25),
    panel.grid.major.x = element_blank(),
    panel.grid.minor = element_blank(),
    panel.border = element_rect(color = "black", fill = NA, size = 2),
    axis.ticks = element_line(color = "black", size = 1.25),
    legend.position = 'right',
    legend.text = element_text(size=12)
  )
```

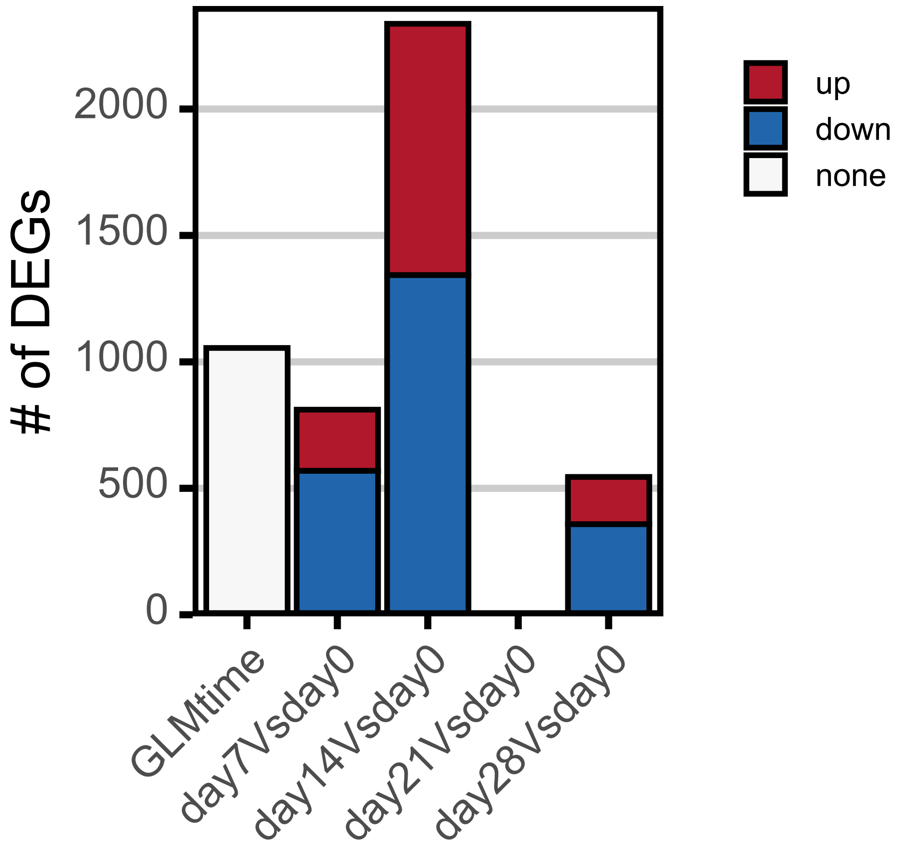


## *k*-means clustering of gene expression over time

```
# Retrieve genes that are differentially expressed in at least one time point
DEGs <- list()
DEGs$Geneid <- lapply(DESeq2_DEGs_filt[2:length(DESeq2_DEGs_filt)], function(x) x$Geneid)
DEGs$GeneSymbol <- lapply(DESeq2_DEGs_filt[2:length(DESeq2_DEGs_filt)], function(x) {
  x$GeneSymbol # keep uniquified gene symbols. To remove: gsub('(.+)_\\d+','\\1',x$GeneSymbol)
})
DEGs <- lapply(DEGs, function(x) x %>% unlist() %>% unique())

# Scale gene expression profiles
rnaseq_scaled <- t(scale(t(RNAseq$filt$DESeq_vst))) %>% as.data.frame()
rnaseq_scaled_DEGs <- rnaseq_scaled[DEGs$GeneSymbol,]
```

## Clustering evaluation

```
# Check within SS at different k
rng<-2:20 #k from 2 to 20
tries <-100 #Run the k Means algorithm 100 times
avg.totw.ss <-integer(length(rng)) #Set up an empty vector to hold all of points
for(v in rng){ # For each value of the range variable
  v.totw.ss <-integer(tries) #Set up an empty vector to hold the 100 tries
  for(i in 1:tries){
    k.temp <-kmeans(rnaseq_scaled_DEGs, centers=v) #Run kmeans
    v.totw.ss[i] <-k.temp$tot.withinss#Store the total withinss
  }
  avg.totw.ss[v-1] <-mean(v.totw.ss) #Average the 100 total withiness
}
```

```r
plot(rng,avg.totw.ss,type="b", main="Total Within SS by Various k",
     ylab="Average Total Within Sum of Squares",
     xlab="Value of k")
```

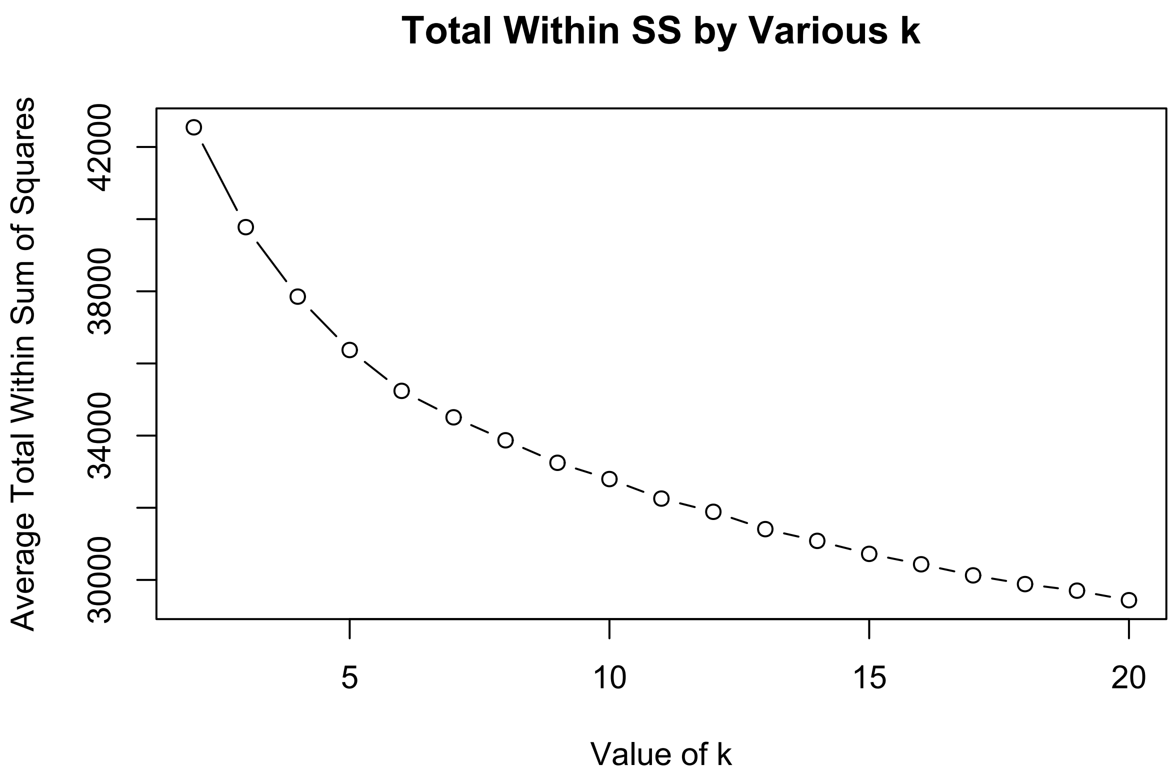


```r
# Check k-means partitions
fit <- cascadeKM(rnaseq_scaled_DEGs, 1, 20, iter = 100)
plot(fit, sortg = TRUE, grpmts.plot = TRUE)
```

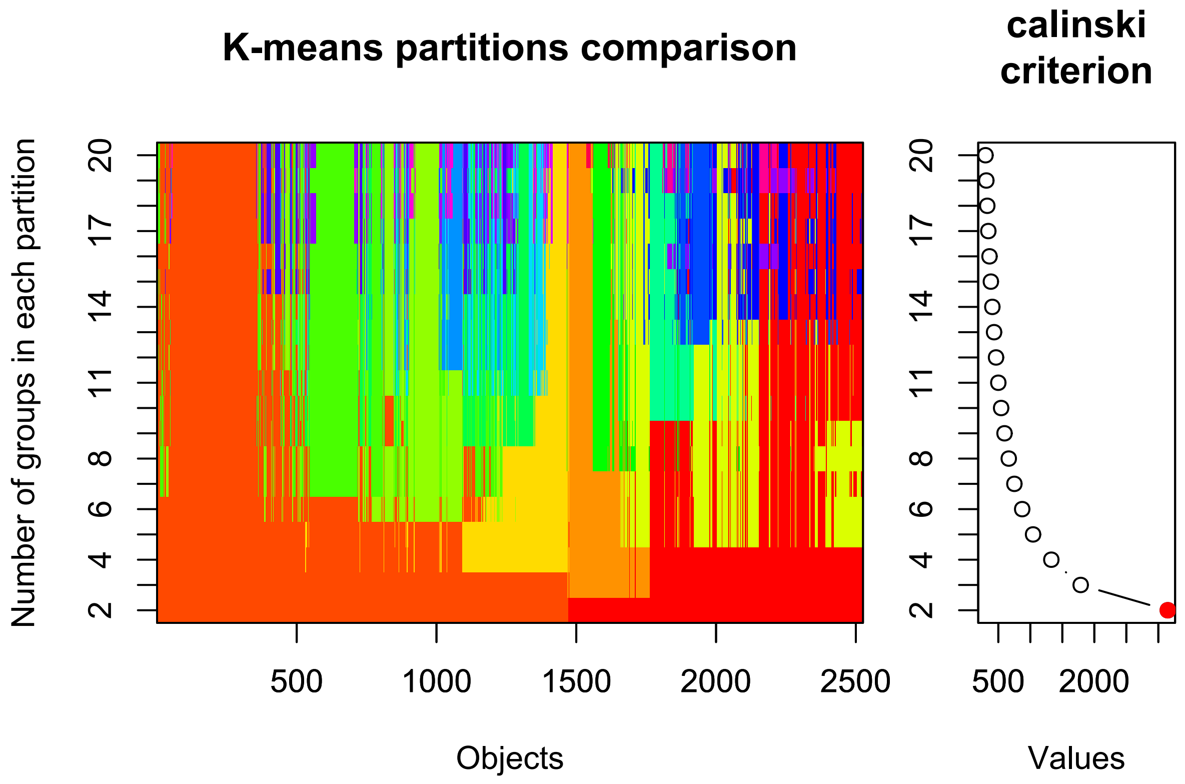


```
calinski.best <- as.numeric(which.max(fit$results[2,]))
cat("Calinski criterion optimal number of clusters:", calinski.best, "\n")
```

```
## Calinski criterion optimal number of clusters: 2
```

```
# Check gap statistic
set.seed(13)
gap <- clusGap(rnaseq_scaled_DEGs, kmeans, 20, B = 100, verbose = interactive())
plot(gap, main = "Gap statistic")
abline(v=which.max(gap$Tab[,3]), lty = 2)
```

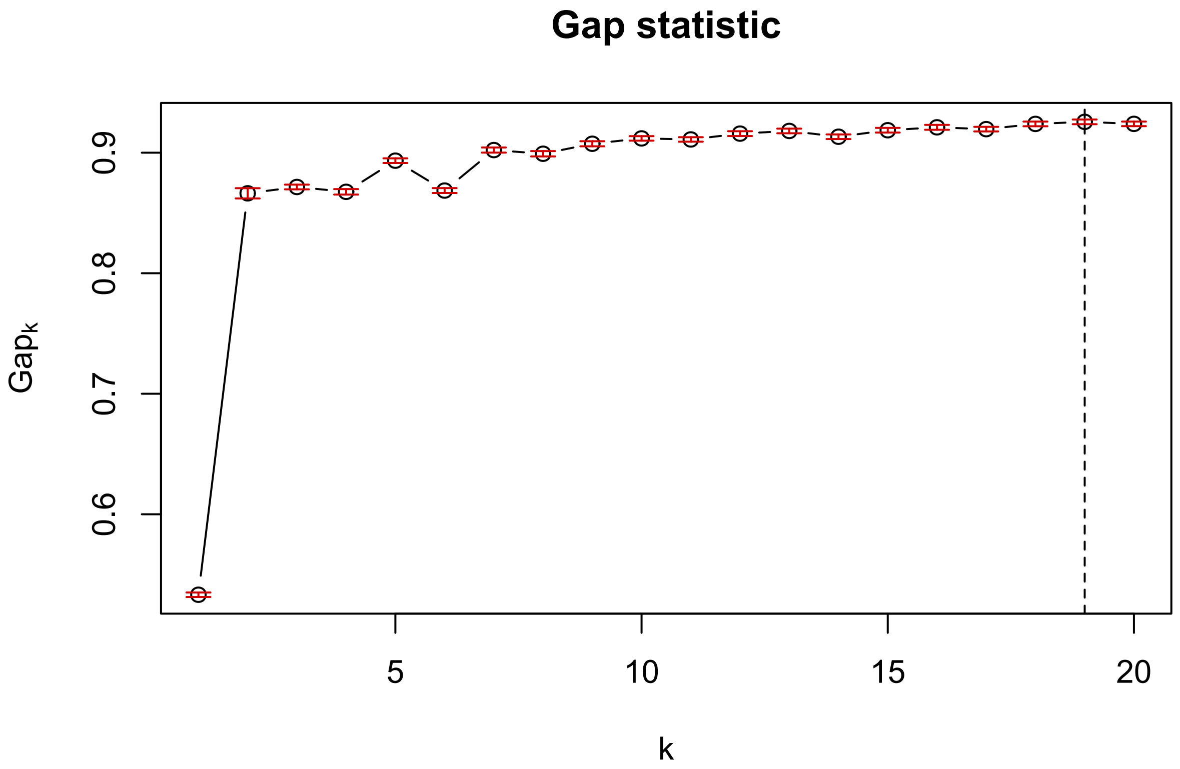


```r
# Check similarity of cluster profiles with k=4
set.seed(100)
DEGs_clust_k4 <- kmeans(rnaseq_scaled_DEGs, centers = 4)

df <- DEGs_clust_k4$centers %>%
  as.data.frame() %>%
  group.transform(group = RNAseq$filt$design$time,
                  FUN = function(x) apply(x, 1, mean))

cor(t(df))
```

```
##            1          2          3          4
## 1  1.0000000  0.9825767  0.9899959 -0.9953062
## 2  0.9825767  1.0000000  0.9928725 -0.9949472
## 3  0.9899959  0.9928725  1.0000000 -0.9933076
## 4 -0.9953062 -0.9949472 -0.9933076  1.0000000
```

```r
# Check similarity of cluster profiles with k=3
set.seed(100)
DEGs_clust_k3 <- kmeans(rnaseq_scaled_DEGs, centers = 3)

df <- DEGs_clust_k3$centers %>%
  as.data.frame() %>%
  group.transform(group = RNAseq$filt$design$time,
                  FUN = function(x) apply(x, 1, mean))

cor(t(df))
```

```
##            1          2          3
## 1  1.0000000 -0.9959542  0.9913322
## 2 -0.9959542  1.0000000 -0.9947338
## 3  0.9913322 -0.9947338  1.0000000
```

```r
# Check similarity of cluster profiles with k=2
set.seed(100)
DEGs_clust_k2 <- kmeans(rnaseq_scaled_DEGs, centers = 2)

df <- DEGs_clust_k2$centers %>%
  as.data.frame() %>%
  group.transform(group = RNAseq$filt$design$time,
                  FUN = function(x) apply(x, 1, mean))
```

```
cor(t(df))
```

```
##           1          2
## 1  1.0000000 -0.9966174
## 2 -0.9966174  1.0000000
```

# Visualize clusters ($k$=2)

## Expression profiles

```
# Average expression profiles per gene (cluster 1 = up, cluster 2 = down)
DEGs_clust_expr <- rnaseq_scaled_DEGs %>%
  group.transform(group = RNAseq$filt$design$time,
                  FUN = function(x) apply(x, 1, mean)) %>%
  rownames_to_column(var = 'GeneSymbol') %>%
  add_column(cluster = recode(.$GeneSymbol, !!!DEGs_clust_k2$cluster), .after = 'GeneSymbol') %>%
  mutate(cluster = recode(cluster, '1'='2', '2'='1'))

# Average expression profiles per cluster (cluster 1 = up, cluster 2 = down)
DEGs_clust_expr_mean <- DEGs_clust_k2$centers %>%
  as.data.frame() %>%
  group.transform(group = RNAseq$filt$design$time,
                  FUN = function(x) apply(x, 1, mean)) %>%
  rownames_to_column(var = 'cluster') %>%
  mutate(cluster = recode(cluster, '1'='2', '2'='1')) %>%
  arrange(cluster)
```

```
df <- DEGs_clust_expr %>%
  pivot_longer(day0:day28, names_to = 'time', values_to = 'expr') %>%
  mutate(cluster = factor(cluster, levels = c('1','2')),
         time = factor(time, levels = names(colPals$time)))

df2 <- DEGs_clust_expr_mean %>%
  pivot_longer(day0:day28, names_to = 'time', values_to = 'expr') %>%
  mutate(cluster = factor(cluster, levels = c('1','2')),
         time = factor(time, levels = names(colPals$time)))

lbls <- c("1" = paste0("Cluster 1 (n=",sum(DEGs_clust_expr$cluster=='1'),")"),
          "2" = paste0("Cluster 2 (n=",sum(DEGs_clust_expr$cluster=='2'),")"))

ggplot(df, aes(time, expr, color=cluster, group=GeneSymbol)) +
  geom_line(size = 1, color=alpha("#AEAEAE", 0.15)) +
  geom_line(data = df2, aes(time, expr, group=cluster), color='black', size = 1.2) +
  geom_point(data = df2, aes(time, expr, group=cluster, fill=time), shape=21, size=3.5, stroke=2, color='black') +
  scale_x_discrete(expand = expansion(mult = c(.06, .06)), labels = c('d0','d7','d14','d21','d28')) +
  scale_y_continuous(limits = c(-1.5,1.5),
                     breaks = seq(-1.5,1.5,0.5),
                     expand = expansion(mult = c(.1, .1))) +
  scale_fill_manual(values = colPals$time) +
  facet_wrap(~cluster, nrow = 1, labeller = as_labeller(lbls)) +
  ylab("Std. expression") +
  xlab("") +
  theme_bw(base_size=20) +
  theme(text = element_text(face = "plain"),
        axis.title.y = element_text(size=18, face = "bold"),
        axis.text.x = element_text(angle = 0, hjust = 0.5, vjust = 0.5),
        axis.text.y = element_text(hjust = 1, vjust = 0.3),
        legend.position = "none",
        axis.ticks = element_line(color = "black", size = 1.25),
        axis.ticks.length = unit(1.5, 'mm'),
        panel.border = element_rect(color = "black", fill = NA, size = 2),
        panel.grid.major.x = element_blank(),
        panel.grid.minor.x = element_blank(),
        panel.grid.major.y = element_blank(),
        panel.grid.minor.y = element_blank(),
        strip.background = element_blank()
        )
```

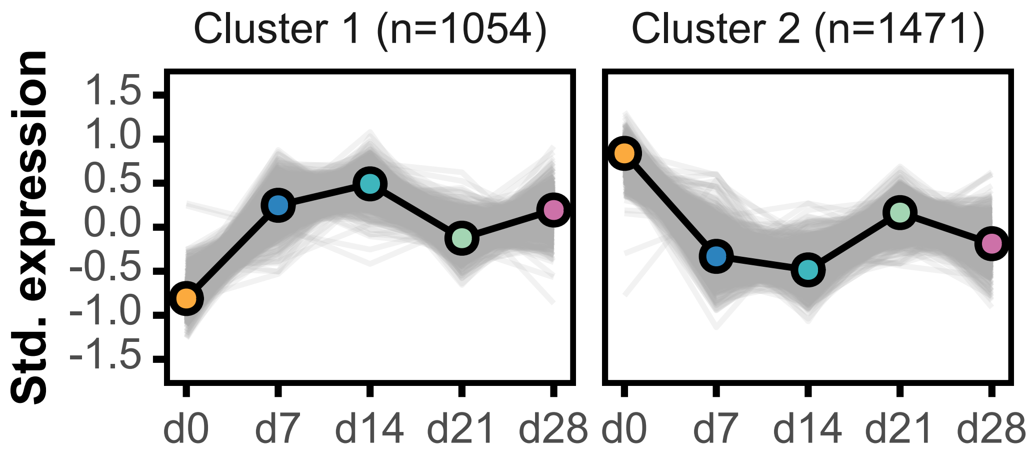


```
ggsave(filename = "plots/fig1G_DEGs_cluster_expr.pdf", width = 5.5, height = 3, units = "in", dpi = 300, device = cairo_pdf)
```

### Heatmap with selected genes

```
comparisons <- names(DESeq2_DEGs)[2:length(DESeq2_DEGs)]

DEGs_clusters_FC <- lapply(setNames(comparisons,comparisons), function(x) {
  DESeq2_DEGs[[x]] %>%
    filter(GeneSymbol %in% DEGs$GeneSymbol) %>%
    select(GeneSymbol, log2FoldChange) %>%
    rename_with(~x, log2FoldChange)
}) %>%
  purrr::reduce(left_join, by = 'GeneSymbol') %>%
  add_column(Cluster = recode(.$GeneSymbol, !!!DEGs_clust_k2$cluster), .after = 'GeneSymbol') %>%
  mutate(Cluster = recode(Cluster, '1'='2', '2'='1')) %>%
  add_column(day0Vsday0 = 0, .after = 'Cluster') %>%
  add_column(Geneid = recode(.$GeneSymbol, !!!setNames(RNAseq$filt$annotation$Geneid,
                                                        nm = RNAseq$filt$annotation$GeneSymbol)),
             .before = 'GeneSymbol') %>%
  arrange(Cluster) %>%
  mutate(Biotype = recode(.$GeneSymbol, !!!setNames(RNAseq$filt$annotation$gene_biotype,
                                                    nm = RNAseq$filt$annotation$GeneSymbol)))
```

```
# relevant genes to annotate
mark.genes <- c("IL7R","ETS1","GATA3","TCF7","TCF1","BCL11B",
                "SPI1","HES1","BCL11A","TCF12","BCL6","BCL2",
                "IER2",
                "CD27", # activation marker
                "CD3G",
                "CD69", # Early activation marker
                "CCR10","CCR2","CCR5",
                "CD160", # inhibits t cell activation
                "CD79B",
                "CD82","CD83",
                "RORA","RORC",
                "FOXP3",
                "CTLA4",
                "PDCD1",
                "CXCR4",
                "CXCL16",
                "ICOS",
                "IL2RA","IL2RB",
                "IL10RA",
                "EOMES",
                "SOCS1","SOCS3",
                "RHOH",
                "DUSP1","DUSP2","DUSP4","DUSP8","DUSP10",
                "FOS","FOSL2",
                "JUN","JUNB","JUND",
                "STAT5",
                "PRKCA",
                "ATF2"
```

```
)

m <- DEGs_clusters_FC %>%
  column_to_rownames(var = 'GeneSymbol') %>%
  select(day0Vsday0:day28Vsday0)

m2 <- DEGs_clusters_FC %>%
  column_to_rownames(var = 'GeneSymbol') %>%
  select(Biotype) %>%
  mutate(Biotype = ifelse(Biotype %in% c('protein_coding', 'lncRNA'), Biotype, 'other')) %>%
  mutate(Biotype = factor(Biotype, levels = c('protein_coding', 'lncRNA', 'other')))

comparison <- gsub('Vs', ' Vs ', colnames(m))

lbls <- c("1" = paste0("Cluster 1 (n=",sum(DEGs_clust_expr$cluster=='1'),")"),
          "2" = paste0("Cluster 2 (n=",sum(DEGs_clust_expr$cluster=='2'),")"))

ha_top <- HeatmapAnnotation(
  Comparison = factor(comparison, levels = unique(comparison)),
  col = list(
    Comparison = setNames(colPals$time,
                          nm = unique(comparison))
  ),
  annotation_name_gp = gpar(fontface = 'bold'),
  border = T
)

ha_right <- rowAnnotation(
  mark = anno_mark(at=which(rownames(m) %in% mark.genes),
                   labels = rownames(m)[which(rownames(m) %in% mark.genes)],
                   padding = unit(1,"mm"),
                   labels_gp = gpar(fontface = 'italic'))
)

p <- Heatmap(m, name = "log2FC",
             row_split=DEGs_clusters_FC$Cluster, cluster_row_slices = F, cluster_rows = T,
             column_title = NULL, cluster_columns = F,
             col = circlize::colorRamp2(breaks=seq(-2, 2, length.out=21),
                                        colors=colorRampPalette(rev(colPals$RdBu))(21)),
             top_annotation = ha_top,
             width = unit(50, "mm"),
             show_row_names = F, row_title = lbls, show_row_dend = T, row_dend_width=unit(10, "mm"), row_gap = unit(2, "mm"),
             show_column_names = T, column_names_gp = gpar(fontsize = 10), column_gap = unit(2, "mm"),
             border = T) +
  Heatmap(m2, name = "Biotype",
          col = colPals$biotype,
          right_annotation = ha_right,
          width = unit(10, "mm"),
          show_row_names = F,
          border = T)

draw(p, merge_legend = T, align_heatmap_legend = "heatmap_top")
```

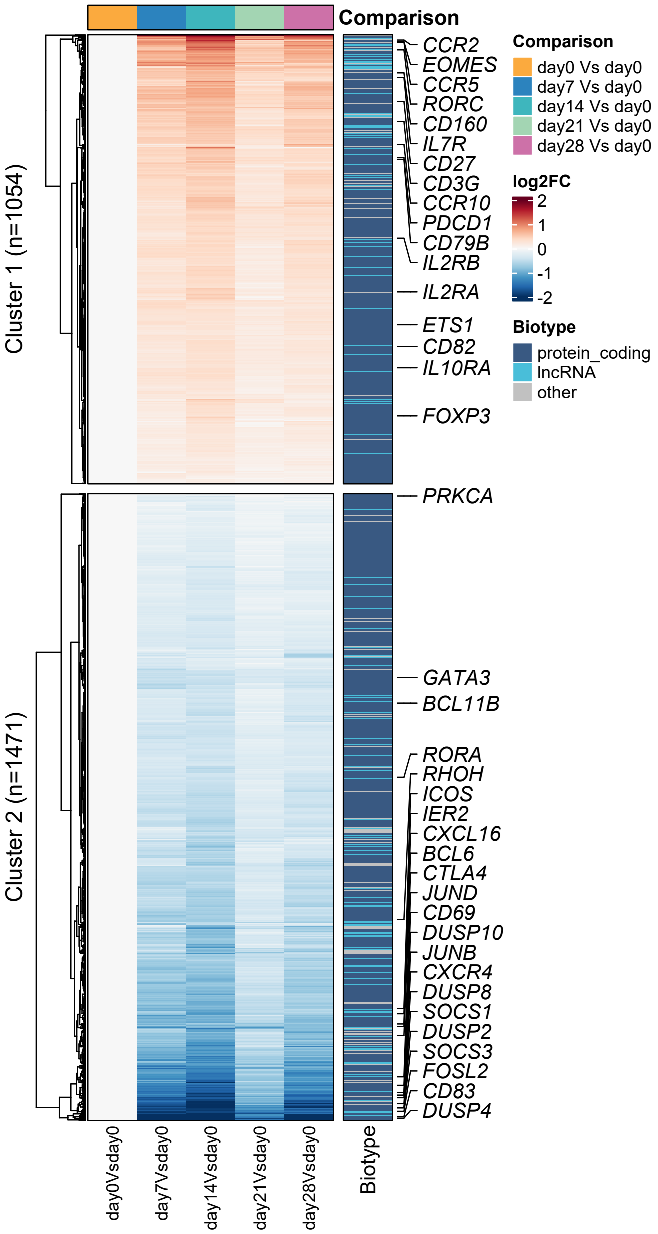


```
pdf("plots/fig2A_DEGs_cluster_heatmap_w_biotype.pdf", width = 10, height = 10)
draw(p, merge_legend = T, align_heatmap_legend = "heatmap_top")
dev.off()
```

```
## cairo_pdf
##         2
```

## Biotype distribution

```
df <- DEGs_clusters_FC %>%
  select(Cluster, Biotype) %>%
  mutate(Biotype = ifelse(Biotype %in% names(colPals$biotype), Biotype, 'other')) %>%
  mutate(Biotype = factor(Biotype, levels = names(colPals$biotype))) %>%
  group_by(Cluster,Biotype,.drop = FALSE) %>%
  summarise(n = n())

lbls <- c("1" = paste0("Cluster 1 (n=",sum(DEGs_clust_expr$cluster=='1'),")"),
          "2" = paste0("Cluster 2 (n=",sum(DEGs_clust_expr$cluster=='2'),")"))

ggplot(df, aes(x=Biotype, y=n, fill=Biotype)) +
```

```
  geom_bar(stat = "identity", width = 0.8, size = 1, colour = "black") +
  geom_text(aes(label = n), vjust = -0.5, size = 4) +
  facet_wrap(~Cluster, nrow = 1, labeller = as_labeller(lbls)) +
  xlab("") +
  ylab("# of genes") +
  scale_x_discrete(expand =expansion(mult = c(.08, .08))) +
  scale_y_continuous(expand =expansion(mult = c(.01, .1))) +
  scale_fill_manual(values = colPals$biotype) +
  theme_bw(base_size = 16) +
  theme(
    axis.title.y = element_text(face = 'bold', size = 16),
    axis.text.x.bottom = element_text(angle = 45, hjust = 1, vjust = 1, size = 14),
    axis.text.y = element_text(vjust = 0.3),
    panel.grid.major.y = element_line(color = "grey80", linetype = "solid", size = 1),
    panel.grid.major.x = element_blank(),
    panel.grid.minor = element_blank(),
    panel.border = element_rect(color = "black", fill = NA, size = 1),
    axis.ticks = element_line(color = "black", size = 1),
    legend.position = 'none',
    strip.background = element_blank(),
    strip.text = element_text(face = 'bold', size = 16)
  )
```

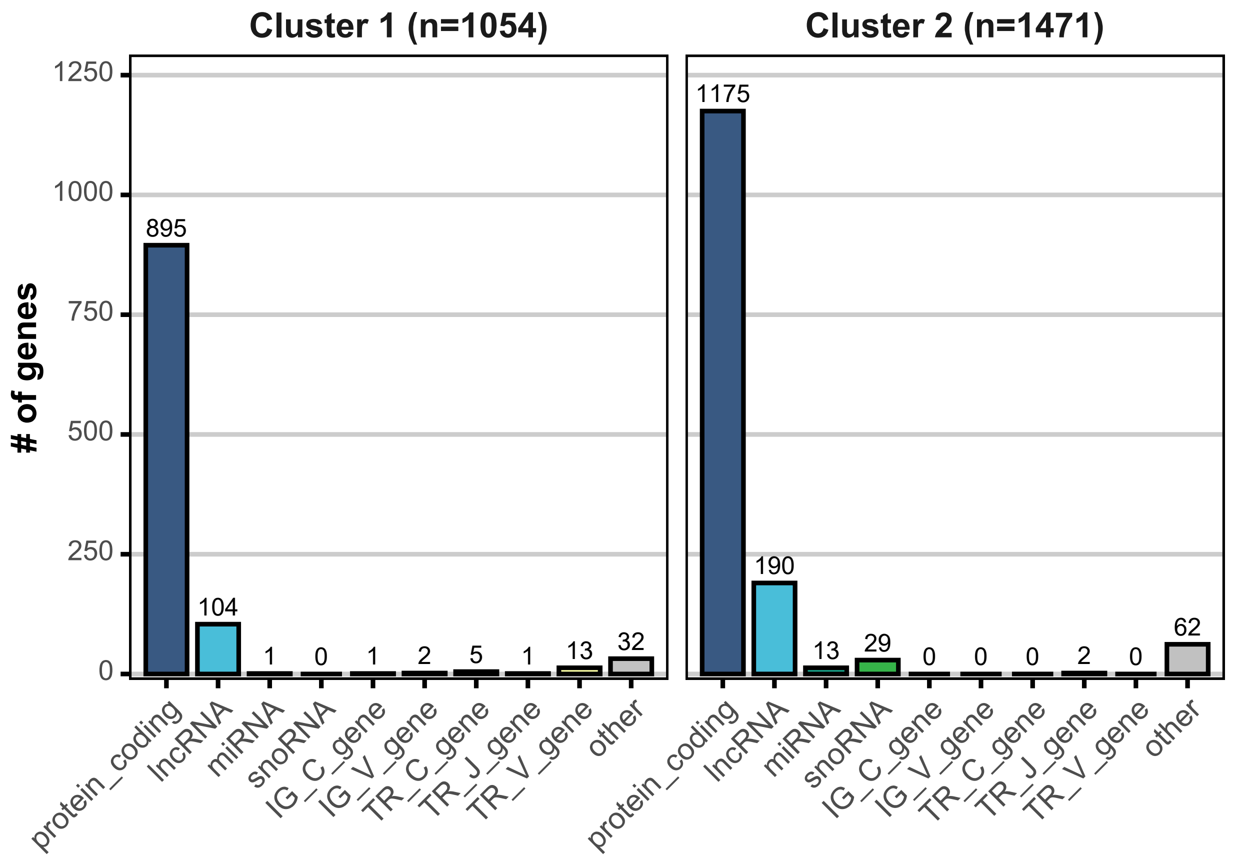


```
ggsave(filename = "plots/figS7_DEGs_cluster_biotype_distr.pdf", width = 8, height = 6, units = "in", dpi = 300, device = cairo_pdf)
```

# Exports

```
DEGs_clusters <- list(DEGs_clust_expr = DEGs_clust_expr,
                      DEGs_clust_expr_mean = DEGs_clust_expr_mean,
                      DEGs_clusters_FC = DEGs_clusters_FC)
saveRDS(DEGs_clusters, file= "data/rnaseq/DEGs_kmeans_clusters.rds")
```

```r
openxlsx::write.xlsx(
  list(DEGs_clusters_log2FC = DEGs_clusters_FC),
  file = "tables/dataS4_DEGs_cluster_assignment.xlsx",
  rowNames=F,
  overwrite=T
)
```

# SessionInfo

```r
sessionInfo()
```

```
## R version 4.2.1 (2022-06-23 ucrt)
## Platform: x86_64-w64-mingw32/x64 (64-bit)
## Running under: Windows 10 x64 (build 19044)
##
## Matrix products: default
##
## locale:
## [1] LC_COLLATE=English_United States.utf8
## [2] LC_CTYPE=English_United States.utf8
## [3] LC_MONETARY=English_United States.utf8
## [4] LC_NUMERIC=C
## [5] LC_TIME=English_United States.utf8
##
## attached base packages:
## [1] grid      stats     graphics  grDevices utils     datasets  methods
## [8] base
##
## other attached packages:
##  [1] ComplexHeatmap_2.14.0 cluster_2.1.3         vegan_2.6-4
##  [4] lattice_0.20-45       permute_0.9-7         RColorBrewer_1.1-3
##  [7] patchwork_1.1.2       magrittr_2.0.3        forcats_1.0.0
## [10] stringr_1.5.0         dplyr_1.1.1           purrr_1.0.1
## [13] readr_2.1.4           tidyr_1.3.0           tibble_3.2.1
## [16] ggplot2_3.4.2         tidyverse_1.3.2
##
## loaded via a namespace (and not attached):
##  [1] nlme_3.1-157        matrixStats_0.63.0  fs_1.6.1
##  [4] lubridate_1.9.2     doParallel_1.0.17   httr_1.4.5
##  [7] tools_4.2.1         backports_1.4.1     utf8_1.2.3
## [10] R6_2.5.1            DBI_1.1.3           BiocGenerics_0.44.0
## [13] mgcv_1.8-40         colorspace_2.1-0    GetoptLong_1.0.5
## [16] withr_2.5.0         tidyselect_1.2.0    compiler_4.2.1
## [19] cli_3.6.1           rvest_1.0.3         xml2_1.3.3
## [22] labeling_0.4.2      scales_1.2.1        digest_0.6.31
## [25] rmarkdown_2.21      pkgconfig_2.0.3     htmltools_0.5.5
## [28] highr_0.10          dbplyr_2.3.2        fastmap_1.1.1
## [31] rlang_1.1.0         GlobalOptions_0.1.2 readxl_1.4.2
## [34] rstudioapi_0.14     shape_1.4.6         generics_0.1.3
## [37] farver_2.1.1        jsonlite_1.8.4      zip_2.2.2
## [40] googlesheets4_1.1.0 Matrix_1.5-3        Rcpp_1.0.10
## [43] munsell_0.5.0       S4Vectors_0.36.2    fansi_1.0.4
## [46] lifecycle_1.0.3     stringi_1.7.12      yaml_2.3.7
## [49] MASS_7.3-57         parallel_4.2.1      crayon_1.5.2
## [52] haven_2.5.2         splines_4.2.1       circlize_0.4.15
## [55] hms_1.1.3           knitr_1.42          pillar_1.9.0
## [58] rjson_0.2.21        codetools_0.2-18    stats4_4.2.1
## [61] reprex_2.0.2        glue_1.6.2          evaluate_0.20
## [64] modelr_0.1.11       png_0.1-8           vctrs_0.6.1
## [67] tzdb_0.3.0          foreach_1.5.2       cellranger_1.1.0
## [70] gtable_0.3.3        clue_0.3-64         openxlsx_4.2.5.1
## [73] xfun_0.38           broom_1.0.4         googledrive_2.1.0
## [76] gargle_1.3.0        iterators_1.0.14    IRanges_2.32.0
## [79] timechange_0.2.0
```

# Step 2.2: Over-representation & gene set enrichment analysis

Carlos Gallardo & Christian Oertlin

17 April, 2023

```r
# Import libraries and helper functions
source("code/helper_functions.R")
library(tidyverse)
library(magrittr)
library(patchwork)
library(RColorBrewer)
library(qusage)
library(hypeR)
library(gage)
library(ComplexUpset)

# Colors
colPals <- vector(mode = "list")
colPals$time <- setNames(c("#FBAA3E", "#2C83BE", "#3EB6BD", "#A3D5B3", "#CD71A8"),
                         nm = c("day0", "day7", "day14", "day21", "day28"))
colPals$time_light <- setNames(c("#FDD6A1", "#A2CDE9", "#AFE2E5", "#DDF0E3", "#E8BDD6"),
                               nm = c("day0", "day7", "day14", "day21", "day28"))
colPals$time_dark <- setNames(c("#D87E04", "#174564", "#1F5C60", "#49A065", "#AA3C7E"),
                              nm = c("day0", "day7", "day14", "day21", "day28"))
colPals$inferno <- c("#000004", "#420A68", "#932667", "#DD513A", "#FCA50A", "#FCFFA4")
colPals$blood_cells <- setNames(c("#E54D34","#77A2D5","#B58B80"),
                                nm = c("granulocytes", "lymphocytes", "monocytes"))
colPals$cell_types <- setNames(c("#83D1F6","#FBAA3E","#FCCA7C","#B58B80","#E54D34",
                                 "#B3177E","#9A509F","#77A2D5","#CAC1DD","#36B449","#C1C1C1"),
                               nm = c("B cell", "Macrophage M1", "Macrophage M2",
                                      "Monocyte", "Neutrophil", "NK cell",
                                      "T cell CD4+ (non-regulatory)", "T cell CD8+",
                                      "T cell regulatory (Tregs)", "Myeloid dendritic cell",
                                      "uncharacterized cell"))
colPals$RdBu <- brewer.pal(11, name = "RdBu")
colPals$biotype <- setNames(c("#395982","#49BED9","#18A38A","#36B449","#826F99",
                              "#9852A5","#FBAA3E","#FCCA7C","#FCFFA4","#C1C1C1"),
                            nm = c("protein_coding", "lncRNA", "miRNA", "snoRNA",
                                   "IG_C_gene", "IG_V_gene", "TR_C_gene",
                                   "TR_J_gene", "TR_V_gene", "other"))
```

# Load data

```r
# RNA-seq
RNAseq <- readRDS(file='data/rnaseq/rnaseq_volunteers_9&10_excl.rds')
DESeq2_DEGs <- readRDS(file='data/rnaseq/DESeq2_DEGs_unfilt_volunteers_9&10_excl.rds')
DESeq2_DEGs_filt <- readRDS(file='data/rnaseq/DESeq2_DEGs_filt_volunteers_9&10_excl.rds')
DEGs_clusters <- readRDS(file='data/rnaseq/DEGs_kmeans_clusters.rds')

# Gene Sets
gene_sets <- list(
  # GO (2021/09/10) http://current.geneontology.org/products/pages/downloads.html
  gobp = readGMT('data/resources/gobp_human_sep2021.gmt'),
  gomf = readGMT('data/resources/gomf_human_sep2021.gmt'),
  gocc = readGMT('data/resources/gocc_human_sep2021.gmt'),
  # Reactome (2021/09/10) https://reactome.org/download-data
  reactome = readGMT('data/resources/reactome_human_sep2021.gmt'),
  # MSigDB v7.4 (2021/11/19) https://www.gsea-msigdb.org/gsea/msigdb/human/collections.jsp
  msig_immune = readGMT('data/resources/c7.immunesigdb.v7.4.symbols.gmt')
)
```

# Over-representation analysis (ORA)

```
# get unique gene names per cluster
DEG_sets <- list(
  cluster_1_up = DEGs_clusters$DEGs_clusters_FC %>%
    filter(Cluster == '1') %>%
    pull(GeneSymbol) %>%
    gsub('(.+)_\\d+','\\1',.) %>%
    str_subset(., pattern = '^ENS', negate = T) %>%
    unique(),
  cluster_2_down = DEGs_clusters$DEGs_clusters_FC %>%
    filter(Cluster == '2') %>%
    pull(GeneSymbol) %>%
    gsub('(.+)_\\d+','\\1',.) %>%
    str_subset(., pattern = '^ENS', negate = T) %>%
    unique()
)

# get gene background
gene_background <- RNAseq$filt$annotation$GeneSymbol %>%
  gsub('(.+)_\\d+','\\1',.) %>%
  str_subset(., pattern = '^ENS', negate = T) %>%
  unique()

# store over-representation analysis results
hyper_res <- list()
res_ORA <- list()
```

# GO Biological Process (GO_BP)

```
# GO Biological Process
hyper_res$gobp <- hypeR::hypeR(signature = DEG_sets,
                               genesets = gene_sets$gobp$genesets,
                               test = 'hypergeometric',
                               background = gene_background)

# append descriptions and filter
res_ORA$gobp <- lapply(setNames(names(DEG_sets),names(DEG_sets)), function(x) {
  hyper_res$gobp$data[[x]]$data %>%
    mutate(description=recode(label,
                              !!!setNames(gene_sets$gobp$geneset.descriptions,
                                          gene_sets$gobp$geneset.names))) %>%
    mutate(cluster = x,
           gene_set = 'GO_BP') %>%
    filter(fdr<0.05)
}) %>% bind_rows()

paste("GO Biological Process (FDR<0.05):", nrow(res_ORA$gobp))
```

```
## [1] "GO Biological Process (FDR<0.05): 5"
```

# GO Molecular Function (GO_MF)

```
# GO Molecular Function
hyper_res$gomf <- hypeR::hypeR(signature = DEG_sets,
                               genesets = gene_sets$gomf$genesets,
                               test = 'hypergeometric',
                               background = gene_background)

# append descriptions and filter
res_ORA$gomf <- lapply(setNames(names(DEG_sets),names(DEG_sets)), function(x) {
  hyper_res$gomf$data[[x]]$data %>%
    mutate(description=recode(label,
                              !!!setNames(gene_sets$gomf$geneset.descriptions,
                                          gene_sets$gomf$geneset.names))) %>%
    mutate(cluster = x,
           gene_set = 'GO_MF') %>%
    filter(fdr<0.05)
}) %>% bind_rows()

paste("GO Molecular Function (FDR<0.05):", nrow(res_ORA$gomf))
```

```
## [1] "GO Molecular Function (FDR<0.05): 20"
```

## GO Cellular Component (GO_CC)

```
# GO Cellular Component
hyper_res$gocc <- hypeR::hypeR(signature = DEG_sets,
                               genesets = gene_sets$gocc$genesets,
                               test = 'hypergeometric',
                               background = gene_background)

# append descriptions and filter
res_ORA$gocc <- lapply(setNames(names(DEG_sets),names(DEG_sets)), function(x) {
  hyper_res$gocc$data[[x]]$data %>%
    mutate(description=recode(label,
                              !!!setNames(gene_sets$gocc$geneset.descriptions,
                                          gene_sets$gocc$geneset.names))) %>%
    mutate(cluster = x,
           gene_set = 'GO_CC') %>%
    filter(fdr<0.05)
}) %>% bind_rows()

paste("GO Cellular Component (FDR<0.05):", nrow(res_ORA$gocc))
```

```
## [1] "GO Cellular Component (FDR<0.05): 18"
```

## Reactome Pathways (REACT)

```
# Reactome Pathways
hyper_res$reactome <- hypeR::hypeR(signature = DEG_sets,
                               genesets = gene_sets$reactome$genesets,
                               test = 'hypergeometric',
                               background = gene_background)

# append descriptions and filter
res_ORA$reactome <- lapply(setNames(names(DEG_sets),names(DEG_sets)), function(x) {
  hyper_res$reactome$data[[x]]$data %>%
    mutate(description=recode(label,
                              !!!setNames(gene_sets$reactome$geneset.descriptions,
                                          gene_sets$reactome$geneset.names))) %>%
    mutate(cluster = x,
           gene_set = 'REACT') %>%
    filter(fdr<0.05)
}) %>% bind_rows()

paste("Reactome Pathways (FDR<0.05):", nrow(res_ORA$reactome))
```

```
## [1] "Reactome Pathways (FDR<0.05): 3"
```

```
res_ORA_all <- res_ORA %>%
  bind_rows() %>%
  mutate(overlap_ratio = overlap/geneset,
         cluster = recode(cluster, !!!setNames(c('1', '2'), names(DEG_sets)))) %>%
  select(gene_set, cluster, label, description, geneset,
         overlap, overlap_ratio, pval, fdr, hits)

lbls <- c("1" = paste0("Cluster 1 (n=",sum(DEGs_clusters$DEGs_clust_expr$cluster=='1'),")"),
          "2" = paste0("Cluster 2 (n=",sum(DEGs_clusters$DEGs_clust_expr$cluster=='2'),")"),
          "GO_BP" = "GO_BP",
          "GO_MF" = "GO_MF",
          "GO_CC" = "GO_CC",
          "REACT" = "REACT")

ggplot(res_ORA_all, aes(x=overlap_ratio, y=description, fill=cluster, label=overlap)) +
  geom_bar(stat='identity', size=1, width=0.6, color='black') +
  geom_text(color = 'black', angle = 0, hjust=-0.2, vjust=0.4, fontface='bold') +
  scale_x_continuous(breaks = c(0,0.5,1),
                     limits = c(0,1),
                     expand = expansion(mult = c(.01, .05))) +
  scale_fill_manual(values = colPals$RdBu[c(2,10)]) +
  facet_grid(gene_set~cluster, scales="free_y", space = "free_y", labeller = as_labeller(lbls)) +
  xlab('Overlap Ratio') +
  ylab('') +
  theme_bw() +
  theme(
    text = element_text(family = 'Arial', size = 14),
    axis.text.x.bottom = element_text(size = 10, hjust = 0.5, vjust = 0.5),
    axis.text.y.left = element_text(size = 10, hjust = 1, vjust = 0.3),
    panel.border = element_rect(color = "black", fill = NA, size = 1),
    axis.ticks = element_line(color = "black", size = 1),
    axis.ticks.length = unit(1.5, 'mm'),
```

```
    panel.grid.major.x = element_line(color = "grey80", linetype = "solid", size = 1),
    panel.grid.minor.x = element_line(color = "grey80", linetype = "solid", size = 1),
    panel.grid.major.y = element_blank(),
    panel.grid.minor.y = element_blank(),
    legend.position = 'none',
    strip.background = element_blank(),
    strip.text = element_text(face = "bold"),
    strip.text.y = element_text(angle = 90)
  )
```

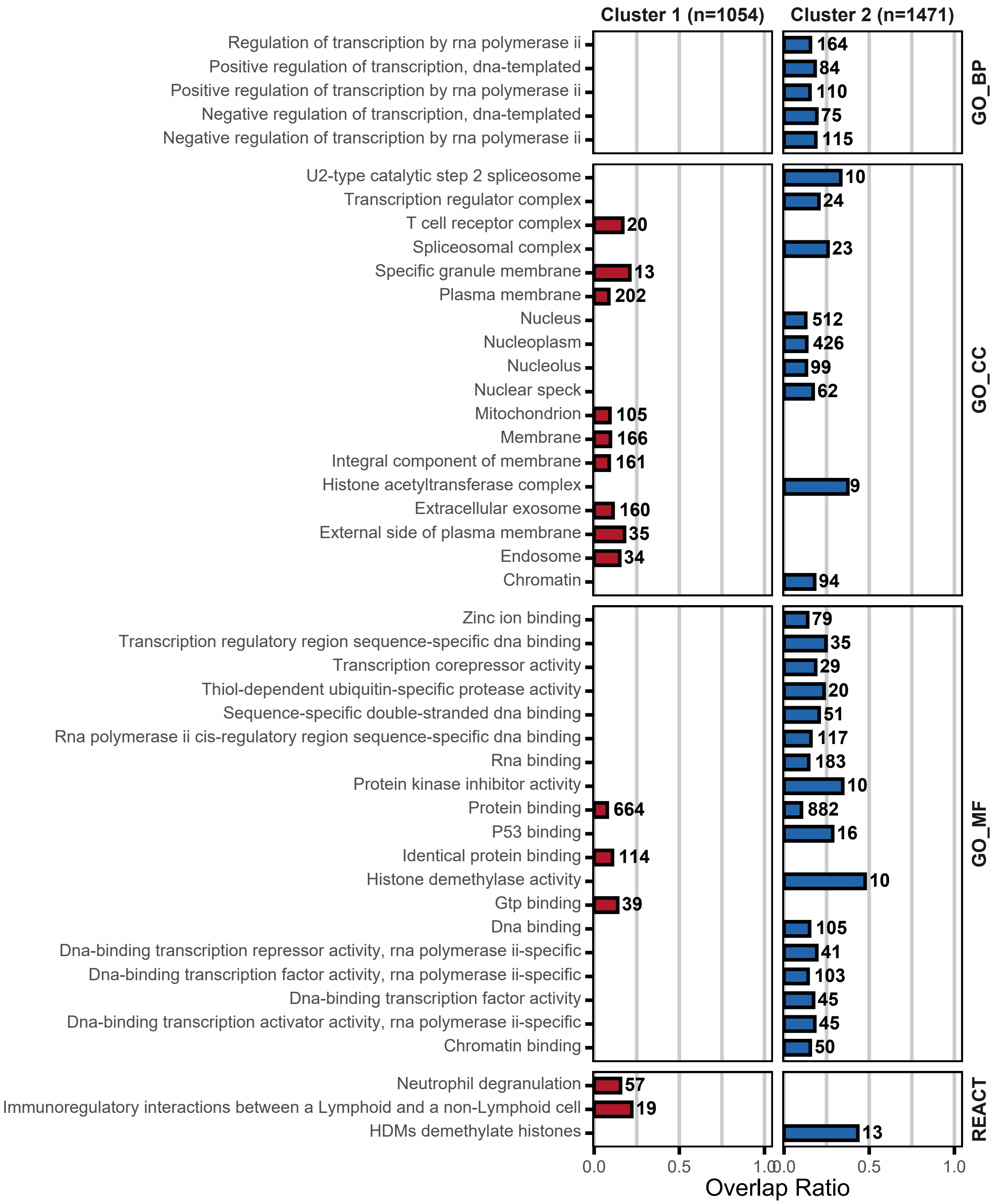

```
ggsave(filename = "plots/figS6_DEGs_clusters_ORA_results.pdf", width = 8.5, height = 10, units = "in", dpi = 300, device = cairo_pdf)
```

# Gene set enrichment analysis (GSEA)

```
# run GSEA on immune gene sets from MSigDB (Broad Institute)
# filter gene sets with little relevance to isolated T cells (i.e., B cells, etc.)
gene_sets$msig_immune$genesets <- gene_sets$msig_immune$genesets[grepl("TCELL",names(gene_sets$msig_immune$genesets))]
gene_sets$msig_immune$genesets <- gene_sets$msig_immune$genesets[!grepl("NK",names(gene_sets$msig_immune$genesets))]
gene_sets$msig_immune$genesets <- gene_sets$msig_immune$genesets[!grepl("MAST",names(gene_sets$msig_immune$genesets))]
gene_sets$msig_immune$genesets <- gene_sets$msig_immune$genesets[!grepl("BCELL",names(gene_sets$msig_immune$genesets))]
gene_sets$msig_immune$genesets <- gene_sets$msig_immune$genesets[!grepl("MONOCYTE",names(gene_sets$msig_immune$genesets))]
gene_sets$msig_immune$genesets <- gene_sets$msig_immune$genesets[!grepl("NEUTROPHIL",names(gene_sets$msig_immune$genesets))]
gene_sets$msig_immune$genesets <- gene_sets$msig_immune$genesets[!grepl("EOSINOPHIL",names(gene_sets$msig_immune$genesets))]
gene_sets$msig_immune$genesets <- gene_sets$msig_immune$genesets[!grepl("DC",names(gene_sets$msig_immune$genesets))]
gene_sets$msig_immune$genesets <- gene_sets$msig_immune$genesets[!grepl("HIV",names(gene_sets$msig_immune$genesets))]
tokeep <- gene_sets$msig_immune$geneset.names %in% names(gene_sets$msig_immune$genesets)
gene_sets$msig_immune$geneset.names <- gene_sets$msig_immune$geneset.names[tokeep]
gene_sets$msig_immune$geneset.descriptions <- gene_sets$msig_immune$geneset.descriptions[tokeep]


# rank genes by different time point comparisons
gene_ranks <- lapply(DESeq2_DEGs[2:length(DESeq2_DEGs)], function(x) {
  df <- x %>%
    mutate(GeneSymbol = gsub('(.+)_\\d+','\\1',.$GeneSymbol)) %>%
    filter(!duplicated(GeneSymbol)) %>%
    mutate(rank_score = -log10(padj) * sign(log2FoldChange)) %>%
    arrange(desc(rank_score))
  rank_score <- setNames(df$rank_score, df$GeneSymbol)
  rank(rank_score)
})

gage_GSEA <- runGage(gene_ranks, gene_sets$msig_immune$genesets, cutOff = 0.05)
```

```
## [1] "gs.data needs to be a matrix-like object!"
## [1] "gs.data needs to be a matrix-like object!"
## [1] "gs.data needs to be a matrix-like object!"
## [1] "there are 30 signficantly up-regulated gene sets"
## [1] "there are 59 signficantly down-regulated gene sets"
## [1] "gs.data needs to be a matrix-like object!"
## [1] "gs.data needs to be a matrix-like object!"
## [1] "gs.data needs to be a matrix-like object!"
## [1] "there are 90 signficantly up-regulated gene sets"
## [1] "there are 50 signficantly down-regulated gene sets"
## [1] "gs.data needs to be a matrix-like object!"
## [1] "gs.data needs to be a matrix-like object!"
## [1] "gs.data needs to be a matrix-like object!"
## [1] "there are 24 signficantly up-regulated gene sets"
## [1] "there are 52 signficantly down-regulated gene sets"
## [1] "gs.data needs to be a matrix-like object!"
## [1] "gs.data needs to be a matrix-like object!"
## [1] "gs.data needs to be a matrix-like object!"
## [1] "there are 40 signficantly up-regulated gene sets"
## [1] "there are 56 signficantly down-regulated gene sets"
```

```
res_GSEA_sig <- lapply(setNames(names(gage_GSEA),names(gage_GSEA)), function(x) {
  greater <- gage_GSEA[[x]]$sig$greater %>%
    as.data.frame() %>%
    rownames_to_column(var = 'description') %>%
    mutate(group = 'greater',
           comparison = x)
  less <- gage_GSEA[[x]]$sig$less %>%
    as.data.frame() %>%
    rownames_to_column(var = 'description') %>%
    mutate(group = 'less',
           comparison = x)
  bind_rows(greater, less)
}) %>% bind_rows()

# curate and group relevant immune sets that are enriched
immune_set_groups <- res_GSEA_sig %>%
  arrange(q.val) %>%
  filter(!duplicated(description)) %>%
  select(description) %>%
  mutate(label = '') %>%
  # untreated CD4 T cells
  mutate(label = ifelse(grepl('72H',description) & grepl('UNTREATED',description) &
                        grepl('CD4',description) & grepl('UP',description),
```

```
                         '72h untr. CD4: UP', label)) %>%
  mutate(label = ifelse(grepl('72H',description) & grepl('UNTREATED',description) &
                           grepl('CD4',description) & grepl('DN',description),
                         '72h untr. CD4: DN', label)) %>%
  # activated memory CD4 T cells
  mutate(label = ifelse(grepl('ACT',description) & grepl('MEMORY',description) &
                           grepl('CD4',description) & grepl('UP',description),
                         'Activated CD4 Memory: UP', label)) %>%
  mutate(label = ifelse(grepl('ACT',description) & grepl('MEMORY',description) &
                           grepl('CD4',description) & grepl('DN',description),
                         'Activated CD4 Memory: DN', label)) %>%
  # activated CD4 T cells
  mutate(label = ifelse(grepl('ACT',description) & !grepl('MEMORY|TH2|TH1',description) &
                           grepl('CD4',description) & grepl('UP',description),
                         'Activated CD4: UP', label)) %>%
  mutate(label = ifelse(grepl('ACT',description) & !grepl('MEMORY|TH2|TH1',description) &
                           grepl('CD4',description) & grepl('DN',description),
                         'Activated CD4: DN', label)) %>%
  # conventional T regs vs CD4 naive
  mutate(label = ifelse(grepl('NAIVE_CD4',description) & grepl('CONV_TREG',description) &
                           grepl('UP',description),
                         'Conv. Treg vs CD4 Naive: UP', label)) %>%
  mutate(label = ifelse(grepl('NAIVE_CD4',description) & grepl('CONV_TREG',description) &
                           grepl('DN',description),
                         'Conv. Treg vs CD4 Naive: DN', label)) %>%
  # CD4 vs CD8 naive
  mutate(label = ifelse(grepl('CD8_VS_CD4_NAIVE',description) & grepl('UP',description),
                         'CD4 Naive vs CD8 Naive: UP', label)) %>%
  mutate(label = ifelse(grepl('CD8_VS_CD4_NAIVE',description) & grepl('DN',description),
                         'CD4 Naive vs CD8 Naive: DN', label)) %>%
  # CD8 naive vs CD8 stem memory
  mutate(label = ifelse(grepl('CD8_STEM_CELL_MEMORY_VS_NAIVE_CD8',description) & grepl('UP',description),
                         'CD8 Naive vs CD8 Stem Memory: UP', label)) %>%
  mutate(label = ifelse(grepl('CD8_STEM_CELL_MEMORY_VS_NAIVE_CD8',description) & grepl('DN',description),
                         'CD8 Naive vs CD8 Stem Memory: DN', label)) %>%
  # CD4 HDAC inhibitor-treated
  mutate(label = ifelse(grepl('HDAC_INHIBITOR_TREATED_CD4',description) & grepl('DN',description),
                         'CD4 HDAC inhibitor: DN', label)) %>%
  # TCF1 KO
  mutate(label = ifelse(grepl('WT_VS_TCF1_KO_MEMORY_CD8_TCELL_UP',description),
                         'TCF1 KO: UP', label)) %>%
  # BCL6 Low
  mutate(label = ifelse(grepl('BCL6_HIGH_TFH_VS_TFH_CD4_TCELL_DN',description),
                         'BCL6 Low: DN', label)) %>%
  filter(!label == '')

# intersect gene sets to gene background
immune_sets_background <- lapply(gene_sets$msig_immune$genesets, function(x) {
  genes_in_background <- intersect(x, gene_background)
  genes_in_background
})

# get enrichment scores for all enriched sets in all comparisons
res_GSEA_all_selected <- lapply(setNames(names(gage_GSEA),names(gage_GSEA)), function(x) {

  greater <- gage_GSEA[[x]]$all$greater %>%
    as.data.frame() %>%
    rownames_to_column(var = 'description') %>%
    filter(description %in% immune_set_groups$description & stat.mean > 0) %>%
    mutate(DEG_overlap = lapply(immune_sets_background[description], function(x){
      length(intersect(x, unlist(DEG_sets)))
    }) %>% unlist() %>% as.numeric()) %>%
    mutate(overlap_ratio = DEG_overlap/set.size) %>%
    mutate(group = 'greater',
           comparison = x,
           label = recode(description, !!!setNames(immune_set_groups$label,
                                                  nm = immune_set_groups$description)),
           hits = lapply(immune_sets_background[description], function(x){
             paste(x, collapse = ',')
           }) %>% unlist() %>% as.character())

  less <- gage_GSEA[[x]]$all$less %>%
    as.data.frame() %>%
    rownames_to_column(var = 'description') %>%
    filter(description %in% immune_set_groups$description & stat.mean < 0) %>%
    mutate(DEG_overlap = lapply(immune_sets_background[description], function(x){
      length(intersect(x, unlist(DEG_sets)))
    }) %>% unlist() %>% as.numeric()) %>%
    mutate(overlap_ratio = DEG_overlap/set.size) %>%
    mutate(group = 'less',
```

```
           comparison = x,
           label = recode(description, !!!setNames(immune_set_groups$label,
                                                   nm = immune_set_groups$description)),
           hits = lapply(immune_sets_background[description], function(x){
             paste(x, collapse = ',')
           }) %>% unlist() %>% as.character())

  bind_rows(greater, less)

}) %>%
  bind_rows() %>%
  dplyr::rename(p_geomean = p.geomean, enrichment_score = stat.mean, pval = p.val, qval = q.val,
                set_size = set.size) %>%
  select(label, description, comparison, group, p_geomean, enrichment_score,
         set_size, DEG_overlap, overlap_ratio, exp1, pval, qval, hits)

# collapse immune set groups and average enrichment scores by comparison
immune_set_group_lbls <- c(
  'Activated CD4: UP', 'Activated CD4: DN', 'Activated CD4 Memory: UP', 'Activated CD4 Memory: DN',
  '72h untr. CD4: UP', '72h untr. CD4: DN', 'CD4 Naive vs CD8 Naive: UP', 'CD4 Naive vs CD8 Naive: DN',
  'Conv. Treg vs CD4 Naive: UP', 'Conv. Treg vs CD4 Naive: DN', 'CD8 Naive vs CD8 Stem Memory: UP',
  'CD8 Naive vs CD8 Stem Memory: DN', 'CD4 HDAC inhibitor: DN', 'TCF1 KO: UP', 'BCL6 Low: DN'
)
res_GSEA_collapsed <- lapply(setNames(immune_set_group_lbls,immune_set_group_lbls), function(i){
  df <- res_GSEA_all_selected %>%
    filter(label == i) %>%
    group_by(label, comparison)

  df %>%
    summarise(enrichment_score_mean = mean(enrichment_score),
              pval_mean = mean(pval),
              qval_mean = mean(qval)) %>%
    mutate(hits = lapply(df$hits, function(x){
      strsplit(x, split = ',')
    }) %>% unlist() %>% unique() %>% paste(collapse = ','))
}) %>%
  bind_rows() %>%
  mutate(set_size = lapply(hits, function(x) {
    strsplit(x, ',')[[1]] %>% length()
  }) %>% unlist() %>% as.numeric(),
  DEG_overlap = lapply(hits, function(x){
    strsplit(x, ',')[[1]] %>% intersect(., unlist(DEG_sets)) %>% length()
  }) %>% unlist() %>% as.numeric()) %>%
  mutate(overlap_ratio = DEG_overlap/set_size) %>%
  select(label, comparison, enrichment_score_mean, set_size, DEG_overlap,
         overlap_ratio, pval_mean, qval_mean, hits) %>%
  mutate(label = factor(label, levels = immune_set_group_lbls),
         comparison = factor(comparison, levels = c('day7Vsday0','day14Vsday0',
                                                    'day21Vsday0','day28Vsday0'))) %>%
  arrange(label, comparison)
```

## Average enrichment of collapsed immune sets

```
df <- res_GSEA_collapsed %>%
  mutate(type = ifelse(enrichment_score_mean > 0, 'up', 'down')) %>%
  mutate(type = factor(type, levels = c('up', 'down')))

ggplot(df, aes(x=label, y=enrichment_score_mean, fill=type)) +
  geom_bar(stat='identity', size=1, width=0.6, color='black') +
  geom_hline(yintercept = 0, color='black', size=1) +
  scale_y_continuous(breaks = c(-10,-5,0,5,10),
                     limits = c(-10,10),
                     expand = expansion(mult = c(.01, .05))) +
  scale_fill_manual(values = colPals$RdBu[c(2,10)]) +
  facet_wrap(~comparison, ncol=1, strip.position='right') +
  xlab('') +
  ylab('Enrichment score') +
  theme_bw() +
  theme(
    text = element_text(family = 'Arial', size = 14),
    axis.text.x.bottom = element_text(angle=50, size = 12, hjust = 1, vjust = 1),
    axis.text.y.left = element_text(size = 12, hjust = 1, vjust = 0.3),
    panel.border = element_rect(color = "black", fill = NA, size = 1),
    axis.ticks = element_line(color = "black", size = 1),
    axis.ticks.length = unit(1.1, 'mm'),
    panel.grid.major.y = element_line(color = "grey80", linetype = "solid", size = 1),
    panel.grid.minor.y = element_blank(),
    panel.grid.major.x = element_blank(),
```

```
    panel.grid.minor.x = element_blank(),
    legend.position = 'none',
    strip.background = element_blank(),
    strip.text = element_text(face = "bold"),
    strip.text.y = element_text(angle = 0)
  )
```

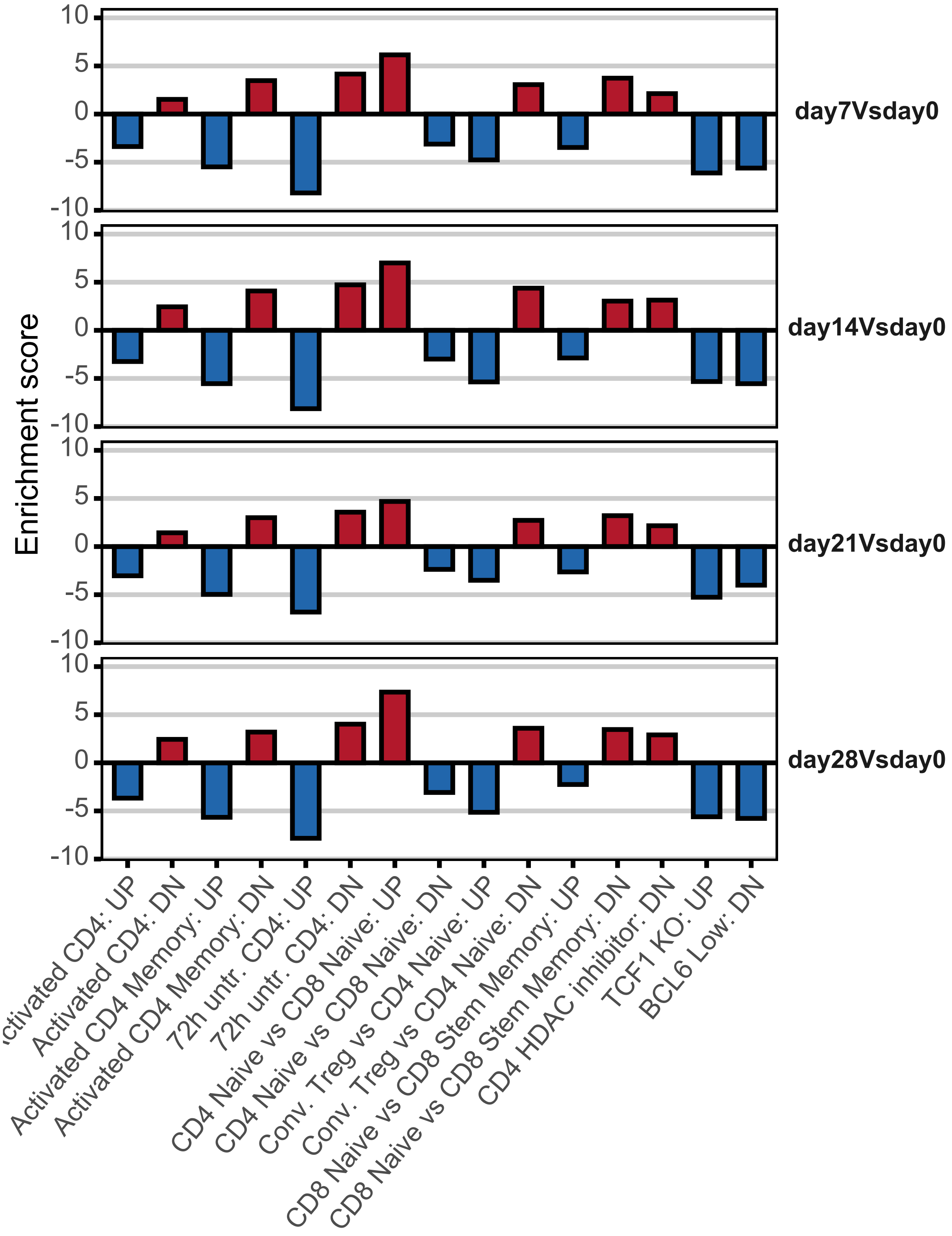

```
ggsave(filename = "plots/fig2C_GSEA_immune_gene_sets.pdf", width = 6, height = 9, units = "in", dpi = 300, device = cairo_pdf)
```

## Gene overlap between immune sets

```
immune_set_all_genes <- lapply(setNames(immune_set_group_lbls,immune_set_group_lbls), function(x){
  genes <- res_GSEA_collapsed %>%
    filter(label == x) %>%
    pull(hits) %>%
    unique()
  strsplit(genes, split = ',')[[1]]
}) %>% unlist() %>% unique()

gene_expr_change <- DESeq2_DEGs$day14Vsday0 %>%
  mutate(GeneSymbol = gsub('(.+)_\\d+','\\1',GeneSymbol)) %>%
  filter(GeneSymbol %in% immune_set_all_genes) %>%
  filter(!duplicated(GeneSymbol)) %>%
  mutate(change = 'unchanged') %>%
  mutate(change = ifelse(GeneSymbol %in% unlist(DEG_sets) & log2FoldChange > 0, 'up', change)) %>%
  mutate(change = ifelse(GeneSymbol %in% unlist(DEG_sets) & log2FoldChange < 0, 'down', change))

gene_expr_change <- setNames(gene_expr_change$change,
                             nm = gene_expr_change$GeneSymbol)

df <- lapply(setNames(immune_set_group_lbls,immune_set_group_lbls), function(x){
  genes <- res_GSEA_collapsed %>%
    filter(label == x) %>%
    pull(hits) %>%
    unique()
  genes <- strsplit(genes, split = ',')[[1]]
  immune_set_all_genes %in% genes
}) %>%
  bind_cols() %>%
  mutate(genes = immune_set_all_genes) %>%
  mutate(change = recode(genes, !!!gene_expr_change)) %>%
  column_to_rownames(var = 'genes') %>%
  mutate(change = factor(change, levels = c('up', 'down', 'unchanged')))

upset(df,
      immune_set_group_lbls,
      min_size = 20,
      set_sizes=upset_set_size(position = 'left'),
      base_annotations=list(
        'Intersection size'=intersection_size(
          counts=F,
          mapping=aes(fill=change),
          color="black",
          size=0.5
        ) + scale_fill_manual(values = c(colPals$RdBu[c(2,10)], '#C1C1C1'))
      ),
      name='Intersection',
      width_ratio=0.1,
      height_ratio=0.7,
      sort_sets=FALSE)
```

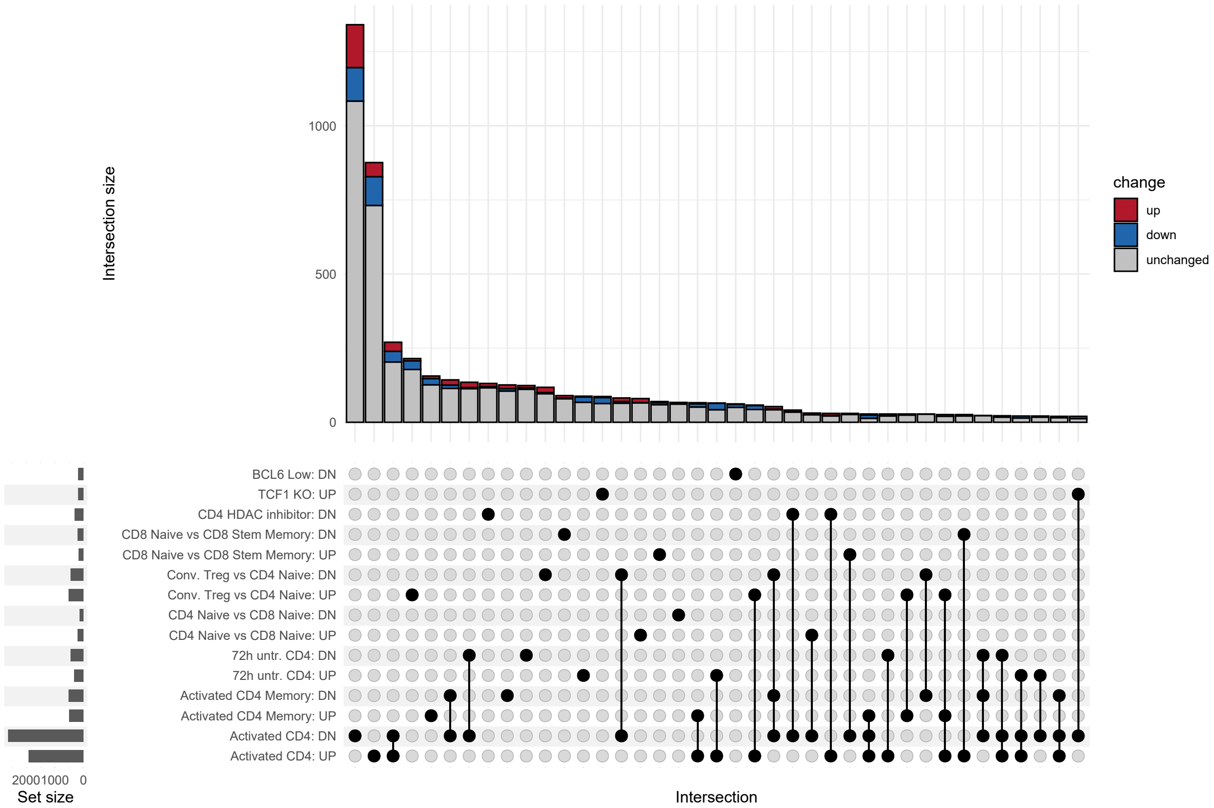


```
ggsave(filename = "plots/figS8_GSEA_immune_gene_sets_overlap.pdf", width = 12, height = 6, units = "in", dpi = 300, device = cairo_pdf)
```

# Exports

```
gene_set_analysis_res <- list(ORA_results = res_ORA_all,
                              GSEA_immune = res_GSEA_all_selected,
                              GSEA_immune_collapsed = res_GSEA_collapsed)

openxlsx::write.xlsx(
  gene_set_analysis_res,
  file = "tables/dataS5_gene_set_analysis.xlsx",
  rowNames=F,
  overwrite=T
)
```

# SessionInfo

```
sessionInfo()
```

```
## R version 4.2.1 (2022-06-23 ucrt)
## Platform: x86_64-w64-mingw32/x64 (64-bit)
## Running under: Windows 10 x64 (build 19044)
##
## Matrix products: default
##
## locale:
## [1] LC_COLLATE=English_United States.utf8
## [2] LC_CTYPE=English_United States.utf8
## [3] LC_MONETARY=English_United States.utf8
## [4] LC_NUMERIC=C
## [5] LC_TIME=English_United States.utf8
##
## attached base packages:
## [1] stats     graphics  grDevices utils     datasets  methods   base
##
```

```
## other attached packages:
##  [1] ComplexUpset_1.3.5 gage_2.48.0        hypeR_1.14.0       qusage_2.32.0
##  [5] limma_3.54.2       RColorBrewer_1.1-3 patchwork_1.1.2    magrittr_2.0.3
##  [9] forcats_1.0.0      stringr_1.5.0      dplyr_1.1.1        purrr_1.0.1
## [13] readr_2.1.4        tidyr_1.3.0        tibble_3.2.1       ggplot2_3.4.2
## [17] tidyverse_1.3.2
##
## loaded via a namespace (and not attached):
##   [1] googledrive_2.1.0      colorspace_2.1-0       ellipsis_0.3.2
##   [4] estimability_1.4.1     XVector_0.38.0         fs_1.6.1
##   [7] rstudioapi_0.14        farver_2.1.1           bit64_4.0.5
##  [10] AnnotationDbi_1.60.2   fansi_1.0.4            mvtnorm_1.1-3
##  [13] lubridate_1.9.2        xml2_1.3.3             cachem_1.0.7
##  [16] knitr_1.42             polyclip_1.10-4        jsonlite_1.8.4
##  [19] broom_1.0.4            GO.db_3.16.0           dbplyr_2.3.2
##  [22] png_0.1-8              graph_1.76.0           ggforce_0.4.1
##  [25] shiny_1.7.4            msigdbr_7.5.1          compiler_4.2.1
##  [28] httr_1.4.5             emmeans_1.8.5          backports_1.4.1
##  [31] Matrix_1.5-3           fastmap_1.1.1          gargle_1.3.0
##  [34] cli_3.6.1              later_1.3.0            tweenr_2.0.2
##  [37] visNetwork_2.1.2       htmltools_0.5.5        tools_4.2.1
##  [40] igraph_1.4.1           GenomeInfoDbData_1.2.9 gtable_0.3.3
##  [43] glue_1.6.2             reshape2_1.4.4         Rcpp_1.0.10
##  [46] Biobase_2.58.0         cellranger_1.1.0       vctrs_0.6.1
##  [49] Biostrings_2.66.0      babelgene_22.9         svglite_2.1.1
##  [52] nlme_3.1-157           xfun_0.38              openxlsx_4.2.5.1
##  [55] rvest_1.0.3            timechange_0.2.0       mime_0.12
##  [58] lifecycle_1.0.3        googlesheets4_1.1.0    zlibbioc_1.44.0
##  [61] MASS_7.3-57            scales_1.2.1           hms_1.1.3
##  [64] promises_1.2.0.1       yaml_2.3.7             memoise_2.0.1
##  [67] stringi_1.7.12         RSQLite_2.3.0          highr_0.10
##  [70] S4Vectors_0.36.2       BiocGenerics_0.44.0    zip_2.2.2
##  [73] GenomeInfoDb_1.34.9    bitops_1.0-7           rlang_1.1.0
##  [76] pkgconfig_2.0.3        systemfonts_1.0.4      evaluate_0.20
##  [79] lattice_0.20-45        labeling_0.4.2         htmlwidgets_1.6.2
##  [82] bit_4.0.5              tidyselect_1.2.0       plyr_1.8.8
##  [85] R6_2.5.1               IRanges_2.32.0         fftw_1.0-7
##  [88] generics_0.1.3         DBI_1.1.3              pillar_1.9.0
##  [91] haven_2.5.2            withr_2.5.0            RCurl_1.98-1.12
##  [94] KEGGREST_1.38.0        reactable_0.4.4        modelr_0.1.11
##  [97] crayon_1.5.2           utf8_1.2.3             tzdb_0.3.0
## [100] rmarkdown_2.21         grid_4.2.1             readxl_1.4.2
## [103] blob_1.2.4             reprex_2.0.2           digest_0.6.31
## [106] webshot_0.5.4          xtable_1.8-4           httpuv_1.6.9
## [109] stats4_4.2.1           munsell_0.5.0          viridisLite_0.4.1
## [112] kableExtra_1.3.4
```

# Step 2.3: RT-qPCR/RNAseq expression measurments for selected genes

Carlos Gallardo & Christian Oertlin

17 April, 2023

```
# Import libraries and helper functions
source("code/helper_functions.R")
library(tidyverse)
library(magrittr)
library(patchwork)
library(RColorBrewer)
library(openxlsx)
library(ComplexHeatmap)

# Colors
colPals <- vector(mode = "list")
colPals$time <- setNames(c("#FBAA3E", "#2C83BE", "#3EB6BD", "#A3D5B3", "#CD71A8"),
                         nm = c("day0", "day7", "day14", "day21", "day28"))
colPals$time_light <- setNames(c("#FDD6A1", "#A2CDE9", "#AFE2E5", "#DDF0E3", "#E8BDD6"),
                               nm = c("day0", "day7", "day14", "day21", "day28"))
colPals$time_dark <- setNames(c("#D87E04", "#174564", "#1F5C60", "#49A065", "#AA3C7E"),
                              nm = c("day0", "day7", "day14", "day21", "day28"))
colPals$inferno <- c("#000004", "#420A68", "#932667", "#DD513A", "#FCA50A", "#FCFFA4")
colPals$blood_cells <- setNames(c("#E54D34","#77A2D5","#B58B80"),
                                nm = c("granulocytes", "lymphocytes", "monocytes"))
colPals$cell_types <- setNames(c("#83D1F6","#FBAA3E","#FCCA7C","#B58B80","#E54D34",
                                 "#B3177E","#9A509F","#77A2D5","#CAC1DD","#36B449","#C1C1C1"),
                               nm = c("B cell", "Macrophage M1", "Macrophage M2",
                                      "Monocyte", "Neutrophil", "NK cell",
                                      "T cell CD4+ (non-regulatory)", "T cell CD8+",
                                      "T cell regulatory (Tregs)", "Myeloid dendritic cell",
                                      "uncharacterized cell"))
colPals$RdBu <- brewer.pal(11, name = "RdBu")
colPals$biotype <- setNames(c("#395982","#49BED9","#18A38A","#36B449","#826F99",
                              "#9852A5","#FBAA3E","#FCCA7C","#FCFFA4","#C1C1C1"),
                            nm = c("protein_coding", "lncRNA", "miRNA", "snoRNA",
                                   "IG_C_gene", "IG_V_gene", "TR_C_gene",
                                   "TR_J_gene", "TR_V_gene", "other"))
colPals$rtqpcr_rnaseq <- setNames(c("#B80D48","#2B6A6C"),
                                  nm = c("rtqpcr", "rnaseq"))
```

## Note:

> Genes for RT-qPCR analysis were selected based on prior interest before RNAseq analysis and are, thus, not entirely driven by it. Housekeeping gene used is HPRT1. HPRT1 has been identified as "stable" gene during simulated microgravity conditions (see Elgindi et al. 2021).

## Load data

```
# RNA-seq
RNAseq <- readRDS(file='data/rnaseq/rnaseq_volunteers_9&10_excl.rds')
DESeq2_DEGs <- readRDS(file='data/rnaseq/DESeq2_DEGs_unfilt_volunteers_9&10_excl.rds')
DESeq2_DEGs_filt <- readRDS(file='data/rnaseq/DESeq2_DEGs_filt_volunteers_9&10_excl.rds')

# RT-qPCR
RTqPCR_ct <- openxlsx::read.xlsx('data/rtqpcr/rtqpcr_cq_values.xlsx',
                              startRow = 2,
                              colNames = T,
                              rowNames = F) %>%
```

```
  dplyr::rename(sample = Sample.ID) %>%
  filter(!grepl('v\\.(9|10)', sample)) %>%
  select_if(~ !any(is.na(.))) %>%
  mutate(sample = RNAseq$filt$design$sample) %>%
  left_join(RNAseq$filt$design)
```

# Housekeeping genes

Cq measurements for V1 and V2 showed high variability indicating experimental difficulties with samples from these volunteers. Also note that samples V9 and V10 are removed according to our exclusion criteria. Overall, RT-qPCR results show high variability with inconsistent trend for some genes between volunteers. However, we identify genes that show a robust profile in all volunteers.

```
ggplot(RTqPCR_ct, aes(x=sample, y=HPRT1, fill=volunteer)) +
  geom_bar(stat='identity', width=0.6, color='black') +
  scale_fill_manual(values = brewer.pal(11, name = "Set3")) +
  xlab('') +
  ylab('Threshold cycle (Ct)') +
  theme_bw() +
  theme(
    axis.text.x.bottom = element_text(angle=90, hjust = 1, vjust = 0.5),
    legend.justification = 'top'
  )
```

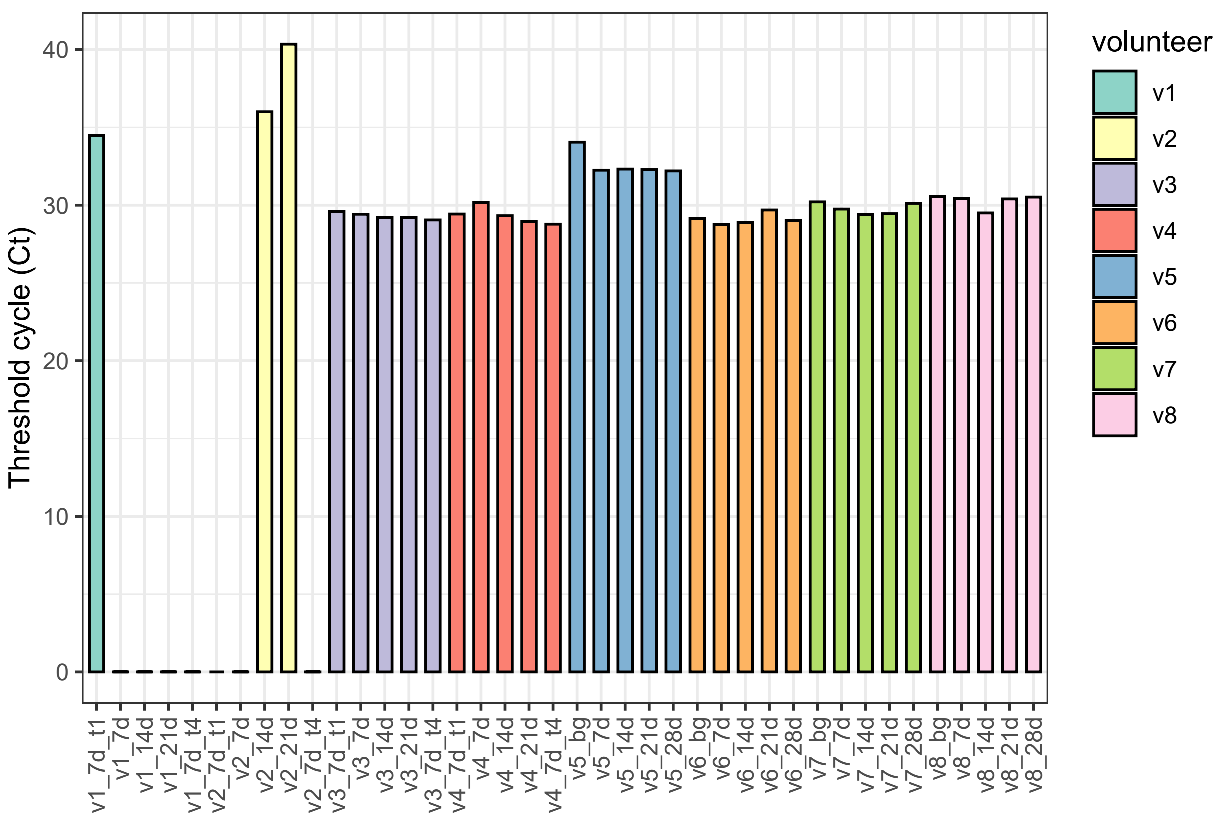


# Normalize expression

```
# normalize to HPRT1
RTqPCR_dct <- RTqPCR_ct %>%
  column_to_rownames(var = 'sample') %>%
  select(-batch,-volunteer,-time) %>%
```

```
  apply(., MARGIN = 2, FUN=function(x) x - .$HPRT1) %>%
  as.data.frame()

RTqPCR_ddct <- RTqPCR_dct %>%
  mutate(volunteer = RTqPCR_ct$volunteer,
         time = RTqPCR_ct$time) %>%
  group_by(volunteer) %>%
  group_split()

# calculate ddct to day0
RTqPCR_ddct <- lapply(RTqPCR_ddct, function(df) {

  volunteer <- df %>% dplyr::pull(volunteer)
  time <- df %>% dplyr::pull(time)

  baseline <- df %>%
    filter(time == 'day0') %>%
    select(-volunteer, -time) %>%
    as.numeric()

  df %>%
    select(-volunteer, -time) %>%
    apply(., MARGIN = 1, FUN=function(x) x - baseline) %>%
    t() %>%
    as.data.frame() %>%
    mutate(volunteer = volunteer,
           time = time)

}) %>% bind_rows()

# calculate expression as 2^-ddCT
RTqPCR_expr <- RTqPCR_ddct
RTqPCR_expr[,1:39] <- 2^(-RTqPCR_expr[,1:39])

# calculate log2 expression
RTqPCR_log2expr <- RTqPCR_expr
RTqPCR_log2expr[,1:39] <- log2(RTqPCR_log2expr[,1:39])
```

# Heatmap with quantifications relative to day0

```
m <- t(RTqPCR_log2expr[,1:39])
colnames(m) <- paste(RTqPCR_log2expr$volunteer, RTqPCR_log2expr$time)

volunteer <- gsub('(v\\d+) .*$', '\\1', colnames(m))
comparison <- gsub('_', ' Vs ', gsub('v\\d+ ', '', colnames(m)))

ha_top <- HeatmapAnnotation(
  Volunteer = factor(volunteer, levels = unique(volunteer)),
  Comparison = factor(comparison, levels = unique(comparison)),
  col = list(
    Volunteer = setNames(brewer.pal(length(unique(volunteer)),"Set3"),
                         nm = unique(volunteer)),
    Comparison = setNames(brewer.pal(length(unique(comparison)),"Dark2"),
                          nm = unique(comparison))
  ),
  annotation_name_gp = gpar(fontface = 'bold'),
  border = T
)

p <- Heatmap(m, name = "log2(2^ddCt)",
             cluster_row_slices = F,
             cluster_rows = T,
             column_title = NULL,
             column_split=factor(volunteer, levels = unique(volunteer)),
             cluster_columns = F,
             col = circlize::colorRamp2(breaks=seq(-2, 2, length.out=21),
                                        colors=colorRampPalette(c("#2166AC", "white", "#B2182B"))(21)),
             top_annotation = ha_top,
             width = unit(170, "mm"),
             show_row_names = T,
             row_title = NULL,
             show_row_dend = T,
             row_dend_width=unit(10, "mm"),
             row_gap = unit(2, "mm"),
             show_column_names = T,
             column_names_gp = gpar(fontsize = 10),
             column_gap = unit(2, "mm"),
```

```
             border = T)

draw(p, merge_legend = T, align_heatmap_legend = "heatmap_top")
```

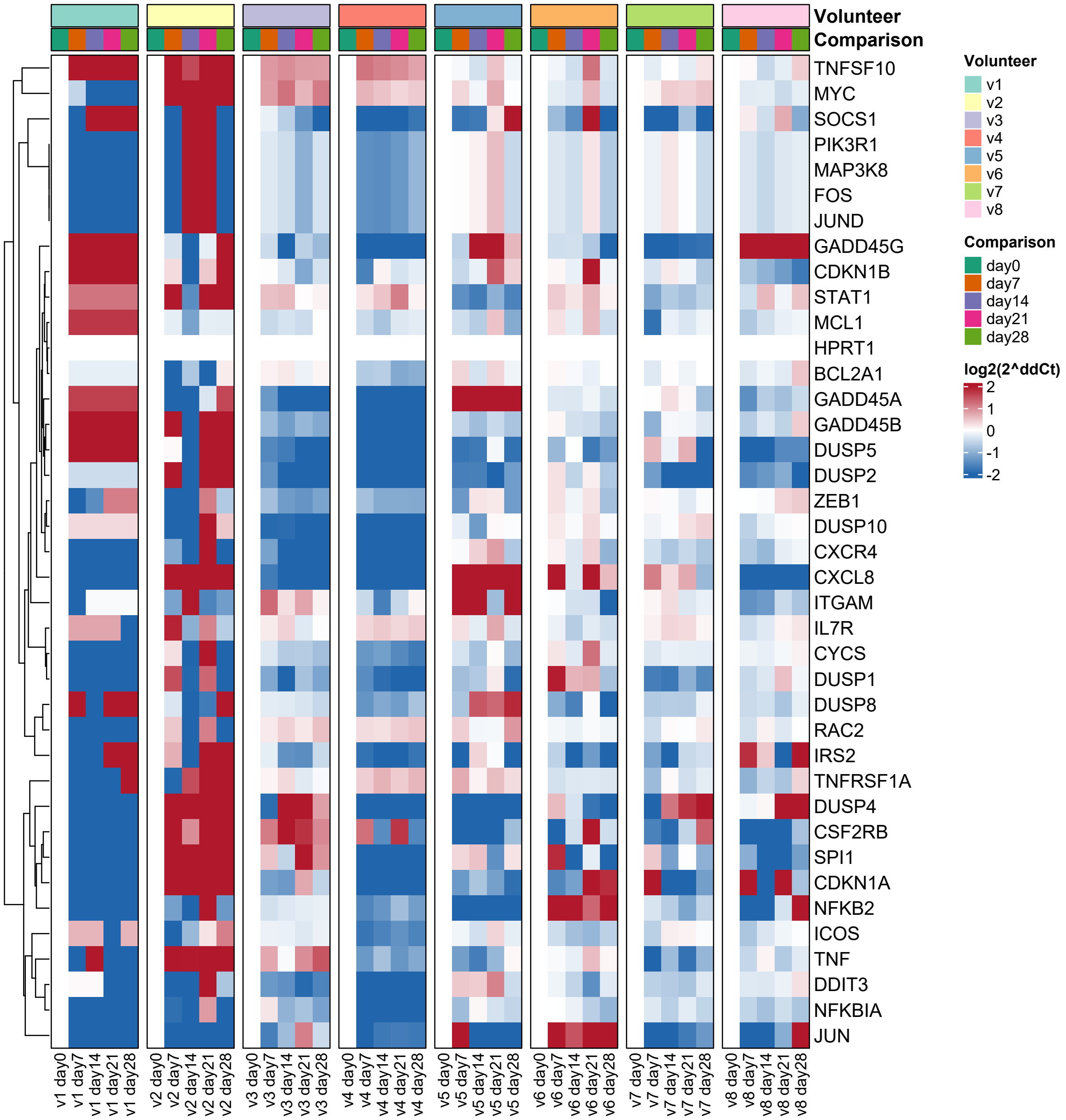


# Compare RT-qPCR and RNAseq

```
# RT-qPCR genes (without housekeeping: HPRT1)
RTqPCR_genes <- colnames(RTqPCR_log2expr[,2:39])

# calculate fold changes per volunteer
RNAseq_log2FC <- calcPerVolunteerFCs(log2(RNAseq$filt$DESeq_norm),
                                     RNAseq$filt$design,
                                     comparison = list(
                                       c("day0","day0"),
                                       c("day7","day0"),
                                       c("day14","day0"),
                                       c("day21","day0"),
```

```
                                      c("day28","day0")
                                    ))
```

```
## [1] "v1" "v2" "v3" "v4" "v5" "v6" "v7" "v8"
## [1] "v1"
## [1] "v2"
## [1] "v3"
## [1] "v4"
## [1] "v5"
## [1] "v6"
## [1] "v7"
## [1] "v8"
```

```
RNAseq_log2FC <- RNAseq_log2FC[!apply(RNAseq_log2FC,1,FUN=function(x) any(is.infinite(x))),]
RNAseq_log2FC[is.na(RNAseq_log2FC)] <- 0
RNAseq_log2FC <- RNAseq_log2FC %>%
  as.data.frame() %>%
  rownames_to_column(var = 'GeneSymbol') %>%
  mutate(GeneSymbol = gsub('(.+)_\\d+','\\1',GeneSymbol)) %>%
  filter(GeneSymbol %in% RTqPCR_genes) %>%
  filter(!duplicated(GeneSymbol))

# shared genes
shared_genes <- RNAseq_log2FC$GeneSymbol

# long format data frames
RTqPCR_log2expr_long <- RTqPCR_log2expr %>%
  select(shared_genes, 'volunteer', 'time') %>%
  pivot_longer(shared_genes, names_to = 'GeneSymbol', values_to = 'log2FC') %>%
  mutate(type = 'rtqpcr',
         volunteer = as.character(volunteer),
         time = as.character(time))

RNAseq_log2FC_long <- RNAseq_log2FC %>%
  column_to_rownames(var = 'GeneSymbol') %>%
  t() %>%
  as.data.frame() %>%
  mutate(volunteer = gsub('(v\\d+) .*','\\1',rownames(.)),
         time = gsub('v\\d+ (day\\d+)_day0','\\1',rownames(.))) %>%
  select(shared_genes, 'volunteer', 'time') %>%
  pivot_longer(shared_genes, names_to = 'GeneSymbol', values_to = 'log2FC') %>%
  mutate(type = 'rnaseq')

# long format gene mean expression per time point
RTqPCR_log2expr_long_mean <- RTqPCR_log2expr_long %>%
  filter(!volunteer %in% c('v1','v2')) %>%
  group_by(GeneSymbol, time) %>%
  summarise(log2FC = mean(log2FC)) %>%
  arrange(factor(GeneSymbol, levels = shared_genes),
          factor(time, levels = names(colPals$time))) %>%
  mutate(type = 'rtqpcr')

RNAseq_log2FC_long_mean <- RNAseq_log2FC_long %>%
  filter(!volunteer %in% c('v1','v2')) %>%
  group_by(GeneSymbol, time) %>%
  summarise(log2FC = mean(log2FC)) %>%
  arrange(factor(GeneSymbol, levels = shared_genes),
          factor(time, levels = names(colPals$time))) %>%
  mutate(type = 'rnaseq')
```

## Correlation

```
# we exclude V1 and V2 due to high variability and inflated expression measurements
volunteer_order <- paste('v', seq(3,8), sep = '')
time_order <- paste('day', c(0,7,14,21,28), sep = '')

# calculate correlations
res_cor <- lapply(setNames(shared_genes, shared_genes), function(x) {

  rtqpcr_log2FC <- RTqPCR_log2expr_long %>%
    filter(volunteer %in% volunteer_order & GeneSymbol == x) %>%
    arrange(factor(volunteer, levels = volunteer_order),
            factor(time, levels = time_order)) %>%
    pull(log2FC)

  rnaseq_log2FC <- RNAseq_log2FC_long %>%
    filter(volunteer %in% volunteer_order & GeneSymbol == x) %>%
    arrange(factor(volunteer, levels = volunteer_order),
            factor(time, levels = time_order)) %>%
```

```
    pull(log2FC)

  rtqpcr_rnaseq_cor <- cor(rtqpcr_log2FC, rnaseq_log2FC, method = "pearson")

  data.frame(GeneSymbol = x,
             r = rtqpcr_rnaseq_cor)

}) %>% bind_rows()


# calculate correlations per volunteer
gene_volunteer_comb <- expand.grid(list(GeneSymbol = shared_genes,
                                        volunteer = unique(volunteer))) %>%
  filter(!duplicated(.))
gene_volunteer_comb <- split(gene_volunteer_comb, seq(nrow(gene_volunteer_comb)))

res_cor_by_volunteer <- lapply(gene_volunteer_comb, function(x) {

  rtqpcr_log2FC <- RTqPCR_log2expr_long %>%
    filter(volunteer == x$volunteer & GeneSymbol == x$GeneSymbol) %>%
    arrange(factor(time, levels = time_order)) %>%
    pull(log2FC)

  rnaseq_log2FC <- RNAseq_log2FC_long %>%
    filter(volunteer == x$volunteer & GeneSymbol == x$GeneSymbol) %>%
    arrange(factor(time, levels = time_order)) %>%
    pull(log2FC)

  rtqpcr_rnaseq_cor <- cor(rtqpcr_log2FC, rnaseq_log2FC, method = "pearson")

  data.frame(volunteer = x$volunteer,
             GeneSymbol = x$GeneSymbol,
             r = rtqpcr_rnaseq_cor)

}) %>% bind_rows()

res_cor_by_volunteer_mean <- res_cor %>%
  group_by(GeneSymbol) %>%
  summarise(r_mean = mean(r))
```

```
selected_genes <- c('DUSP1','DUSP2','DUSP4','DUSP10','JUN','JUND','FOS','SOCS1')

df <- bind_rows(RTqPCR_log2expr_long,
                RNAseq_log2FC_long) %>%
  filter(!volunteer %in% c('v1','v2')) %>%
  mutate(r = recode(GeneSymbol, !!!setNames(res_cor$r,
                                            nm = res_cor$GeneSymbol))) %>%
  filter(GeneSymbol %in% selected_genes) %>%
  mutate(GeneSymbol = factor(GeneSymbol, levels = selected_genes),
         time = factor(time, levels = names(colPals$time)),
         type = factor(type, levels = names(colPals$rtqpcr_rnaseq)))

df2 <- bind_rows(RTqPCR_log2expr_long_mean,
                 RNAseq_log2FC_long_mean) %>%
  mutate(r = recode(GeneSymbol, !!!setNames(res_cor$r,
                                            nm = res_cor$GeneSymbol))) %>%
  filter(GeneSymbol %in% selected_genes) %>%
  mutate(GeneSymbol = factor(GeneSymbol, levels = selected_genes),
         time = factor(time, levels = names(colPals$time)),
         type = factor(type, levels = names(colPals$rtqpcr_rnaseq)))

ggplot(df, aes(x=time, y=log2FC, color=type)) +
  geom_hline(yintercept = 0, color='grey80', size=1) +
  geom_point(shape=16, size=3, stroke=0, alpha=0.4) +
  facet_wrap(~GeneSymbol, ncol=4, scales = 'free') +
  geom_line(data = df2, aes(x=time, y=log2FC, group=type, color=type), size = 1.25) +
  geom_point(data = df2, aes(x=time, y=log2FC, group=type, fill=time), shape=21, size=3.5, stroke=1.25) +
  geom_text(aes(label=paste('r =',round(r,2)), group=r),x = -Inf, y = Inf, hjust = -0.2, vjust = 1.8, inherit.aes = FALSE) +
  scale_y_continuous(expand = expansion(mult = c(.1, .25))) +
  xlab('') +
  ylab('Relative quantification (log2FC)') +
  scale_color_manual(values = c(colPals$time, colPals$rtqpcr_rnaseq)) +
  scale_fill_manual(values = c(colPals$time, colPals$rtqpcr_rnaseq)) +
  theme_bw() +
  theme(
    text = element_text(family = 'Arial', size = 14),
    axis.text.x.bottom = element_text(angle = 90, hjust = 1, vjust = 0.5),
    panel.border = element_rect(color = "black", fill = NA, size = 2),
    axis.ticks = element_line(color = "black", size = 1.25),
    axis.ticks.length = unit(1.5, 'mm'),
```

```
    panel.grid.major.x = element_blank(),
    panel.grid.minor.x = element_blank(),
    panel.grid.major.y = element_blank(),
    panel.grid.minor.y = element_blank(),
    legend.position = 'top',
    strip.background = element_blank(),
    strip.text = element_text(face = "bold.italic"),
    strip.text.y = element_text(angle = 90)
  )
```

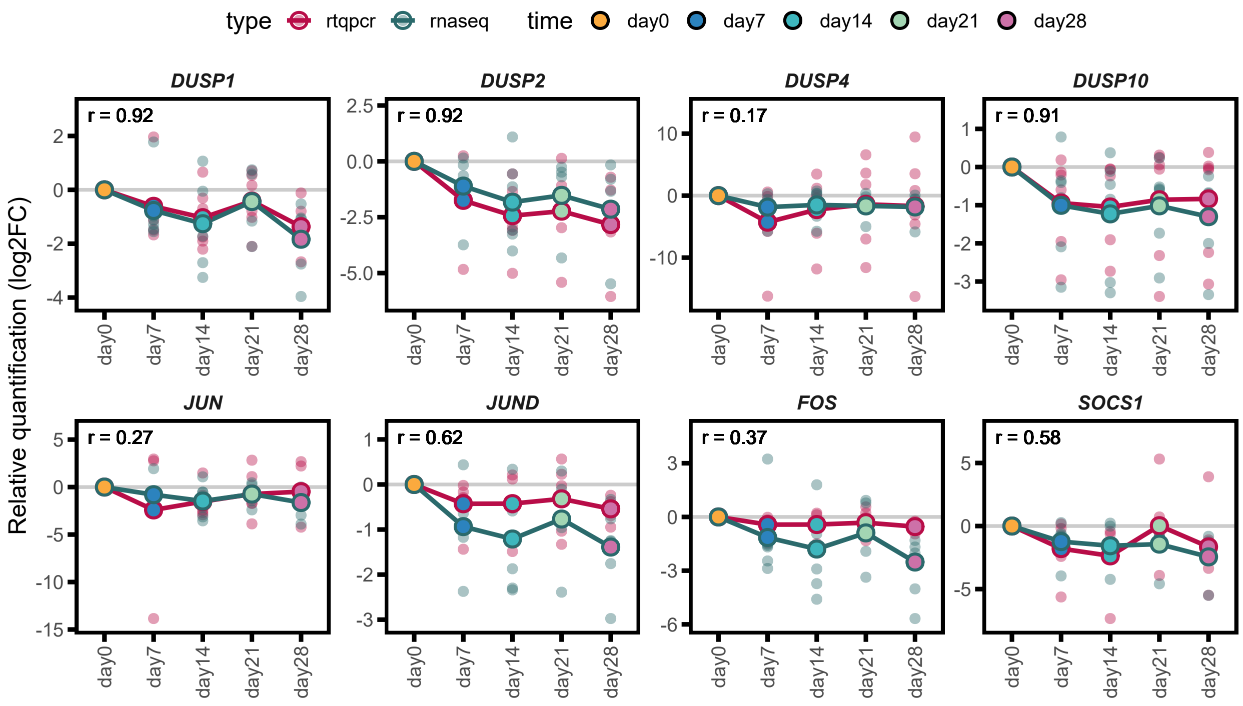


```
ggsave(filename = "plots/fig2B_RTqPCR_RNAseq_selected_log2FC.pdf", width = 10, height = 6, units = "in", dpi = 300, device = cairo_pdf)
```

# SessionInfo

```
sessionInfo()
```

```
## R version 4.2.1 (2022-06-23 ucrt)
## Platform: x86_64-w64-mingw32/x64 (64-bit)
## Running under: Windows 10 x64 (build 19044)
##
## Matrix products: default
##
## locale:
## [1] LC_COLLATE=English_United States.utf8
## [2] LC_CTYPE=English_United States.utf8
## [3] LC_MONETARY=English_United States.utf8
## [4] LC_NUMERIC=C
## [5] LC_TIME=English_United States.utf8
##
## attached base packages:
## [1] grid      stats     graphics  grDevices utils     datasets  methods
## [8] base
##
## other attached packages:
##  [1] ComplexHeatmap_2.14.0 openxlsx_4.2.5.1      RColorBrewer_1.1-3
##  [4] patchwork_1.1.2       magrittr_2.0.3        forcats_1.0.0
##  [7] stringr_1.5.0         dplyr_1.1.1           purrr_1.0.1
## [10] readr_2.1.4           tidyr_1.3.0           tibble_3.2.1
## [13] ggplot2_3.4.2         tidyverse_1.3.2
##
## loaded via a namespace (and not attached):
```

```
##  [1] httr_1.4.5          jsonlite_1.8.4      foreach_1.5.2
##  [4] modelr_0.1.11       highr_0.10          stats4_4.2.1
##  [7] googlesheets4_1.1.0 cellranger_1.1.0    yaml_2.3.7
## [10] pillar_1.9.0        backports_1.4.1     glue_1.6.2
## [13] digest_0.6.31       rvest_1.0.3         colorspace_2.1-0
## [16] htmltools_0.5.5     pkgconfig_2.0.3     GetoptLong_1.0.5
## [19] broom_1.0.4         haven_2.5.2         scales_1.2.1
## [22] tzdb_0.3.0          timechange_0.2.0    googledrive_2.1.0
## [25] farver_2.1.1        generics_0.1.3      IRanges_2.32.0
## [28] withr_2.5.0         BiocGenerics_0.44.0 cli_3.6.1
## [31] crayon_1.5.2        readxl_1.4.2        evaluate_0.20
## [34] fs_1.6.1            fansi_1.0.4         doParallel_1.0.17
## [37] xml2_1.3.3          tools_4.2.1         hms_1.1.3
## [40] GlobalOptions_0.1.2 gargle_1.3.0        lifecycle_1.0.3
## [43] matrixStats_0.63.0  S4Vectors_0.36.2    munsell_0.5.0
## [46] reprex_2.0.2        cluster_2.1.3       zip_2.2.2
## [49] compiler_4.2.1      rlang_1.1.0         iterators_1.0.14
## [52] rstudioapi_0.14     circlize_0.4.15     rjson_0.2.21
## [55] labeling_0.4.2      rmarkdown_2.21      gtable_0.3.3
## [58] codetools_0.2-18    DBI_1.1.3           R6_2.5.1
## [61] lubridate_1.9.2     knitr_1.42          fastmap_1.1.1
## [64] utf8_1.2.3          clue_0.3-64         shape_1.4.6
## [67] stringi_1.7.12      parallel_4.2.1      Rcpp_1.0.10
## [70] vctrs_0.6.1         png_0.1-8           dbplyr_2.3.2
## [73] tidyselect_1.2.0    xfun_0.38
```

# Step 3.2: FACS analysis

Carlos Gallardo & Christian Oertlin

17 April, 2023

```
# Import libraries and helper functions
source("code/helper_functions.R")
library(tidyverse)
library(magrittr)
library(patchwork)
library(RColorBrewer)
library(openxlsx)

# Colors
colPals <- vector(mode = "list")
colPals$time <- setNames(c("#FBAA3E", "#2C83BE", "#3EB6BD", "#A3D5B3", "#CD71A8"),
                         nm = c("day0", "day7", "day14", "day21", "day28"))
colPals$time_light <- setNames(c("#FDD6A1", "#A2CDE9", "#AFE2E5", "#DDF0E3", "#E8BDD6"),
                               nm = c("day0", "day7", "day14", "day21", "day28"))
colPals$time_dark <- setNames(c("#D87E04", "#174564", "#1F5C60", "#49A065", "#AA3C7E"),
                              nm = c("day0", "day7", "day14", "day21", "day28"))
colPals$inferno <- c("#000004", "#420A68", "#932667", "#DD513A", "#FCA50A", "#FCFFA4")
colPals$blood_cells <- setNames(c("#E54D34","#77A2D5","#B58B80"),
                                nm = c("granulocytes", "lymphocytes", "monocytes"))
colPals$cell_types <- setNames(c("#83D1F6","#FBAA3E","#FCCA7C","#B58B80","#E54D34",
                                 "#B3177E","#9A509F","#77A2D5","#CAC1DD","#36B449","#C1C1C1"),
                               nm = c("B cell", "Macrophage M1", "Macrophage M2",
                                      "Monocyte", "Neutrophil", "NK cell",
                                      "T cell CD4+ (non-regulatory)", "T cell CD8+",
                                      "T cell regulatory (Tregs)", "Myeloid dendritic cell",
                                      "uncharacterized cell"))
colPals$RdBu <- brewer.pal(11, name = "RdBu")
colPals$biotype <- setNames(c("#395982","#49BED9","#18A38A","#36B449","#826F99",
                              "#9852A5","#FBAA3E","#FCCA7C","#FCFFA4","#C1C1C1"),
                            nm = c("protein_coding", "lncRNA", "miRNA", "snoRNA",
                                   "IG_C_gene", "IG_V_gene", "TR_C_gene",
                                   "TR_J_gene", "TR_V_gene", "other"))
colPals$rtqpcr_rnaseq <- setNames(c("#B80D48","#2B6A6C"),
                                  nm = c("rtqpcr", "rnaseq"))
```

## Load data

```
# FACS data
filenames <- list.files(path= "data/facs", pattern = "^facs_",full.names = T)

facs_data <- lapply(filenames, function(x) {

  valunteer <- gsub('.*(v\\d+).*', '\\1', x)

  df <- read.table(file = x,
                   stringsAsFactors = F,
                   sep = ",",
                   header = T,
                   fill = T,
                   quote = "")

  colnames(df) <- c('time', 'gate', 'perc_among_CD3', 'perc_among_lymphocytes',
                    'MFI', 'MFI_CD25_peak_vs_MFI_contr_lymph_peak')

  df <- df %>%
    add_column(volunteer = valunteer, .before = 'time') %>%
    mutate(perc_among_CD3 = as.numeric(perc_among_CD3),
           perc_among_lymphocytes = as.numeric(perc_among_lymphocytes),
           MFI = as.numeric(MFI),
           MFI_CD25_peak_vs_MFI_contr_lymph_peak = as.numeric(MFI_CD25_peak_vs_MFI_contr_lymph_peak))
```

```
  df

}) %>% bind_rows()
```

# Mean MFI of CD25 over control

```
# mean fluorescence intensity (MFI) at different time points
df <- facs_data %>%
  filter(volunteer %in% paste0('v', seq(1,8))) %>%
  filter(!is.na(MFI_CD25_peak_vs_MFI_contr_lymph_peak)) %>%
  filter(MFI_CD25_peak_vs_MFI_contr_lymph_peak != 0) %>%
  mutate(time = recode(time, !!!setNames(names(colPals$time),
                                        nm = c('-7d','7d','14d','21d','+7d')))) %>%
  filter(time %in% names(colPals$time)) %>%
  mutate(time = factor(time, levels = names(colPals$time)))

df2 <- df %>%
  group_by(time) %>%
  select(time, MFI_CD25_peak_vs_MFI_contr_lymph_peak) %>%
  summarize_each(dplyr::funs(mean, sd, se=sd(.)/sqrt(n())), MFI_CD25_peak_vs_MFI_contr_lymph_peak)

ggplot() +
  geom_point(data = df, aes(x=time, y=MFI_CD25_peak_vs_MFI_contr_lymph_peak, color=time),
             shape=16, size=4, stroke=0, alpha=0.5) +
  geom_errorbar(data=df2, aes(x=time, y=mean, ymin=mean-se*1.96, ymax=mean+se*1.96), width=.2, lwd=1.5) +
  # geom_line(data = df2, aes(x=time, y=mean, group=1), color='black', size = 1.5) +
  geom_point(data = df2, aes(x=time, y=mean, fill=time), color="black", shape=21, size=5, stroke=2, alpha=1) +
  scale_y_continuous(expand = expansion(mult = c(.05, .05))) +
  scale_color_manual(values = colPals$time) +
  scale_fill_manual(values = colPals$time) +
  xlab('') +
  ylab('MFI CD25 / MFI Control') +
  theme_bw(base_size = 20) +
  theme(panel.grid.major.y = element_line(color = "grey80", linetype = "solid", size = 1),
        panel.grid.minor.y = element_blank(),
        panel.grid.major.x = element_blank(),
        panel.grid.minor.x = element_blank(),
        panel.border = element_rect(color = "black", fill = NA, size = 2),
        axis.ticks = element_line(color = "black", size = 1.25),
        legend.position = "none"
  )
```

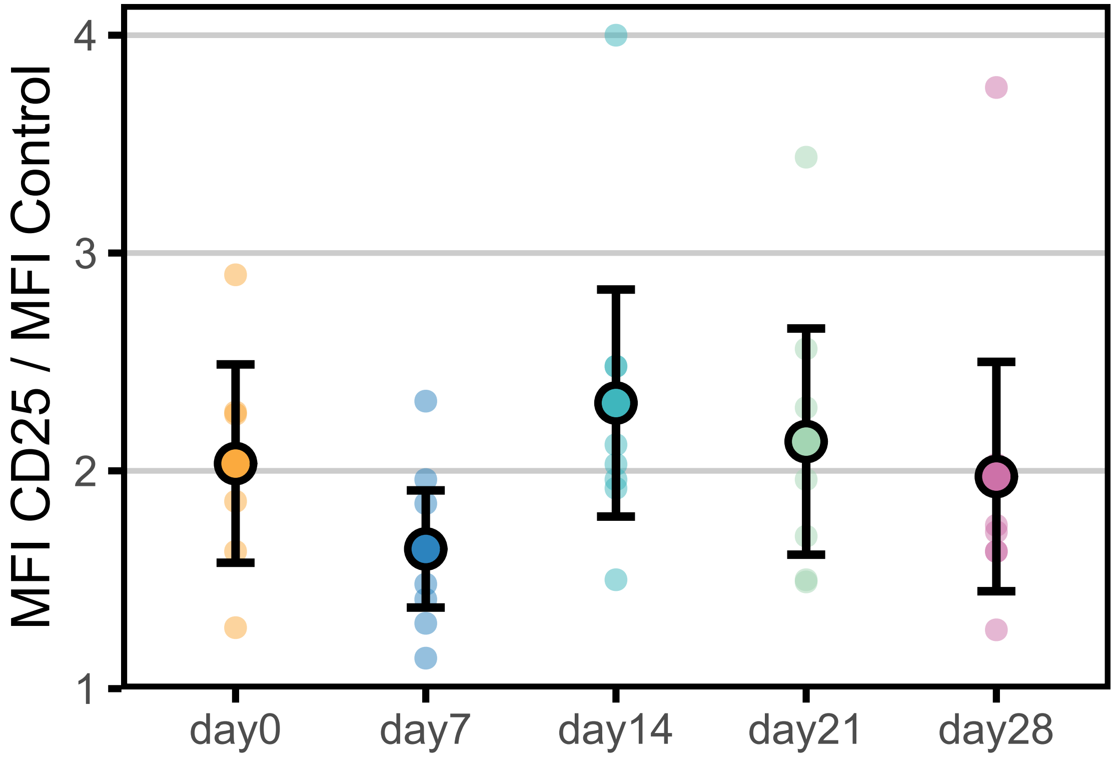


```
ggsave(filename = "plots/fig3C_FACS_MFI_CD25_over_control.pdf", width = 6, height = 4.5, units = "in", dpi = 300, device = cairo_pdf)
```

```
# perform paired t-tests
tests_comb <- expand.grid(time1 = names(colPals$time), time2 = names(colPals$time)) %>%
  filter(time1 != time2) %>%
  t() %>%
  as.data.frame()

res_t_test <- lapply(tests_comb, function(x) {
  time1 <- x[1]
  time2 <- x[2]

  group1 <- df %>%
    filter(time == time1) %>%
    pull(MFI_CD25_peak_vs_MFI_contr_lymph_peak)

  id1 <- df %>%
    filter(time == time1) %>%
    pull(volunteer)

  group2 <- df %>%
    filter(time == time2) %>%
    pull(MFI_CD25_peak_vs_MFI_contr_lymph_peak)

  id2 <- df %>%
    filter(time == time2) %>%
    pull(volunteer)

  test_table <- data.frame(volunteer = unique(df$volunteer)) %>%
    mutate(group1 = recode(volunteer, !!!setNames(group1,
                                                  id1)),
           group2 = recode(volunteer, !!!setNames(group2,
                                                  id2))) %>%
    mutate(group1 = as.numeric(group1),
           group2 = as.numeric(group2))

  c(time_1 = time1,
    time_2 = time2,
    p_val = t.test(test_table$group1, test_table$group2, paired = T)$p.value)
```

```r
}) %>% bind_rows() %>%
  mutate(p_adj = p.adjust(p_val, method = 'BH'))

res_t_test
```

```
## # A tibble: 20 x 4
##    time_1 time_2 p_val              p_adj
##    <chr>  <chr>  <chr>              <dbl>
##  1 day7   day0   0.102276964699089  0.311
##  2 day14  day0   0.182054125417157  0.311
##  3 day21  day0   0.186706873102431  0.311
##  4 day28  day0   0.804372845689488  0.804
##  5 day0   day7   0.102276964699089  0.311
##  6 day14  day7   0.0761883643823477 0.311
##  7 day21  day7   0.163774733496774  0.311
##  8 day28  day7   0.366268787921531  0.458
##  9 day0   day14  0.182054125417157  0.311
## 10 day7   day14  0.0761883643823477 0.311
## 11 day21  day14  0.271483492878872  0.388
## 12 day28  day14  0.0517690280085766 0.311
## 13 day0   day21  0.186706873102431  0.311
## 14 day7   day21  0.163774733496774  0.311
## 15 day14  day21  0.271483492878872  0.388
## 16 day28  day21  0.729468613696315  0.804
## 17 day0   day28  0.804372845689488  0.804
## 18 day7   day28  0.366268787921531  0.458
## 19 day14  day28  0.0517690280085766 0.311
## 20 day21  day28  0.729468613696315  0.804
```

# SessionInfo

```r
sessionInfo()
```

```
## R version 4.2.1 (2022-06-23 ucrt)
## Platform: x86_64-w64-mingw32/x64 (64-bit)
## Running under: Windows 10 x64 (build 19044)
##
## Matrix products: default
##
## locale:
## [1] LC_COLLATE=English_United States.utf8
## [2] LC_CTYPE=English_United States.utf8
## [3] LC_MONETARY=English_United States.utf8
## [4] LC_NUMERIC=C
## [5] LC_TIME=English_United States.utf8
##
## attached base packages:
## [1] stats     graphics  grDevices utils     datasets  methods   base
##
## other attached packages:
##  [1] openxlsx_4.2.5.1   RColorBrewer_1.1-3 patchwork_1.1.2    magrittr_2.0.3
##  [5] forcats_1.0.0      stringr_1.5.0      dplyr_1.1.1        purrr_1.0.1
##  [9] readr_2.1.4        tidyr_1.3.0        tibble_3.2.1       ggplot2_3.4.2
## [13] tidyverse_1.3.2
##
## loaded via a namespace (and not attached):
##  [1] tidyselect_1.2.0    xfun_0.38           haven_2.5.2
##  [4] gargle_1.3.0        colorspace_2.1-0    vctrs_0.6.1
##  [7] generics_0.1.3      htmltools_0.5.5     yaml_2.3.7
## [10] utf8_1.2.3          rlang_1.1.0         pillar_1.9.0
## [13] glue_1.6.2          withr_2.5.0         DBI_1.1.3
## [16] dbplyr_2.3.2        modelr_0.1.11       readxl_1.4.2
## [19] lifecycle_1.0.3     munsell_0.5.0       gtable_0.3.3
## [22] cellranger_1.1.0    zip_2.2.2           rvest_1.0.3
## [25] evaluate_0.20       labeling_0.4.2      knitr_1.42
## [28] tzdb_0.3.0          fastmap_1.1.1       fansi_1.0.4
## [31] highr_0.10          Rcpp_1.0.10         broom_1.0.4
## [34] scales_1.2.1        backports_1.4.1     googlesheets4_1.1.0
## [37] jsonlite_1.8.4      farver_2.1.1        fs_1.6.1
## [40] hms_1.1.3           digest_0.6.31       stringi_1.7.12
## [43] grid_4.2.1          cli_3.6.1           tools_4.2.1
## [46] crayon_1.5.2        pkgconfig_2.0.3     xml2_1.3.3
## [49] reprex_2.0.2        googledrive_2.1.0   lubridate_1.9.2
## [52] timechange_0.2.0    rmarkdown_2.21      httr_1.4.5
## [55] rstudioapi_0.14     R6_2.5.1            compiler_4.2.1
```

# Step 3.1: Comparison of gene expression changes to NASA twin study

Carlos Gallardo & Christian Oertlin

17 April, 2023

```r
# Import libraries and helper functions
source("code/helper_functions.R")
library(tidyverse)
library(magrittr)
library(patchwork)
library(RColorBrewer)
library(openxlsx)
library(ComplexHeatmap)

# Colors
colPals <- vector(mode = "list")
colPals$time <- setNames(c("#FBAA3E", "#2C83BE", "#3EB6BD", "#A3D5B3", "#CD71A8"),
                        nm = c("day0", "day7", "day14", "day21", "day28"))
colPals$time_light <- setNames(c("#FDD6A1", "#A2CDE9", "#AFE2E5", "#DDF0E3", "#E8BDD6"),
                              nm = c("day0", "day7", "day14", "day21", "day28"))
colPals$time_dark <- setNames(c("#D87E04", "#174564", "#1F5C60", "#49A065", "#AA3C7E"),
                             nm = c("day0", "day7", "day14", "day21", "day28"))
colPals$inferno <- c("#000004", "#420A68", "#932667", "#DD513A", "#FCA50A", "#FCFFA4")
colPals$blood_cells <- setNames(c("#E54D34","#77A2D5","#B58B80"),
                               nm = c("granulocytes", "lymphocytes", "monocytes"))
colPals$cell_types <- setNames(c("#83D1F6","#FBAA3E","#FCCA7C","#B58B80","#E54D34",
                                "#B3177E","#9A509F","#77A2D5","#CAC1DD","#36B449","#C1C1C1"),
                              nm = c("B cell", "Macrophage M1", "Macrophage M2",
                                     "Monocyte", "Neutrophil", "NK cell",
                                     "T cell CD4+ (non-regulatory)", "T cell CD8+",
                                     "T cell regulatory (Tregs)", "Myeloid dendritic cell",
                                     "uncharacterized cell"))
colPals$RdBu <- brewer.pal(11, name = "RdBu")
colPals$biotype <- setNames(c("#395982","#49BED9","#18A38A","#36B449","#826F99",
                             "#9852A5","#FBAA3E","#FCCA7C","#FCFFA4","#C1C1C1"),
                           nm = c("protein_coding", "lncRNA", "miRNA", "snoRNA",
                                  "IG_C_gene", "IG_V_gene", "TR_C_gene",
                                  "TR_J_gene", "TR_V_gene", "other"))
colPals$rtqpcr_rnaseq <- setNames(c("#B80D48","#2B6A6C"),
                                 nm = c("rtqpcr", "rnaseq"))
```

# Load data

```r
# RNA-seq
RNAseq <- readRDS(file='data/rnaseq/rnaseq_volunteers_9&10_excl.rds')
DESeq2_DEGs <- readRDS(file='data/rnaseq/DESeq2_DEGs_unfilt_volunteers_9&10_excl.rds')
DESeq2_DEGs_filt <- readRDS(file='data/rnaseq/DESeq2_DEGs_filt_volunteers_9&10_excl.rds')
DEGs_clusters <- readRDS(file='data/rnaseq/DEGs_kmeans_clusters.rds')

# DEGs from comparisons in NASA twin study
NASA_twin <- read.table(file = 'data/resources/nasa_twin_study_DEGs.csv',
                        skip = 1,
                        stringsAsFactors = FALSE,
                        sep = "\t",
                        header = TRUE,
                        fill = FALSE,
                        quote = "")
```

# Heatmap comparing DI changes to NASA study

```
# T cell DEG clusters in dry immersion
# filter genes without gene symbol (ENSID) and also duplicated entries for a gene symbol
DEG_clusters_FC_filt <- DEGs_clusters$DEGs_clusters_FC %>%
  filter(!grepl('^ENS', GeneSymbol)) %>%
  mutate(GeneSymbol = gsub('(.+)_\\d+','\\1',GeneSymbol)) %>%
  filter(!duplicated(GeneSymbol))


# relevant comparisons in NASA study for CD4+ and CD8+ T cells
# we average log2FC values of the different RNA-seq experiment types
NASA_twin_selected <- NASA_twin %>%
  filter(CellType %in% c('CD4','CD8')) %>%
  filter(Coefficient %in% c('Post-flight vs Pre-flight',
                            'In+Post-flight vs Pre-flight',
                            'Post-flight vs Pre+In-flight')) %>%
  dplyr::rename(GeneSymbol = Gene, log2FC = log2.Fold.Change) %>%
  group_by(CellType, Coefficient, GeneSymbol) %>%
  summarise(log2FC = mean(log2FC)) %>%
  unite(col = 'condition', CellType, Coefficient, sep = '_', remove = T) %>%
  pivot_wider(names_from = condition, values_from = log2FC, names_prefix = '')

# combine t cell dry immersion data with NASA study
DEGs_DI_NASA_combined <- DEG_clusters_FC_filt %>%
  left_join(NASA_twin_selected, by = 'GeneSymbol') %>%
  select(-Cluster) %>%
  relocate(Biotype, .after = last_col())
```

```
# relevant genes to annotate
mark.genes <- c("IL7R","ETS1","GATA3","TCF7","TCF1","BCL11B",
                "SPI1","HES1","BCL11A","TCF12","BCL6","BCL2",
                "IER2",
                "CD27", # activation marker
                "CD3G",
                "CD69", # Early activation marker
                "CCR10","CCR2","CCR5",
                "CD160", # inhibits t cell activation
                "CD79B",
                "CD82","CD83",
                "RORA","RORC",
                "FOXP3",
                "CTLA4",
                "PDCD1",
                "CXCR4",
                "CXCL16",
                "ICOS",
                "IL2RA","IL2RB",
                "IL10RA",
                "EOMES",
                "SOCS1","SOCS3",
                "RHOH",
                "DUSP1","DUSP2","DUSP4","DUSP8","DUSP10",
                "FOS","FOSL2",
                "JUN","JUNB","JUND",
                "STAT5",
                "PRKCA",
                "ATF2"
)

# genes changed in at least 1 of the NASA comparisons
detected_NASA_comparisons <- DEGs_DI_NASA_combined %>%
  column_to_rownames(var = 'GeneSymbol') %>%
  select(`CD4_In+Post-flight vs Pre-flight`:`CD8_Post-flight vs Pre-flight`) %>%
  mutate_all(~replace(., is.na(.), 0)) %>%
  mutate_all(abs) %>%
  mutate(sum_change = rowSums(.)) %>%
  filter(sum_change > 0) %>%
  rownames_to_column(var = 'GeneSymbol') %>%
  pull(GeneSymbol)

mark.genes <- intersect(mark.genes, detected_NASA_comparisons)


m <- DEGs_DI_NASA_combined %>%
  column_to_rownames(var = 'GeneSymbol') %>%
  select(day0Vsday0:`CD8_Post-flight vs Pre-flight`)

set.seed(25)
clust_mat <- m
```

```r
clust_mat[is.na(clust_mat)] <- 0
clust = kmeans(clust_mat, centers = 3)

cell_type <- c(rep('CD3+',5), rep('CD4+',3), rep('CD8+',3))

comparison <- gsub('Vs', ' Vs ', colnames(m))
comparison <- gsub(' vs ', ' Vs ', comparison)
comparison <- gsub('CD\\d+_', '', comparison)

lbls <- c("1" = paste0("Cluster 1 (n=",sum(clust$cluster=='1'),")"),
          "2" = paste0("Cluster 2 (n=",sum(clust$cluster=='2'),")"),
          "3" = paste0("Cluster 3 (n=",sum(clust$cluster=='3'),")"))

ha_top <- HeatmapAnnotation(
  Cell_type = factor(cell_type, levels = unique(cell_type)),
  Comparison = factor(comparison, levels = unique(comparison)),
  col = list(
    Cell_type = setNames(c('#E54D34','#9A509F','#77A2D5'),
                         nm = unique(cell_type)),
    Comparison = setNames(c(colPals$time,'#826F99','#18A38A','#395982'),
                         nm = unique(comparison))
  ),
  annotation_name_gp = gpar(fontface = 'bold'),
  border = T
)

ha_right <- rowAnnotation(
  mark = anno_mark(at=which(rownames(m) %in% mark.genes),
                   labels = rownames(m)[which(rownames(m) %in% mark.genes)],
                   padding = unit(1,"mm"),
                   labels_gp = gpar(fontface = 'italic'))
)

p <- Heatmap(m, name = "log2FC",
             row_split = clust$cluster, cluster_row_slices = F, cluster_rows = T,
             column_title = NULL, column_split=factor(cell_type, levels = unique(cell_type)), cluster_columns = F,
             col = circlize::colorRamp2(breaks=seq(-2, 2, length.out=21),
                                        colors=colorRampPalette(rev(colPals$RdBu))(21)),
             top_annotation = ha_top,
             right_annotation = ha_right,
             width = unit(110, "mm"),
             na_col = "grey80",
             show_row_names = F,
             row_title = lbls,
             show_row_dend = T, row_dend_width=unit(10, "mm"), row_gap = unit(2, "mm"),
             show_column_names = T, column_names_gp = gpar(fontsize = 10), column_gap = unit(2, "mm"),
             border = T)

draw(p, merge_legend = T, align_heatmap_legend = "heatmap_top")
```

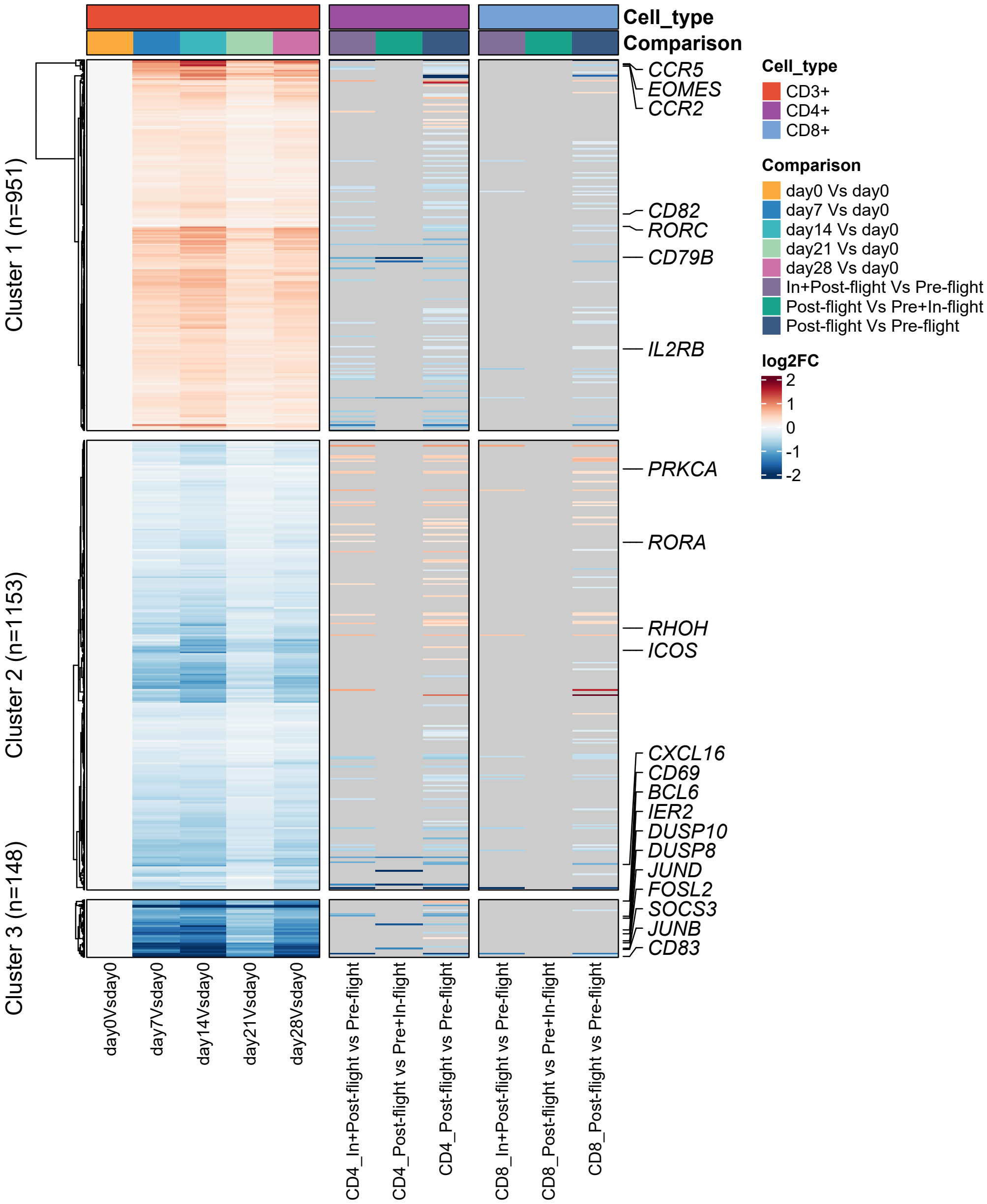


```
pdf("plots/figS9_DEG_NASA_twin_study_comparison.pdf", width = 10, height = 10)
draw(p, merge_legend = T, align_heatmap_legend = "heatmap_top")
dev.off()
```

```
## cairo_pdf
##         2
```

# Heatmap of heavily downregulated genes (cluster 3)

```
# add cluster information
DEGs_DI_NASA_combined <- DEGs_DI_NASA_combined %>%
  add_column(Cluster = recode(.$GeneSymbol, !!!clust$cluster), .after = 'GeneSymbol') %>%
  arrange(Cluster)

# subset cluster 3
DEGs_DI_NASA_combined_cl3 <- DEGs_DI_NASA_combined %>%
  filter(Cluster == '3')

# generate heatmap
```

```r
m <- DEGs_DI_NASA_combined_cl3 %>%
  column_to_rownames(var = 'GeneSymbol') %>%
  select(day0Vsday0:`CD8_Post-flight vs Pre-flight`)

cell_type <- c(rep('CD3+',5), rep('CD4+',3), rep('CD8+',3))

comparison <- gsub('Vs', ' Vs ', colnames(m))
comparison <- gsub(' vs ', ' Vs ', comparison)
comparison <- gsub('CD\\d+_', '', comparison)

ha_top <- HeatmapAnnotation(
  Cell_type = factor(cell_type, levels = unique(cell_type)),
  Comparison = factor(comparison, levels = unique(comparison)),
  col = list(
    Cell_type = setNames(c('#E54D34','#9A509F','#77A2D5'),
                         nm = unique(cell_type)),
    Comparison = setNames(c(colPals$time,'#826F99','#18A38A','#395982'),
                         nm = unique(comparison))
  ),
  annotation_name_gp = gpar(fontface = 'bold'),
  border = T
)

ha_right <- rowAnnotation(
  mark = anno_mark(at=which(rownames(m) %in% mark.genes),
                  labels = rownames(m)[which(rownames(m) %in% mark.genes)],
                  padding = unit(1,"mm"),
                  labels_gp = gpar(fontface = 'italic'))
)

p <- Heatmap(m, name = "log2FC",
             cluster_row_slices = F, cluster_rows = T,
             column_title = NULL, column_split=factor(cell_type, levels = unique(cell_type)), cluster_columns = F,
             col = circlize::colorRamp2(breaks=seq(-2, 2, length.out=21),
                                        colors=colorRampPalette(rev(colPals$RdBu))(21)),
             top_annotation = ha_top,
             right_annotation = ha_right,
             width = unit(110, "mm"),
             na_col = "grey80",
             show_row_names = F,
             show_row_dend = T, row_dend_width=unit(10, "mm"), row_gap = unit(2, "mm"),
             show_column_names = T, column_names_gp = gpar(fontsize = 10), column_gap = unit(2, "mm"),
             border = T)

draw(p, merge_legend = T, align_heatmap_legend = "heatmap_top")
```

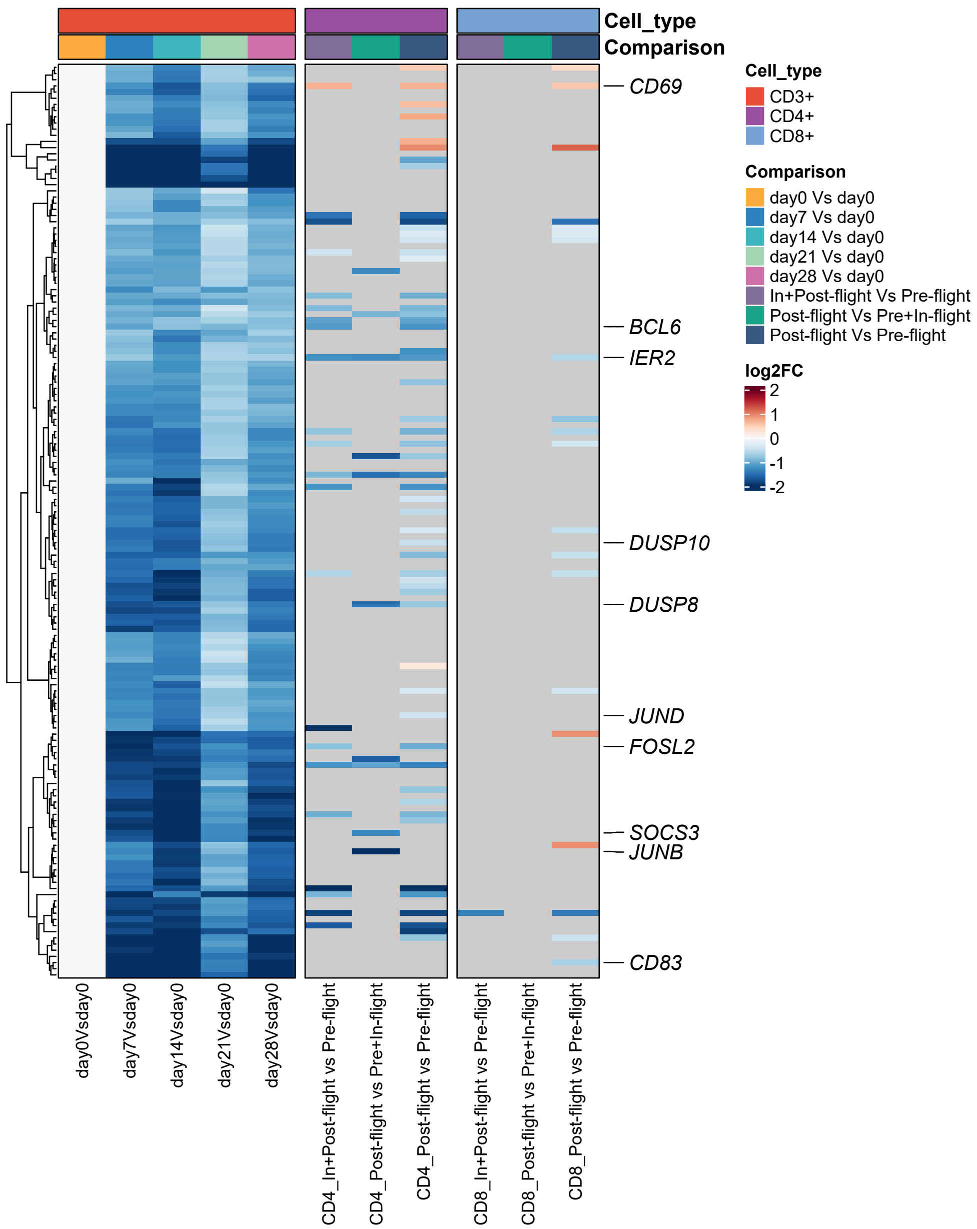


```
pdf("plots/fig3A_DEG_NASA_twin_study_comparison_downregulated.pdf", width = 10, height = 10)
draw(p, merge_legend = T, align_heatmap_legend = "heatmap_top")
dev.off()
```

```
## cairo_pdf
##         2
```

# Expression change of selected genes

```
selected_genes <- c('CD69','IER2','SOCS3','JUNB','FOSL2','DUSP8')

df <- DEGs_DI_NASA_combined_cl3 %>%
  filter(GeneSymbol %in% selected_genes) %>%
  pivot_longer(day0Vsday0:`CD8_Post-flight vs Pre-flight`,
               names_to = 'comparison',
               values_to = 'log2FC') %>%
  mutate(GeneSymbol = factor(GeneSymbol, levels = selected_genes),
         comparison = factor(comparison, levels = unique(comparison)),
         log2FC = ifelse(is.na(log2FC), 0, log2FC),
         type = 'unchanged') %>%
```

```
  mutate(type = ifelse(log2FC > 0, 'up', 'down')) %>%
  mutate(type = factor(type, levels = c('up', 'down', 'unchanged')))


ggplot(df, aes(x=comparison, y=log2FC, fill=type)) +
  geom_bar(stat='identity', size=1, width=0.6, color='black') +
  geom_hline(yintercept = 0, color='black', size=1) +
  scale_y_continuous(breaks = c(-2,0,2),
                     limits = c(-2.5,2.5),
                     expand = expansion(mult = c(.05, .01))) +
  scale_fill_manual(values = colPals$RdBu[c(2,10)]) +
  facet_wrap(~GeneSymbol, ncol=2) +
  xlab('') +
  ylab('Relative quantification (log2FC)') +
  theme_bw() +
  theme(
    text = element_text(family = 'Arial', size = 14),
    axis.text.x.bottom = element_text(angle=50, size = 12, hjust = 1, vjust = 1),
    axis.text.y.left = element_text(size = 12, hjust = 1, vjust = 0.3),
    panel.border = element_rect(color = "black", fill = NA, size = 1),
    axis.ticks = element_line(color = "black", size = 1),
    axis.ticks.length = unit(1.1, 'mm'),
    panel.grid.major.y = element_line(color = "grey80", linetype = "solid", size = 1),
    panel.grid.minor.y = element_blank(),
    panel.grid.major.x = element_blank(),
    panel.grid.minor.x = element_blank(),
    legend.position = 'none',
    strip.background = element_blank(),
    strip.text = element_text(face = "bold.italic")
  )
```

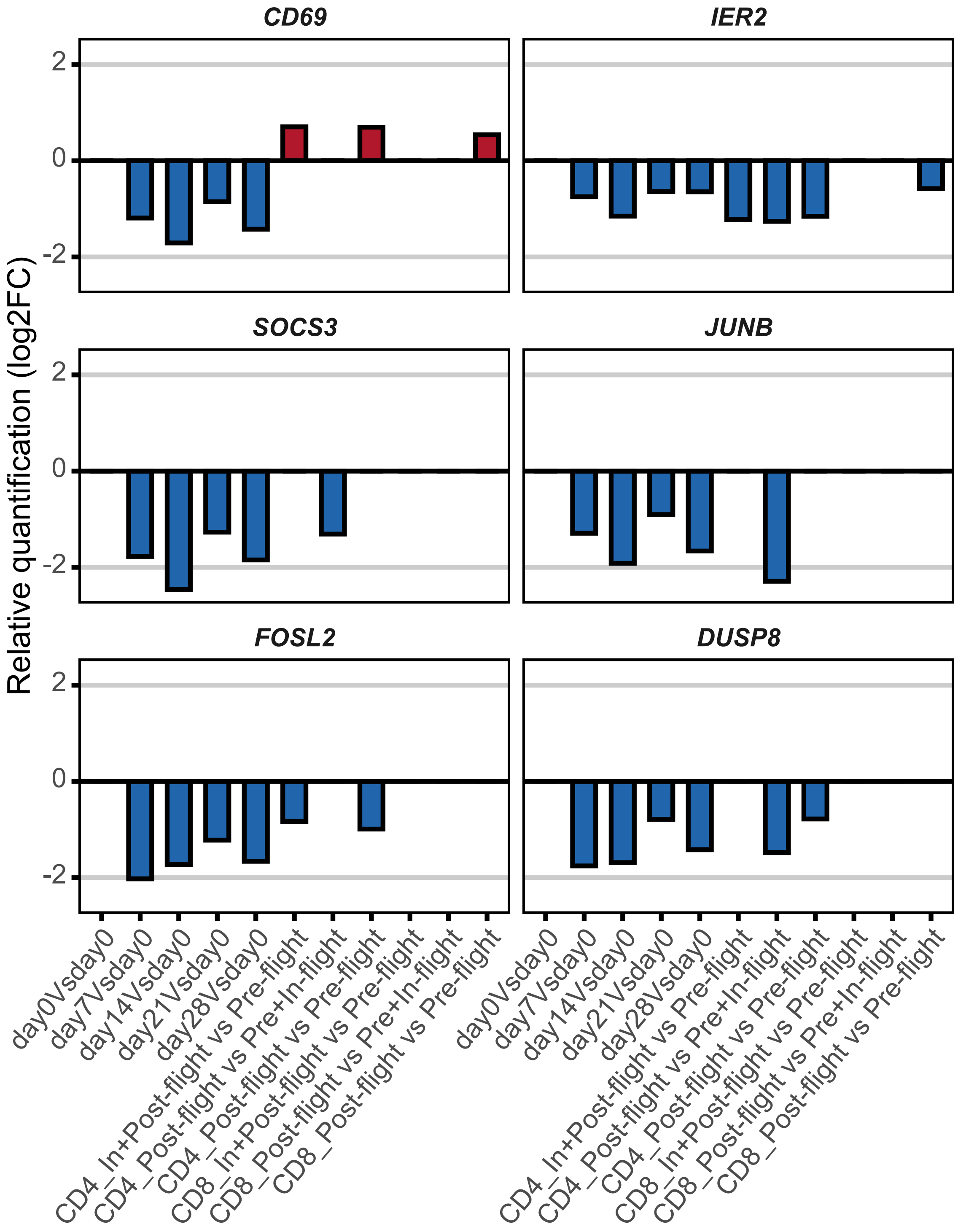


```
ggsave(filename = "plots/fig3B_DEG_NASA_twin_study_comparison_selected_log2FC.pdf", width = 6, height = 8, units = "in", dpi = 300, device = cairo
```

# Exports

```
openxlsx::write.xlsx(
  list(DEGs_DI_NASA_combined = DEGs_DI_NASA_combined),
  file = "tables/dataS7_DEGs_DI_NASA_combined.xlsx",
  rowNames=F,
  overwrite=T
)
```

# SessionInfo

```
sessionInfo()
```

```
## R version 4.2.1 (2022-06-23 ucrt)
## Platform: x86_64-w64-mingw32/x64 (64-bit)
## Running under: Windows 10 x64 (build 19044)
##
## Matrix products: default
##
## locale:
## [1] LC_COLLATE=English_United States.utf8
## [2] LC_CTYPE=English_United States.utf8
## [3] LC_MONETARY=English_United States.utf8
## [4] LC_NUMERIC=C
## [5] LC_TIME=English_United States.utf8
##
## attached base packages:
## [1] grid      stats     graphics  grDevices utils     datasets  methods
## [8] base
##
## other attached packages:
##  [1] ComplexHeatmap_2.14.0 openxlsx_4.2.5.1      RColorBrewer_1.1-3
##  [4] patchwork_1.1.2       magrittr_2.0.3        forcats_1.0.0
##  [7] stringr_1.5.0         dplyr_1.1.1           purrr_1.0.1
## [10] readr_2.1.4           tidyr_1.3.0           tibble_3.2.1
## [13] ggplot2_3.4.2         tidyverse_1.3.2
##
## loaded via a namespace (and not attached):
##  [1] httr_1.4.5          jsonlite_1.8.4      foreach_1.5.2
##  [4] modelr_0.1.11       highr_0.10          stats4_4.2.1
##  [7] googlesheets4_1.1.0 cellranger_1.1.0    yaml_2.3.7
## [10] pillar_1.9.0        backports_1.4.1     glue_1.6.2
## [13] digest_0.6.31       rvest_1.0.3         colorspace_2.1-0
## [16] htmltools_0.5.5     pkgconfig_2.0.3     GetoptLong_1.0.5
## [19] broom_1.0.4         haven_2.5.2         scales_1.2.1
## [22] tzdb_0.3.0          timechange_0.2.0    googledrive_2.1.0
## [25] farver_2.1.1        generics_0.1.3      IRanges_2.32.0
## [28] withr_2.5.0         BiocGenerics_0.44.0 cli_3.6.1
## [31] crayon_1.5.2        readxl_1.4.2        evaluate_0.20
## [34] fs_1.6.1            fansi_1.0.4         doParallel_1.0.17
## [37] xml2_1.3.3          tools_4.2.1         hms_1.1.3
## [40] GlobalOptions_0.1.2 gargle_1.3.0        lifecycle_1.0.3
## [43] matrixStats_0.63.0  S4Vectors_0.36.2    munsell_0.5.0
## [46] reprex_2.0.2        cluster_2.1.3       zip_2.2.2
## [49] compiler_4.2.1      rlang_1.1.0         iterators_1.0.14
## [52] rstudioapi_0.14     circlize_0.4.15     rjson_0.2.21
## [55] rmarkdown_2.21      gtable_0.3.3        codetools_0.2-18
## [58] DBI_1.1.3           R6_2.5.1            lubridate_1.9.2
## [61] knitr_1.42          fastmap_1.1.1       utf8_1.2.3
## [64] clue_0.3-64         shape_1.4.6         stringi_1.7.12
## [67] parallel_4.2.1      Rcpp_1.0.10         vctrs_0.6.1
## [70] png_0.1-8           dbplyr_2.3.2        tidyselect_1.2.0
## [73] xfun_0.38
```

# Step 4: Noncoding RNA analysis

Carlos Gallardo & Christian Oertlin

17 April, 2023

```
# Import libraries and helper functions
source("code/helper_functions.R")
library(tidyverse)
library(magrittr)
library(ggrepel)
library(RColorBrewer)
library(pheatmap)
library(viridis)
library(patchwork)
library(ComplexHeatmap)

# Colors
colPals <- vector(mode = "list")
colPals$time <- setNames(c("#FBAA3E", "#2C83BE", "#3EB6BD", "#A3D5B3", "#CD71A8"),
                         nm = c("day0", "day7", "day14", "day21", "day28"))
colPals$time_light <- setNames(c("#FDD6A1", "#A2CDE9", "#AFE2E5", "#DDF0E3", "#E8BDD6"),
                               nm = c("day0", "day7", "day14", "day21", "day28"))
colPals$time_dark <- setNames(c("#D87E04", "#174564", "#1F5C60", "#49A065", "#AA3C7E"),
                              nm = c("day0", "day7", "day14", "day21", "day28"))
colPals$inferno <- c("#000004", "#420A68", "#932667", "#DD513A", "#FCA50A", "#FCFFA4")
colPals$blood_cells <- setNames(c("#E54D34","#77A2D5","#B58B80"),
                                nm = c("granulocytes", "lymphocytes", "monocytes"))
colPals$cell_types <- setNames(c("#83D1F6","#FBAA3E","#FCCA7C","#B58B80","#E54D34",
                                 "#B3177E","#9A509F","#77A2D5","#CAC1DD","#36B449","#C1C1C1"),
                               nm = c("B cell", "Macrophage M1", "Macrophage M2",
                                      "Monocyte", "Neutrophil", "NK cell",
                                      "T cell CD4+ (non-regulatory)", "T cell CD8+",
                                      "T cell regulatory (Tregs)", "Myeloid dendritic cell",
                                      "uncharacterized cell"))
colPals$RdBu <- brewer.pal(11, name = "RdBu")
colPals$biotype <- setNames(c("#395982","#49BED9","#18A38A","#36B449","#826F99",
                              "#9852A5","#FBAA3E","#FCCA7C","#FCFFA4","#C1C1C1"),
                            nm = c("protein_coding", "lncRNA", "miRNA", "snoRNA",
                                   "IG_C_gene", "IG_V_gene", "TR_C_gene",
                                   "TR_J_gene", "TR_V_gene", "other"))
colPals$rtqpcr_rnaseq <- setNames(c("#B80D48","#2B6A6C"),
                                  nm = c("rtqpcr", "rnaseq"))
```

# Load data

```
# Gene expression and DEGs
RNAseq <- readRDS(file='data/rnaseq/rnaseq_volunteers_9&10_excl.rds')
DESeq2_DEGs <- readRDS(file='data/rnaseq/DESeq2_DEGs_unfilt_volunteers_9&10_excl.rds')
DESeq2_DEGs_filt <- readRDS(file='data/rnaseq/DESeq2_DEGs_filt_volunteers_9&10_excl.rds')

# Normalized k-mer counts
kmer_counts <- vector("list")

kmer_counts[["canonical_background_lncRNA_3mers"]] <- read.table(
  file = 'data/ncrna_analysis/kmer_counts/canonical_background_lncRNA_3mers.txt',
  sep = "\t",
  header = TRUE)

kmer_counts[["canonical_background_lncRNA_4mers"]] <- read.table(
  file = 'data/ncrna_analysis/kmer_counts/canonical_background_lncRNA_4mers.txt',
  sep = "\t",
  header = TRUE)

kmer_counts[["canonical_background_lncRNA_5mers"]] <- read.table(
  file = 'data/ncrna_analysis/kmer_counts/canonical_background_lncRNA_5mers.txt',
  sep = "\t",
```

```
  header = TRUE)

kmer_counts[["canonical_background_lncRNA_6mers"]] <- read.table(
  file = 'data/ncrna_analysis/kmer_counts/canonical_background_lncRNA_6mers.txt',
  sep = "\t",
  header = TRUE)
```

# Filter DE lncRNA from pairwise wald tests

```
# Pairwise comparisons
lncRNA <- RNAseq$unfilt$annotation %>%
  filter(gene_biotype == "lncRNA") %>%
  pull(ensembl_gene_id_version) %>%
  intersect(., lapply(DESeq2_DEGs_filt[2:5], function(x) x$ensembl_gene_id_version) %>% unlist() %>% unique())

kmer_counts_pairwise_DE <- lapply(kmer_counts, function(x) filter(x, Geneid %in% lncRNA))
kmer_counts_pairwise_DE <- lapply(kmer_counts_pairwise_DE, function(x) column_to_rownames(x, var = 'Geneid'))
```


```
# Pairwise comparisons
lncRNA <- RNAseq$unfilt$annotation %>%
  filter(gene_biotype == "lncRNA") %>%
  pull(ensembl_gene_id_version) %>%
  intersect(., lapply(DESeq2_DEGs_filt[2:5], function(x) x$ensembl_gene_id_version) %>% unlist() %>% unique())

kmer_counts_pairwise_DE <- lapply(kmer_counts, function(x) filter(x, Geneid %in% lncRNA))
kmer_counts_pairwise_DE <- lapply(kmer_counts_pairwise_DE, function(x) column_to_rownames(x, var = 'Geneid'))
```


```
kmer_counts_pairwise_DE_cor <- lapply(kmer_counts_pairwise_DE, function(x) cor(t(x), method = 'pearson'))
```

# Group lncRNA based on k-mer enrichment

```
p1 <- Heatmap(kmer_counts_pairwise_DE_cor$canonical_background_lncRNA_3mers,
        show_row_names = F,
        show_column_names = F,
        clustering_distance_rows = function(x) as.dist(1-x),
        clustering_distance_columns = function(x) as.dist(1-x),
        col = circlize::colorRamp2(breaks=seq(-0.8, 0.8, length.out=21),
                                   colors=colorRampPalette(rev(RColorBrewer::brewer.pal(n=11, name = "RdBu")))(21)))

p2 <- Heatmap(kmer_counts_pairwise_DE_cor$canonical_background_lncRNA_4mers,
        show_row_names = F,
        show_column_names = F,
        clustering_distance_rows = function(x) as.dist(1-x),
        clustering_distance_columns = function(x) as.dist(1-x),
        col = circlize::colorRamp2(breaks=seq(-0.6, 0.6, length.out=21),
                                   colors=colorRampPalette(rev(RColorBrewer::brewer.pal(n=11, name = "RdBu")))(21)))

p3 <- Heatmap(kmer_counts_pairwise_DE_cor$canonical_background_lncRNA_5mers,
        show_row_names = F,
        show_column_names = F,
        clustering_distance_rows = function(x) as.dist(1-x),
        clustering_distance_columns = function(x) as.dist(1-x),
        col = circlize::colorRamp2(breaks=seq(-0.4, 0.4, length.out=21),
                                   colors=colorRampPalette(rev(RColorBrewer::brewer.pal(n=11, name = "RdBu")))(21)))

p4 <- Heatmap(kmer_counts_pairwise_DE_cor$canonical_background_lncRNA_6mers,
        show_row_names = F,
        show_column_names = F,
        clustering_distance_rows = function(x) as.dist(1-x),
        clustering_distance_columns = function(x) as.dist(1-x),
        col = circlize::colorRamp2(breaks=seq(-0.2, 0.2, length.out=21),
                                   colors=colorRampPalette(rev(RColorBrewer::brewer.pal(n=11, name = "RdBu")))(21)))

(ggplotify::as.ggplot(p1) | ggplotify::as.ggplot(p2)) / (ggplotify::as.ggplot(p3) | ggplotify::as.ggplot(p4))
```

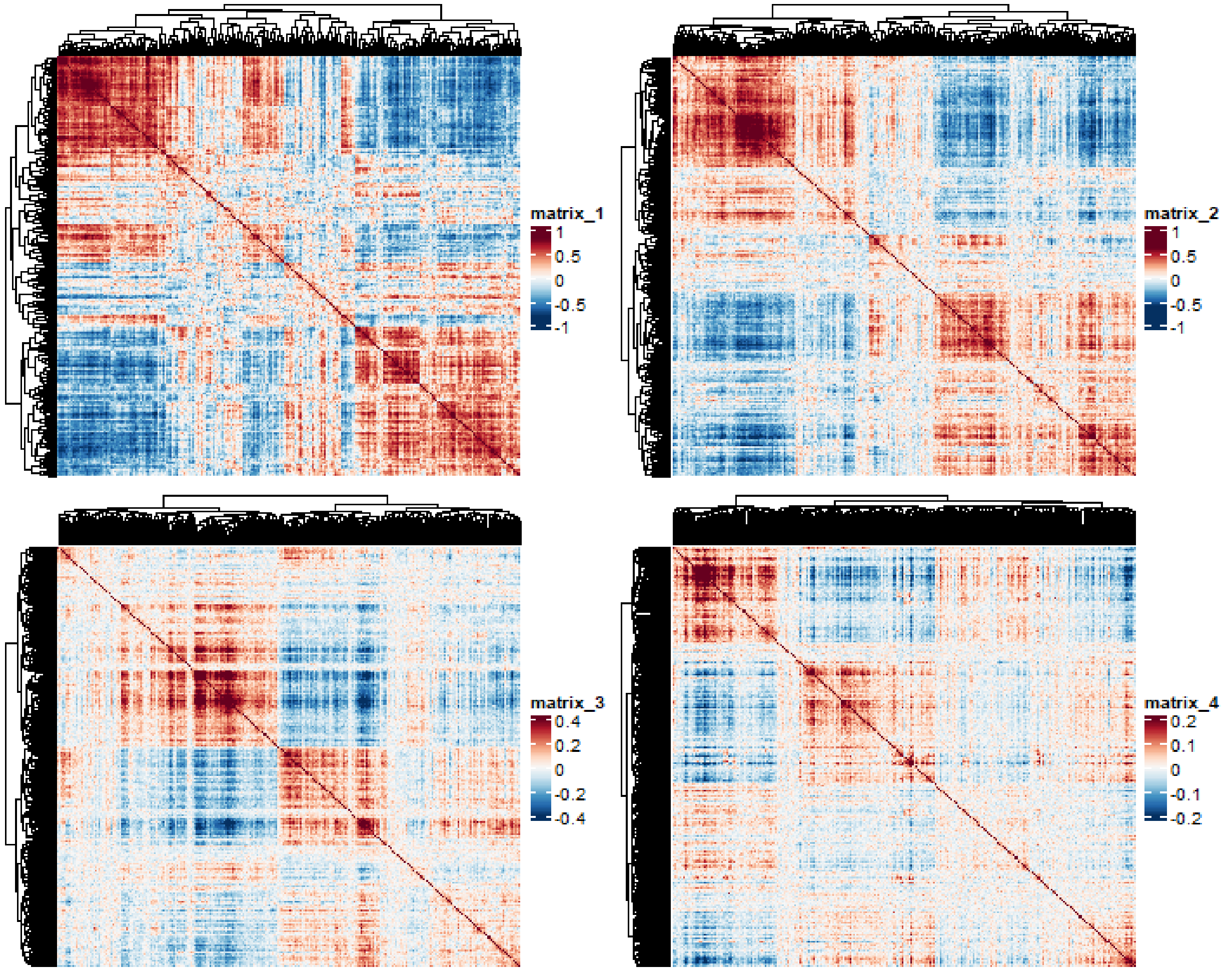


```
pdf("plots/figS7_lncRNA_cor_mat_3mers.pdf", width = 6, height = 5)
draw(p1)
dev.off()
```

```
## png
##   2
```

```
pdf("plots/figS7_lncRNA_cor_mat_4mers.pdf", width = 6, height = 5)
draw(p2)
dev.off()
```

```
## png
##   2
```

```
pdf("plots/figS7_lncRNA_cor_mat_5mers.pdf", width = 6, height = 5)
draw(p3)
dev.off()
```

```
## png
##   2
```

```
pdf("plots/figS7_lncRNA_cor_mat_6mers.pdf", width = 6, height = 5)
draw(p4)
dev.off()
```

```
## png
##   2
```

# Clustering on 6-mer enrichment profiles

```
mark_genes <- c('PVT1', 'MALAT1', 'PRANCR', 'DLEU2', 'HCG11', 'CHASERR', 'PDCD4-AS1', 'LINC00861')

set.seed(2)
```

```
lncRNA_cor_mtx <- cor(t(kmer_counts_pairwise_DE$canonical_background_lncRNA_6mers), method = 'pearson')
colnames(lncRNA_cor_mtx) <- plyr::mapvalues(x = colnames(lncRNA_cor_mtx),
                                            from = DESeq2_DEGs$day7Vsday0$ensembl_gene_id_version,
                                            to = DESeq2_DEGs$day7Vsday0$GeneSymbol,
                                            warn_missing = F)
rownames(lncRNA_cor_mtx) <- colnames(lncRNA_cor_mtx)
lncRNA_cl <-kmeans(kmer_counts_pairwise_DE$canonical_background_lncRNA_6mers, centers=3)
p1 <- Heatmap(lncRNA_cor_mtx,
              cluster_rows = T,
              cluster_row_slices = F,
              cluster_columns = T,
              cluster_column_slices = F,
              split = lncRNA_cl$cluster,
              column_split = lncRNA_cl$cluster,
              show_row_names = F,
              show_column_names = F,
              show_row_dend = T,
              show_column_dend = T,
              border = T,
              gap = unit(2, "mm"),
              column_gap = unit(2, "mm"),
              row_dend_gp = gpar(lwd=unit(0.1, "mm")),
              column_dend_gp = gpar(lwd=unit(0.1, "mm")),
              col = circlize::colorRamp2(breaks=seq(-0.2, 0.2, length.out=21),
                                         colors=colorRampPalette(rev(RColorBrewer::brewer.pal(n=11, name = "RdBu")))(21))
) +
  rowAnnotation(mark = anno_mark(at=which(rownames(lncRNA_cor_mtx) %in% mark_genes),
                                 labels = rownames(lncRNA_cor_mtx)[which(rownames(lncRNA_cor_mtx) %in% mark_genes)]))

p1
```

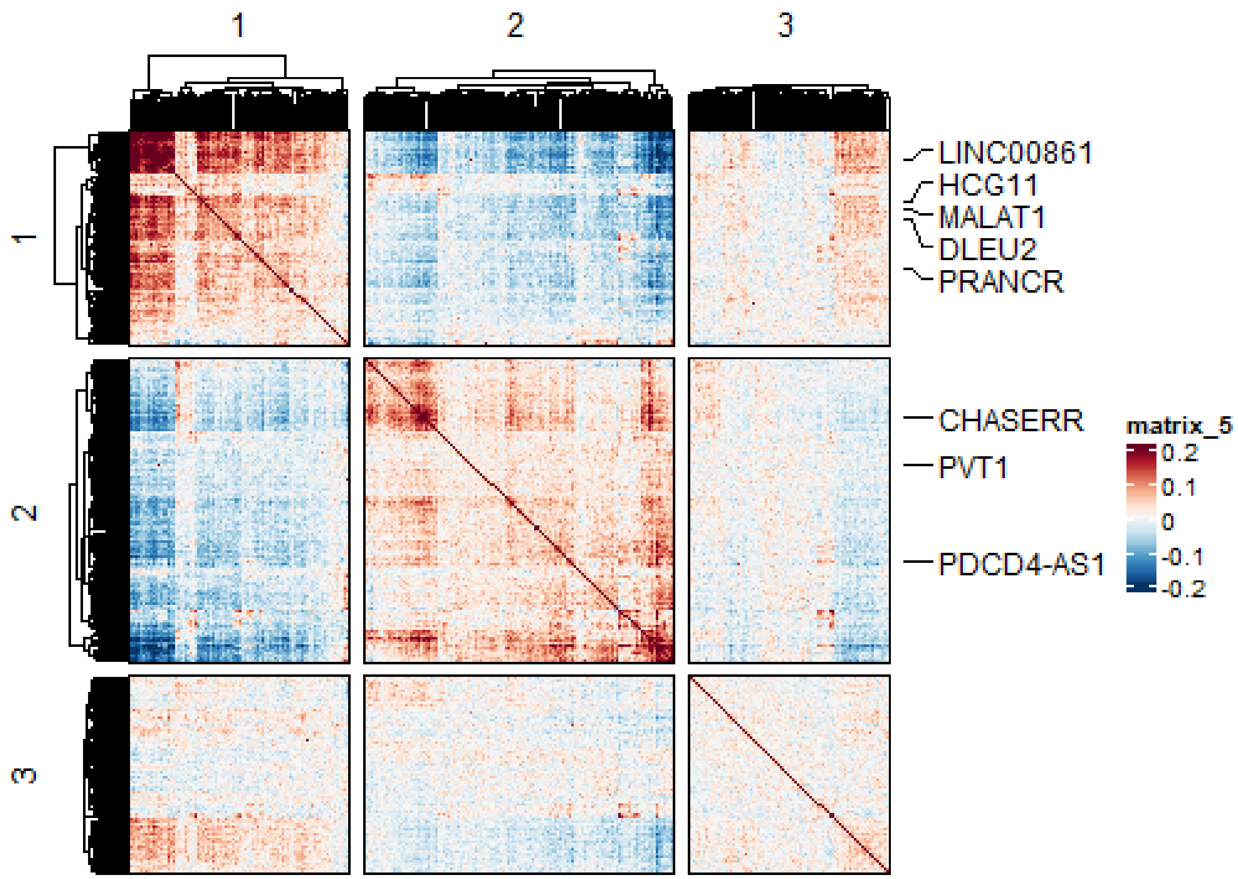


```
pdf("plots/fig4A_lncRNA_cor_mat_6mers_clusters.pdf", width = 7, height = 5)
draw(p1)
dev.off()
```

```
## png
##   2
```

# Expression plots for selected lncRNAs

```
RNAseq_expr_tpm <- RNAseq$filt$rawdata
rownames(RNAseq_expr_tpm) <- NULL
RNAseq_expr_tpm <- RNAseq_expr_tpm %>%
  column_to_rownames(var = 'Geneid_version') %>%
  select(-c('Geneid', 'Length', 'GeneSymbol')) %>%
  tpm.normalize(., RNAseq$filt$rawdata$Length) %>%
  rownames_to_column(var = 'Geneid_version')
colnames(RNAseq_expr_tpm) <- c('Geneid_version', paste(RNAseq$filt$design$volunteer,
                                                       RNAseq$filt$design$time,
                                                       sep = '_'))

lncRNA_expr <- as.data.frame(lncRNA_cl$cluster) %>%
  dplyr::rename(Cluster = 'lncRNA_cl$cluster') %>%
  rownames_to_column(var = 'Geneid_version') %>%
  left_join(RNAseq_expr_tpm, by = 'Geneid_version') %>%
  mutate(Geneid_version = plyr::mapvalues(x = Geneid_version,
                                          from = DESeq2_DEGs$day7Vsday0$ensembl_gene_id_version,
                                          to = DESeq2_DEGs$day7Vsday0$GeneSymbol,
                                          warn_missing = F)) %>%
  dplyr::rename(Gene = Geneid_version)

lncRNA_expr_mean <- lncRNA_expr %>%
  column_to_rownames(var = 'Gene') %>%
  select(-Cluster) %>%
  group.transform(group = RNAseq$filt$design$time,
                  FUN = function(x) apply(x, 1, mean)) %>%
  rownames_to_column(var = 'Gene') %>%
  add_column(Cluster = lncRNA_expr$Cluster, .after = 'Gene')

df <- lapply(setNames(mark_genes, mark_genes), function(x) {

  lncRNA_expr %>%
    filter(Gene == x) %>%
    column_to_rownames(var = 'Gene') %>%
    select(-Cluster) %>%
    t() %>%
    as.data.frame() %>%
    dplyr::rename(TPM = x) %>%
    mutate(time = RNAseq$filt$design$time,
           gene = x)

}) %>% bind_rows()

df2 <- lapply(setNames(mark_genes, mark_genes), function(x) {

  lncRNA_expr_mean %>%
    filter(Gene == x) %>%
    column_to_rownames(var = 'Gene') %>%
    select(-Cluster) %>%
    t() %>%
    as.data.frame() %>%
    dplyr::rename(TPM = x) %>%
    rownames_to_column(var = 'time') %>%
    mutate(gene = x)

}) %>% bind_rows()

ggplot(df, aes(x=time, y=TPM, color=time)) +
  geom_point(shape=16, size=3, stroke=0, alpha=0.4) +
  facet_wrap(~gene, ncol=4, scales = 'free') +
  geom_line(data = df2, aes(x=time, y=TPM, group=1), color='black', size = 1.25) +
  geom_point(data = df2, aes(x=time, y=TPM, group=1, fill=time),
             shape=21, size=3.5, stroke=1.25, color='black') +
  xlab('') +
  ylab('Transcripts per million (TPM)') +
  scale_color_manual(values = colPals$time) +
  scale_fill_manual(values = colPals$time) +
  guides(color = F) +
  theme_bw() +
  theme(
    text = element_text(family = 'Arial', size = 14),
    axis.text.x.bottom = element_text(angle = 90, hjust = 1, vjust = 0.5),
    panel.border = element_rect(color = "black", fill = NA, size = 2),
    axis.ticks = element_line(color = "black", size = 1.25),
    axis.ticks.length = unit(1.5, 'mm'),
    panel.grid.major.x = element_blank(),
    panel.grid.minor.x = element_blank(),
```

```
    panel.grid.major.y = element_blank(),
    panel.grid.minor.y = element_blank(),
    legend.position = 'top',
    strip.background = element_blank(),
    strip.text = element_text(face = "bold.italic"),
    strip.text.y = element_text(angle = 90)
  )
```

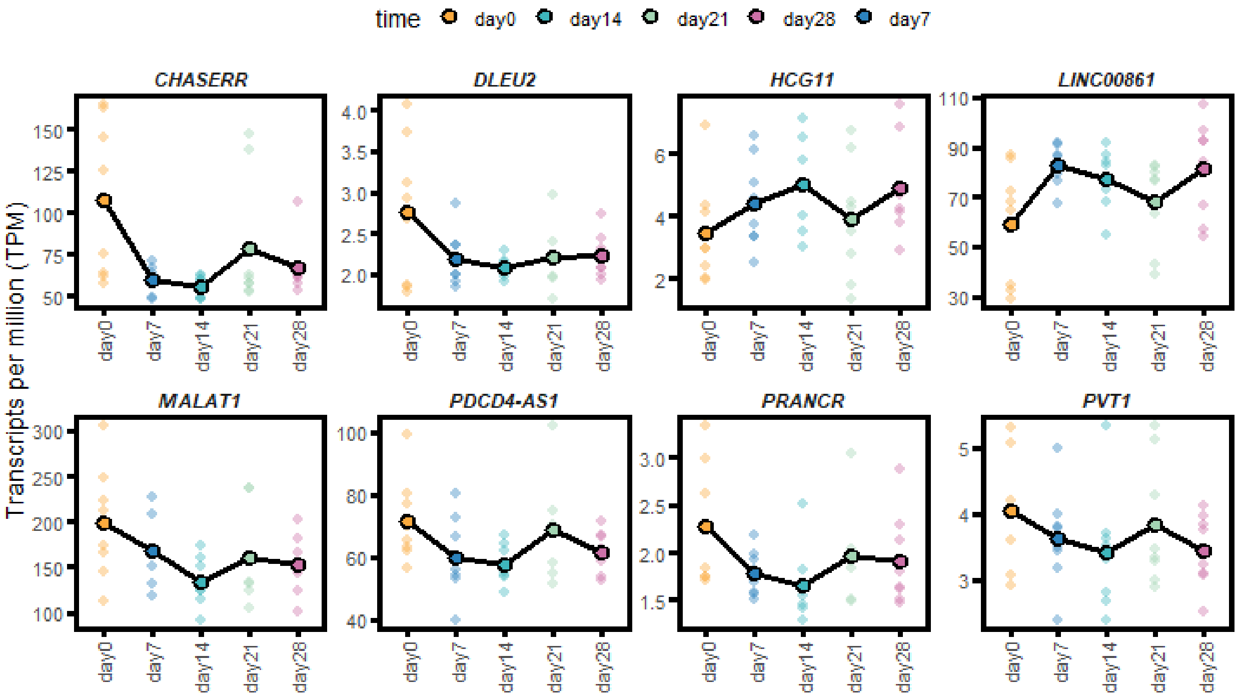


```
ggsave("plots/fig4B_lncRNA_selected_expression_tpm.pdf", width = 10, height = 6, units = "in", dpi = 300, device = cairo_pdf)
```

# Motif enrichemnt in lncRNA clusters

```
lncRNA <- rownames(kmer_counts_pairwise_DE$canonical_background_lncRNA_6mers)
lncRNA_cl1 <- names(lncRNA_cl$cluster[lncRNA_cl$cluster == 1])
lncRNA_cl2 <- names(lncRNA_cl$cluster[lncRNA_cl$cluster == 2])
lncRNA_cl3 <- names(lncRNA_cl$cluster[lncRNA_cl$cluster == 3])

lncRNA_cl1_pwm <- kmer_counts_pairwise_DE$canonical_background_lncRNA_6mers[lncRNA %in% lncRNA_cl1, ] %>%
  colSums(.) %>%
  sort(., decreasing = T) %>%
  head(100) %>%
  names(.) %>%
  paste0(., collapse = "") %>%
  str_split(., pattern = '') %>%
  unlist() %>%
  matrix(., ncol = 6, byrow = T) %>%
  apply(., MARGIN = 2, FUN = function(x) table(x)) %>%
  apply(., MARGIN = 2, FUN = function(x) x/100)

lncRNA_cl2_pwm <- kmer_counts_pairwise_DE$canonical_background_lncRNA_6mers[lncRNA %in% lncRNA_cl2, ] %>%
  colSums(.) %>%
  sort(., decreasing = T) %>%
  head(100) %>%
  names(.) %>%
  paste0(., collapse = "") %>%
  str_split(., pattern = '') %>%
  unlist() %>%
  matrix(., ncol = 6, byrow = T) %>%
  apply(., MARGIN = 2, FUN = function(x) table(x)) %>%
  apply(., MARGIN = 2, FUN = function(x) x/100)
```

```r
lncRNA_cl3_pwm <- kmer_counts_pairwise_DE$canonical_background_lncRNA_6mers[lncRNA %in% lncRNA_cl3, ] %>%
  colSums(.) %>%
  sort(., decreasing = T) %>%
  head(100) %>%
  names(.) %>%
  paste0(., collapse = "") %>%
  str_split(., pattern = '') %>%
  unlist() %>%
  matrix(., ncol = 6, byrow = T) %>%
  apply(., MARGIN = 2, FUN = function(x) table(x)) %>%
  apply(., MARGIN = 2, FUN = function(x) x/100)

lncRNA_cl1_pwm
```

```
##   [,1] [,2] [,3] [,4] [,5] [,6]
## A 0.30 0.35 0.40 0.35 0.32 0.36
## C 0.08 0.03 0.04 0.03 0.04 0.07
## G 0.09 0.09 0.09 0.09 0.10 0.11
## T 0.53 0.53 0.47 0.53 0.54 0.46
```

```r
lncRNA_cl2_pwm
```

```
##   [,1] [,2] [,3] [,4] [,5] [,6]
## A 0.06 0.07 0.06 0.08 0.02 0.04
## C 0.46 0.51 0.49 0.44 0.44 0.47
## G 0.41 0.39 0.41 0.44 0.49 0.45
## T 0.07 0.03 0.04 0.04 0.05 0.04
```

```r
lncRNA_cl3_pwm
```

```
##   [,1] [,2] [,3] [,4] [,5] [,6]
## A 0.38 0.46 0.33 0.37 0.40 0.43
## C 0.12 0.15 0.17 0.20 0.20 0.12
## G 0.21 0.15 0.25 0.18 0.29 0.24
## T 0.29 0.24 0.25 0.25 0.11 0.21
```

```r
rownames(lncRNA_cl1_pwm) <- gsub('T','U', rownames(lncRNA_cl1_pwm))
rownames(lncRNA_cl2_pwm) <- gsub('T','U', rownames(lncRNA_cl2_pwm))
rownames(lncRNA_cl3_pwm) <- gsub('T','U', rownames(lncRNA_cl3_pwm))

p1 <- ggseqlogo::ggseqlogo(lncRNA_cl1_pwm, method = 'bits' ) +
  theme_classic(base_size = 25, base_family = 'Arial')

p2 <- ggseqlogo::ggseqlogo(lncRNA_cl2_pwm, method = 'bits' ) +
  theme_classic(base_size = 25, base_family = 'Arial')

p3 <- ggseqlogo::ggseqlogo(lncRNA_cl3_pwm, method = 'bits' ) +
  theme_classic(base_size = 25, base_family = 'Arial')

p1/p2/p3
```

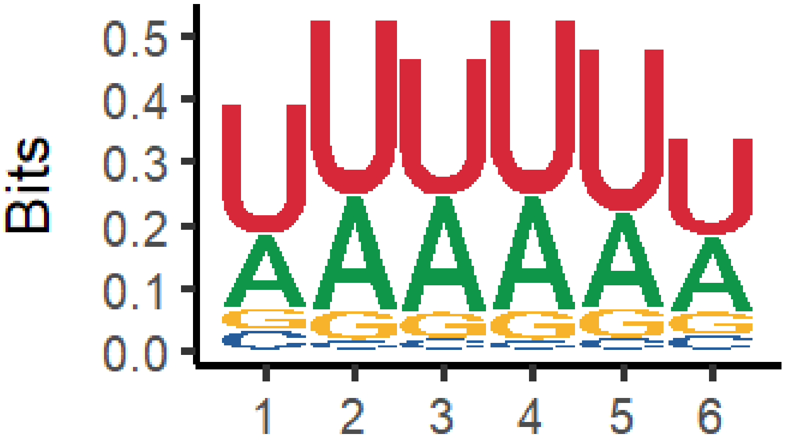
Bits
0.0
0.1
0.2
0.3
0.4
0.5
1 2 3 4 5 6

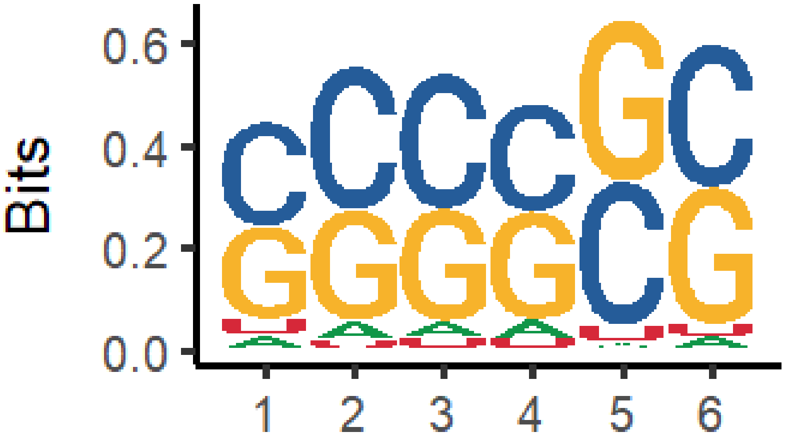
Bits
0.0
0.2
0.4
0.6
1 2 3 4 5 6

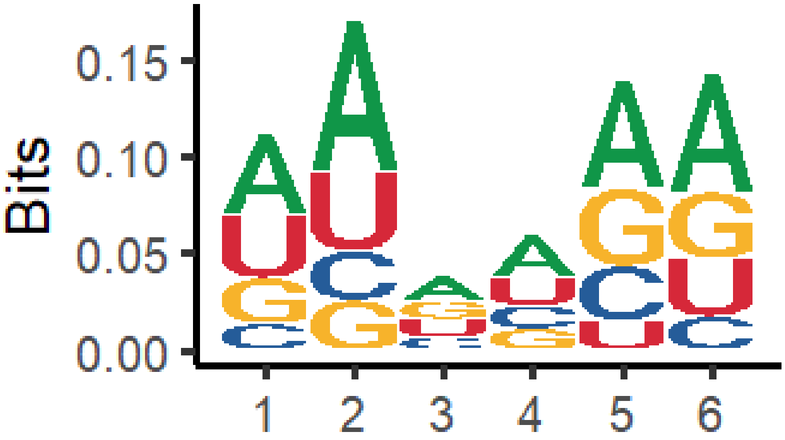
Bits
0.00
0.05
0.10
0.15
1 2 3 4 5 6

```r
ggsave("plots/fig4C_lncRNA_6mers_logo_cl1_withUs.pdf", plot = p1, width = 6, height = 4, units = "in", dpi = 300, device = cairo_pdf)
ggsave("plots/fig4D_lncRNA_6mers_logo_cl2_withUs.pdf", plot = p2, width = 6, height = 4, units = "in", dpi = 300, device = cairo_pdf)
ggsave("plots/fig4E_lncRNA_6mers_logo_cl3_withUs.pdf", plot = p3, width = 6, height = 4, units = "in", dpi = 300, device = cairo_pdf)
```

# SessionInfo

```r
sessionInfo()
```

```
## R version 4.2.1 (2022-06-23 ucrt)
## Platform: x86_64-w64-mingw32/x64 (64-bit)
## Running under: Windows 10 x64 (build 19044)
##
## Matrix products: default
##
## locale:
## [1] LC_COLLATE=English_United States.utf8
## [2] LC_CTYPE=English_United States.utf8
## [3] LC_MONETARY=English_United States.utf8
## [4] LC_NUMERIC=C
## [5] LC_TIME=English_United States.utf8
##
## attached base packages:
## [1] grid      stats     graphics  grDevices utils     datasets  methods
## [8] base
##
## other attached packages:
##  [1] ComplexHeatmap_2.14.0 patchwork_1.1.2       viridis_0.6.2
##  [4] viridisLite_0.4.1     pheatmap_1.0.12       RColorBrewer_1.1-3
##  [7] ggrepel_0.9.3         magrittr_2.0.3        forcats_1.0.0
## [10] stringr_1.5.0         dplyr_1.1.1           purrr_1.0.1
## [13] readr_2.1.4           tidyr_1.3.0           tibble_3.2.1
## [16] ggplot2_3.4.2         tidyverse_1.3.2
##
## loaded via a namespace (and not attached):
##  [1] matrixStats_0.63.0  fs_1.6.1            lubridate_1.9.2
##  [4] doParallel_1.0.17   httr_1.4.5          tools_4.2.1
##  [7] backports_1.4.1     utf8_1.2.3          R6_2.5.1
## [10] DBI_1.1.3           BiocGenerics_0.44.0 colorspace_2.1-0
## [13] GetoptLong_1.0.5    withr_2.5.0         tidyselect_1.2.0
## [16] gridExtra_2.3       compiler_4.2.1      cli_3.6.1
## [19] rvest_1.0.3         xml2_1.3.3          labeling_0.4.2
## [22] scales_1.2.1        digest_0.6.31       yulab.utils_0.0.6
## [25] rmarkdown_2.21      pkgconfig_2.0.3     htmltools_0.5.5
## [28] dbplyr_2.3.2        fastmap_1.1.1       highr_0.10
## [31] rlang_1.1.0         GlobalOptions_0.1.2 readxl_1.4.2
## [34] rstudioapi_0.14     shape_1.4.6         gridGraphics_0.5-1
## [37] generics_0.1.3      farver_2.1.1        jsonlite_1.8.4
## [40] googlesheets4_1.1.0 ggplotify_0.1.0     Rcpp_1.0.10
## [43] munsell_0.5.0       S4Vectors_0.36.2    fansi_1.0.4
## [46] lifecycle_1.0.3     stringi_1.7.12      yaml_2.3.7
## [49] plyr_1.8.8          ggseqlogo_0.1       parallel_4.2.1
## [52] crayon_1.5.2        haven_2.5.2         circlize_0.4.15
## [55] hms_1.1.3           knitr_1.42          pillar_1.9.0
## [58] rjson_0.2.21        codetools_0.2-18    stats4_4.2.1
## [61] reprex_2.0.2        glue_1.6.2          evaluate_0.20
## [64] modelr_0.1.11       png_0.1-8           vctrs_0.6.1
## [67] tzdb_0.3.0          foreach_1.5.2       cellranger_1.1.0
## [70] gtable_0.3.3        clue_0.3-64         xfun_0.38
## [73] broom_1.0.4         googledrive_2.1.0   gargle_1.3.0
## [76] iterators_1.0.14    IRanges_2.32.0      cluster_2.1.3
## [79] timechange_0.2.0
```
